# Circular RNA expression profiles in umbilical cord blood exosomes from normal and gestational diabetes mellitus patients

**DOI:** 10.1042/BSR20201946

**Published:** 2020-11-17

**Authors:** Minkai Cao, Le Zhang, Yu Lin, Zhengying Li, Jianjuan Xu, Zhonghua Shi, Zhong Chen, Jinqi Ma, Juan Wen

**Affiliations:** 1 Department of Obstetrics, The Affiliated Wuxi Maternity and Child Health Care Hospital of Nanjing Medical University, Wuxi 214002, China; 2 Department of Neonatology, The Affiliated Wuxi Children’s Hospital of Nanjing Medical University, Wuxi 214023, China; 3 Nanjing Maternity and Child Health Care Institute, Women’s Hospital of Nanjing Medical University, Nanjing Maternity and Child Health Care Hospital, Nanjing 210004, China; 4 Department of Obstetrics, Women’s Hospital of Nanjing Medical University, Nanjing Maternity and Child Health Care Hospital, Nanjing 210004, China; 5 Department of Gynaecology and Obstetrics, The Affiliated Wuxi People’s Hospital of Nanjing Medical University, Wuxi 214023, China

**Keywords:** circular RNA, exosomes, gestational diabetes mellitus, umbilical cord blood

## Abstract

Circular RNA (circRNA) is a novel member of endogenous noncoding RNAs with widespread distribution and diverse cellular functions. Recently, circRNAs have been identified for their enrichment and stability in exosomes. However, the roles of circRNAs from umbilical cord blood exosomes in gestational diabetes mellitus (GDM) occurrence and fetus growth remains poorly understood. In the present study, we used microarray technology to construct a comparative circRNA profiling of umbilical cord blood exosomes between GDM patients and controls. We found the exosome particle size was larger, and the exosome concentration was higher in the GDM patients. A total of 88,371 circRNAs in umbilical cord blood exosomes from two groups were evaluated. Of these, 229 circRNAs were significantly up-regulated and 278 circRNAs were significantly down-regulated in the GDM patients. Gene Ontology (GO) and Kyoto Encyclopedia of Genes and Genomes (KEGG) biological pathway analyses demonstrated that circRNA parental genes involved in the regulation of metabolic process, growth and development were significantly enriched, which are important in GDM development and fetus growth. Further circRNA/miRNA interactions analysis showed that most of the exosomal circRNAs harbored miRNA binding sites, and some miRNAs were associated with GDM. Collectively, these results lay a foundation for extensive studies on the role of exosomal circRNAs in GDM development and fetus growth.

## Introduction

Gestational diabetes mellitus (GDM) is defined as glucose intolerance with onset or first recognition during pregnancy, which has become one of most common complications of pregnancy [1]. According to the latest statistics of International Diabetes Federation in 2019, one in six live births worldwide is affected by hyperglycemia during pregnancy (http://www.diabetesatlas.org). In recent years, with the improvement of China's living standard and the implementation of the ´universal two-child policy’, there are more and more overweight elderly pregnant women, resulting in a sharp rise in the incidence of GDM in China [2]. Accumulating evidences suggested that GDM patients have a greater risk of pregnancy complications, such as cesarean delivery, shoulder dystocia, macrosomia, and neonatal hypoglycemia [3]. After delivery, the GDM patients also have an elevated risk for Type 2 diabetes (T2D) and metabolic syndrome [4]. For the offspring of GDM mothers, the adverse maternal environment may increase the risk of obesity, T2D and cardiovascular disease, later in life [5]. The theory of ´Developmental Origin of Health and Disease (DOHaD)’ suggested that, the nutrition and nurturing environment in the first 1000 days of life (such as the intrauterine high-glucose environment) is crucial to the health of individuals throughout their lives [6]. During fetal development, the umbilical vein transport oxygenated blood with nutrition and other factors from placenta to the developing fetus. Changes of certain nutrients and factors in umbilical cord blood may have a crucial role in fetal programming [7]. Study on the substance contained in the umbilical cord blood may therefore help us to understand the influence of GDM on fetal development.

Exosomes have emerged as critical mediators of intercellular communication, both locally and systemically, by regulating a diverse range of biological processes between cells [8,9]. Many studies have reported that exosomes play an important role in the regulation of pregnancy complications such as GDM and preeclampsia [10,11]. Specific exosomes derived from placenta can be detected in maternal blood as early as the 6th week of gestation, and the concentration of placenta-derived exosomes increases with the progress of gestation [12]. Moreover, exosomes isolated from maternal blood have biological activity *in vitro* and can get inside of the target cells through endocytosis [13]. For GDM patients in early, middle and late pregnancy, the concentration of placenta-derived exosomes was significantly higher than that of normal pregnant women at the same period [12], suggesting that maternal high glucose can stimulate placenta to release exosomes to circulation. Circular RNA (circRNA) is a novel member of endogenous noncoding RNAs with widespread distribution and diverse cellular functions. Recently, circRNAs have been identified for their enrichment and stability in exosomes [14,15]. An increasing number of studies have shown that exosomal circRNAs participate in the processes of cell growth, angiogenesis, epithelial–mesenchymal transition, and targeted therapy [16,17]. Therefore, we speculate that exosomal circRNA in umbilical cord blood may play an important role in regulating GDM and fetal growth/development as a new placenta-derived factor. However, the roles of exosomal circRNAs from umbilical cord blood in GDM occurrence and fetus growth remains poorly understood. In the present study, we used microarray technology to construct a comparative circRNA profiling of umbilical cord blood exosomes between GDM patients and controls, aimed to lay a foundation for extensive studies on the role of exosomal circRNAs in GDM development and fetus growth.

## Materials and methods

### Patients and sample collection

All participants and clinical information were collected at the Nanjing Maternity and Child Health Care Hospital, Wuxi Maternity and Child Health Care Hospital and Wuxi People’s Hospital from August 2018 to July 2019. For all participants between 24 and 28 weeks of gestation, a glucose challenge test (GCT) was conducted. The GDM cases were defined as pregnant woman with fasting blood glucose ≥ 5.5 mmol/l or 2 h plasma glucose ≥ 8.0 mmol/l following a 75 g oral glucose tolerance test (OGTT) [18]. Participants diagnosed with metabolic syndrome and related diseases before pregnancy were excluded from the present study. The pregnant women without diabetes were included as controls. The controls were matched to GDM cases for maternal age and gestational age. At last, 46 umbilical cord blood samples were collected from the umbilical vein immediately after delivery of fetus during cesarean section (23 GDM patients and 23 controls) according to the standard operating procedure. All participants were divided into two sets, 6 participants (3 GDM patients and 3 controls) for microarray screening and 40 participants (20 GDM patients and 20 controls) for validation.

### Exosome purification and analysis

Exosomes were prepared from the umbilical cord blood. Briefly, umbilical cord blood was centrifuged at 3000 ***g*** for 15 min at 4°C. Supernatants were then centrifuged at 12,000 ***g*** for 30 min at 4°C. Then supernatants were filtered through 0.45 μm polyvinylidene fluoride (PVDF) membrane, and isolated in a final ultracentrifugation at 100,000 ***g*** for 180 min at 4°C. The exosome pellet was resuspended in PBS or lysis buffer. The resulting exosomes were next analyzed with the Nanosight Nano ZS device (Malvern Instruments, U.K.).

### Exosomal RNA extraction and microarray analysis

Total exosomal RNA was extracted using Serum/Plasma Kit (QIAGEN, Germany) according to the manufacturer’s instructions and checked for RNA integration by an Agilent Bioanalyzer 2100 (Agilent technologies, U.S.A.). Total RNA was amplified and labeled by Low Input Quick Amp Labeling Kit (Agilent technologies, U.S.A.), following the manufacturer’s instructions. Labeled cRNA was purified by RNeasy mini kit (QIAGEN, Germany). Each slide was hybridized with 1.65 μg Cy3-labeled cRNA using Gene Expression Hybridization Kit (Agilent technologies, U.S.A.) in Hybridization Oven (Agilent technologies, U.S.A.), according to the manufacturer’s instructions. After 17 h hybridization, slides were washed in staining dishes (Thermo Shandon, U.S.A.) with Gene Expression Wash Buffer Kit (Agilent technologies, U.S.A.), followed the manufacturer’s instructions. Slides were scanned by Agilent Microarray Scanner (Agilent technologies, U.S.A.) with default settings. Data were extracted with Feature Extraction software 12.0 (Agilent technologies, U.S.A.). Raw data were normalized by Quantile algorithm, limma packages in R.

### Quantitative real-time PCR (qPCR)

The specific divergent primers were designed using Primer-BLAST to amplify the circular transcripts through head-to-tail splicing. All the primers were synthesized with Realgene (Nanjing, China). The primer sequences are listed in Supplementary Table S1.

After determining the best annealing temperatures, qPCR was performed to measure the relative circRNA expression levels using PowerUP SYBR Green Master Mix (Applied Biosystems, U.S.A.) on a Life Tech-ViiA7 system (Applied Biosystems, U.S.A.). Glyceraldehyde phosphate dehydrogenase (GAPDH) served as the internal control, the relative expression level of each circRNA was calculated with the 2^−△△Ct^ method.

### Functional enrichment analyses

The parental gene functions of the differentially expressed circRNAs were analyzed using DAVID Bioinformatics Resources 6.8. Gene Ontology (GO) analysis of the parental genes was performed based on three terms, namely, biological processes, cellular components and molecular functions (http://www.geneontology.org/). The related biological pathways were analyzed by Kyoto Encyclopedia of Genes and Genomes (KEGG, http://www.genome.jp/kegg). We regarded the -log (*P*-value) as the enrichment score that indicated the significance of correlation.

### Annotation for circRNA/miRNA interactions

The miRanda (http://www.microrna.org/microrna/home.do/) was used to predict circRNA/miRNA interactions. With the database, we searched miRNA response elements (MREs) on circRNAs, and selected miRNAs based on the seed-match sequences.

### Statistical analyses

Statistical analyses were performed with SPSS 18.0 and GraphPad prism 5.0. All the data are displayed as the mean ± standard deviation (SD). Student’s *t*-test was used to assess the differences between experimental groups. Differences with *P*-values < 0.05 were considered statistically significant.

## Results

### The particle size and concentration of exosomes were correlated with GDM

**Figure 1:**
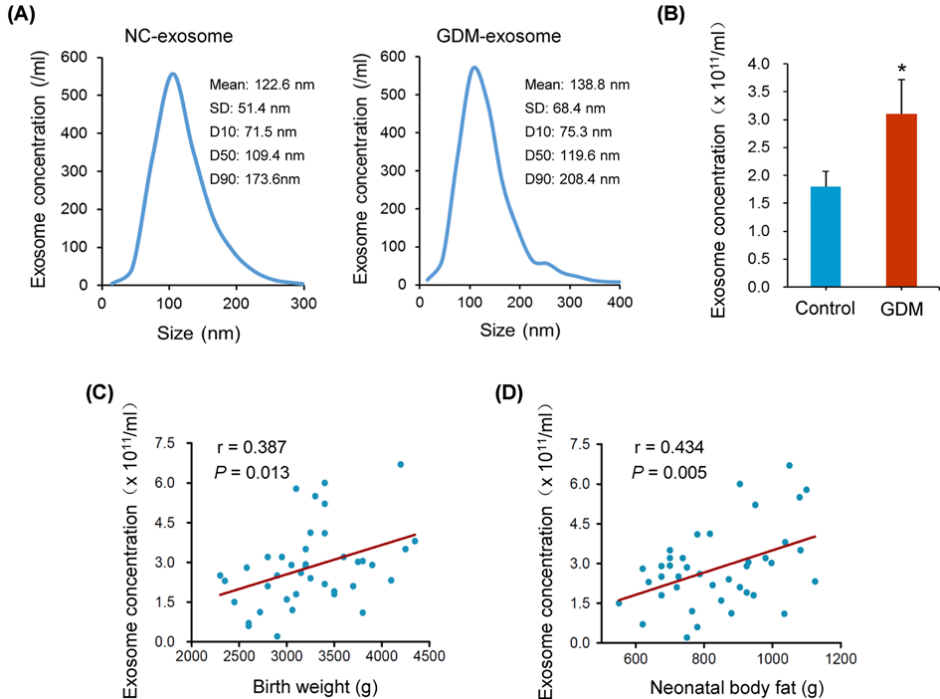
The particle size and concentration of exosomes were correlated with GDM (**A**) The particle size of umbilical cord blood exosomes from GDM patients and controls. (**B**) The concentration of umbilical cord blood exosomes from GDM patients and controls. (**C** and **D**) Exosome concentration was positively associated with neonatal birth weight and body fat. *P<0.05.

We analyzed the particle size and concentration of exosomes in umbilical cord blood from 3 GDM patients and 3 controls. The results showed that the particle size was significantly larger (*P*<0.001), and the exosome concentration was significantly higher in the GDM patients (*P*=0.012), when compared with controls (Figure 1A,B). We also analyzed the association of exosome concentration in umbilical cord blood with neonatal birth weight and body fat in 20 GDM patients and 20 controls. The basic information of the 40 subjects was shown in Supplementary Table S2. We found the exosome concentration was positively associated with neonatal birth weight and body fat (birth weight: *r* = 0.387, *P*=0.013; neonatal body fat: *r* = 0.434, *P*=0.005; Figure 1C,D).

### circRNA expression profiling in umbilical cord blood exosomes from GDM patients compared with controls

**Figure 2:**
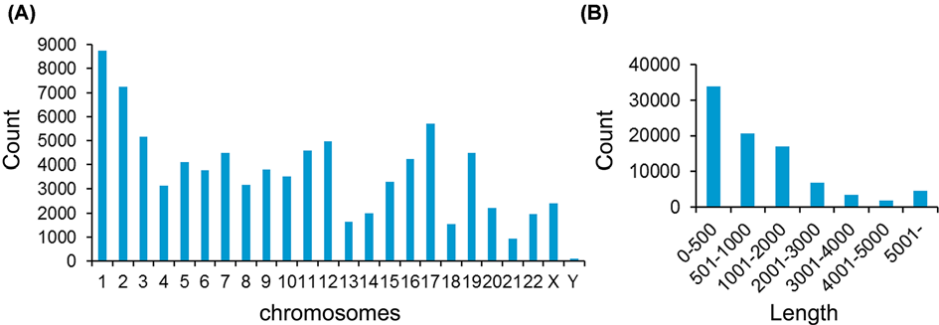
General characteristics of circRNAs in umbilical cord blood exosomes (**A**) Chromosomal distribution of all circRNAs. (**B**) Length distributions of all circRNAs.

To study the general characteristics of all the circRNAs in umbilical cord blood exosomes, we performed a preliminary analysis of all the microarray data. A total of 88,371 circRNAs were evaluated, known in circBase (http://circrna.org/). Figure 2A shows that these circRNA parental genes are widely scattered on almost all human chromosomes, and Figure 2B depicts the length distributions of these circRNAs.

**Figure 3:**
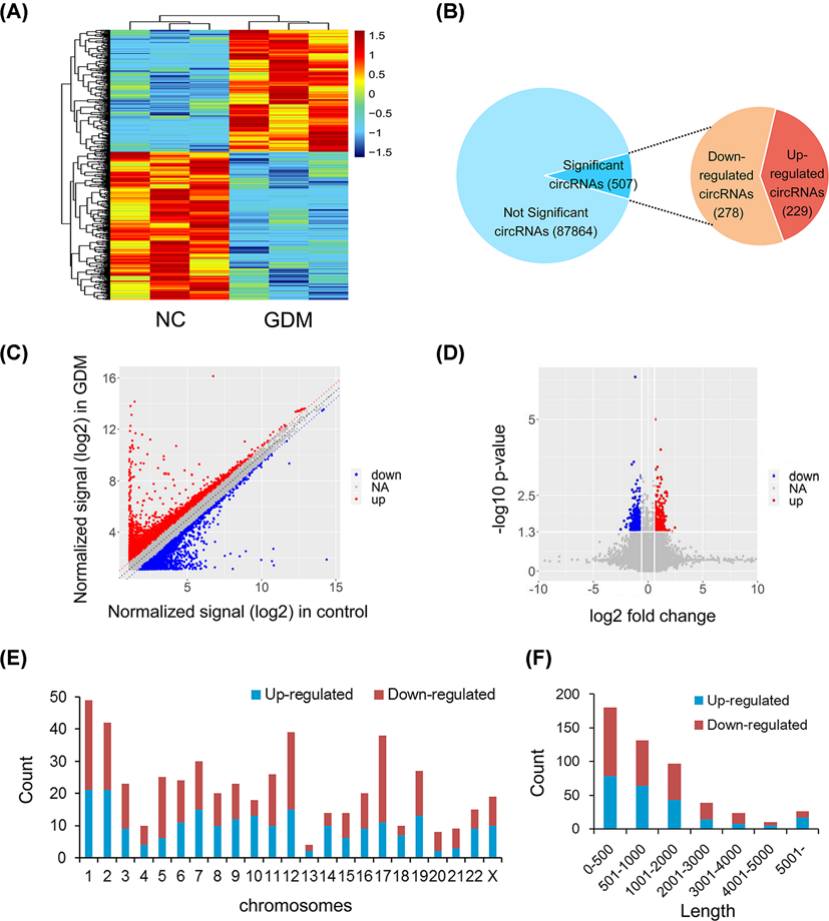
CircRNA expression profiling in umbilical cord blood exosomes from GDM patients compared with controls (**A**) Clustered heat map analysis of differentially expressed circRNAs. (**B**) The total circRNAs detected by microarray and differentially expressed circRNAs between two groups. (**C**) Scatter plots of circRNAs signal values. (**D**) Volcano plots visualizing the differentially expressed circRNAs. (**E**) Chromosome distributions of the differential circRNAs. (**F**) Length distributions of the differential circRNAs.

Hierarchical cluster analysis revealed the circRNA expression levels in umbilical cord blood exosomes from the GDM patients and controls (Figure 3A), and showed that these levels were distinguishable between the two groups. Totally, 507 circRNAs were screened as differentially expressed circRNAs by the filter criteria of fold change (FC) ≥ 1.5, *P* value < 0.05. Of these, 229 circRNAs were up-regulated, and 278 circRNAs were down-regulated in the GDM patients (Figure 3B). A scatter plot is used to display the differentially expressed circRNAs (Figure 3C), and the significantly differentially expressed circRNAs are shown in the volcano plot (Figure 3D). The top 20 up- and down-regulated circRNAs are listed in Table 1. These data suggested that circRNA expression in GDM patients was different from that in the normal controls. Additionally, Figure 3E depicts the chromosome distributions of these differential circRNAs and Figure 3F shows that the lengths of the differential circRNAs were mostly shorter than 2000 bp.

**Table 1:** The top 20 up-regulated and down-regulated circRNAs between GDM patients and controls

circRNA_ID	Chr	Length	Host gene	Normalized signal (log2)						Fold change	*P*	Regulation
				GDM- sample1	GDM- sample2	GDM- sample3	NC- sample1	NC- sample2	NC- sample3			
circ_0033104	14	2635	ATG2B	3.03	4.33	2.94	1.14	1.15	1.13	5.44	0.037	Up
circ_0004561	8	8151	C8orf83	2.71	4.17	4.70	1.32	2.62	1.22	4.62	0.050	Up
circ_0058247	2	5784	USP37	2.71	3.86	2.64	1.31	1.23	1.24	3.79	0.044	Up
circ_0005616	12	883	WNK1	3.96	2.64	2.69	1.84	1.26	1.25	3.39	0.045	Up
circ_0034091	15	148	NIPA2	3.25	2.99	3.37	1.17	1.42	1.79	3.32	0.003	Up
circ_0022742	11	820	ARL2	3.35	3.19	2.72	1.18	1.13	1.75	3.31	0.003	Up
circ_0011612	1	14126	EIF2C1	2.82	3.04	2.71	1.25	1.19	1.19	3.14	0.002	Up
circ_0063759	22	143	FBLN1	2.66	2.62	3.34	1.14	1.31	1.34	3.14	0.015	Up
circ_0020833	11	1485	OSBPL5	2.53	2.09	3.26	1.09	1.08	1.10	3.08	0.045	Up
circ_0010898	1	785	GALE	3.04	2.23	3.36	1.46	1.30	1.30	3.02	0.043	Up
circ_0024650	11	960	ARHGEF12	2.54	2.58	2.89	1.09	1.07	1.10	3.01	0.005	Up
circ_0012764	1	742	TM2D1	2.46	2.72	3.49	1.16	1.20	1.71	2.99	0.021	Up
circ_0046060	17	323	RPTOR	2.93	3.12	2.93	1.38	1.70	1.24	2.91	0.003	Up
circ_0074673	5	748	G3BP1	2.03	2.70	3.05	1.20	1.11	1.13	2.85	0.039	Up
circ_0050910	19	1686	ACTN4	2.53	2.98	2.01	1.08	1.09	1.09	2.79	0.037	Up
circ_0019007	10	579	WAPAL	2.55	2.74	2.36	1.08	1.09	1.09	2.77	0.006	Up
circ_0053887	2	369	LTBP1	3.32	3.28	3.43	1.19	1.78	2.39	2.77	0.044	Up
circ_0027041	12	331	RBMS2	2.78	3.22	3.39	1.10	2.29	1.38	2.77	0.032	Up
circ_0050908	19	82	ACTN4	2.53	2.42	3.56	1.17	1.21	1.91	2.75	0.040	Up
circ_0033110	14	2717	ATG2B	1.18	1.18	1.18	4.18	3.84	2.56	5.65	0.041	Down
circ_0005132	8	503	ARHGEF10	1.12	1.12	2.22	3.09	3.29	4.51	4.56	0.022	Down
circ_0026089	12	3744	MLL2	1.10	1.12	1.14	3.78	2.95	2.50	4.16	0.034	Down
circ_0087613	9	911	SLC35D2	1.25	1.64	1.81	4.21	3.41	2.72	3.96	0.035	Down
circ_0014841	1	2154	SPTA1	1.86	1.15	1.14	4.06	3.00	2.66	3.85	0.028	Down
circ_0007193	21	424	DOPEY2	1.55	1.15	1.17	3.14	3.61	2.52	3.62	0.018	Down
circ_0025360	12	422	FOXJ2	1.24	2.42	1.72	4.03	3.24	3.39	3.29	0.017	Down
circ_0050714	19	187	WDR62	1.12	1.19	1.61	3.44	2.77	2.61	3.17	0.009	Down
circ_0000420	12	3543	ZFC3H1	1.46	2.02	1.24	3.63	3.50	2.31	3.13	0.043	Down
circ_0080054	7	227	TMED4	1.39	1.10	2.27	3.75	3.43	2.52	3.12	0.032	Down
circ_0041805	17	1176	GPS2	1.22	1.82	1.85	3.87	3.21	2.48	3.11	0.042	Down
circ_0042352	17	617	FBXW10	1.73	1.21	1.21	3.62	2.80	2.41	3.10	0.032	Down
circ_0089739	9	591	EHMT1	1.22	1.23	1.31	3.11	3.09	2.29	3.08	0.027	Down
circ_0086833	9	657	C9orf100	2.45	2.28	1.23	3.98	3.74	3.23	3.04	0.029	Down
circ_0062292	22	83	DGCR8	1.29	1.27	1.29	3.20	2.73	2.63	3.02	0.012	Down
circ_0024576	11	650	MCAM	1.34	1.31	1.33	2.60	3.10	3.01	3.01	0.009	Down
circ_0061688	21	144	DSCR3	1.16	1.17	2.28	3.17	3.65	2.67	2.99	0.028	Down
circ_0028543	12	1673	RBM19	1.13	1.14	1.15	2.26	2.88	2.88	2.95	0.017	Down
circ_0044645	17	219	SPAG9	1.20	1.22	1.25	3.18	2.72	2.29	2.94	0.028	Down
circ_0083597	8	371	HR	2.07	1.15	1.17	3.49	3.23	2.27	2.93	0.035	Down

### Validation of differentially expressed circRNA by qPCR

**Figure 4:**
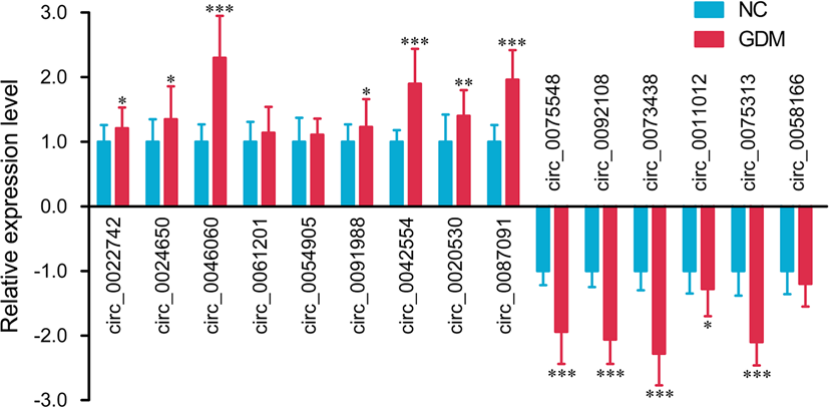
Validation of differentially expressed circRNA by qPCR *P<0.05, **P<0.01, and ***P<0.001.

Based on relatively high abundance, FC ≥ 2, *P*<0.01, and their host genes, we selected 15 candidate circRNAs to validate their expression in umbilical cord blood exosomes from additional 20 GDM patients and 20 controls, including 9 up-regulated circRNAs (circ_0022742, circ_0024650, circ_0046060, circ_0061201, circ_0054905, circ_0091988, circ_0042554, circ_0020530 and circ_0087091) and 6 down-regulated circRNAs (circ_0075548, circ_0092108, circ_0073438, circ_0011012, circ_0075313 and circ_0058166). In parallel with the microarray data, qPCR results showed that the expression of circ_0046060, circ_0042554 and circ_0087091 were increased, and the expression of circ_0075548, circ_0092108, circ_0073438 and circ_0075313 were decreased in GDM patients (Figure 4).

### GO and KEGG pathway analysis of the circRNA parental genes

**Figure 5:**
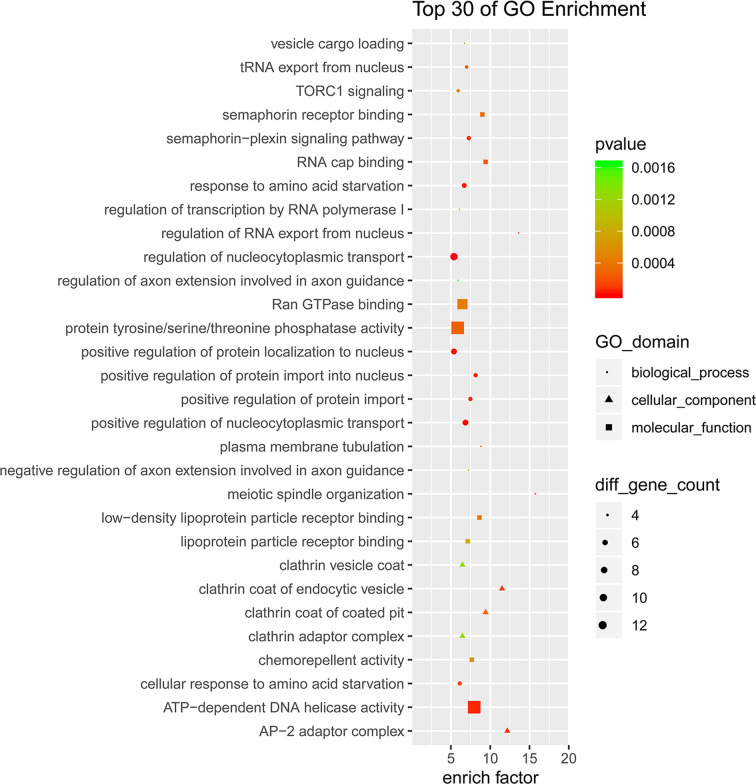
GO analysis of the differential circRNA parental genes

To examine the biological functions of these differential circRNAs, GO analysis and KEGG pathways analysis were performed. In the GO analysis, the number of parental genes corresponding to GO entries was determined, and the enrichment score was regarded as the -log (*P*-value): For biological process, the term with the most genes was cellular process (GO:0009987, count = 394), and the most significantly enriched term was organelle organization (GO: 0006996, *P* = 5.45E-10); for cellular component, the term with the most genes was cell (GO:0005623, count = 413), and the most significantly enriched term was cytosol (GO:0005829, *P* = 2.15E-14); and for molecular function, the term with the most genes was binding (GO:0005488, count = 380), and the most significantly enriched term was ATP binding (GO:0005524, *P* = 3.72E-10) (Figure 5). All mRNAs annotated involved in these GO terms were listed in Supplementary Table S3. Here, the majority of mRNAs appear to be associated with metabolic process, growth and development.

**Figure 6:**
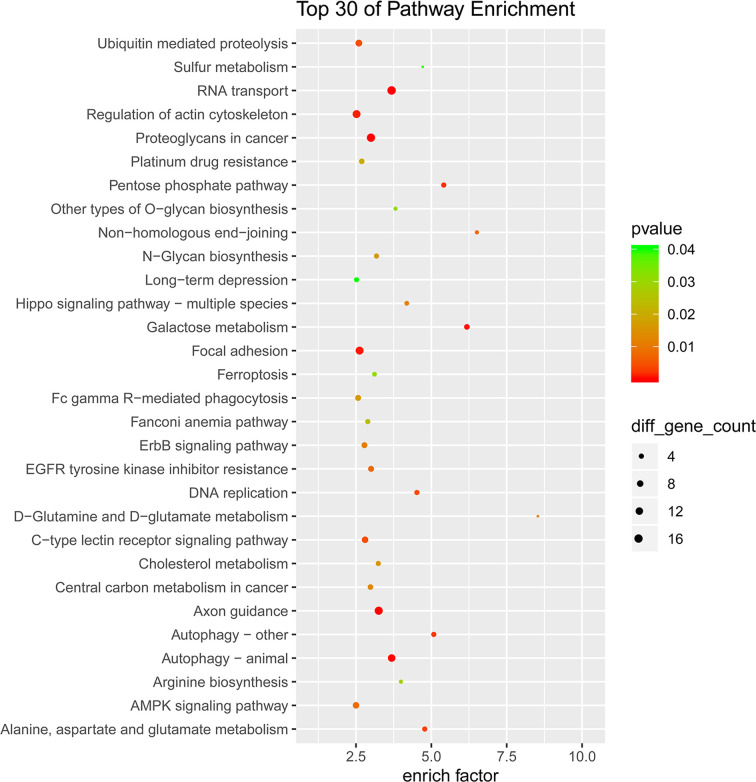
KEGG pathway analysis of the differential circRNA parental genes

Similarly, of the top 30 enrichment pathways in the KEGG pathway analysis, galactose metabolism (hsa00052), pentose phosphate pathway (hsa00030), glycan biosynthesis (hsa00514 and hsa00510), cholesterol metabolism (hsa04979), DNA replication (hsa03030), and RNA transport (hsa03013), which are related to glycometabolism, lipometabolism, growth and development, are most likely involved in GDM occurrence and fetal growth (Figure 6). All mRNAs annotated involved in these pathways were listed in Supplemntary Table S4.

### Prediction of circRNA/microRNA interactions

Recent studies have showed that RNAs regulate each other with miRNA response elements (MREs) and this mechanism is known as ‘competing endogenous RNA (ceRNA)’ hypothesis [19]. CircRNAs can regulate miRNA-targeted gene expression, transcription and protein synthesis as ceRNA molecules or efficient miRNA sponges [19]. Interactions between the differential circRNAs and miRNAs were theoretically predicted by miRanda based on the MREs. We found that 2417 miRNAs could be paired with 504 differentially expressed circRNAs, with the criteria of a max score ≥ 140 and a max energy ≤ -20 (Supplemntary Table S5); the lower the max energy is, the more significant the correlation. These results suggest that circRNAs may participate in GDM pathogenesis via interactions with GDM incidence-related miRNAs.

## Discussion

Emerging evidences show that exosomal circRNAs could be released from various cells and carry signaling molecules for cellular communication and even organ crosstalk [15]. Although exosomal circRNAs have received increasing attention in recent years, the characteristics and functions of exosomal circRNAs remain poorly understood. Our study is the first to construct circRNA differential expression profiling in umbilical cord blood exosomes of GDM patients as a starting point to explore the relationship between exosomal circRNAs and GDM development. We found the exosome particle size was larger, and the exosome concentration was higher in the GDM patients. And more than 80,000 circRNAs in umbilical cord blood exosomes were evaluated, including 507 differentially expressed circRNAs in GDM patients compared with controls, which suggested that the exosomal circRNA expression patterns in the GDM samples were different from those in controls.

Based on GO and KEGG biological pathway analyses, several significantly enriched pathways (such as pentose phosphate pathway, cholesterol metabolism, galactose metabolism, cellular process, DNA replication, RNA transport, etc.) were found to be closely related to metabolic process, growth, and development, which are important in GDM development and fetus growth. The pentose phosphate pathway (PPP) is primarily catabolic and serves as an alternative glucose oxidizing pathway for the generation of NADPH that is required for reductive biosynthetic reactions such as those of cholesterol biosynthesis, bile acid synthesis, steroid hormone biosynthesis, and fatty acid synthesis [20]. Previous study also reported that the expression levels of genes coding for enzymes within the PPP were significantly down-regulated in white blood cells and placental tissue taken from GDM patients [21]. Circulating lipid patterns in GDM versus normal pregnancy have been extensively studied, with most studies observing higher triglyceride levels across all trimesters of pregnancy in women with GDM [22,23]. Impaired glucose metabolism during pregnancy may also associated with changes in fetal cholesterol metabolism [24]. Galactose metabolism was also impaired in GDM patients, with higher d-galactose among women who later developed GDM [25]. While cellular process, DNA replication and RNA transport are central to fetus growth and development.

Recent studies have reported that exosomal circRNAs regulate gene expression through a miRNA sponge mechanism [19]. circRNAs have many miRNA-binding sites that competitively bind to miRNAs. Thus, circRNAs may alleviate the inhibitory effects of miRNAs on target molecules, thereby regulating gene expression. For instance, tumor-released exosomal circ-PDE8A promotes invasive growth via the miR-338/MACC1/MET pathway in pancreatic cancer [26]; exosomal circRNA derived from gastric tumor promotes white adipose browning by targeting the miR-133/PRDM16 pathway [27]; and exosomal circ100284 regulates the cell cycle by acting as a sponge of miR-217 [28]. In our study, we found that most of the exosomal circRNAs harbored miRNA-binding sites, and some miRNAs were associated with GDM. For example, miR-330, miR-23a, and miR-16-5p were reported to be up-regulated in the plasma of GDM patients [29–32]. miR-330, miR-23a and miR-16-5p were matched with circ_0092108, which was verified to down-regulated in umbilical cord blood exosomes of GDM patients. Thus, we speculate that the roles of circRNAs in GDM may be related to miRNA-mediated effects. However, the underlying mechanism of the circRNA–miRNA–target gene interaction needs further investigation.

In summary, the microarray results revealed, for the first time, a significant difference in exosomal circRNA expression between GDM patients and the control group. Bioinformatics analysis further predicted the potential effects of these differentially expressed circRNAs and their interactions with miRNAs. However, the exploration of exosomal circRNAs in GDM is just beginning, and more functional analyses and investigations are needed in the future to gain a more comprehensive understanding of this topic. Collectively, these results lay a foundation for extensive studies on the role of exosomal circRNAs in GDM development and fetus growth.

## Supplementary Material

Supplementary Tables S1-S3

Supplementary Table

## Data Availability

All datasets generated for this study are included in the manuscript and/or the supplementary files. The original data was available online (https://doi.org/10.6084/m9.figshare.13140626.v1).

## Abbreviations

ceRNA: competing endogenous RNA
circRNA: circular RNA
GDM: gestational diabetes mellitus
GO: Gene Ontology
KEGG: Kyoto Encyclopedia of Genes and Genomes
MRE: miRNA response element
T2D: Type 2 diabetes
