# Supplementary material for: Circular RNA expression profiles in umbilical cord blood exosomes from normal and gestational diabetes mellitus patients
Source: Biosci Rep. 2020 Nov 17;40(11):BSR20201946. doi: 10.1042/BSR20201946 (PMC7670577; doi:10.1042/BSR20201946)
Supplement: Supplementary Tables S1-S3 [file BSR-2020-1946_supp.pdf]

TABLE S1 Sequences of the divergent primers

Gene	Forward (5'-3')	Reverse (5'-3')	Product Size (bp)
**GAPDH**	TGCCACTCAGAAGACTGTGG	TTCAGCTCTGGGATGACCTT	128
**circ_0022742**	GGCCTTTGGCTACCATACCA	TTGTCCAGGCCAATCGGGG	132
**circ_0024650**	GGGGGTCTTGATGACAGTGG	AGCTTTGTTATCTGAGATGCGT	135
**circ_0046060**	CCCTGCGCTGAACTACACG	ACGATCAAGCCAGCATTGGA	111
**circ_0061201**	CGGCACTATGAGGACTTCCC	AGTGGCGCCCCTGGTTATAG	106
**circ_0054905**	GGCAAACTGCTCAAGGGATTC	TCACTCAGTTCCACAGGTCG	112
**circ_0091988**	CCAAGCCTAGCAAGACAGCA	TAGTGCAGGATCAGGGTCCA	84
**circ_0042554**	AACATTCTAGGAGCTGTCACCA	CTACAGACTCATCCGCAGCC	133
**circ_0020530**	GACACCGGCTGACGACTTC	TCCGATTTGTAGATCATGGTGGT	75
**circ_0087091**	GAGGATCATGACAGGTGACCA	GCTCTTGAGATCATTCTTGGCA	71
**circ_0075548**	CTGGCCACCATGGAGAATAC	AGATGCAGCTGGCTGTGATT	132
**circ_0092108**	GTCCCACCAACTCTGCACTC	CAGCCACGGCTGTTTGC	138
**circ_0073438**	TGAACAACCAGAAGAGTTACCTTG	AGTCATCAAGGCTGTGGAGT	137
**circ_0011012**	CCAACAATTCTGCAGCCATCC	CGAGTATGGGTTAGTCCCGC	84
**circ_0075313**	CACCCTGGAGAAAGCCTACC	GTTTGGTCCTCCACCGCTC	119
**circ_0058166**	AGGTGAAGATGGGTTGGATGAA	ACTTTTGCTGAATCGGCTGC	103

GAPDH, glyceraldehyde phosphate dehydrogenase.

TABLE S2 Clinical data for the GDM patients and normal controls

Variables	GDM (n = 20)	Controls (n = 20)	*P*
Maternal age (years)	29.40 ± 3.59	28.35 ± 4.53	0.422
Gestational age (weeks)	39.10 ± 1.29	39.15 ± 1.14	0.897
Birth weight (g)	3433.00 ± 477.59	3060.00 ± 502.51	0.021
Neonatal body fat (g)	892.15 ± 149.71	786.13 ± 141.81	0.027
Fasting plasma glucose/mmol/I	5.46 ± 0.23	4.47 ± 0.27	<0.001
1 h plasma glucose/mmol/L	10.70 ± 0.40	9.36 ± 0.31	<0.001
2 h plasma glucose/mmol/L	8.93 ± 0.61	7.50 ± 0.38	<0.001

TABLE S3 GO enrichment analysis of the circRNA parental genes

GO ID	GO term	Type	GeneRatio	BgRatio	Gene list	*P* value	Q value	Enrich_factor
GO:0035455	response to interferon-alpha	biological_process	5/398	20/22360	IFNAR1 EIF2AK2 ADAR PDE12 GAS6	0.000009748	0.05039	14.05
GO:0031904	endosome lumen	cellular_component	6/398	37/22360	LGMN LRPAP1 AP4E1 CTSS AP4M1 APP	0.00002173	0.05616	9.11
GO:0002407	dendritic cell chemotaxis	biological_process	5/398	26/22360	ANO6 GAS6 CCR6 CCL5 CXCR4	0.00003507	0.06043	10.8
GO:0036336	dendritic cell migration	biological_process	5/398	29/22360	GAS6 CCR6 CCL5 CXCR4 ANO6	0.00006005	0.0776	9.69
GO:0046827	positive regulation of protein export from nucleus	biological_process	4/398	22/22360	GAS6 CAMK1 GSK3B TP53	0.0001915	0.1414	10.21
GO:0030554	adenyl nucleotide binding	molecular_function	52/398	1711/22360	EIF2AK2 ATP2B1 P2RX4 AKT2 SLFN13 NUBPL MAP3K2 PEAK1 YES1 OAS3 ADCY2 GK5 CNBD2 TIE1 MYO5A ABCB5 AAK1 MYO1C MAPKAPK5 NMNAT1 NLRP3 LIG1 MSH6 ATP6V1A RFC2 ABCC9 SCYL3 TP53 SHPK ACSL6 TTLL3 GCLC CHEK2 ABCC3 GSK3B TAOK1 TTLL7 ABCC10 IRAK4 TNK2 GRK5 MAPK12 MKNK1 CAMK1 MAP3K9 DDX54 DGKZ PRKAR2A NAGK TBCK DDX51 DHX30	0.0003134	0.1473	1.71
GO:0004532	exoribonuclease activity	molecular_function	5/398	41/22360	ISG20L2 DIS3L EXOSC6 PNPT1 PDE12	0.0003321	0.1431	6.85
GO:0010256	endomembrane system organization	biological_process	20/398	466/22360	COG1 RAB29 CXCR4 TOR1AIP2 TMEM170A XKR4 UBXN2A F2R A4GALT SYP WHAMM MYO5A RAB22A FHDC1 AKT2 TRIM72 BCAS3 STX16 VPS28 ANO6	0.000369	0.1122	2.41
GO:0090503	RNA phosphodiester bond hydrolysis, exonucleolytic	biological_process	5/398	42/22360	EXOSC6 PDE12 PNPT1 DIS3L ISG20L2	0.0003739	0.1074	6.69
GO:0005524	ATP binding	molecular_function	50/398	1641/22360	OAS3 GK5 ADCY2 YES1 TIE1 AKT2 NUBPL SLFN13 EIF2AK2 ATP2B1 P2RX4 PEAK1 MAP3K2 RFC2 ABCC9 ATP6V1A MSH6 LIG1 NMNAT1 NLRP3 SHPK TP53 SCYL3 ABCB5 MYO5A MAPKAPK5 MYO1C AAK1 ABCC10 TNK2 IRAK4 ABCC3 CHEK2 GCLC ACSL6 TTLL3 TAOK1 TTLL7 GSK3B NAGK DGKZ DHX30 DDX51 TBCK CAMK1 MAPK12 MKNK1 GRK5 DDX54 MAP3K9	0.0003809	0.1036	1.71
GO:0019079	viral genome replication	biological_process	9/398	135/22360	ADAR CCL5 RAB29 EIF2AK2 C19ORF66 APOBEC3D GAS6 PDE12 OAS3	0.0005182	0.1276	3.75

GO:0005737	cytoplasm	cellular_component	281/398	12820/22360	PPM1K TRIM72 ZDHHC15 BCAS3 ICOSLG GK5 NPAT FBXL4 CNBD2 UBXN2A ZNF106 EIF2AK2 ASPA VPS52 CARD19 RAB40B ELMOD1 SRSF10 COQ6 SNX27 RPL7L1 MTFMT DDX54 SPATS2 DESI1 GAS6 CNKSR3 MGLL IRAK4 H6PD TMEM170A USP28 TTLL3 SLC25A44 SRGAP2B CD3G IKZF3 ESYT3 MARVELD2 GALNT16 PIGL KDSR WNT4 GSDMB PDE4C STAC2 PLEKHA2 TAF8 DPP9 IFNAR1 C19ORF66 RSPH3 CC2D2A DDI2 KRT18 RGS17 PNPT1 VPS33A DIS3L TBCK RAB3B A4GALT RABGAP1 SLC30A6 TMEM106B CAMK1 FTO TTYH1 MLX STX16 ANO6 GJC1 TRAF3IP1 C1ORF210 F2R FARP1 METTL21A ERMAP SYAP1 SCAI PIK3R3 CYP4V2 MYO1C DRG1 SLC35E2B PSMD9 FAM161A TMTC1 FGD5 BAALC PGM2L1 SLFN13 STYX PSTPIP2 EYA3 PEAK1 VPS28 NAGK DNAL1 ARFRP1 RPRD1B PLEKHB2 RNF115 DENND2A RCAN1 CORO7 NXN PDP2 ABCC10 DYNC2LI1 FDPS CCDC125 B3GALT1 QPRT TRIM25 SEC62 GNS AAK1 ADCY2 SLC37A2 WHAMM LTBR ZNF274 NOA1 TBCCD1 CRYZL1 IBA57 STEAP2 DCTD DUOX1 PRR11 HMCN2 CS USP44 TMEM187 SEZ6 ARNT2 GSDMA TTLL7 RFTN1 NLRP3 SYT12 MXD1 SHPK XIAP SPTAN1 CYB5R4 SLC33A1 EXOSC6 ZC3H14 SLC25A26 TSEN2 MLXIPL PYGB P2RX4 FHDC1 MED22 NUBPL AKT2 GALNT15 DGKZ FITM2 SNX21 SSTR2 MAPK12 GNPTG CXCR4 EPN1 CLCC1 SUMF2 CYP51A1 CEP112 KIAA1191 SLC9A7 C9ORF78 COL8A1 RAB29 PKNOX1 MSH6 POU5F1 SPC25 SYP SMARCC1 AS3MT HIF1AN ITGA3 AP4E1 SLC39A13 RILPL1 OAS3 GMEB1 CX3CL1 KLHL1 GABRB3 PRKAR2A ADAR LGMN CYB561A3 MMS22L GRK5 MR1 SYNRG SRCIN1 LRRC8B TNK2 TTC3 TENM2 HBE1 ABCC3 HP ACOX1 LIG1 ATP6V1A ABCC9 PDE12 TP53 TONSL CALCRL GPAT4 APOL1 MAPKAPK5 TMOD3 LRTOMT TTI2 AP4M1 OGFOD1 MTAP TMEM266 MAP3K2 ACOT8 PIGR NDUFA10 APP KDELC2 RNF7 HERC4 SOX11 EMC10 ACSL6 GCLC RGS12 GSK3B NCOR1 NMNAT1 WBP2 CTSS FBXL18 MCM3AP SCYL3 COX6B2 ICA1L SMCR8 APOBEC3D MYO5A LRPAP1 YES1 PCSK7 SLC35E1 BBS1 ZFPM2 RAB22A OTUD4 TRMT10B ODF2L COG1 ADD2 TOR1AIP2 DHX30 SULT1B1 NDUFAF7 CASP8 COX5B SMAD2 MKNK1 LETMD1 SAMD4B SLC35F6 CHEK2 ATXN3 TAOK1 PTPRM	0.0007221	0.1493	1.23
GO:0006892	post-Golgi vesicle-mediated transport	biological_process	8/398	117/22360	VPS52 BBS1 STEAP2 CORO7 KRT18 MYO5A ARFRP1 AP4M1	0.0008028	0.1596	3.84
GO:0032555	purine ribonucleotide binding	molecular_function	59/398	2093/22360	CAMK1 MAPK12 MKNK1 GRK5 DDX54 MAP3K9 ARFRP1 NOA1 NAGK DGKZ PRKAR2A RAB3B DHX30 DDX51 TBCK ABCC3 CHEK2 GCLC ACSL6 TTLL3 TAOK1 TTLL7 GSK3B ABCC10 TNK2 IRAK4 ABCB5 MYO5A MAPKAPK5 DRG1 MYO1C AAK1 RFC2 ABCC9 ATP6V1A MSH6 RAB29 LIG1 NMNAT1 NLRP3 SHPK TP53 SCYL3 AKT2 NUBPL SLFN13 RAB22A EIF2AK2 ATP2B1 P2RX4 PEAK1 MAP3K2 RAB40B OAS3 GK5 ADCY2 YES1 TIE1 CNBD2	0.0008425	0.1405	1.58
GO:0035639	purine ribonucleoside triphosphate binding	molecular_function	57/398	2012/22360	SHPK SCYL3 TP53 MSH6 ATP6V1A ABCC9 RFC2 NLRP3 NMNAT1 LIG1 RAB29 MYO1C DRG1 MAPKAPK5 AAK1 ABCB5 MYO5A TIE1 OAS3 ADCY2 GK5 YES1 PEAK1 RAB40B MAP3K2 RAB22A AKT2 SLFN13 NUBPL ATP2B1 P2RX4 EIF2AK2 RAB3B TBCK DDX51 DHX30 ARFRP1 DGKZ NAGK NOA1 MAP3K9 DDX54 MAPK12 MKNK1 CAMK1 GRK5 TNK2 IRAK4 ABCC10 GSK3B TTLL7 TAOK1 ABCC3 TTLL3 ACSL6 GCLC CHEK2	0.000911	0.1472	1.59
GO:0017076	purine nucleotide binding	molecular_function	59/398	2106/22360	ARFRP1 PRKAR2A DGKZ NOA1 NAGK RAB3B DDX51 TBCK DHX30 MAPK12 MKNK1 CAMK1 GRK5 MAP3K9 DDX54 ABCC10 TNK2 IRAK4 ABCC3 ACSL6 TTLL3 CHEK2 GCLC GSK3B TAOK1 TTLL7 ATP6V1A MSH6 RFC2 ABCC9 NMNAT1 NLRP3 RAB29 LIG1 SHPK SCYL3 TP53 ABCB5 MYO5A DRG1 MYO1C MAPKAPK5 AAK1 OAS3 GK5 ADCY2 YES1 CNBD2 TIE1 RAB22A AKT2 NUBPL SLFN13 EIF2AK2 ATP2B1 P2RX4 PEAK1 MAP3K2	0.0009746	0.1527	1.57

					RAB40B			
GO:0000175	3'-5'-exoribonuclease activity	molecular_function	4/398	33/22360	PNPT1 PDE12 ISG20L2 DIS3L	0.001015	0.1543	6.81
GO:0043229	intracellular organelle	cellular_component	313/398	14539/22360	AAK1 GNS WHAMM ZNF274 LTBR SLC37A2 ZNF445 ISG20L2 INTS6 DUOX1 STEAP2 IBA57 PRR11 NOA1 TBCCD1 SEZ6 ARNT2 ZNF430 TMEM187 RFTN1 N4BP2L2 TTLL7 SPN ZNF789 MDM4 USP44 ZNF573 ZNF345 CS CYP4V2 SCAI SYAP1 SLC35E2B DRG1 MED17 MYO1C METTL21A PSTPIP2 STYX UBE3C VPS28 PEAK1 EYA3 ZNF7 C1ORF61 BAALC SBNO2 TMTC1 FAM161A FGD5 PSMD9 DENND2A PDP2 CIART NXN CORO7 ZNF441 RCAN1 RPRD1B ARFRP1 DNAL1 PLEKHB2 SEC62 DYNC2LI1 FDPS ABCC10 TRIM25 B3GALT1 ZNF286B BRDT MARVELD2 RARG GALNT16 IKZF3 CD3G ESYT3 C19ORF66 IFNAR1 DPP9 RSPH3 TAF8 PNPT1 RGS17 KRT18 ZMYM5 DDI2 CC2D2A PIGL C1ORF174 PLEKHA2 ZNF80 KDSR WNT4 FTO TTYH1 SLC30A6 TMEM106B CAMK1 RABGAP1 A4GALT ANO6 GJC1 STX16 MLX DIS3L SNORA13 RRP12 ZHX3 VPS33A RAB3B DDX51 TBCK FARP1 TRAF3IP1 C1ORF210 F2R CRX TRIM72 ZNF814 ICOSLG BCAS3 ZDHHC15 PPM1K RIPPLY3 ASPA HNRNPUL1 EIF2AK2 ATP2B1 TCF4 SRSF10 CARD19 ELMOD1 RAB40B VPS52 GK5 ZNF106 UBXN2A FBXL4 NPAT ZKSCAN1 DDX54 COQ6 MTFMT RPL7L1 SNX27 TTLL3 SLC25A44 R3HCC1 DESI1 GAS6 USP28 TMEM170A H6PD MGLL IRAK4 RRP1 APOBEC3D MYO5A KLHL8 FBXL18 WBP2 CTSS NMNAT1 SMCR8 ICA1L COX6B2 SCYL3 MCM3AP OTUD4 ZNF669 RAB22A ODF2L SLC35E1 ZNF70 PCSK7 YES1 LRPAP1 ZFPM2 BBS1 SAMD4B MKNK1 LETMD1 SMAD2 COX5B NSRP1 COG1 CASP8 NDUFAF7 SUMO2 ZNF677 TOR1AIP2 DHX30 ADD2 KMT2D CHEK2 TAOK1 ATXN3 SLC35F6 TONSL C2ORF49 TMOD3 MAPKAPK5 APOL1 GPAT4 CALCRL ABCC9 PDE12 ATP6V1A LIG1 ZBTB37 TP53 MTAP ZNF264 ZNF570 MAP3K2 AP4M1 TTI2 NUDT22 LRTOMT OGFOD1 APP NDUFA10 ZNF772 ACOT8 PIGR RGS12 ACSL6 NCOR1 GSK3B SOX11 HERC4 ZNF483 RNF7 KDELC2 EMC10 FOXD2 SMARCC1 SYP SPC25 SLC39A13 AP4E1 HIF1AN POU5F1 PKNOX1 MSH6 RAB29 COL8A1 GABRB3 KLHL1 RILPL1 OAS3 SON GMEB1 SYNRG MR1 MMS22L GRK5 ADAR PRKAR2A LGMN CYB561A3 ZNF766 ABCC3 ZNF583 ACOX1 HP MNT SRCIN1 TTC3 TENM2 TNK2 LRRC8B NAP1L6 CCDC36 XIAP EXOSC6 CYB5R4 SLC33A1 SPTAN1 CWC25 RFC2 MXD1 SYT12 NLRP3 ZNF8 NUBPL AKT2 MED22 FHDC1 P2RX4 GALNT15 ZC3H14 PYGB MLXIPL SLC25A26 TSEN2 MAPK12 SNX21 ZNF800 GNPTG DGKZ FITM2 CEP112 CYP51A1 C9ORF78 SLC9A7 C9ORF84 SUMF2 FCF1 CLCC1 PCGF5 EPN1 CXCR4	0.00108	0.1509	1.21
GO:0046872	metal ion binding	molecular_function	111/398	4533/22360	ZNF583 HBE1 TNK2 TENM2 TTC3 ANKMY1 TTYH1 FTO ZNF766 CYB561A3 ADAR ZHX3 ZMYM5 TRABD2B STAC2 ZNF80 PDE4C GMEB1 OAS3 GALNT16 HIF1AN ITGA3 ZNF286B RARG ESYT3 IKZF3 MSH6 ZNF839 CYP51A1 SUMF2 PCGF5 IRAK4 GAS6 ZNF800 PCDH9 ZKSCAN1 MAPK12 NT5DC3 DGKZ GALNT15 AKT2 ASPA NUBPL ATP2B1 P2RX4 ZNF106 ZC3H14 CYB5R4 SPTAN1 ZDHHC15 TRIM72 XIAP ZNF814 ZNF8 SYT12 RNF166 PPM1K ZNF789 ZNF430 KMT2D CHEK2 HMCN2 ZNF345 ZNF573 USP44 MDM4 MKNK1 SMAD2 DCTD DUOX1 STEAP2 COX5B ZNF677 DZANK1 ZNF274 ZNF669 ZFPM2 ZNF70 ADCY2 ZNF445 PRR3 MYO5A APOBEC3D GNS GCLC TRIM25 ZNF483 FDPS RNF7 PDP2 ZNF441 ZNF772 APP RNF115 ZNF570 MAP3K2 EYA3 ZNF264 SLFN13 PGM2L1 OGFOD1 FGD5 PCDHB9 NUDT22 ZNF7 DRG1 CYP4V2 TP53 PDE12 ZBTB37 LIG1	0.001259	0.1549	1.38
GO:0016896	exoribonuclease activity, producing 5'-phosphomonoesters	molecular_function	4/398	35/22360	DIS3L ISG20L2 PNPT1 PDE12	0.001293	0.1519	6.42

GO:0000166	nucleotide binding	molecular_function	64/398	2359/22360	RAB22A OAS3 ADCY2 YES1 MYO5A AAK1 MSH6 NMNAT1 RAB29 SCYL3 ABCC3 CHEK2 ACOX1 TAOK1 TTLL7 TNK2 MKNK1 CAMK1 DUOX1 GRK5 PRKAR2A NOA1 RAB3B CRYZL1 TBCK DDX51 DHX30 AKT2 NUBPL SLFN13 EIF2AK2 ATP2B1 P2RX4 PEAK1 MAP3K2 RAB40B GK5 CNBD2 TIE1 ABCB5 DRG1 MYO1C MAPKAPK5 ATP6V1A RFC2 ABCC9 NLRP3 LIG1 SHPK TP53 ACSL6 TTLL3 GCLC GSK3B ABCC10 H6PD IRAK4 MAPK12 MAP3K9 DDX54 ARFRP1 DGKZ COQ6 NAGK	0.001345	0.1545	1.52
GO:0002687	positive regulation of leukocyte migration	biological_process	9/398	155/22360	ANO6 GAS6 LGMN SPN P2RX4 APP CCL5 PGF CCR6	0.001434	0.1611	3.26
GO:0070050	neuron cellular homeostasis	biological_process	4/398	36/22360	ATP2B1 TAOK1 CACNB2 CX3CL1	0.001451	0.1596	6.24
GO:1901653	cellular response to peptide	biological_process	17/398	414/22360	MYO1C GSK3B RABGAP1 TRIM72 AKT2 SMARCC1 SYAP1 MYO5A APP GCLC PIK3R3 ZNF106 LGMN TP53 ADCY2 ATP6V1A PRKAR2A	0.001514	0.163	2.31
GO:0043231	intracellular membrane-bounded organelle	cellular_component	272/398	12553/22360	ATXN3 TAOK1 CHEK2 KMT2D SLC35F6 NSRP1 COX5B MKNK1 LETMD1 SMAD2 SAMD4B ZNF677 SUMO2 TOR1AIP2 DHX30 NDUFAF7 CASP8 COG1 OTUD4 ZNF669 ZFPM2 YES1 LRPAP1 ZNF70 PCSK7 SLC35E1 APOBEC3D MYO5A RRP1 SCYL3 MCM3AP COX6B2 SMCR8 NMNAT1 FBXL18 WBP2 CTSS KLHL8 GSK3B NCOR1 ACSL6 RGS12 EMC10 RNF7 KDELC2 HERC4 ZNF483 SOX11 ZNF772 NDUFA10 APP PIGR ACOT8 MAP3K2 ZNF570 ZNF264 MTAP OGFOD1 NUDT22 LRTOMT TTI2 AP4M1 CALCRL GPAT4 MAPKAPK5 APOL1 C2ORF49 TONSL TP53 LIG1 ZBTB37 ATP6V1A PDE12 ACOX1 ZNF583 ABCC3 NAP1L6 LRRC8B TNK2 TTC3 TENM2 MNT MMS22L GRK5 MR1 SYNRG ZNF766 CYB561A3 LGMN ADAR GMEB1 SON OAS3 RILPL1 HIF1AN AP4E1 SLC39A13 SMARCC1 FOXD2 SPC25 RAB29 COL8A1 MSH6 PKNOX1 POU5F1 SLC9A7 C9ORF78 CYP51A1 PCGF5 EPN1 CXCR4 CLCC1 SUMF2 FCF1 C9ORF84 GNPTG ZNF800 MAPK12 FITM2 DGKZ GALNT15 P2RX4 MED22 FHDC1 NUBPL AKT2 MLXIPL SLC25A26 TSEN2 PYGB ZC3H14 CWC25 CYB5R4 SLC33A1 SPTAN1 EXOSC6 XIAP CCDC36 ZNF8 NLRP3 MXD1 RFC2 SPN ZNF789 N4BP2L2 SEZ6 ARNT2 ZNF430 ZNF345 CS ZNF573 USP44 MDM4 PRR11 STEAP2 IBA57 DUOX1 NOA1 LTBR ZNF274 WHAMM ISG20L2 INTS6 ZNF445 SLC37A2 AAK1 GNS SEC62 B3GALT1 TRIM25 ABCC10 FDPS ZNF441 RCAN1 CORO7 NXN PDP2 CIART ARFRP1 RPRD1B EYA3 UBE3C STYX PSMD9 TMTC1 FGD5 SBNO2 BAALC C1ORF61 ZNF7 DRG1 MED17 MYO1C SLC35E2B SYAP1 SCAI CYP4V2 METTL21A CRX F2R MLX STX16 GJC1 A4GALT FTO TTYH1 SLC30A6 TMEM106B CAMK1 DDX51 RAB3B RRP12 ZHX3 VPS33A DIS3L SNORA13 DDI2 ZMYM5 KRT18 RGS17 PNPT1 TAF8 C19ORF66 IFNAR1 DPP9 KDSR WNT4 PLEKHA2 ZNF80 C1ORF174 PIGL GALNT16 RARG ZNF286B BRDT ESYT3 IKZF3 SLC25A44 H6PD MGLL IRAK4 TMEM170A USP28 DESI1 GAS6 R3HCC1 DDX54 ZKSCAN1 SNX27 RPL7L1 MTFMT COQ6 CARD19 ELMOD1 RAB40B VPS52 SRSF10 TCF4 EIF2AK2 ATP2B1 HNRNPUL1 ASPA NPAT FBXL4 ZNF106 UBXN2A GK5 ZDHHC15 BCAS3 ZNF814 RIPPLY3 PPM1K	0.001622	0.1612	1.22
GO:0046777	protein autophosphorylation	biological_process	12/398	251/22360	MKNK1 YES1 CHEK2 EIF2AK2 GRK5 PEAK1 TNK2 MAPKAPK5 GSK3B MAP3K9 AAK1 TAOK1	0.001781	0.1737	2.69
GO:0045069	regulation of viral genome replication	biological_process	7/398	106/22360	PDE12 OAS3 CCL5 ADAR APOBEC3D EIF2AK2 C19ORF66	0.001865	0.07967	3.71
GO:0007009	plasma membrane organization	biological_process	7/398	107/22360	WHAMM CXCR4 A4GALT TRIM72 ANO6 AKT2 XKR4	0.001974	0.08364	3.68
GO:0003712	transcription coregulator activity	molecular_function	22/398	612/22360	ZNF274 NCOR1 N4BP2L2 KMT2D MED22 ZFPM2 GMEB1 NPAT WNT4 PSMD9 SOX11 MNT MED17 DDX54 TONSL BRDT SCAI RARG SMARCC1 MXD1 WBP2 ZHX3	0.002105	0.08705	2.02

GO:0008144	drug binding	molecular_function	53/398	1915/22360	TNK2 IRAK4 ABCC10 TAOK1 TTLL7 GSK3B ABCC3 HBE1 CHEK2 GCLC ACSL6 TTLL3 DHX30 TBCK DDX51 NAGK DGKZ DDX54 MAP3K9 CAMK1 MKNK1 MAPK12 GRK5 TIE1 PYGB GK5 ADCY2 OAS3 YES1 PEAK1 MAP3K2 NUBPL SLFN13 AKT2 EIF2AK2 P2RX4 ATP2B1 SHPK TP53 SCYL3 RFC2 ABCC9 ATP6V1A MSH6 LIG1 NMNAT1 NLRP3 MAPKAPK5 MYO1C AAK1 HIF1AN ABCB5 MYO5A	0.002218	0.09099	1.55
GO:0001786	phosphatidylserine binding	molecular_function	5/398	61/22360	TRIM72 GSDMA GSDMB GAS6 SYT12	0.002294	0.09337	4.6
GO:0043169	cation binding	molecular_function	111/398	4630/22360	DUOX1 MKNK1 DCTD SMAD2 COX5B STEAP2 DZANK1 ZNF677 KMT2D ZNF430 CHEK2 ZNF789 MDM4 USP44 ZNF573 ZNF345 HMCN2 APOBEC3D MYO5A PRR3 GNS ZNF669 ZNF274 ADCY2 ZNF70 ZNF445 ZFPM2 APP PDP2 ZNF772 ZNF441 RNF115 GCLC FDPS ZNF483 RNF7 TRIM25 CYP4V2 DRG1 PDE12 LIG1 ZBTB37 TP53 SLFN13 ZNF264 PGM2L1 ZNF570 EYA3 MAP3K2 PCDHB9 ZNF7 NUDT22 OGFOD1 FGD5 FTO TTYH1 ANKMY1 ZHX3 ADAR CYB561A3 ZNF766 HBE1 ZNF583 TTC3 TENM2 TNK2 ZNF286B RARG GALNT16 ITGA3 HIF1AN ZNF839 IKZF3 MSH6 ESYT3 TRABD2B ZMYM5 OAS3 STAC2 PDE4C ZNF80 GMEB1 MAPK12 PCDH9 ZNF800 ZKSCAN1 NT5DC3 DGKZ CYP51A1 GAS6 SUMF2 PCGF5 IRAK4 TRIM72 ZNF814 XIAP CYB5R4 SPTAN1 ZDHHC15 SYT12 PPM1K RNF166 ZNF8 NUBPL ASPA AKT2 P2RX4 ATP2B1 GALNT15 ZC3H14 ZNF106	0.002485	0.09881	1.35
GO:0003714	transcription corepressor activity	molecular_function	12/398	264/22360	NPAT N4BP2L2 NCOR1 DDX54 WNT4 ZNF274 ZFPM2 SCAI ZHX3 MNT TONSL MXD1	0.002745	0.1028	2.55
GO:0004708	MAP kinase kinase activity	molecular_function	6/398	88/22360	MAPKAPK5 F2R EIF2AK2 TAOK1 MAP3K9 MAP3K2	0.002892	0.1075	3.83
GO:0032869	cellular response to insulin stimulus	biological_process	11/398	234/22360	SYAP1 SMARCC1 GCLC PIK3R3 MYO5A ATP6V1A AKT2 TRIM72 RABGAP1 MYO1C ZNF106	0.002985	0.1102	2.64
GO:0046825	regulation of protein export from nucleus	biological_process	4/398	43/22360	TP53 CAMK1 GSK3B GAS6	0.002997	0.1099	5.23
GO:0009615	response to virus	biological_process	15/398	371/22360	HNRNPUL1 SLFN13 IFNAR1 EIF2AK2 C19ORF66 APOBEC3D CXCR4 TRIM25 OAS3 PDE12 ABCC9 NLRP3 ADAR TRAF3IP1 CCL5	0.003115	0.1134	2.27
GO:0097367	carbohydrate derivative binding	molecular_function	64/398	2447/22360	TAOK1 TTLL7 ABCC3 CHEK2 TNK2 CAMK1 MKNK1 GRK5 RAB3B DHX30 DDX51 TBCK NOA1 PRKAR2A RAB22A ADCY2 OAS3 YES1 LRPAP1 AAK1 PGF MYO5A SCYL3 GNS MSH6 RAB29 CTSS NMNAT1 GSK3B GCLC ACSL6 TTLL3 IRAK4 ABCC10 DDX54 MAP3K9 MAPK12 APP ARFRP1 NAGK DGKZ PEAK1 MAP3K2 RAB40B NUBPL SLFN13 AKT2 EIF2AK2 P2RX4 ATP2B1 TIE1 CNBD2 GK5 MAPKAPK5 DRG1 MYO1C ABCB5 SHPK TP53 RFC2 ABCC9 ATP6V1A LIG1 NLRP3	0.003164	0.1144	1.47
GO:1901985	positive regulation of protein acetylation	biological_process	4/398	44/22360	CAMK1 WBP2 TAOK1 FAM161A	0.00329	0.1181	5.11
GO:0004540	ribonuclease activity	molecular_function	8/398	146/22360	TSEN2 EXOSC6 PNPT1 ISG20L2 OAS3 DIS3L SLFN13 PDE12	0.003497	0.1213	3.08
GO:0004712	protein serine/threonine/tyrosine kinase activity	molecular_function	7/398	118/22360	MAP3K2 MAP3K9 EIF2AK2 TAOK1 TNK2 MAPKAPK5 F2R	0.003543	0.1221	3.33
GO:0031234	extrinsic component of cytoplasmic side of plasma membrane	cellular_component	6/398	92/22360	FARP1 ESYT3 STAC2 CNR2 YES1 SYAP1	0.003669	0.07709	3.66
GO:0030175	filopodium	cellular_component	7/398	119/22360	SRCIN1 ITGA3 APP MYO5A FARP1 TENM2 TTYH1	0.003724	0.07793	3.3

GO:0043227	membrane-bounded organelle	cellular_component	301/398	14259/22360	NOA1 STEAP2 IBA57 DUOX1 PRR11 MDM4 ZNF345 CS ZNF573 USP44 TMEM187 SEZ6 ARNT2 ZNF430 SPN ZNF789 RFTN1 N4BP2L2 GNS AAK1 ISG20L2 INTS6 ZNF445 SLC37A2 WHAMM LTBR ZNF274 NAGK ARFRP1 RPRD1B PLEKHB2 ZNF441 RCAN1 CORO7 NXN PDP2 CIART ABCC10 FDPS B3GALT1 QPRT TRIM25 SEC62 METTL21A SYAP1 SCAI CYP4V2 DRG1 MED17 MYO1C SLC35E2B C1ORF61 ZNF7 PSMD9 TMTC1 FGD5 SBNO2 BAALC UBE3C STYX EYA3 VPS28 RRP12 VPS33A ZHX3 DIS3L SNORA13 DDX51 RAB3B A4GALT GPR155 FTO TTYH1 TMEM106B SLC30A6 CAMK1 MLX STX16 GJC1 ANO6 C1ORF210 CRX F2R CD3G IKZF3 ESYT3 RARG MARVELD2 ZNF286B BRDT GALNT16 C1ORF174 PIGL KDSR WNT4 PLEKHA2 ZNF80 TAF8 IFNAR1 DPP9 C19ORF66 TRABD2B DDI2 ZMYM5 KRT18 RGS17 PNPT1 COQ6 SNX27 RPL7L1 MTFMT DDX54 ZKSCAN1 DESI1 GAS6 R3HCC1 H6PD MGLL IRAK4 TMEM170A USP28 SLC25A44 PPM1K RIPPLY3 ZNF814 TRIM72 ZDHHC15 BCAS3 ICOSLG GK5 NPAT FBXL4 ZNF106 UBXN2A TCF4 EIF2AK2 ATP2B1 HNRNPUL1 ASPA CARD19 RAB40B ELMOD1 VPS52 SRSF10 COG1 ADD2 ZNF677 SUMO2 TOR1AIP2 DHX30 NDUFAF7 CASP8 COX5B MKNK1 LETMD1 SMAD2 SAMD4B NSRP1 SLC35F6 CHEK2 KMT2D ATXN3 TAOK1 NMNAT1 FBXL18 WBP2 CTSS KLHL8 SCYL3 MCM3AP ICA1L COX6B2 SMCR8 APOBEC3D MYO5A SLC26A2 RRP1 YES1 LRPAP1 ZNF70 PCSK7 SLC35E1 BBS1 ZFPM2 RAB22A OTUD4 ZNF669 ACOT8 PIGR NDUFA10 APP ZNF772 RNF7 KDELC2 HERC4 ZNF483 SOX11 EMC10 ACSL6 RGS12 GSK3B NCOR1 LIG1 ZBTB37 ATP6V1A PDE12 TP53 C2ORF49 TONSL CALCRL GPAT4 MAPKAPK5 APOL1 NUDT22 LRTOMT TTI2 AP4M1 OGFOD1 ZNF264 MTAP MAP3K2 ZNF570 PRKAR2A ADAR ZNF766 CYB561A3 LGMN MMS22L GRK5 MR1 SYNRG MNT NAP1L6 LRRC8B TNK2 TTC3 TENM2 ZNF583 ABCC3 HP ACOX1 RAB29 COL8A1 MSH6 PKNOX1 POU5F1 SYP SMARCC1 FOXD2 SPC25 HIF1AN ITGA3 AP4E1 SLC39A13 OAS3 RILPL1 GMEB1 SON GABRB3 DGKZ SLC12A2 FITM2 SNX21 MAPK12 GNPTG ZNF800 LRRC57 C9ORF84 PCGF5 EPN1 CXCR4 CLCC1 SUMF2 FCF1 CYP51A1 SLC9A7 C9ORF78 NLRP3 MXD1 SYT12 RFC2 ZNF8 XIAP CCDC36 CWC25 CYB5R4 SLC33A1 SPTAN1 EXOSC6 ZC3H14 MLXIPL SLC25A26 TSEN2 PYGB P2RX4 MED22 FHDC1 NUBPL AKT2 UEVLD GALNT15	0.003866	0.08058	1.19
GO:0048156	tau protein binding	molecular_function	4/398	46/22360	SMAD2 GSK3B LGMN TAOK1	0.003938	0.08175	4.89
GO:0016740	transferase activity	molecular_function	81/398	3266/22360	HERC4 FDPS RNF7 GAS6 KDELC2 QPRT B3GALT1 IRAK4 CXCR4 TRIM25 GSK3B DGKZ NAGK CCL5 MTFMT RNF115 MAPK12 APP MAP3K9 GNPTG GK5 LRTOMT PYGB FBXL4 TIE1 TMTC1 UBE3C AKT2 MTAP PGM2L1 EIF2AK2 PEAK1 GALNT15 MAP3K2 RFC2 METTL21A SHPK TP53 TRIM72 SYAP1 XIAP PIK3R3 MAPKAPK5 ZDHHC15 BCAS3 GPAT4 SRCIN1 TNK2 CS USP44 F2R TTC3 KMT2D CHEK2 DCUN1D2 TAOK1 PRKAR2A ADAR SULT1B1 NDUFAF7 TBCK SUMO2 MKNK1 FTO CAMK1 A4GALT IBA57 GRK5 OAS3 ADCY2 YES1 TRMT10B PNPT1 NMNAT1 FBXL18 SMCR8 SCYL3 MCM3AP AS3MT GALNT16 AAK1	0.004004	0.08279	1.39
GO:0045071	negative regulation of viral genome replication	biological_process	5/398	69/22360	CCL5 APOBEC3D EIF2AK2 C19ORF66 OAS3	0.004113	0.08403	4.07
GO:0035196	production of miRNAs involved in gene silencing by miRNA	biological_process	4/398	47/22360	SMAD2 NCOR1 TP53 ADAR	0.004295	0.08539	4.78
GO:0002067	glandular epithelial cell differentiation	biological_process	4/398	48/22360	GPAT4 RARG GSK3B WNT4	0.004675	0.09223	4.68

GO:0050900	leukocyte migration	biological_process	18/398	501/22360	CXCR4 IRAK4 LGMN ADD2 CNR2 SLC12A2 YES1 GAS6 CCL5 ANO6 SPN PGF ITGA3 CX3CL1 C5AR2 APP P2RX4 CCR6	0.004939	0.09707	2.02
GO:0040014	regulation of multicellular organism growth	biological_process	5/398	72/22360	FTO LGMN APP RABGAP1 GPAT4	0.005019	0.09827	3.9
GO:0030119	AP-type membrane coat adaptor complex	cellular_component	4/398	49/22360	AP4E1 AP4M1 VPS33A SYNRG	0.005078	0.09686	4.59
GO:0050921	positive regulation of chemotaxis	biological_process	8/398	155/22360	CCR4 PGF LGMN ANO6 CCR6 P2RX4 CCL5 GAS6	0.005126	0.09741	2.9
GO:0051171	regulation of nitrogen compound metabolic process	biological_process	146/398	6472/22360	TRAF3IP1 SRCIN1 MNT CRX F2R TENM2 TNK2 ZNF583 DCUN1D2 ADAR ZHX3 PRKAR2A LGMN ZNF766 FTO CAMK1 MLX PAPLN OAS3 GMEB1 WNT4 SON ZNF80 TAF8 CX3CL1 TRABD2B ZMYM5 PNPT1 POU5F1 IKZF3 PKNOX1 MSH6 FOXD2 SMARCC1 RARG ZNF286B BRDT ITGA3 PGF HIF1AN CNKSR3 GAS6 PCGF5 IRAK4 CXCR4 CYP51A1 C9ORF78 C5AR2 MAPK12 DDX54 ITIH5 ZNF800 ZKSCAN1 ZC3H14 NPAT MLXIPL UBXN2A EIF2AK2 P2RX4 TCF4 AKT2 MED22 SRSF10 OR7D2 NLRP3 RFC2 MXD1 RIPPLY3 ZNF8 ZNF814 XIAP BCAS3 EXOSC6 SLC33A1 MDM4 USP44 ZNF573 ZNF345 CHEK2 ARNT2 KMT2D ZNF430 N4BP2L2 TAOK1 SPN ZNF789 ATXN3 SUMO2 ZNF677 CASP8 SAMD4B MKNK1 SMAD2 NSRP1 ZNF445 YES1 ZNF70 ADCY2 ZFPM2 ZNF669 OTUD4 LTBR ZNF274 WBP2 NMNAT1 SMCR8 PPP1R37 SOX11 ZNF483 TRIM25 EMC10 GCLC NCOR1 GSK3B CCL5 RPRD1B ACOT8 APP ZNF772 MAP3K9 ZNF441 RCAN1 CIART PDP2 NXN ZNF7 C1ORF61 FAM161A PSMD9 OGFOD1 SBNO2 STYX ZNF264 MAP3K2 EYA3 VPS28 ZNF570 ZBTB37 PDE12 TP53 PIK3R3 SCAI SYAP1 TONSL MAPKAPK5 MED17	0.005366	0.1016	1.27
GO:0003824	catalytic activity	molecular_function	177/398	8029/22360	TRMT10B RAB22A OTUD4 PCSK7 ADCY2 YES1 ISG20L2 AAK1 APOBEC3D MYO5A PPP1R37 SMCR8 MCM3AP SCYL3 GNS NMNAT1 CTSS FBXL18 PTPRM ATXN3 TTLL7 TAOK1 KMT2D CHEK2 CS USP44 CNR2 DCTD MKNK1 DUOX1 IBA57 STEAP2 COX5B NDUFAF7 SULT1B1 CASP8 CRYZL1 TOR1AIP2 DHX30 SUMO2 PEAK1 EYA3 MAP3K2 UBE3C MTAP SLFN13 STYX PGM2L1 OGFOD1 TMTC1 FGD5 LRTOMT NUDT22 MYO1C DRG1 MAPKAPK5 GPAT4 CYP4V2 SYAP1 PIK3R3 TP53 ATP6V1A ABCC9 PDE12 METTL21A LIG1 GSK3B RGS12 ACSL6 GCLC B3GALT1 QPRT TRIM25 ABCC10 HERC4 DYNC2LI1 FDPS CCDC125 KDELC2 RNF7 NXN PDP2 RCAN1 MAP3K9 NDUFA10 APP RNF115 ARFRP1 ACOT8 CCL5 DNAL1 NAGK RGS17 PNPT1 DDI2 TRABD2B CX3CL1 DPP9 PDE4C KDSR WNT4 PIGL OAS3 GALNT16 HIF1AN AS3MT MSH6 RAB29 ACOX1 DCUN1D2 HP FARP1 ABCC3 HBE1 TNK2 TTC3 F2R SRCIN1 PAPLN RABGAP1 CAMK1 FTO A4GALT GRK5 RAB3B DDX51 TBCK LGMN CYB561A3 DIS3L PRKAR2A ADAR UEVLD GALNT15 RAB40B ELMOD1 AKT2 ASPA VNN2 ATP2B1 EIF2AK2 FBXL4 PYGB TIE1 TSEN2 GK5 CYB5R4 EXOSC6 ZDHHC15 BCAS3 TRIM72 ABCB5 XIAP SHPK RFC2 NLRP3 PPM1K KIAA1191 CYP51A1 TTLL3 USP28 MGLL CXCR4 IRAK4 H6PD C9ORF84 DESI1 GAS6 GNPTG ITIH5 DDX54 MAPK12 MTFMT PLPP2 NT5DC3 COQ6 DGKZ	0.005558	0.1049	1.24
GO:0000186	activation of MAPKK activity	biological_process	5/398	74/22360	TAOK1 EIF2AK2 MAP3K9 MAP3K2 F2R	0.005702	0.1072	3.8
GO:0072678	T cell migration	biological_process	5/398	74/22360	SLC12A2 SPN CCL5 APP CCR6	0.005702	0.1072	3.8

GO:0051252	regulation of RNA metabolic process	biological_process	99/398	4178/22360	EXOSC6 MED17 SLC33A1 BCAS3 TONSL SCAI ZNF814 XIAP ZNF8 TP53 RIPPLY3 PDE12 MXD1 ZBTB37 NLRP3 OR7D2 SRSF10 ZNF570 MAP3K2 MED22 ZNF264 EIF2AK2 TCF4 SBNO2 NPAT PSMD9 MLXIPL ZC3H14 ZNF7 C1ORF61 CIART ZKSCAN1 ZNF800 DDX54 ZNF772 ZNF441 RCAN1 APP RPRD1B CCL5 C9ORF78 NCOR1 GCLC TRIM25 IRAK4 PCGF5 SOX11 ZNF483 GAS6 ITGA3 HIF1AN BRDT ZNF286B RARG FOXD2 SMARCC1 POU5F1 PKNOX1 IKZF3 WBP2 PNPT1 ZNF274 ZMYM5 ZNF669 CX3CL1 TAF8 ZFPM2 ZNF80 SON GMEB1 WNT4 ZNF70 OAS3 ZNF445 YES1 NSRP1 MLX CAMK1 SAMD4B FTO SMAD2 SUMO2 ZNF677 ZNF766 ZHX3 N4BP2L2 ZNF789 KMT2D ZNF430 ARNT2 CHEK2 ZNF583 F2R TENM2 ZNF573 ZNF345 CRX MNT MDM4	0.005785	0.1072	1.33
GO:2000404	regulation of T cell migration	biological_process	4/398	51/22360	CCL5 APP CCR6 SPN	0.00596	0.08259	4.41
GO:0002690	positive regulation of leukocyte chemotaxis	biological_process	6/398	101/22360	ANO6 PGF CCL5 CCR6 GAS6 LGMN	0.006004	0.08298	3.34
GO:0015297	antiporter activity	molecular_function	6/398	101/22360	SLC35E2B SLC35E1 SLC26A2 SLC37A2 SLC9A7 SLC9A4	0.006004	0.08298	3.34
GO:0090316	positive regulation of intracellular protein transport	biological_process	8/398	160/22360	RAB29 GAS6 AKT2 CAMK1 BCAS3 TP53 GSK3B MYO1C	0.006262	0.08451	2.81
GO:0001954	positive regulation of cell-matrix adhesion	biological_process	4/398	52/22360	GSK3B WNT4 PLEKHA2 CX3CL1	0.006441	0.0867	4.32
GO:0097159	organic cyclic compound binding	molecular_function	154/398	6908/22360	SAMD4B DUOX1 MKNK1 SMAD2 IBA57 NSRP1 NOA1 ZNF677 SUMO2 DHX30 CRYZL1 ARNT2 ZNF430 KMT2D CHEK2 TAOK1 RFTN1 TTLL7 ZNF789 ZNF573 CS ZNF345 RRP1 MYO5A APOBEC3D PRR3 AAK1 WBP2 NMNAT1 SCYL3 MCM3AP ZNF669 OTUD4 RAB22A ZNF274 TRMT10B ZNF70 ADCY2 ZNF445 YES1 ISG20L2 ZFPM2 APP CIART ZNF772 MAP3K9 ZNF441 RCAN1 ARFRP1 NAGK GCLC ACSL6 NCOR1 GSK3B SOX11 FDPS ABCC10 ZNF483 TRIM25 CYP4V2 MAPKAPK5 DRG1 MYO1C ABCC9 ATP6V1A ZBTB37 LIG1 TP53 SLFN13 ZNF264 ZNF570 PEAK1 MAP3K2 ZNF7 CAMK1 GRK5 MLX DIS3L RRP12 ADAR ZHX3 PRKAR2A RAB3B ZNF766 TBCK DDX51 ABCC3 HBE1 ZNF583 ACOX1 MNT TNK2 CRX ZNF286B SMARCC1 SYP FOXD2 RARG POU5F1 ZNF839 IKZF3 MSH6 PKNOX1 RAB29 C19ORF66 PNPT1 KRT18 OAS3 SON ZNF80 GMEB1 MAPK12 SPATS2 ZNF800 ZKSCAN1 DDX54 DGKZ COQ6 RPL7L1 CYP51A1 TTLL3 C9ORF84 R3HCC1 FCF1 PCGF5 H6PD IRAK4 ABCB5 ZNF814 EXOSC6 CYB5R4 RFC2 MXD1 NLRP3 SHPK ZNF8 AKT2 NUBPL HNRNPUL1 EIF2AK2 ATP2B1 P2RX4 TCF4 SRSF10 RAB40B GK5 ZC3H14 TIE1 ZNF106 MIR654 CNBD2 PYGB MLXIPL TSEN2	0.006501	0.08728	1.25
GO:0005654	nucleoplasm	cellular_component	93/398	3906/22360	ZHX3 ADAR CASP8 SUMO2 MKNK1 LETMD1 SMAD2 FTO MMS22L GRK5 NSRP1 MLX MDM4 MNT SLC35F6 ZNF345 USP44 TENM2 ARNT2 KMT2D CHEK2 ACOX1 SPN ZNF789 ATXN3 IKZF3 KLHL8 MSH6 POU5F1 NMNAT1 SMCR8 MCM3AP SMARCC1 RARG HIF1AN RILPL1 OAS3 ISG20L2 INTS6 SON ZFPM2 GMEB1 TAF8 C19ORF66 DDI2 RPRD1B DGKZ SNX27 MAPK12 CIART FDPS RNF7 USP28 FCF1 PCGF5 MGLL TRIM25 C9ORF78 GSK3B NCOR1 MXD1 RFC2 METTL21A LIG1 TP53 TONSL SYAP1 XIAP C2ORF49 SCAI DRG1 MED17 MYO1C EXOSC6 MAPKAPK5 CWC25 TTI2 NUDT22 ZC3H14 FBXL4 ZNF106 PSMD9 MLXIPL TSEN2 NPAT MED22 HNRNPUL1 STYX AKT2 SRSF10 EYA3 MAP3K2 ELMOD1	0.006512	0.0872	1.34
GO:0072676	lymphocyte migration	biological_process	7/398	131/22360	CCR6 APP CCL5 SPN GAS6 CX3CL1 SLC12A2	0.006517	0.08704	3
GO:0015108	chloride transmembrane transporter activity	molecular_function	7/398	131/22360	GABRB3 APOL1 ANO6 TTYH1 SLC12A2 CLCC1 SLC26A2	0.006517	0.08704	3

GO:0036094	small molecule binding	molecular_function	69/398	2764/22360	DHX30 DDX51 TBCK CRYZL1 RAB3B NOA1 PRKAR2A GRK5 DUOX1 CAMK1 MKNK1 TNK2 TTLL7 TAOK1 ACOX1 CHEK2 HBE1 ABCC3 SCYL3 RAB29 NMNAT1 MSH6 AAK1 HIF1AN MYO5A SYP YES1 OAS3 ADCY2 RAB22A NAGK COQ6 DGKZ ARFRP1 DDX54 MAP3K9 MAPK12 IRAK4 H6PD ABCC10 GSK3B GCLC TTLL3 ACSL6 TP53 SHPK LIG1 NLRP3 ABCC9 RFC2 ATP6V1A MAPKAPK5 MYO1C DRG1 ABCB5 OGFOD1 TIE1 CNBD2 PYGB GK5 RAB40B MAP3K2 PEAK1 ATP2B1 P2RX4 EIF2AK2 AKT2 SLFN13 NUBPL	0.006592	0.08759	1.4
GO:0030595	leukocyte chemotaxis	biological_process	11/398	259/22360	GAS6 C5AR2 CCR6 CCL5 CX3CL1 CNR2 SLC12A2 LGMN CXCR4 PGF ANO6	0.006597	0.08744	2.39
GO:0003725	double-stranded RNA binding	molecular_function	5/398	77/22360	RFTN1 EIF2AK2 ADAR DHX30 OAS3	0.006853	0.0906	3.65
GO:1902476	chloride transmembrane transport	biological_process	7/398	133/22360	GABRB3 APOL1 TTYH1 ANO6 CLCC1 SLC12A2 SLC26A2	0.007109	0.0935	2.96
GO:0002685	regulation of leukocyte migration	biological_process	10/398	228/22360	GAS6 C5AR2 CCR6 APP CCL5 P2RX4 ANO6 SPN LGMN PGF	0.00717	0.09407	2.46
GO:0019438	aromatic compound biosynthetic process	biological_process	108/398	4666/22360	LIG1 ZBTB37 TP53 SCAI TONSL GPAT4 DRG1 MED17 MAPKAPK5 C1ORF61 ZNF7 PSMD9 SBNO2 ZNF264 MTAP MAP3K2 ZNF570 NAGK CCL5 RPRD1B APP ZNF441 RCAN1 ZNF772 PDP2 CIART ZNF483 SOX11 QPRT TRIM25 ACSL6 GCLC NCOR1 NMNAT1 WBP2 MYO5A SLC26A2 YES1 INTS6 ZNF445 ADCY2 ZNF70 ZFPM2 ZNF669 ZNF274 ZNF677 SUMO2 IBA57 COX5B SMAD2 DCTD MDM4 ZNF345 ZNF573 CHEK2 ARNT2 ZNF430 KMT2D ZNF789 N4BP2L2 OR7D2 NLRP3 MXD1 RFC2 RIPPLY3 ZNF8 XIAP ZNF814 BCAS3 SLC33A1 MLXIPL NPAT TCF4 EIF2AK2 MED22 SRSF10 DDX54 ZNF800 ZKSCAN1 GAS6 PCGF5 IRAK4 IKZF3 PKNOX1 POU5F1 SMARCC1 FOXD2 RARG ZNF286B BRDT HIF1AN ITGA3 WNT4 GMEB1 ZNF80 PDE4C TAF8 CX3CL1 ZMYM5 ZHX3 ZNF766 CAMK1 MLX MNT CRX F2R TENM2 ZNF583	0.007917	0.1008	1.3
GO:1901028	regulation of mitochondrial outer membrane permeabilization involved in apoptotic signaling pathway	biological_process	4/398	55/22360	SLC35F6 GSK3B CASP8 TP53	0.008048	0.1022	4.09
GO:0004715	non-membrane spanning protein tyrosine kinase activity	molecular_function	4/398	55/22360	TNK2 YES1 PEAK1 EIF2AK2	0.008048	0.1022	4.09
GO:1990778	protein localization to cell periphery	biological_process	13/398	338/22360	BBS1 RAB3B STAC2 GAS6 RAB29 ARFRP1 RILPL1 AP4M1 ITGA3 KRT18 CACNB2 MYO5A AKT2	0.008302	0.1049	2.16

GO:0044237	cellular metabolic process	biological_process	256/398	12162/22360	MSH6 PKNOX1 POU5F1 RAB29 AS3MT SMARCC1 FOXD2 HIF1AN ITGA3 PGF OAS3 SON GMEB1 CX3CL1 PRKAR2A ADAR ZNF766 LGMN GRK5 MMS22L MNT SRCIN1 TNK2 TENM2 TTC3 ZNF583 HBE1 ACOX1 DCUN1D2 HP MXD1 RFC2 NLRP3 SHPK ZNF8 CCDC36 XIAP SLC33A1 CYB5R4 SPTAN1 EXOSC6 CWC25 ZC3H14 PYGB MLXIPL TSEN2 MED22 AKT2 NUBPL VNN2 P2RX4 UEVLD GALNT15 DGKZ FITM2 MAPK12 C5AR2 ZNF800 GNPTG C9ORF84 SUMF2 FCF1 PCGF5 CXCR4 C9ORF78 KLHL8 NMNAT1 FBXL18 CTSS WBP2 SMCR8 SCYL3 SLC26A2 RRP1 MYO5A APOBEC3D ZNF70 PCSK7 YES1 LRPAP1 ZFPM2 ZNF669 OTUD4 TRMT10B NDUFAF7 SULT1B1 CASP8 ZNF677 SUMO2 MKNK1 SMAD2 SAMD4B COX5B NSRP1 KMT2D CHEK2 PTPRM ATXN3 TAOK1 ATP6V1A PDE12 ZBTB37 LIG1 TP53 TONSL C2ORF49 MAPKAPK5 APOL1 CALCRL GPAT4 LRTOMT OGFOD1 ZNF264 MTAP ZNF570 MAP3K2 ACOT8 CCL5 NDUFA10 APP ZNF772 HERC4 ZNF483 SOX11 RNF7 KDELC2 EMC10 ACSL6 GCLC GSK3B NCOR1 IKZF3 ESYT3 ZNF286B BRDT RARG GALNT16 PIGL PLEKHA2 ZNF80 PDE4C WNT4 KDSR TRABD2B TAF8 PNPT1 ZMYM5 DIS3L SNORA13 VPS33A ZHX3 RAB3B DDX51 TBCK CSF2RA FTO CAMK1 A4GALT PAPLN MLX TRAF3IP1 F2R CRX FARP1 OR7D2 PPM1K RIPPLY3 TRIM72 ZNF814 ZDHHC15 BCAS3 GK5 FBXL4 TIE1 UBXN2A NPAT HNRNPUL1 ASPA TCF4 EIF2AK2 SRSF10 RAB40B NT5DC3 COQ6 MTFMT PLPP2 RPL7L1 ZKSCAN1 ITIH5 DDX54 GAS6 CNKSR3 USP28 H6PD IRAK4 MGLL TTLL3 GNS PPP1R37 CCR6 AAK1 ADCY2 ISG20L2 INTS6 ZNF445 ZNF274 LTBR NOA1 CRYZL1 DCTD DUOX1 IBA57 MDM4 CS ZNF345 ZNF573 USP44 ARNT2 ZNF430 SPN ZNF789 N4BP2L2 TTLL7 METTL21A CYP4V2 SYAP1 PIK3R3 SCAI DRG1 MED17 C1ORF61 ZNF7 SBNO2 PSMD9 FAM161A TMTC1 UBE3C STYX SLFN13 PGM2L1 PEAK1 VPS28 EYA3 RPRD1B NAGK RNF115 NXN CIART PDP2 MAP3K9 RCAN1 ZNF441 FDPS QPRT B3GALT1 TRIM25	0.008599	0.1071	1.18
GO:0036211	protein modification process	biological_process	105/398	4535/22360	MAPK12 C5AR2 MTFMT PLPP2 TTLL3 GAS6 CNKSR3 SUMF2 USP28 PCGF5 IRAK4 CXCR4 TRIM72 XIAP SPTAN1 ZDHHC15 NLRP3 PPM1K AKT2 EIF2AK2 UEVLD GALNT15 RAB40B FBXL4 TIE1 UBXN2A MLXIPL CSF2RA CAMK1 A4GALT GRK5 PRKAR2A ADAR RAB3B TBCK DCUN1D2 SRCIN1 TRAF3IP1 TNK2 F2R TTC3 GALNT16 HIF1AN PGF CX3CL1 TRABD2B PIGL APP NXN PDP2 MAP3K9 RCAN1 RPRD1B CCL5 RNF115 GCLC GSK3B NCOR1 HERC4 RNF7 KDELC2 EMC10 B3GALT1 TRIM25 SYAP1 PIK3R3 MAPKAPK5 APOL1 METTL21A TP53 UBE3C STYX PEAK1 VPS28 MAP3K2 EYA3 OGFOD1 PSMD9 FAM161A TMTC1 MKNK1 SMAD2 NDUFAF7 SUMO2 KMT2D CHEK2 PTPRM ATXN3 TAOK1 TTLL7 MDM4 USP44 AAK1 KLHL8 NMNAT1 FBXL18 WBP2 PPP1R37 SMCR8 SCYL3 OTUD4 ZNF274 LTBR ADCY2 YES1	0.008712	0.1083	1.3
GO:0006464	cellular protein modification process	biological_process	105/398	4535/22360	AAK1 NMNAT1 WBP2 FBXL18 KLHL8 SCYL3 PPP1R37 SMCR8 OTUD4 LTBR ZNF274 YES1 ADCY2 SMAD2 MKNK1 SUMO2 NDUFAF7 CHEK2 KMT2D ATXN3 TTLL7 TAOK1 PTPRM MDM4 USP44 SYAP1 PIK3R3 APOL1 MAPKAPK5 METTL21A TP53 UBE3C STYX MAP3K2 EYA3 PEAK1 VPS28 PSMD9 TMTC1 FAM161A OGFOD1 APP RCAN1 MAP3K9 NXN PDP2 CCL5 RPRD1B RNF115 GCLC GSK3B NCOR1 KDELC2 RNF7 HERC4 B3GALT1 TRIM25 EMC10 HIF1AN PGF GALNT16 CX3CL1 TRABD2B PIGL A4GALT GRK5 CSF2RA CAMK1 PRKAR2A ADAR TBCK RAB3B DCUN1D2 SRCIN1 TRAF3IP1 TNK2 F2R TTC3 XIAP TRIM72 ZDHHC15 SPTAN1 NLRP3 PPM1K EIF2AK2 AKT2 RAB40B UEVLD GALNT15 MLXIPL FBXL4 UBXN2A TIE1 C5AR2 MAPK12 PLPP2 MTFMT TTLL3 GAS6 CNKSR3 IRAK4 CXCR4 PCGF5 SUMF2 USP28	0.008712	0.1083	1.3
GO:1990869	cellular response to chemokine	biological_process	6/398	110/22360	SLC12A2 CX3CL1 CCL5 CCR6 CCR4 CXCR4	0.009333	0.09906	3.06

GO:0071375	cellular response to peptide hormone stimulus	biological_process	13/398	343/22360	SYAP1 SMARCC1 MYO5A PIK3R3 GCLC AKT2 TRIM72 RABGAP1 MYO1C PRKAR2A ADCY2 ATP6V1A ZNF106	0.009378	0.09933	2.13
GO:0055114	oxidation-reduction process	biological_process	34/398	1206/22360	H6PD CS CNR2 HP GSK3B KIAA1191 ACOX1 HBE1 CYP51A1 CYB561A3 CRYZL1 NOA1 COQ6 ACOT8 PDP2 NXN COX5B NDUFA10 STEAP2 DUOX1 FTO KDSR OGFOD1 PYGB PNPT1 UEVLD PGM2L1 AKT2 TRABD2B SHPK PPM1K HIF1AN CYB5R4 CYP4V2	0.009708	0.1026	1.58
GO:0005769	early endosome	cellular_component	16/398	458/22360	VPS28 RFTN1 RAB22A AKT2 SNX21 STEAP2 MYO5A APP F2R SNX27 CXCR4 FGD5 AP4M1 RAB29 VPS33A C1ORF210	0.009863	0.1022	1.96
GO:0002066	columnar/cuboidal epithelial cell development	biological_process	4/398	58/22360	GPAT4 LRTOMT RARG GSK3B	0.009922	0.1026	3.87
GO:0008408	3'-5' exonuclease activity	molecular_function	4/398	58/22360	ISG20L2 DIS3L PNPT1 PDE12	0.009922	0.1026	3.87
GO:0005813	centrosome	cellular_component	18/398	539/22360	KRT18 EYA3 GSK3B ODF2L CEP112 RABGAP1 FAM161A PCGF5 TSEN2 BBS1 TBCCD1 TRAF3IP1 PRKAR2A DYNC2LI1 RPRD1B RILPL1 DIS3L TTI2	0.01044	0.1071	1.88
GO:0012505	endomembrane system	cellular_component	115/398	5060/22360	GPAT4 CALCRL APOL1 SLC35E2B MYO1C SCAI SYAP1 CYP4V2 TP53 VPS28 FGD5 TMTC1 LRTOMT AP4M1 CORO7 APP PIGR PLEKHB2 ARFRP1 SEC62 ACSL6 B3GALT1 EMC10 KDELC2 AAK1 MYO5A ICA1L MCM3AP SCYL3 CTSS GNS LTBR WHAMM RAB22A LRPAP1 YES1 SLC35E1 PCSK7 SLC37A2 STEAP2 DUOX1 TOR1AIP2 COG1 RFTN1 ATXN3 CHEK2 TMEM187 SEZ6 ZDHHC15 SPTAN1 SLC33A1 CYB5R4 NLRP3 SYT12 VPS52 RAB40B CARD19 GALNT15 P2RX4 AKT2 FHDC1 UBXN2A PYGB DDX54 GNPTG SNX21 SNX27 FITM2 COQ6 SLC9A7 CYP51A1 IRAK4 MGLL CXCR4 EPN1 H6PD SUMF2 CLCC1 TMEM170A GAS6 SLC39A13 GALNT16 AP4E1 SYP ESYT3 COL8A1 RAB29 MSH6 PNPT1 IFNAR1 WNT4 KDSR PIGL MLX ANO6 GJC1 STX16 GRK5 A4GALT SYNRG SLC30A6 TMEM106B TTYH1 MR1 LGMN CYB561A3 RAB3B VPS33A RRP12 HP LRRC8B TENM2 F2R TNK2 C1ORF210	0.01054	0.1079	1.28
GO:0031252	cell leading edge	cellular_component	15/398	423/22360	BCAS3 AAK1 PTPRM MYO1C APP ATP2B1 MYO5A DUOX1 AKT2 RAB22A FGD5 CXCR4 SCYL3 SRCIN1 DGKZ	0.01056	0.1079	1.99
GO:0034707	chloride channel complex	cellular_component	4/398	59/22360	ANO6 TTYH1 GABRB3 CLCC1	0.01061	0.1082	3.81
GO:0051607	defense response to virus	biological_process	11/398	276/22360	NLRP3 APOBEC3D EIF2AK2 C19ORF66 TRAF3IP1 ADAR OAS3 SLFN13 ABCC9 PDE12 TRIM25	0.01064	0.1083	2.24
GO:0006821	chloride transport	biological_process	7/398	143/22360	SLC26A2 SLC12A2 TTYH1 CLCC1 ANO6 APOL1 GABRB3	0.01071	0.1088	2.75
GO:0060326	cell chemotaxis	biological_process	13/398	350/22360	P2RX4 CCR6 C5AR2 CX3CL1 PGF ANO6 CCL5 GAS6 SLC12A2 CNR2 CCR4 CXCR4 LGMN	0.01107	0.1122	2.09
GO:1904064	positive regulation of cation transmembrane transport	biological_process	7/398	144/22360	STAC2 CACNB2 ANO6 F2R CX3CL1 CNKSR3 CTSS	0.01114	0.1127	2.73
GO:0072659	protein localization to plasma membrane	biological_process	11/398	278/22360	AKT2 RILPL1 ARFRP1 MYO5A GAS6 STAC2 RAB3B CACNB2 KRT18 ITGA3 BBS1	0.01122	0.1133	2.22
GO:0030173	integral component of Golgi membrane	cellular_component	4/398	60/22360	SLC39A13 PCSK7 STEAP2 A4GALT	0.01133	0.1126	3.75
GO:0050920	regulation of chemotaxis	biological_process	10/398	244/22360	CCL5 P2RX4 CCR6 C5AR2 GAS6 PGF CCR4 CXCR4 LGMN ANO6	0.01157	0.1148	2.3
GO:0005516	calmodulin binding	molecular_function	9/398	210/22360	ADD2 MYO1C SPTAN1 MAPKAPK5 MYO5A ATP2B1 MKNK1 KCNN3 CAMK1	0.01163	0.1152	2.41
GO:0050709	negative regulation of protein secretion	biological_process	8/398	177/22360	F2R PSMD9 CYP51A1 CX3CL1 C5AR2 GAS6 NLRP3 SRCIN1	0.01167	0.1153	2.54
GO:0048471	perinuclear region of cytoplasm	cellular_component	23/398	749/22360	RAB40B VPS52 KRT18 TAOK1 PTPRM TAF8 EIF2AK2 P2RX4 SEZ6 CX3CL1 GSDMA TNK2 HIF1AN ITGA3 STX16 CYB5R4 SLC39A13 SYAP1 APP LGMN RAB3B RAB29 VPS33A	0.01169	0.1153	1.73

GO:1901652	response to peptide	biological_process	18/398	546/22360	ZNF106 TP53 LGMN ATP6V1A ADCY2 PRKAR2A PNPT1 MYO1C RABGAP1 GSK3B TRIM72 AKT2 APP GCLC PIK3R3 MYO5A SMARCC1 SYAP1	0.01186	0.1168	1.85
GO:0071417	cellular response to organonitrogen compound	biological_process	20/398	627/22360	P2RX4 GCLC AKT2 RABGAP1 GABRB3 GSK3B ADCY2 ZNF106 APP PIK3R3 MYO5A SMARCC1 SYAP1 TRIM72 MYO1C NLRP3 PRKAR2A ATP6V1A TP53 LGMN	0.01196	0.1175	1.79
GO:0015698	inorganic anion transport	biological_process	9/398	211/22360	SLC26A2 SLC37A2 SLC12A2 TTYH1 CLCC1 ANO6 APOL1 LRRC8B GABRB3	0.012	0.1177	2.4
GO:0016482	cytosolic transport	biological_process	8/398	178/22360	RAB29 DENND2A ARFRP1 AP4M1 VPS52 CORO7 STX16 SRSF10	0.01207	0.1182	2.52
GO:0061629	RNA polymerase II-specific DNA-binding transcription factor binding	molecular_function	11/398	282/22360	RARG ARNT2 BCAS3 NCOR1 DDX54 HIF1AN GSK3B MED17 WBP2 CRX BBS1	0.01246	0.1084	2.19
GO:0016605	PML body	cellular_component	6/398	117/22360	SPN CHEK2 TP53 SUMO2 CIART TENM2	0.01276	0.1109	2.88
GO:0046824	positive regulation of nucleocytoplasmic transport	biological_process	4/398	62/22360	GSK3B GAS6 TP53 CAMK1	0.01288	0.1104	3.62
GO:0061951	establishment of protein localization to plasma membrane	biological_process	4/398	62/22360	RILPL1 BBS1 ARFRP1 KRT18	0.01288	0.1104	3.62
GO:0005829	cytosol	cellular_component	124/398	5547/22360	SHPK MXD1 NLRP3 SPTAN1 EXOSC6 XIAP FBXL4 CNBD2 UBXN2A ZNF106 TSEN2 MLXIPL SRSF10 VPS52 ELMOD1 CARD19 AKT2 ASPA EIF2AK2 SNX27 RPL7L1 SPATS2 MAPK12 SSTR2 USP28 IRAK4 MGLL EPN1 C9ORF78 TTLL3 MSH6 IKZF3 POU5F1 RAB29 HIF1AN AS3MT SPC25 PDE4C STAC2 GSDMB OAS3 RILPL1 PNPT1 CC2D2A DDI2 KRT18 DPP9 C19ORF66 RAB3B DIS3L PRKAR2A STX16 ANO6 MLX RABGAP1 CAMK1 GRK5 MMS22L TTC3 ACOX1 FARP1 HBE1 TP53 ATP6V1A PDE12 MYO1C DRG1 MAPKAPK5 SYAP1 PIK3R3 OGFOD1 BAALC PSMD9 TTI2 AP4M1 VPS28 MAP3K2 MTAP TMEM266 STYX PSTPIP2 PGM2L1 RNF115 ARFRP1 ACOT8 RPRD1B NAGK NXN CORO7 APP DENND2A QPRT TRIM25 HERC4 FDPS RNF7 SEC62 GSK3B NCOR1 RGS12 GCLC MCM3AP FBXL18 AAK1 MYO5A BBS1 YES1 TRMT10B WHAMM SULT1B1 CASP8 ADD2 CRYZL1 DHX30 SMAD2 DCTD MKNK1 SAMD4B STEAP2 SLC35F6 ATXN3 TTLL7 TAOK1 GSDMA	0.01306	0.1116	1.26
GO:0055085	transmembrane transport	biological_process	47/398	1822/22360	MYO5A ABCB5 SLC26A2 APOL1 SLC39A13 SLC35E2B CACNB2 SLC33A1 CTSS ABCC9 ATP6V1A P2RX4 ATP2B1 AKT2 KCNN3 CX3CL1 GABRB3 PNPT1 YES1 SLC35E1 SLC37A2 SLC25A26 PSMD9 STAC2 APP COX5B STEAP2 SLC30A6 TTYH1 GPR155 ANO6 GJC1 SLC9A4 SLC12A2 KCNJ15 ADD2 ABCC3 SLC25A44 SLC9A7 SEC62 CNKSR3 GAS6 ABCC10 LRRC8B F2R CLCC1 SLC35F6	0.0134	0.1143	1.45
GO:0043467	regulation of generation of precursor metabolites and energy	biological_process	7/398	149/22360	NOA1 PDE12 AKT2 GSK3B MLXIPL NCOR1 PNPT1	0.01346	0.1146	2.64
GO:0032870	cellular response to hormone stimulus	biological_process	23/398	759/22360	KMT2D AKT2 ATP2B1 GCLC CHEK2 NCOR1 RABGAP1 ADCY2 ZNF106 SSTR2 TRIM72 RARG SYAP1 SMARCC1 PIK3R3 MYO5A MYO1C DDX54 PGF ATP6V1A PRKAR2A CTSS WBP2	0.01358	0.1155	1.7
GO:0046847	filopodium assembly	biological_process	4/398	63/22360	TENM2 TTYH1 FGD5 BCAS3	0.0137	0.1163	3.57
GO:0098659	inorganic cation import across plasma membrane	biological_process	5/398	90/22360	SLC9A7 SLC9A4 ABCC9 KCNJ15 SLC12A2	0.01391	0.1179	3.12
GO:0043244	regulation of protein complex disassembly	biological_process	6/398	120/22360	SPTAN1 SMCR8 TMOD3 OGFOD1 ADD2 TAOK1	0.01449	0.1226	2.81
GO:0010811	positive regulation of cell-substrate adhesion	biological_process	6/398	120/22360	WNT4 GSK3B ITGA3 COL8A1 CX3CL1 PLEKHA2	0.01449	0.1226	2.81

GO:0005930	axoneme	cellular_component	6/398	120/22360	PRKAR2A TTLL3 BBS1 DNAL1 TRAF3IP1 DYNC2LI1	0.01449	0.1226	2.81
GO:0097194	execution phase of apoptosis	biological_process	5/398	91/22360	NMNAT1 TP53 TAOK1 XKR4 CASP8	0.01461	0.1214	3.09
GO:0002792	negative regulation of peptide secretion	biological_process	8/398	184/22360	SRCIN1 GAS6 NLRP3 C5AR2 CX3CL1 CYP51A1 PSMD9 F2R	0.01472	0.1221	2.44
GO:0008286	insulin receptor signaling pathway	biological_process	7/398	152/22360	SMARCC1 PIK3R3 ATP6V1A AKT2 TRIM72 RABGAP1 ZNF106	0.01502	0.1244	2.59
GO:0003014	renal system process	biological_process	6/398	121/22360	ITGA3 LGMN GAS6 PRKAR2A F2R ADCY2	0.0151	0.1249	2.79
GO:0097014	ciliary plasm	cellular_component	6/398	121/22360	BBS1 TTLL3 PRKAR2A TRAF3IP1 DNAL1 DYNC2LI1	0.0151	0.1249	2.79
GO:0005881	cytoplasmic microtubule	cellular_component	4/398	65/22360	DYNC2LI1 FHDC1 BCAS3 FAM161A	0.01546	0.1275	3.46
GO:0031228	intrinsic component of Golgi membrane	cellular_component	4/398	65/22360	STEAP2 A4GALT SLC39A13 PCSK7	0.01546	0.1275	3.46
GO:0032868	response to insulin	biological_process	11/398	291/22360	ATP6V1A ZNF106 MYO5A PIK3R3 GCLC SMARCC1 SYAP1 TRIM72 AKT2 RABGAP1 MYO1C	0.01565	0.1286	2.12
GO:0008134	transcription factor binding	molecular_function	21/398	685/22360	WBP2 NLRP3 TP53 SUMO2 RARG SMAD2 DDX54 BCAS3 MLX HIF1AN MED17 DRG1 BBS1 MLXIPL CRX PSMD9 ZFPM2 TCF4 ARNT2 NCOR1 GSK3B	0.01567	0.1286	1.72
GO:0072341	modified amino acid binding	molecular_function	5/398	93/22360	TRIM72 GSDMA SYT12 GSDMB GAS6	0.0161	0.121	3.02
GO:0032388	positive regulation of intracellular transport	biological_process	9/398	221/22360	CAMK1 AKT2 RAB29 GAS6 CACNB2 MYO1C TP53 BCAS3 GSK3B	0.01615	0.1212	2.29
GO:0005253	anion channel activity	molecular_function	6/398	123/22360	APOL1 ANO6 TTYH1 CLCC1 LRRC8B GABRB3	0.01638	0.1227	2.74
GO:0006893	Golgi to plasma membrane transport	biological_process	4/398	66/22360	STEAP2 BBS1 ARFRP1 KRT18	0.0164	0.1227	3.4
GO:0097345	mitochondrial outer membrane permeabilization	biological_process	4/398	66/22360	TP53 CASP8 GSK3B SLC35F6	0.0164	0.1227	3.4
GO:0006139	nucleobase-containing compound metabolic process	biological_process	146/398	6700/22360	ZC3H14 NPAT TSEN2 MLXIPL HNRNPUL1 MED22 EIF2AK2 TCF4 SRSF10 RFC2 MXD1 NLRP3 OR7D2 ZNF8 RIPPLY3 CCDC36 ZNF814 XIAP EXOSC6 SLC33A1 CWC25 BCAS3 C9ORF84 GAS6 FCF1 USP28 IRAK4 PCGF5 C9ORF78 MTFMT RPL7L1 ZKSCAN1 ZNF800 DDX54 OAS3 ZNF80 PDE4C SON GMEB1 WNT4 CX3CL1 TAF8 PNPT1 ZMYM5 POU5F1 MSH6 PKNOX1 IKZF3 BRDT ZNF286B RARG SMARCC1 FOXD2 ITGA3 HIF1AN MNT TENM2 F2R CRX ZNF583 SNORA13 DIS3L ADAR ZHX3 ZNF766 DDX51 CAMK1 FTO MMS22L MLX ZNF7 C1ORF61 SBNO2 TMTC1 PSMD9 MTAP SLFN13 ZNF264 PGM2L1 ZNF570 MAP3K2 EYA3 PDE12 ZBTB37 LIG1 TP53 TONSL SCAI C2ORF49 MAPKAPK5 MED17 DRG1 GPAT4 SOX11 ZNF483 TRIM25 QPRT GCLC ACSL6 NCOR1 RPRD1B ACOT8 CCL5 NAGK APP CIART PDP2 ZNF772 RCAN1 ZNF441 ZNF70 ADCY2 ZNF445 INTS6 ISG20L2 YES1 ZFPM2 ZNF669 OTUD4 TRMT10B ZNF274 WBP2 NMNAT1 RRP1 SLC26A2 CCR6 APOBEC3D MDM4 ZNF573 ZNF345 ZNF430 KMT2D ARNT2 CHEK2 TAOK1 N4BP2L2 ATXN3 ZNF789 SULT1B1 ZNF677 SUMO2 SAMD4B SMAD2 DCTD COX5B NSRP1	0.01646	0.1224	1.22
GO:0050808	synapse organization	biological_process	15/398	445/22360	LGMN ADD2 F2R RAB29 SRCIN1 PCDHB9 GABRB3 ITGA3 CACNB2 MYO5A APP FARP1 CAMK1 CX3CL1 SEZ6	0.01647	0.1223	1.89
GO:0090501	RNA phosphodiester bond hydrolysis	biological_process	8/398	188/22360	TSEN2 PNPT1 EXOSC6 ISG20L2 PDE12 SLFN13 OAS3 DIS3L	0.01673	0.1241	2.39

GO:0000981	DNA-binding transcription factor activity, RNA polymerase II-specific	molecular_function	44/398	1711/22360	ZBTB37 POU5F1 IKZF3 MXD1 PKNOX1 TP53 ZNF814 FOXD2 SMARCC1 RARG ZNF286B ZNF445 ZNF7 ZNF70 GMEB1 MLXIPL ZFPM2 ZNF80 TCF4 ZNF669 ZNF264 ZMYM5 ZNF274 ZNF570 ZHX3 ZNF677 ZNF766 SMAD2 ZNF772 MLX ZNF441 ZNF800 ZKSCAN1 MNT SOX11 ZNF483 CRX ZNF345 ZNF583 ARNT2 KMT2D ZNF430 NCOR1 ZNF789	0.01725	0.1274	1.44
GO:1903900	regulation of viral life cycle	biological_process	8/398	189/22360	TRIM25 ADAR CCL5 EIF2AK2 C19ORF66 APOBEC3D PDE12 OAS3	0.01726	0.1273	2.38
GO:0034767	positive regulation of ion transmembrane transport	biological_process	7/398	156/22360	CTSS CNKSR3 CX3CL1 F2R ANO6 CACNB2 STAC2	0.01731	0.1275	2.52
GO:0035869	ciliary transition zone	cellular_component	4/398	67/22360	DYNC2LI1 CC2D2A TRAF3IP1 FAM161A	0.01737	0.1277	3.35
GO:0004527	exonuclease activity	molecular_function	5/398	95/22360	DIS3L EXOSC6 PDE12 PNPT1 ISG20L2	0.01768	0.1298	2.96
GO:0032588	trans-Golgi network membrane	cellular_component	7/398	157/22360	VPS52 COG1 APP ARFRP1 STX16 AP4E1 AP4M1	0.01792	0.1314	2.5
GO:0035023	regulation of Rho protein signal transduction	biological_process	7/398	157/22360	FGD5 ITGA3 ARHGEF39 F2R SCAI CCDC125 FARP1	0.01792	0.1314	2.5
GO:0072091	regulation of stem cell proliferation	biological_process	4/398	68/22360	CX3CL1 SOX11 EIF2AK2 N4BP2L2	0.01839	0.1345	3.3
GO:0004896	cytokine receptor activity	molecular_function	5/398	96/22360	CSF2RA CXCR4 CCR6 CCR4 IFNAR1	0.01851	0.1325	2.93
GO:0022857	transmembrane transporter activity	molecular_function	37/398	1398/22360	ANO6 GJC1 APP COX5B TTYH1 SLC30A6 SLC9A4 SLC12A2 KCNJ15 SLC9A7 SLC25A44 ABCC3 LRRC8B SLC35F6 CLCC1 CNKSR3 GAS6 ABCC10 SLC39A13 SLC35E2B APOL1 SLC33A1 CACNB2 MYO5A ABCB5 SLC26A2 CTSS ABCC9 ATP6V1A GABRB3 P2RX4 ATP2B1 KCNN3 SLC25A26 STAC2 SLC35E1 SLC37A2	0.01851	0.1325	1.49
GO:0006725	cellular aromatic compound metabolic process	biological_process	150/398	6932/22360	ZNF70 ADCY2 INTS6 YES1 ISG20L2 ZNF445 ZFPM2 ZNF669 OTUD4 TRMT10B ZNF274 NMNAT1 WBP2 SLC26A2 RRP1 CCR6 MYO5A APOBEC3D MDM4 ZNF345 ZNF573 KMT2D ZNF430 ARNT2 CHEK2 ZNF789 ATXN3 N4BP2L2 TAOK1 SULT1B1 SUMO2 ZNF677 DCTD SMAD2 DUOX1 SAMD4B IBA57 COX5B NSRP1 LRTOMT C1ORF61 ZNF7 SBNO2 PSMD9 TMTC1 ZNF264 MTAP SLFN13 PGM2L1 ZNF570 MAP3K2 EYA3 PDE12 ZBTB37 LIG1 TP53 TONSL SCAI C2ORF49 MED17 DRG1 MAPKAPK5 GPAT4 ZNF483 SOX11 QPRT TRIM25 ACSL6 GCLC NCOR1 ACOT8 RPRD1B CCL5 NAGK APP CIART PDP2 ZNF441 RCAN1 ZNF772 OAS3 PDE4C ZNF80 SON WNT4 GMEB1 CX3CL1 TAF8 PNPT1 ZMYM5 PKNOX1 MSH6 IKZF3 POU5F1 BRDT ZNF286B RARG FOXD2 SMARCC1 HIF1AN ITGA3 MNT F2R TENM2 CRX ZNF583 SNORA13 DIS3L ADAR ZHX3 ZNF766 DDX51 CAMK1 FTO MMS22L MLX ZC3H14 TSEN2 MLXIPL NPAT HNRNPUL1 MED22 TCF4 EIF2AK2 SRSF10 MXD1 RFC2 NLRP3 OR7D2 ZNF8 RIPPLY3 CCDC36 XIAP ZNF814 SLC33A1 EXOSC6 BCAS3 CWC25 C9ORF84 GAS6 FCF1 USP28 IRAK4 PCGF5 C9ORF78 MTFMT RPL7L1 ZKSCAN1 ZNF800 DDX54	0.01878	0.1341	1.22
GO:0007050	cell cycle arrest	biological_process	10/398	262/22360	AKT2 MDM4 MAPK12 CHEK2 WHAMM GAS6 PNPT1 TP53 PRR11 MLXIPL	0.01879	0.134	2.14
GO:0031589	cell-substrate adhesion	biological_process	13/398	374/22360	CX3CL1 TTYH1 WHAMM PEAK1 ATXN3 GSK3B ITGA3 BCAS3 GAS6 SRCIN1 COL8A1 PLEKHA2 WNT4	0.01881	0.1339	1.95
GO:0018105	peptidyl-serine phosphorylation	biological_process	11/398	299/22360	MLXIPL CNKSR3 GAS6 GSK3B MAPKAPK5 APP CHEK2 CAMK1 AKT2 MKNK1 MAPK12	0.01899	0.135	2.07
GO:0009898	cytoplasmic side of plasma membrane	cellular_component	7/398	159/22360	FARP1 CNR2 ATP2B1 SYAP1 YES1 ESYT3 STAC2	0.01918	0.1362	2.47
GO:0007030	Golgi organization	biological_process	7/398	160/22360	MYO5A RAB29 COG1 FHDC1 BCAS3 STX16 UBXN2A	0.01984	0.1407	2.46
GO:0036064	ciliary basal body	cellular_component	6/398	128/22360	DYNC2LI1 ODF2L RILPL1 TRAF3IP1 FAM161A BBS1	0.01993	0.1411	2.63
GO:0031625	ubiquitin protein ligase binding	molecular_function	12/398	339/22360	LTBR GSK3B ATXN3 SMAD2 CHEK2 CASP8 TNK2 SUMO2 TP53 CXCR4 POU5F1 PRKAR2A	0.02012	0.1423	1.99

GO:0010810	regulation of cell-substrate adhesion	biological_process	9/398	229/22360	CX3CL1 COL8A1 PLEKHA2 PEAK1 WNT4 GSK3B ATXN3 BCAS3 ITGA3	0.02021	0.1427	2.21
GO:1904427	positive regulation of calcium ion transmembrane transport	biological_process	4/398	70/22360	F2R STAC2 CX3CL1 CACNB2	0.02054	0.1354	3.21
GO:0019904	protein domain specific binding	molecular_function	23/398	790/22360	CASP8 ICA1L TP53 PRKAR2A HIF1AN ITGA3 SSTR2 SMAD2 SYP MYO5A APP TNK2 SON PLEKHA2 ZNF106 CRX AP4M1 SRCIN1 ACOX1 SRSF10 CARD19 STYX ATP2B1	0.02111	0.1369	1.64
GO:0051603	proteolysis involved in cellular protein catabolic process	biological_process	23/398	790/22360	VPS28 ATXN3 GSK3B STYX UBE3C GCLC UBXN2A TTC3 USP28 USP44 FBXL4 TRIM25 PSMD9 RNF7 TRIM72 SMARCC1 RNF115 CASP8 SUMO2 LGMN KLHL8 CTSS FBXL18	0.02111	0.1369	1.64
GO:0035257	nuclear hormone receptor binding	molecular_function	7/398	162/22360	WBP2 RARG MED17 NCOR1 DDX54 BCAS3 CRX	0.02121	0.1372	2.43
GO:0098657	import into cell	biological_process	22/398	748/22360	LRPAP1 XKR4 TNK2 EPN1 AKT2 RAB22A VPS28 HP SLC9A7 ABCC9 KCNJ15 SLC12A2 SLC9A4 RAB3B SYNRG APP SYP STEAP2 ANO6 APOL1 AAK1 CALCRL	0.02131	0.1377	1.65
GO:0005794	Golgi apparatus	cellular_component	46/398	1828/22360	CHEK2 SLC9A7 GAS6 CLCC1 TENM2 F2R B3GALT1 SLC30A6 SYNRG STEAP2 A4GALT APP STX16 GNPTG CORO7 DDX54 ARFRP1 COQ6 COG1 RAB3B FHDC1 WHAMM GALNT15 RAB40B VPS52 LTBR PCSK7 SLC35E1 AP4M1 YES1 LRPAP1 UBXN2A WNT4 FGD5 SYAP1 MYO5A SLC33A1 AP4E1 GALNT16 SLC39A13 SLC35E2B ZDHHC15 MSH6 NLRP3 RAB29 SCYL3	0.02143	0.1383	1.41
GO:0051928	positive regulation of calcium ion transport	biological_process	6/398	130/22360	CACNB2 STAC2 CX3CL1 F2R CCL5 P2RX4	0.02149	0.1385	2.59
GO:0005634	nucleus	cellular_component	180/398	8498/22360	NAP1L6 CRX TNK2 TTC3 TENM2 MNT ACOX1 ZNF583 DDX51 ZNF766 ZHX3 ADAR RRP12 SNORA13 DIS3L MLX MMS22L GRK5 CAMK1 FTO GMEB1 ZNF80 SON PLEKHA2 C1ORF174 OAS3 RILPL1 DDI2 ZMYM5 KRT18 RGS17 PNPT1 TAF8 C19ORF66 DPP9 MSH6 PKNOX1 IKZF3 POU5F1 HIF1AN RARG SPC25 SMARCC1 FOXD2 BRDT ZNF286B MGLL IRAK4 PCGF5 EPN1 TMEM170A CLCC1 FCF1 USP28 DESI1 R3HCC1 C9ORF84 C9ORF78 SNX27 RPL7L1 DGKZ DDX54 ZKSCAN1 ZNF800 MAPK12 TSEN2 MLXIPL NPAT FBXL4 ZNF106 ZC3H14 CARD19 ELMOD1 RAB40B SRSF10 TCF4 P2RX4 ATP2B1 EIF2AK2 MED22 HNRNPUL1 AKT2 ASPA ZNF8 RIPPLY3 NLRP3 MXD1 RFC2 CWC25 BCAS3 EXOSC6 XIAP ZNF814 CCDC36 ZNF345 CS SLC35F6 ZNF573 USP44 MDM4 ATXN3 ZNF789 SPN TAOK1 N4BP2L2 CHEK2 ZNF430 KMT2D ARNT2 TOR1AIP2 DHX30 ZNF677 SUMO2 CASP8 PRR11 NSRP1 SMAD2 MKNK1 LETMD1 SAMD4B ZFPM2 INTS6 ISG20L2 ZNF445 ZNF70 ZNF274 OTUD4 ZNF669 MCM3AP SMCR8 NMNAT1 WBP2 FBXL18 KLHL8 AAK1 APOBEC3D RRP1 TRIM25 RNF7 ZNF483 HERC4 FDPS SOX11 GSK3B NCOR1 RGS12 RPRD1B RCAN1 ZNF441 ZNF772 NXN CIART APP PSMD9 SBNO2 BAALC OGFOD1 C1ORF61 NUDT22 ZNF7 TTI2 EYA3 MAP3K2 ZNF570 UBE3C ZNF264 MTAP STYX TP53 METTL21A LIG1 ZBTB37 MED17 MYO1C DRG1 MAPKAPK5 SYAP1 SCAI C2ORF49 TONSL	0.02163	0.1392	1.19
GO:0046982	protein heterodimerization activity	molecular_function	17/398	541/22360	ARNT2 SMAD2 TAF8 TCF4 GCLC MLX PGF ITGA3 ADCY2 IKZF3 PKNOX1 CD3G ZHX3 TENM2 MLXIPL ADD2 TP53	0.02191	0.1409	1.77
GO:0009725	response to hormone	biological_process	29/398	1058/22360	CASP8 ATP6V1A PRKAR2A WBP2 CTSS MYO1C PGF DDX54 ITGA3 SSTR2 TRIM72 RARG STEAP2 SMARCC1 SYAP1 PIK3R3 MYO5A ZNF106 ADCY2 PNPT1 NCOR1 RABGAP1 KMT2D ARNT2 MTAP AKT2 ATP2B1 GCLC CHEK2	0.02277	0.1462	1.54
GO:1902110	positive regulation of mitochondrial membrane permeability involved in	biological_process	4/398	72/22360	CASP8 TP53 GSK3B SLC35F6	0.02285	0.1465	3.12

	apoptotic process							
GO:0070098	chemokine-mediated signaling pathway	biological_process	5/398	101/22360	CX3CL1 CCR4 CCL5 CCR6 CXCR4	0.0231	0.1465	2.78
GO:2001141	regulation of RNA biosynthetic process	biological_process	89/398	3918/22360	WBP2 IKZF3 PKNOX1 POU5F1 FOXD2 SMARCC1 RARG ZNF286B BRDT HIF1AN ITGA3 YES1 ZNF445 ZNF70 WNT4 GMEB1 ZNF80 ZFPM2 TAF8 CX3CL1 ZNF669 ZMYM5 ZNF274 ZHX3 ZNF766 SUMO2 ZNF677 SMAD2 CAMK1 MLX MDM4 MNT CRX ZNF345 TENM2 F2R ZNF573 ZNF583 CHEK2 ARNT2 KMT2D ZNF430 ZNF789 N4BP2L2 OR7D2 NLRP3 ZBTB37 MXD1 RIPPLY3 ZNF8 TP53 XIAP ZNF814 SCAI TONSL BCAS3 SLC33A1 MED17 C1ORF61 ZNF7 PSMD9 MLXIPL NPAT SBNO2 TCF4 EIF2AK2 MED22 ZNF264 MAP3K2 ZNF570 SRSF10 CCL5 RPRD1B APP RCAN1 ZNF441 ZNF772 DDX54 ZNF800 ZKSCAN1 CIART GAS6 ZNF483 SOX11 PCGF5 IRAK4 TRIM25 GCLC NCOR1	0.02371	0.1502	1.28
GO:0040017	positive regulation of locomotion	biological_process	19/398	629/22360	SPN ARHGEF39 AKT2 P2RX4 F2R EMC10 GAS6 MYO1C ANO6 BCAS3 ITGA3 PGF DUOX1 CCR6 PIK3R3 APP LGMN CCR4 CCL5	0.02383	0.1508	1.7
GO:1903829	positive regulation of cellular protein localization	biological_process	12/398	347/22360	GSK3B ITGA3 BCAS3 MYO1C VPS28 CAMK1 AKT2 TP53 STAC2 CASP8 GAS6 RAB29	0.02389	0.151	1.94
GO:0001952	regulation of cell-matrix adhesion	biological_process	6/398	133/22360	WNT4 GSK3B BCAS3 PEAK1 CX3CL1 PLEKHA2	0.02401	0.1515	2.53
GO:0043627	response to estrogen	biological_process	4/398	73/22360	KMT2D BCAS3 TRIM25 WBP2	0.02407	0.1517	3.08
GO:0019898	extrinsic component of membrane	cellular_component	11/398	310/22360	PIK3R3 SYAP1 FARP1 AAK1 NOA1 YES1 COQ6 CNR2 MGLL STAC2 ESYT3	0.02446	0.1533	1.99
GO:1905477	positive regulation of protein localization to membrane	biological_process	6/398	134/22360	ITGA3 TP53 CASP8 AKT2 MYO1C STAC2	0.02489	0.1558	2.52
GO:0005802	trans-Golgi network	cellular_component	11/398	311/22360	AP4E1 STX16 SLC9A7 CORO7 VPS52 AP4M1 PCSK7 ARFRP1 RAB29 APP COG1	0.02501	0.1563	1.99
GO:0044257	cellular protein catabolic process	biological_process	24/398	846/22360	SMARCC1 TRIM72 CTSS FBXL18 KLHL8 SUMO2 LGMN CASP8 RNF115 GCLC CHEK2 STYX UBE3C GSK3B ATXN3 VPS28 RNF7 TRIM25 PSMD9 UBXN2A USP44 USP28 TTC3 FBXL4	0.02502	0.1562	1.59
GO:0042147	retrograde transport, endosome to Golgi	biological_process	5/398	103/22360	STX16 ARFRP1 VPS52 DENND2A RAB29	0.02515	0.1459	2.73
GO:2000401	regulation of lymphocyte migration	biological_process	4/398	74/22360	APP CCL5 CCR6 SPN	0.02534	0.146	3.04
GO:1902686	mitochondrial outer membrane permeabilization involved in programmed cell death	biological_process	4/398	74/22360	CASP8 TP53 SLC35F6 GSK3B	0.02534	0.146	3.04
GO:0032482	Rab protein signal transduction	biological_process	4/398	74/22360	RAB3B RAB22A RAB40B RAB29	0.02534	0.146	3.04
GO:0070988	demethylation	biological_process	4/398	74/22360	OTUD4 CYP51A1 FTO APOBEC3D	0.02534	0.146	3.04
GO:0005768	endosome	cellular_component	30/398	1114/22360	VPS52 SLC9A7 RFTN1 VPS28 IFNAR1 RAB22A AKT2 CXCR4 IRAK4 FGD5 TNK2 F2R LRPAP1 C1ORF210 AP4M1 CALCRL AP4E1 SNX21 STEAP2 APP MYO5A TMEM106B LGMN CYB561A3 PLEKHB2 SNX27 RAB3B VPS33A CTSS RAB29	0.02589	0.1485	1.51

GO:0006357	regulation of transcription by RNA polymerase II	biological_process	68/398	2903/22360	PSMD9 MLXIPL SBNO2 C1ORF61 ZNF7 ZNF570 TCF4 MED22 ZNF264 TP53 ZNF8 RIPPLY3 NLRP3 ZBTB37 MXD1 BCAS3 MED17 SLC33A1 XIAP ZNF814 PCGF5 ZNF483 SOX11 NCOR1 RPRD1B ZNF441 ZNF772 ZKSCAN1 ZNF800 APP GMEB1 ZNF80 ZFPM2 YES1 ZNF445 ZNF70 ZMYM5 ZNF274 CX3CL1 ZNF669 WBP2 PKNOX1 IKZF3 POU5F1 HIF1AN ITGA3 RARG SMARCC1 FOXD2 ZNF286B CRX ZNF345 TENM2 MDM4 MNT ZNF789 N4BP2L2 ZNF583 ZNF430 KMT2D ARNT2 ZNF766 ZNF677 SUMO2 ZHX3 MLX SMAD2 CAMK1	0.02594	0.1487	1.32
GO:0005254	chloride channel activity	molecular_function	5/398	104/22360	APOL1 ANO6 TTYH1 CLCC1 GABRB3	0.02621	0.1492	2.7
GO:0031981	nuclear lumen	cellular_component	110/398	4985/22360	LETMD1 MKNK1 SMAD2 NSRP1 CASP8 SUMO2 ARNT2 KMT2D CHEK2 SPN ATXN3 ZNF789 MDM4 SLC35F6 ZNF345 USP44 RRP1 KLHL8 NMNAT1 WBP2 SMCR8 MCM3AP ZNF274 ISG20L2 INTS6 ZFPM2 CIART RPRD1B RGS12 GSK3B NCOR1 HERC4 FDPS RNF7 TRIM25 TONSL SYAP1 C2ORF49 SCAI DRG1 MYO1C MED17 MAPKAPK5 METTL21A LIG1 TP53 STYX MAP3K2 EYA3 TTI2 NUDT22 PSMD9 FTO GRK5 MMS22L MLX DIS3L SNORA13 RRP12 ADAR ZHX3 DDX51 ACOX1 MNT TENM2 TTC3 SMARCC1 RARG HIF1AN IKZF3 MSH6 POU5F1 TAF8 C19ORF66 DDI2 KRT18 OAS3 RILPL1 SON GMEB1 MAPK12 DDX54 DGKZ SNX27 RPL7L1 C9ORF78 C9ORF84 USP28 FCF1 PCGF5 MGLL CCDC36 XIAP EXOSC6 CWC25 BCAS3 MXD1 RFC2 HNRNPUL1 MED22 AKT2 TCF4 SRSF10 ELMOD1 ZC3H14 FBXL4 ZNF106 MLXIPL TSEN2 NPAT	0.02645	0.1504	1.24
GO:0003713	transcription coactivator activity	molecular_function	12/398	352/22360	MNT SOX11 WBP2 ZFPM2 PSMD9 GMEB1 NPAT KMT2D BRDT RARG SMARCC1 MED17	0.02651	0.1506	1.92
GO:0005977	glycogen metabolic process	biological_process	4/398	75/22360	PYGB AKT2 GSK3B PGM2L1	0.02665	0.1512	3
GO:0016758	transferase activity, transferring hexosyl groups	molecular_function	9/398	240/22360	KDELC2 A4GALT MTAP GSK3B B3GALT1 TMTC1 GALNT16 PYGB GALNT15	0.027	0.153	2.11
GO:0017111	nucleoside-triphosphatase activity	molecular_function	40/398	1577/22360	ARFRP1 CCL5 DNAL1 RAB3B DDX51 TBCK DHX30 TOR1AIP2 RABGAP1 DDX54 ABCC10 C9ORF84 DYNC2LI1 CCDC125 TNK2 F2R RGS12 ABCC3 GSK3B MSH6 ATP6V1A ABCC9 RFC2 METTL21A RAB29 TP53 ABCB5 MYO5A MYO1C DRG1 BCAS3 WNT4 FGD5 RAB22A CX3CL1 AKT2 ATP2B1 RGS17 ELMOD1 RAB40B	0.02754	0.1559	1.43
GO:2000147	positive regulation of cell motility	biological_process	18/398	597/22360	MYO1C ANO6 BCAS3 ITGA3 PGF DUOX1 CCR6 PIK3R3 APP LGMN CCL5 SPN ARHGEF39 AKT2 P2RX4 F2R EMC10 GAS6	0.02768	0.1565	1.69
GO:0010604	positive regulation of macromolecule metabolic process	biological_process	82/398	3600/22360	SPN DCUN1D2 ATXN3 TAOK1 CHEK2 ARNT2 KMT2D CRX TNK2 ZNF345 F2R SRCIN1 MNT MLX SMAD2 FTO CAMK1 LGMN SUMO2 CASP8 PRKAR2A LTBR PNPT1 TAF8 TRABD2B CX3CL1 OTUD4 WNT4 GMEB1 ZFPM2 YES1 ADCY2 PGF ITGA3 FOXD2 SMARCC1 RARG BRDT NMNAT1 RAB29 WBP2 IKZF3 PKNOX1 MSH6 POU5F1 GSK3B NCOR1 GCLC PCGF5 CXCR4 EMC10 GAS6 SOX11 MAP3K9 C5AR2 APP MAPK12 CCL5 RPRD1B EYA3 MAP3K2 VPS28 TCF4 EIF2AK2 AKT2 PSMD9 MLXIPL FAM161A NPAT SBNO2 C1ORF61 BCAS3 MED17 MYO1C EXOSC6 MAPKAPK5 SYAP1 XIAP PIK3R3 TP53 NLRP3 RFC2	0.02784	0.1571	1.28
GO:0035794	positive regulation of mitochondrial membrane permeability	biological_process	4/398	76/22360	CASP8 SLC35F6 TP53 GSK3B	0.028	0.1568	2.96
GO:0004713	protein tyrosine kinase activity	molecular_function	9/398	243/22360	TIE1 PEAK1 TNK2 APP CCL5 EIF2AK2 GAS6 YES1 SRCIN1	0.02911	0.1628	2.08

GO:1903506	regulation of nucleic acid-templated transcription	biological_process	88/398	3905/22360	C1ORF61 ZNF7 MLXIPL PSMD9 NPAT SBNO2 TCF4 EIF2AK2 ZNF264 MED22 MAP3K2 ZNF570 SRSF10 OR7D2 NLRP3 ZBTB37 MXD1 RIPPLY3 TP53 ZNF8 XIAP ZNF814 SCAI TONSL BCAS3 SLC33A1 MED17 GAS6 ZNF483 SOX11 PCGF5 IRAK4 TRIM25 GCLC NCOR1 RPRD1B APP RCAN1 ZNF441 ZNF772 DDX54 ZNF800 ZKSCAN1 CIART YES1 ZNF445 ZNF70 WNT4 GMEB1 ZNF80 ZFPM2 TAF8 CX3CL1 ZNF669 ZMYM5 ZNF274 WBP2 IKZF3 PKNOX1 POU5F1 SMARCC1 FOXD2 RARG ZNF286B BRDT HIF1AN ITGA3 MDM4 MNT CRX ZNF345 ZNF573 TENM2 F2R ZNF583 CHEK2 ARNT2 ZNF430 KMT2D ZNF789 N4BP2L2 ZHX3 ZNF766 ZNF677 SUMO2 SMAD2 CAMK1 MLX	0.02916	0.1629	1.27
GO:0044389	ubiquitin-like protein ligase binding	molecular_function	12/398	357/22360	LTBR GSK3B ATXN3 CHEK2 SMAD2 TP53 SUMO2 CXCR4 CASP8 TNK2 PRKAR2A POU5F1	0.02935	0.1638	1.89
GO:0001570	vasculogenesis	biological_process	4/398	77/22360	ZFPM2 TIE1 GJC1 HIF1AN	0.0294	0.1639	2.92
GO:0005905	clathrin-coated pit	cellular_component	4/398	77/22360	APP AAK1 EPN1 TNK2	0.0294	0.1639	2.92
GO:0046483	heterocycle metabolic process	biological_process	147/398	6887/22360	WBP2 NMNAT1 APOBEC3D CCR6 RRP1 SLC26A2 ZNF445 ISG20L2 YES1 INTS6 ZNF70 ADCY2 ZFPM2 ZNF669 OTUD4 ZNF274 TRMT10B ZNF677 SUMO2 SULT1B1 COX5B IBA57 SAMD4B SMAD2 DCTD NSRP1 MDM4 ZNF573 ZNF345 CHEK2 ARNT2 ZNF430 KMT2D TAOK1 N4BP2L2 ATXN3 ZNF789 ZBTB37 LIG1 PDE12 TP53 C2ORF49 SCAI TONSL GPAT4 MAPKAPK5 DRG1 MED17 ZNF7 C1ORF61 TMTC1 PSMD9 SBNO2 PGM2L1 SLFN13 MTAP ZNF264 MAP3K2 EYA3 ZNF570 NAGK CCL5 RPRD1B ACOT8 APP ZNF772 RCAN1 ZNF441 CIART PDP2 SOX11 ZNF483 TRIM25 QPRT GCLC ACSL6 NCOR1 POU5F1 IKZF3 MSH6 PKNOX1 SMARCC1 FOXD2 RARG ZNF286B BRDT ITGA3 HIF1AN OAS3 GMEB1 WNT4 SON ZNF80 PDE4C TAF8 CX3CL1 ZMYM5 PNPT1 ADAR ZHX3 DIS3L SNORA13 ZNF766 DDX51 MMS22L FTO CAMK1 MLX MNT CRX TENM2 F2R ZNF583 OR7D2 NLRP3 RFC2 MXD1 RIPPLY3 ZNF8 ZNF814 XIAP CCDC36 CWC25 BCAS3 EXOSC6 SLC33A1 ZC3H14 NPAT MLXIPL TSEN2 EIF2AK2 TCF4 HNRNPUL1 MED22 SRSF10 RPL7L1 MTFMT DDX54 ZNF800 ZKSCAN1 GAS6 C9ORF84 PCGF5 IRAK4 USP28 FCF1 C9ORF78	0.0298	0.1649	1.2
GO:0042802	identical protein binding	molecular_function	47/398	1915/22360	PEAK1 DDI2 LTBR GABRB3 ASPA TCF4 P2RX4 DPP9 EIF2AK2 MLXIPL FAM161A ICOSLG DRG1 SLC39A13 HIF1AN PGF XIAP SYAP1 SYP MYO5A TP53 MSH6 IKZF3 NLRP3 NMNAT1 CD3G PTPRM SLC9A7 ACSL6 CHEK2 TNK2 TENM2 MGLL QPRT DESI1 ANO6 GNPTG MAP3K9 MLX SMAD2 DCTD APP CASP8 ADD2 RPRD1B CCL5 ZHX3	0.02997	0.1657	1.38
GO:0019897	extrinsic component of plasma membrane	cellular_component	7/398	173/22360	CNR2 FARP1 SYAP1 YES1 STAC2 ESYT3 AAK1	0.02999	0.1656	2.27
GO:0016462	pyrophosphatase activity	molecular_function	41/398	1635/22360	MYO5A ABCB5 BCAS3 DRG1 MYO1C METTL21A RAB29 MSH6 ATP6V1A RFC2 ABCC9 TP53 ATP2B1 CX3CL1 RAB22A AKT2 ELMOD1 RAB40B RGS17 NUDT22 WNT4 FGD5 RABGAP1 DDX54 DNAL1 CCL5 ARFRP1 TBCK DDX51 DHX30 TOR1AIP2 RAB3B RGS12 ABCC3 GSK3B ABCC10 C9ORF84 CCDC125 DYNC2LI1 TNK2 F2R	0.0305	0.161	1.41
GO:0031325	positive regulation of cellular metabolic process	biological_process	81/398	3568/22360	GCLC GSK3B GAS6 SOX11 PCGF5 CXCR4 EMC10 C5AR2 APP MAPK12 MAP3K9 CCL5 DGKZ RPRD1B EIF2AK2 P2RX4 TCF4 AKT2 EYA3 MAP3K2 C1ORF61 FAM161A NPAT PSMD9 MLXIPL SBNO2 PIK3R3 SYAP1 XIAP BCAS3 EXOSC6 MAPKAPK5 MED17 NLRP3 RFC2 TP53 CHEK2 ARNT2 KMT2D TAOK1 SPN DCUN1D2 ATXN3 SRCIN1 MNT CRX F2R TNK2 ZNF345 FTO CAMK1 SMAD2 MLX PRKAR2A SUMO2 LGMN CASP8 TAF8 OTUD4 TRABD2B CX3CL1 LTBR PNPT1 YES1 ADCY2 GMEB1 WNT4 ZFPM2 FOXD2 SMARCC1 RARG BRDT PGF RAB29 WBP2 NMNAT1 POU5F1 IKZF3 PKNOX1 MSH6 SMCR8	0.03081	0.1615	1.28
GO:1902108	regulation of mitochondrial membrane permeability involved	biological_process	4/398	78/22360	TP53 GSK3B CASP8 SLC35F6	0.03084	0.1615	2.88

	in apoptotic process							
GO:0071260	cellular response to mechanical stimulus	biological_process	4/398	78/22360	GCLC LTBR MAP3K2 CASP8	0.03084	0.1615	2.88
GO:0072657	protein localization to membrane	biological_process	20/398	690/22360	RILPL1 AP4M1 GAS6 STAC2 BBS1 AKT2 SEC62 RFTN1 KRT18 ARFRP1 RAB29 RAB3B CASP8 TP53 PIGR MYO5A MYO1C CACNB2 ZDHHC15 ITGA3	0.03122	0.1632	1.63
GO:0040012	regulation of locomotion	biological_process	31/398	1178/22360	CCDC125 GAS6 SRCIN1 EMC10 F2R TRIM25 CXCR4 PTPRM SPN CCL5 CCR4 LGMN TBCCD1 DUOX1 APP C5AR2 ANO6 TIE1 WNT4 AKT2 CX3CL1 P2RX4 ARHGEF39 SRGAP2B SCAI CCR6 PIK3R3 MYO1C PGF ITGA3 BCAS3	0.03168	0.1651	1.48
GO:0031146	SCF-dependent proteasomal ubiquitin-dependent protein catabolic process	biological_process	5/398	109/22360	STYX FBXL4 FBXL18 PSMD9 RNF7	0.03203	0.1667	2.58
GO:0043648	dicarboxylic acid metabolic process	biological_process	5/398	109/22360	GCLC NMNAT1 QPRT ASPA ACOT8	0.03203	0.1667	2.58
GO:0048247	lymphocyte chemotaxis	biological_process	4/398	79/22360	SLC12A2 GAS6 CX3CL1 CCL5	0.03233	0.168	2.84
GO:1905710	positive regulation of membrane permeability	biological_process	4/398	79/22360	CASP8 SLC35F6 TP53 GSK3B	0.03233	0.168	2.84
GO:0043242	negative regulation of protein complex disassembly	biological_process	4/398	79/22360	TAOK1 ADD2 TMOD3 SPTAN1	0.03233	0.168	2.84
GO:0006687	glycosphingolipid metabolic process	biological_process	4/398	79/22360	B3GALT1 A4GALT ESYT3 SUMF2	0.03233	0.168	2.84
GO:0048193	Golgi vesicle transport	biological_process	13/398	402/22360	MYO5A WHAMM STEAP2 KRT18 CORO7 VPS52 SPTAN1 RAB29 COG1 GAS6 AP4M1 ARFRP1 BBS1	0.03263	0.1688	1.82
GO:0015031	protein transport	biological_process	54/398	2261/22360	SEC62 ACOX1 RFTN1 GSK3B CYP51A1 F2R DYNC2LI1 TRAF3IP1 GAS6 SRCIN1 STX16 CORO7 SYNRG CAMK1 SMAD2 RABGAP1 DENND2A C5AR2 SNX21 RAB3B TBCK SNX27 ARFRP1 ACOT8 COG1 CCL5 VPS33A VPS28 UEVLD KRT18 VPS52 RAB40B AKT2 STYX RAB22A CX3CL1 BBS1 PSMD9 AP4M1 RILPL1 MYO1C AP4E1 CYB5R4 BCAS3 ZDHHC15 CALCRL MYO5A BTN3A2 TP53 MCM3AP ATP6V1A RAB29 CD3G NLRP3	0.03294	0.1703	1.34
GO:0016787	hydrolase activity	molecular_function	92/398	4128/22360	PPM1K NLRP3 METTL21A ABCC9 PDE12 RFC2 ATP6V1A TP53 XIAP ABCB5 BCAS3 EXOSC6 MYO1C DRG1 NUDT22 FGD5 TSEN2 ATP2B1 EIF2AK2 VNN2 SLFN13 AKT2 STYX ASPA EYA3 RAB40B ELMOD1 CCL5 DNAL1 ARFRP1 ACOT8 NT5DC3 PLPP2 APP MAPK12 DDX54 RCAN1 ITIH5 PDP2 DESI1 GAS6 DYNC2LI1 CCDC125 ABCC10 C9ORF84 MGLL H6PD USP28 RGS12 GSK3B CTSS RAB29 GNS MSH6 PPP1R37 APOBEC3D MYO5A ISG20L2 PCSK7 PIGL OAS3 WNT4 PDE4C DPP9 OTUD4 RAB22A TRABD2B CX3CL1 DDI2 PNPT1 RGS17 ADAR PRKAR2A DIS3L TOR1AIP2 DHX30 DDX51 TBCK LGMN CASP8 RAB3B DCTD RABGAP1 PAPLN F2R USP44 TNK2 FARP1 ABCC3 HP ATXN3 PTPRM	0.03322	0.1715	1.25
GO:0032182	ubiquitin-like protein binding	molecular_function	5/398	110/22360	DDI2 DCUN1D2 CXCR4 UBXN2A VPS28	0.03329	0.1717	2.55
GO:0006511	ubiquitin-dependent protein catabolic process	biological_process	20/398	695/22360	PSMD9 TRIM25 FBXL4 UBXN2A USP28 TTC3 USP44 RNF7 ATXN3 GSK3B VPS28 GCLC UBE3C STYX SUMO2 RNF115 FBXL18 KLHL8 SMARCC1 TRIM72	0.03344	0.1698	1.62
GO:0003700	DNA-binding transcription factor activity	molecular_function	54/398	2265/22360	TONSL ZNF814 TP53 MXD1 ZBTB37 NLRP3 ZNF570 ZNF264 EIF2AK2 TCF4 MLXIPL ZNF7 ZNF800 ZKSCAN1 ZNF772 RCAN1 ZNF441 APP NCOR1 TRIM25 PCGF5 IRAK4 SOX11 ZNF483 GAS6 ZNF286B FOXD2 SMARCC1 RARG POU5F1 IKZF3 PKNOX1 ZNF274 ZMYM5 ZNF669 CX3CL1 ZFPM2 ZNF80 GMEB1 ZNF70 ZNF445 MLX SMAD2 ZNF677 ZNF766 ZHX3 ZNF789 ARNT2 KMT2D ZNF430 ZNF583 ZNF345 CRX MNT	0.03391	0.172	1.34

GO:0043270	positive regulation of ion transport	biological_process	10/398	287/22360	ANO6 CACNB2 P2RX4 CX3CL1 F2R RAB3B STAC2 CNKSR3 CCL5 CTSS	0.03405	0.1726	1.96
GO:0051272	positive regulation of cellular component movement	biological_process	18/398	611/22360	LGMN CCL5 ITGA3 PGF BCAS3 ANO6 MYO1C PIK3R3 CCR6 APP DUOX1 EMC10 F2R GAS6 ARHGEF39 SPN P2RX4 AKT2	0.03411	0.1727	1.66
GO:0035091	phosphatidylinositol binding	molecular_function	9/398	250/22360	GSDMB SNX27 PLEKHB2 PLEKHA2 ESYT3 SNX29 PXDC1 SNX21 GSDMA	0.03453	0.1746	2.02
GO:0090630	activation of GTPase activity	biological_process	5/398	111/22360	BCAS3 TBCK CCDC125 AKT2 RABGAP1	0.03458	0.1747	2.53
GO:0006366	transcription by RNA polymerase II	biological_process	70/398	3046/22360	TAF8 ZNF669 CX3CL1 ZMYM5 ZNF274 ZNF445 YES1 INTS6 ZNF70 GMEB1 ZFPM2 ZNF80 SMARCC1 FOXD2 RARG ZNF286B ITGA3 HIF1AN WBP2 POU5F1 IKZF3 PKNOX1 ZNF583 ARNT2 ZNF430 KMT2D N4BP2L2 ZNF789 MNT MDM4 CRX TENM2 ZNF345 CAMK1 SMAD2 MLX ZHX3 ZNF677 SUMO2 ZNF766 TCF4 MED22 ZNF264 ZNF570 ZNF7 C1ORF61 PSMD9 MLXIPL SBNO2 ZNF814 XIAP BCAS3 SLC33A1 MED17 ZBTB37 NLRP3 MXD1 RIPPLY3 TP53 ZNF8 NCOR1 SOX11 ZNF483 PCGF5 APP ZNF772 ZNF441 ZNF800 ZKSCAN1 RPRD1B	0.03469	0.1751	1.29
GO:0008092	cytoskeletal protein binding	molecular_function	27/398	1008/22360	FHDC1 FARP1 PSTPIP2 WHAMM GSK3B KLHL1 TTLL7 TAOK1 BCL7B TRAF3IP1 CXCR4 FAM161A SMAD2 RABGAP1 MYO5A SPTAN1 CACNB2 MYO1C DRG1 TMOD3 CORO7 BCAS3 RAB29 DNAL1 RAB3B LGMN ADD2	0.0355	0.1782	1.5
GO:0005770	late endosome	cellular_component	10/398	289/22360	CXCR4 CYB561A3 LGMN VPS28 F2R VPS33A IFNAR1 MYO5A TMEM106B RAB22A	0.03559	0.1784	1.94
GO:0032774	RNA biosynthetic process	biological_process	91/398	4094/22360	TP53 ZNF8 RIPPLY3 MXD1 NLRP3 OR7D2 ZBTB37 MED17 DRG1 SLC33A1 BCAS3 TONSL XIAP SCAI ZNF814 SBNO2 MLXIPL PSMD9 NPAT C1ORF61 ZNF7 ZNF570 SRSF10 MAP3K2 ZNF264 MED22 TCF4 EIF2AK2 RPRD1B CCL5 ZKSCAN1 ZNF800 CIART ZNF441 RCAN1 DDX54 ZNF772 APP IRAK4 PCGF5 TRIM25 ZNF483 SOX11 GAS6 NCOR1 GCLC PKNOX1 IKZF3 POU5F1 WBP2 HIF1AN ITGA3 BRDT ZNF286B RARG SMARCC1 FOXD2 ZNF80 ZFPM2 WNT4 GMEB1 ZNF70 INTS6 YES1 ZNF445 ZNF274 ZMYM5 CX3CL1 ZNF669 TAF8 ZNF766 ZNF677 SUMO2 ZHX3 MLX SMAD2 CAMK1 ZNF345 ZNF573 F2R TENM2 CRX MDM4 MNT ZNF789 N4BP2L2 ZNF430 KMT2D ARNT2 ZNF583 CHEK2	0.03616	0.1729	1.25
GO:0048525	negative regulation of viral process	biological_process	6/398	145/22360	CCL5 APOBEC3D TRIM25 EIF2AK2 C19ORF66 OAS3	0.03619	0.1729	2.32
GO:0019941	modification-dependent protein catabolic process	biological_process	20/398	701/22360	KLHL8 FBXL18 RNF115 SUMO2 TRIM72 SMARCC1 RNF7 FBXL4 UBXN2A USP44 TTC3 USP28 PSMD9 TRIM25 UBE3C STYX GCLC VPS28 GSK3B ATXN3	0.03627	0.1731	1.6
GO:0030335	positive regulation of cell migration	biological_process	17/398	573/22360	CCL5 LGMN PIK3R3 CCR6 APP MYO1C ANO6 ITGA3 PGF BCAS3 GAS6 EMC10 F2R AKT2 P2RX4 SPN ARHGEF39	0.03632	0.1732	1.67
GO:0006355	regulation of transcription, DNA-templated	biological_process	86/398	3846/22360	NCOR1 GCLC TRIM25 IRAK4 PCGF5 GAS6 SOX11 ZNF483 ZNF772 RCAN1 ZNF441 CIART ZKSCAN1 ZNF800 APP RPRD1B MAP3K2 SRSF10 ZNF570 EIF2AK2 TCF4 MED22 ZNF264 NPAT PSMD9 MLXIPL SBNO2 ZNF7 C1ORF61 BCAS3 MED17 SLC33A1 ZNF814 XIAP TONSL TP53 ZNF8 RIPPLY3 ZBTB37 NLRP3 OR7D2 MXD1 N4BP2L2 ZNF789 CHEK2 ZNF583 ZNF430 KMT2D ARNT2 CRX ZNF573 TENM2 F2R ZNF345 MNT MDM4 MLX CAMK1 SMAD2 ZNF677 SUMO2 ZNF766 ZHX3 ZMYM5 ZNF274 TAF8 ZNF669 CX3CL1 GMEB1 WNT4 ZFPM2 ZNF80 ZNF445 YES1 ZNF70 ITGA3 HIF1AN RARG SMARCC1 FOXD2 BRDT ZNF286B WBP2 POU5F1 PKNOX1 IKZF3	0.03645	0.1732	1.26

GO:0009893	positive regulation of metabolic process	biological_process	87/398	3899/22360	RFC2 PPM1K NLRP3 TP53 PIK3R3 SYAP1 XIAP MAPKAPK5 EXOSC6 MED17 MYO1C BCAS3 C1ORF61 SBNO2 NPAT FAM161A PSMD9 MLXIPL AKT2 EIF2AK2 P2RX4 TCF4 VPS28 EYA3 MAP3K2 RPRD1B CCL5 DGKZ MAPK12 C5AR2 APP PDP2 MAP3K9 SOX11 GAS6 EMC10 PCGF5 CXCR4 GCLC NCOR1 GSK3B POU5F1 IKZF3 MSH6 PKNOX1 RAB29 WBP2 NMNAT1 SMCR8 BRDT SMARCC1 FOXD2 RARG PGF ITGA3 ADCY2 YES1 ZFPM2 GMEB1 WNT4 OTUD4 TRABD2B CX3CL1 TAF8 PNPT1 LTBR PRKAR2A CASP8 SUMO2 LGMN FTO CAMK1 SMAD2 MLX MNT SRCIN1 F2R TNK2 ZNF345 CRX ARNT2 KMT2D CHEK2 TAOK1 SPN DCUN1D2 ATXN3	0.03708	0.176	1.25
GO:0044265	cellular macromolecule catabolic process	biological_process	33/398	1287/22360	ZC3H14 PSMD9 FBXL4 UBXN2A UBE3C SLFN13 STYX PNPT1 VPS28 NMNAT1 FBXL18 CTSS KLHL8 PDE12 SMARCC1 TRIM72 EXOSC6 RNF7 TRIM25 USP28 TTC3 USP44 GCLC CHEK2 ATXN3 GSK3B DIS3L LGMN SUMO2 RNF115 CASP8 FTO SAMD4B	0.03774	0.1786	1.44
GO:0006807	nitrogen compound metabolic process	biological_process	232/398	11357/22360	ZC3H14 MLXIPL TSEN2 P2RX4 VNN2 AKT2 MED22 GALNT15 UEVLD NLRP3 RFC2 MXD1 ZNF8 XIAP CCDC36 CWC25 EXOSC6 SLC33A1 SPTAN1 C9ORF84 PCGF5 CXCR4 SUMF2 FCF1 CYP51A1 C9ORF78 C5AR2 MAPK12 ZNF800 OAS3 GMEB1 SON CX3CL1 POU5F1 MSH6 PKNOX1 SMARCC1 FOXD2 PGF ITGA3 HIF1AN SRCIN1 MNT TTC3 TENM2 TNK2 ZNF583 HP DCUN1D2 ADAR PRKAR2A ZNF766 LGMN MMS22L GRK5 LRTOMT OGFOD1 MTAP ZNF264 MAP3K2 ZNF570 LIG1 ZBTB37 PDE12 TP53 C2ORF49 TONSL GPAT4 MAPKAPK5 APOL1 RNF7 KDELC2 SOX11 HERC4 ZNF483 EMC10 GCLC ACSL6 NCOR1 GSK3B CCL5 ACOT8 APP ZNF772 YES1 ZNF70 PCSK7 ZFPM2 OTUD4 ZNF669 TRMT10B FBXL18 WBP2 CTSS NMNAT1 KLHL8 SCYL3 SMCR8 APOBEC3D RRP1 SLC26A2 CHEK2 KMT2D TAOK1 ATXN3 PTPRM ZNF677 SUMO2 CASP8 NDUFAF7 SULT1B1 COX5B SAMD4B MKNK1 SMAD2 NSRP1 NPAT TIE1 UBXN2A FBXL4 EIF2AK2 TCF4 ASPA HNRNPUL1 RAB40B SRSF10 PPM1K OR7D2 RIPPLY3 ZNF814 TRIM72 BCAS3 ZDHHC15 CNKSR3 DESI1 GAS6 IRAK4 USP28 TTLL3 RPL7L1 PLPP2 MTFMT DDX54 ITIH5 ZKSCAN1 PIGL KDSR WNT4 ZNF80 PDE4C DPP9 TAF8 TRABD2B ZMYM5 DDI2 PNPT1 IKZF3 ESYT3 RARG ZNF286B BRDT GALNT16 TRAF3IP1 CRX F2R ZHX3 DIS3L SNORA13 TBCK DDX51 RAB3B A4GALT FTO CAMK1 CSF2RA MLX PAPLN ZNF7 C1ORF61 TMTC1 FAM161A PSMD9 SBNO2 PGM2L1 SLFN13 STYX UBE3C EYA3 VPS28 PEAK1 METTL21A SCAI PIK3R3 SYAP1 DRG1 MED17 TRIM25 B3GALT1 QPRT NAGK RPRD1B RNF115 MAP3K9 RCAN1 ZNF441 PDP2 CIART NXN ZNF445 ISG20L2 INTS6 ADCY2 LTBR ZNF274 GNS PPP1R37 CCR6 AAK1 MDM4 ZNF573 USP44 ZNF345 ARNT2 ZNF430 N4BP2L2 TTLL7 SPN ZNF789 NOA1 IBA57 DUOX1 DCTD	0.03793	0.1794	1.15
GO:1901879	regulation of protein depolymerization	biological_process	4/398	83/22360	SPTAN1 TMOD3 ADD2 TAOK1	0.03878	0.1832	2.71
GO:0072395	signal transduction involved in cell cycle checkpoint	biological_process	4/398	83/22360	CHEK2 TP53 SOX11 MDM4	0.03878	0.1832	2.71
GO:0045184	establishment of protein localization	biological_process	56/398	2382/22360	ZDHHC15 CALCRL BCAS3 MYO1C CYB5R4 TP53 NLRP3 ATP6V1A VPS52 RAB40B UEVLD VPS28 AKT2 STYX PSMD9 AP4M1 CORO7 SNX21 DENND2A C5AR2 SNX27 CCL5 ARFRP1 ACOT8 GSK3B SEC62 CYP51A1 GAS6 DYNC2LI1 AP4E1 MYO5A MCM3AP BTN3A2 WBP2 RAB29 CD3G KRT18 RAB22A CX3CL1 BBS1 RILPL1 STX16 SMAD2 RABGAP1 SYNRG CAMK1 TBCK RAB3B CASP8 COG1 VPS33A RFTN1 ACOX1 F2R SRCIN1 TRAF3IP1	0.03921	0.1849	1.32
GO:0043130	ubiquitin binding	molecular_function	4/398	84/22360	DDI2 CXCR4 VPS28 UBXN2A	0.04051	0.1876	2.68

GO:0042886	amide transport	biological_process	55/398	2340/22360	BBS1 RILPL1 KRT18 RAB22A CX3CL1 BTN3A2 MCM3AP CD3G RAB29 AP4E1 MYO5A F2R TRAF3IP1 SRCIN1 ACOX1 RFTN1 RAB3B TBCK VPS33A COG1 STX16 CAMK1 SYNRG SMAD2 RABGAP1 PSMD9 AP4M1 VPS28 UEVLD VPS52 RAB40B STYX AKT2 TP53 ATP6V1A NLRP3 MYO1C CYB5R4 SLC33A1 BCAS3 ZDHHC15 CALCRL DYNC2LI1 GAS6 SEC62 GSK3B CYP51A1 SNX27 ACOT8 ARFRP1 CCL5 CORO7 C5AR2 DENND2A SNX21	0.041	0.1897	1.32
GO:0043434	response to peptide hormone	biological_process	14/398	456/22360	PNPT1 MYO1C RABGAP1 TRIM72 AKT2 MYO5A PIK3R3 GCLC SMARCC1 SYAP1 ZNF106 ADCY2 ATP6V1A PRKAR2A	0.04126	0.1908	1.72
GO:1901135	carbohydrate derivative metabolic process	biological_process	31/398	1207/22360	ACSL6 NCOR1 KDELC2 SUMF2 H6PD B3GALT1 DCTD COX5B A4GALT PDP2 ITIH5 ACOT8 NAGK SULT1B1 MTAP PGM2L1 GALNT15 GK5 ADCY2 PIGL PDE4C TMTC1 MLXIPL SLC26A2 APOBEC3D GALNT16 GPAT4 GNS NMNAT1 SHPK ESYT3	0.04241	0.1861	1.44
GO:0006351	transcription, DNA-templated	biological_process	89/398	4026/22360	MED22 ZNF264 TCF4 EIF2AK2 ZNF570 SRSF10 MAP3K2 C1ORF61 ZNF7 SBNO2 PSMD9 MLXIPL NPAT TONSL XIAP SCAI ZNF814 MED17 DRG1 SLC33A1 BCAS3 MXD1 NLRP3 OR7D2 ZBTB37 TP53 ZNF8 RIPPLY3 GCLC NCOR1 ZNF483 SOX11 GAS6 IRAK4 PCGF5 TRIM25 APP ZKSCAN1 ZNF800 CIART RCAN1 ZNF441 ZNF772 RPRD1B CX3CL1 ZNF669 TAF8 ZNF274 ZMYM5 ZNF70 INTS6 YES1 ZNF445 ZNF80 ZFPM2 WNT4 GMEB1 BRDT ZNF286B RARG SMARCC1 FOXD2 HIF1AN ITGA3 PKNOX1 IKZF3 POU5F1 WBP2 ZNF430 KMT2D ARNT2 ZNF583 CHEK2 ZNF789 N4BP2L2 MDM4 MNT ZNF345 ZNF573 TENM2 F2R CRX SMAD2 CAMK1 MLX ZHX3 ZNF766 ZNF677 SUMO2	0.04277	0.1859	1.24
GO:0007041	lysosomal transport	biological_process	5/398	117/22360	AP4M1 TMEM106B SNX27 VPS52 VPS33A	0.04308	0.1871	2.4
GO:0032611	interleukin-1 beta production	biological_process	5/398	117/22360	MR1 CX3CL1 F2R NLRP3 APP	0.04308	0.1871	2.4
GO:0005215	transporter activity	molecular_function	40/398	1629/22360	ATP6V1A ABCC9 CTSS SLC33A1 CACNB2 SLC35E2B SLC39A13 APOL1 CALCRL SLC26A2 ABCB5 MYO5A STAC2 SLC25A26 SLC37A2 SLC35E1 GABRB3 KCNN3 P2RX4 ATP2B1 SLC12A2 KCNJ15 SLC9A4 GJC1 ANO6 TTYH1 SLC30A6 STEAP2 APP COX5B SLC35F6 CLCC1 LRRC8B ABCC10 GAS6 CNKSR3 SEC62 SLC9A7 SLC25A44 ABCC3	0.04326	0.1876	1.38
GO:0008528	G protein-coupled peptide receptor activity	molecular_function	6/398	151/22360	CCR4 CALCRL CXCR4 F2R CCR6 SSTR2	0.04368	0.1893	2.23
GO:2000145	regulation of cell motility	biological_process	28/398	1073/22360	SRCIN1 GAS6 CCDC125 WNT4 TIE1 EMC10 F2R P2RX4 CX3CL1 AKT2 SPN ARHGEF39 PTPRM SRGAP2B CCL5 LGMN TBCCD1 C5AR2 APP SCAI CCR6 PIK3R3 DUOX1 BCAS3 PGF ITGA3 MYO1C ANO6	0.04386	0.1899	1.47
GO:0015833	peptide transport	biological_process	54/398	2303/22360	CYP51A1 GSK3B SEC62 GAS6 DYNC2LI1 SNX21 DENND2A C5AR2 CORO7 CCL5 ARFRP1 ACOT8 SNX27 AKT2 STYX VPS52 RAB40B UEVLD VPS28 AP4M1 PSMD9 ZDHHC15 CALCRL BCAS3 MYO1C CYB5R4 NLRP3 ATP6V1A TP53 RFTN1 ACOX1 SRCIN1 TRAF3IP1 F2R SMAD2 RABGAP1 CAMK1 SYNRG STX16 VPS33A COG1 TBCK RAB3B RAB22A CX3CL1 KRT18 RILPL1 BBS1 MYO5A AP4E1 RAB29 CD3G MCM3AP BTN3A2	0.04438	0.1912	1.32
GO:0071156	regulation of cell cycle arrest	biological_process	5/398	118/22360	CHEK2 TP53 MLXIPL AKT2 MDM4	0.04462	0.192	2.38
GO:0098562	cytoplasmic side of membrane	cellular_component	7/398	187/22360	ESYT3 STAC2 SYAP1 YES1 ATP2B1 CNR2 FARP1	0.04466	0.1921	2.1
GO:0046902	regulation of mitochondrial membrane permeability	biological_process	4/398	87/22360	SLC35F6 CASP8 GSK3B TP53	0.04601	0.1977	2.58
GO:0048041	focal adhesion assembly	biological_process	4/398	87/22360	WNT4 WHAMM BCAS3 PEAK1	0.04601	0.1977	2.58
GO:0007601	visual perception	biological_process	8/398	225/22360	MYO5A CYP4V2 FAM161A BBS1 CRX EYA3 GJC1 CACNB2	0.04625	0.1964	2

GO:0042626	ATPase activity, coupled to transmembrane movement of substances	molecular_function	6/398	153/22360	ATP2B1 ABCC9 ABCB5 ABCC3 ATP6V1A ABCC10	0.04639	0.1969	2.2
GO:0006261	DNA-dependent DNA replication	biological_process	6/398	153/22360	RFC2 TONSL MSH6 MMS22L LIG1 CHEK2	0.04639	0.1969	2.2
GO:0006796	phosphate-containing compound metabolic process	biological_process	80/398	3594/22360	TBCK SULT1B1 ADAR PRKAR2A GRK5 COX5B CAMK1 MKNK1 CSF2RA DCTD SMAD2 F2R TNK2 TRAF3IP1 SRCIN1 TAOK1 PTPRM CHEK2 FARP1 SCYL3 SMCR8 PPP1R37 NMNAT1 AAK1 PGF SLC26A2 PLEKHA2 PDE4C YES1 ADCY2 PIGL LTBR CX3CL1 PLPP2 FITM2 NAGK CCL5 DGKZ RPRD1B NT5DC3 ACOT8 MAP3K9 GNPTG RCAN1 PDP2 C5AR2 APP NDUFA10 MAPK12 H6PD QPRT CXCR4 IRAK4 EMC10 CNKSR3 GAS6 FDPS NCOR1 GSK3B ACSL6 TP53 SHPK PPM1K PDE12 GPAT4 MAPKAPK5 SPTAN1 PIK3R3 SYAP1 PSMD9 MLXIPL TIE1 GK5 EYA3 MAP3K2 PEAK1 EIF2AK2 PGM2L1 STYX AKT2	0.04711	0.1994	1.25
GO:0019725	cellular homeostasis	biological_process	27/398	1035/22360	ATP6V1A SLC12A2 RFC2 SLC9A4 VPS33A CCL5 FITM2 CCR4 MYO5A C5AR2 APP CCR6 NXN CACNB2 SLC39A13 ANO6 GAS6 F2R MLXIPL CXCR4 LRRC8B CX3CL1 P2RX4 GCLC ATP2B1 SLC9A7 TAOK1	0.04742	0.2006	1.47
GO:0043161	proteasome-mediated ubiquitin-dependent protein catabolic process	biological_process	14/398	465/22360	FBXL18 RNF7 USP44 UBXN2A FBXL4 SUMO2 TRIM25 PSMD9 TRIM72 STYX GCLC SMARCC1 ATXN3 GSK3B	0.04777	0.2019	1.69
GO:0007034	vacuolar transport	biological_process	6/398	154/22360	VPS28 VPS52 SNX27 AP4M1 TMEM106B VPS33A	0.0478	0.2019	2.19
GO:0042737	drug catabolic process	biological_process	6/398	155/22360	HP QPRT SULT1B1 NAGK HBE1 DUOX1	0.04923	0.199	2.17
GO:0035264	multicellular organism growth	biological_process	6/398	155/22360	GPAT4 RABGAP1 LGMN APP RARG FTO	0.04923	0.199	2.17
GO:0044773	mitotic DNA damage checkpoint	biological_process	5/398	121/22360	TAOK1 TP53 CHEK2 MDM4 MSH6	0.04947	0.1992	2.32
GO:0032652	regulation of interleukin-1 production	biological_process	5/398	121/22360	CX3CL1 F2R NLRP3 GAS6 APP	0.04947	0.1992	2.32
GO:0001676	long-chain fatty acid metabolic process	biological_process	5/398	121/22360	ACSL6 MGLL MYO5A ACOT8 ACOX1	0.04947	0.1992	2.32
GO:0006091	generation of precursor metabolites and energy	biological_process	16/398	552/22360	PGM2L1 AKT2 GSK3B NCOR1 ACOX1 PNPT1 MLXIPL H6PD PYGB CS NDUFA10 COX5B CYB5R4 NOA1 PDE12 SHPK	0.04976	0.1986	1.63
GO:0006112	energy reserve metabolic process	biological_process	4/398	89/22360	AKT2 PYGB PGM2L1 GSK3B	0.04994	0.1992	2.52
GO:0031397	negative regulation of protein ubiquitination	biological_process	4/398	89/22360	NXN USP44 VPS28 GCLC	0.04994	0.1992	2.52
GO:0015075	ion transmembrane transporter activity	molecular_function	30/398	1178/22360	SLC37A2 STAC2 ATP2B1 P2RX4 KCNN3 GABRB3 CTSS ABCC9 ATP6V1A MYO5A SLC26A2 APOL1 SLC39A13 CACNB2 SLC33A1 CNKSR3 GAS6 LRRC8B CLCC1 ABCC3 SLC9A7 SLC9A4 KCNJ15 SLC12A2 COX5B APP SLC30A6 TTYH1 ANO6 GJC1	0.05005	0.1993	1.43
GO:0034220	ion transmembrane transport	biological_process	34/398	1364/22360	CTSS ABCC9 ATP6V1A MYO5A SLC26A2 APOL1 SLC39A13 CACNB2 SLC33A1 SLC37A2 STAC2 P2RX4 ATP2B1 AKT2 KCNN3 CX3CL1 GABRB3 SLC9A4 SLC12A2 KCNJ15 APP COX5B STEAP2 SLC30A6 TTYH1 ANO6 GJC1 CNKSR3 GAS6 LRRC8B F2R CLCC1 ABCC3 SLC9A7	0.05007	0.1992	1.4
GO:0050953	sensory perception of light stimulus	biological_process	8/398	229/22360	CYP4V2 MYO5A CACNB2 GJC1 BBS1 CRX EYA3 FAM161A	0.05085	0.2022	1.96
GO:0072089	stem cell proliferation	biological_process	5/398	122/22360	ACSL6 N4BP2L2 EIF2AK2 CX3CL1 SOX11	0.05116	0.2033	2.3

GO:0031090	organelle membrane	cellular_component	88/398	4012/22360	P2RX4 CARD19 RAB40B VPS52 GALNT15 VPS28 AP4M1 SLC25A26 SCAI CYP4V2 TRIM72 ZDHHC15 GPAT4 CYB5R4 SLC33A1 MYO1C NLRP3 ATP6V1A SYT12 ACSL6 CYP51A1 SLC9A7 SLC25A44 SEC62 ABCC10 B3GALT1 MGLL IRAK4 TMEM170A EMC10 SNX21 NDUFA10 APP GNPTG CORO7 COQ6 ARFRP1 SNX27 PLEKHB2 PIGR FITM2 WHAMM TRABD2B RAB22A GABRB3 PNPT1 SLC37A2 PIGL PCSK7 KDSR WNT4 BBS1 SYP AP4E1 GALNT16 SLC39A13 CD3G MCM3AP COX6B2 ESYT3 ABCC3 ATXN3 ACOX1 LRRC8B TNK2 SLC35F6 STEAP2 A4GALT GRK5 COX5B LETMD1 MR1 TTYH1 SLC30A6 TMEM106B SYNRG MLX STX16 GJC1 ANO6 RRP12 NOA1 COG1 VPS33A CYB561A3 TOR1AIP2 RAB3B CASP8	0.05147	0.2043	1.23
GO:0002548	monocyte chemotaxis	biological_process	4/398	90/22360	CX3CL1 ANO6 LGMN CCL5	0.05197	0.2057	2.5
GO:0033036	macromolecule localization	biological_process	77/398	3463/22360	AKT2 STYX P2RX4 VPS28 UEVLD RAB40B VPS52 AP4M1 XKR4 PSMD9 TONSL APOL1 CYB5R4 CACNB2 MYO1C BCAS3 CALCRL ZDHHC15 ATP6V1A NLRP3 TP53 CEP112 CYP51A1 SEC62 NCOR1 GSK3B DYNC2LI1 GAS6 DENND2A C5AR2 SNX21 CORO7 ARFRP1 ACOT8 CCL5 FITM2 PIGR SNX27 CX3CL1 RAB22A TAF8 PNPT1 KRT18 CC2D2A RILPL1 STAC2 BBS1 MYO5A AP4E1 AAK1 ITGA3 RAB29 CD3G WBP2 ESYT3 BTN3A2 MCM3AP ABCC3 ACOX1 RFTN1 ATXN3 TRAF3IP1 SRCIN1 F2R FTO SYNRG CAMK1 RABGAP1 SMAD2 ANO6 STX16 COG1 VPS33A CASP8 RAB3B TOR1AIP2 TBCK	0.05216	0.2063	1.25
GO:0071310	cellular response to organic substance	biological_process	69/398	3067/22360	DDX54 APP SSTR2 MAPK12 CCR4 CCL5 SLC12A2 RABGAP1 NCOR1 GSK3B GCLC TRIM25 CXCR4 IRAK4 GAS6 SOX11 CALCRL MYO1C SLC33A1 PIK3R3 XIAP SYAP1 TRIM72 TP53 ZNF8 SHPK NLRP3 PDE12 ATP6V1A P2RX4 ATP2B1 EIF2AK2 MTAP AKT2 PSMD9 MLXIPL UBXN2A ZNF106 SBNO2 DUOX1 SMAD2 CSF2RA LGMN CASP8 ADAR PRKAR2A ATXN3 CHEK2 KMT2D PGF ITGA3 CCR6 MYO5A RARG SYP SMARCC1 WBP2 CTSS LTBR KRT18 GABRB3 PNPT1 IFNAR1 OTUD4 CX3CL1 WNT4 YES1 ADCY2 OAS3	0.05282	0.2087	1.26
GO:0031929	TOR signaling	biological_process	5/398	123/22360	MAPKAPK5 SMCR8 SYAP1 TBCK GAS6	0.05289	0.2089	2.28
GO:0030031	cell projection assembly	biological_process	18/398	643/22360	BCAS3 ANO6 RABGAP1 TTYH1 DNAL1 CC2D2A ODF2L TTLL3 WHAMM P2RX4 FHDC1 BBS1 FGD5 FAM161A TENM2 TRAF3IP1 RILPL1 DYNC2LI1	0.05314	0.2097	1.57
GO:0051224	negative regulation of protein transport	biological_process	8/398	231/22360	F2R PSMD9 CYP51A1 CX3CL1 C5AR2 SRCIN1 NLRP3 GAS6	0.05327	0.21	1.95
GO:0032874	positive regulation of stress-activated MAPK cascade	biological_process	7/398	194/22360	TAOK1 LTBR MAP3K2 MAP3K9 EMC10 EIF2AK2 APP	0.05362	0.2098	2.03
GO:0032535	regulation of cellular component size	biological_process	12/398	390/22360	LRRC8B ADD2 RAB3B SLC12A2 GSK3B ANO6 TMOD3 MYO1C SPTAN1 WHAMM RARG RAB22A	0.0542	0.2119	1.73
GO:0002064	epithelial cell development	biological_process	8/398	232/22360	GPAT4 GSK3B MARVELD2 SLC9A4 RARG LRTOMT VSIG1 RILPL1	0.05451	0.213	1.94
GO:0051427	hormone receptor binding	molecular_function	7/398	195/22360	CRX DDX54 NCOR1 BCAS3 MED17 RARG WBP2	0.05499	0.2142	2.02
GO:0070304	positive regulation of stress-activated protein kinase signaling cascade	biological_process	7/398	195/22360	EIF2AK2 APP TAOK1 LTBR MAP3K2 MAP3K9 EMC10	0.05499	0.2142	2.02
GO:0019058	viral life cycle	biological_process	12/398	391/22360	CXCR4 TRIM25 GAS6 ADAR CCL5 RAB29 OAS3 PDE12 VPS28 EIF2AK2 C19ORF66 APOBEC3D	0.05514	0.2145	1.72
GO:0030509	BMP signaling pathway	biological_process	6/398	159/22360	SLC33A1 ZNF8 ITGA3 SMAD2 SOX11 XIAP	0.05524	0.2047	2.12
GO:0016810	hydrolase activity, acting on carbon-nitrogen (but not peptide) bonds	molecular_function	6/398	159/22360	ASPA PIGL DCTD ADAR APOBEC3D VNN2	0.05524	0.2047	2.12
GO:0005902	microvillus	cellular_component	4/398	92/22360	SLC26A2 MYO1C ATP6V1A SPN	0.05621	0.208	2.44

GO:0030041	actin filament polymerization	biological_process	7/398	196/22360	CORO7 ADD2 TMOD3 MYO1C SPTAN1 WHAMM PSTPIP2	0.05639	0.2085	2.01
GO:0006664	glycolipid metabolic process	biological_process	5/398	125/22360	A4GALT B3GALT1 SUMF2 PIGL ESYT3	0.05646	0.2086	2.25
GO:0016999	antibiotic metabolic process	biological_process	5/398	125/22360	DUOX1 SULT1B1 NAGK HP HBE1	0.05646	0.2086	2.25
GO:0070997	neuron death	biological_process	13/398	434/22360	NMNAT1 CCL5 RAB29 RILPL1 LGMN CASP8 F2R GCLC APP CX3CL1 AKT2 GSK3B GABRB3	0.05667	0.2091	1.68
GO:0008152	metabolic process	biological_process	267/398	13341/22360	CNR2 MDM4 CS ZNF345 ZNF573 USP44 ARNT2 ZNF430 SPN ZNF789 N4BP2L2 TTLL7 NOA1 CRYZL1 STEAP2 IBA57 DCTD DUOX1 ISG20L2 INTS6 ZNF445 ADCY2 LTBR ZNF274 GNS PPP1R37 CCR6 AAK1 FDPS QPRT B3GALT1 TRIM25 NAGK RPRD1B RNF115 MAP3K9 ZNF441 RCAN1 NXN CIART PDP2 C1ORF61 ZNF7 PSMD9 FAM161A TMTC1 SBNO2 PGM2L1 UBE3C STYX SLFN13 EYA3 PEAK1 VPS28 METTL21A SYAP1 PIK3R3 SCAI CYP4V2 DRG1 MYO1C MED17 TRAF3IP1 CRX F2R FARP1 VPS33A ZHX3 DIS3L SNORA13 DDX51 TBCK RAB3B A4GALT CSF2RA FTO CAMK1 PAPLN MLX PIGL WNT4 KDSR PLEKHA2 ZNF80 PDE4C TAF8 DPP9 TRABD2B DDI2 ZMYM5 PNPT1 IKZF3 ESYT3 RARG ZNF286B BRDT GALNT16 GAS6 DESI1 CNKSR3 H6PD IRAK4 MGLL USP28 TTLL3 COQ6 NT5DC3 PLPP2 RPL7L1 MTFMT ITIH5 DDX54 ZKSCAN1 GK5 NPAT MIR654 FBXL4 TIE1 UBXN2A TCF4 EIF2AK2 HNRNPUL1 ASPA RAB40B SRSF10 OR7D2 PPM1K RIPPLY3 ZNF814 TRIM72 ZDHHC15 BCAS3 CHEK2 KMT2D ATXN3 TAOK1 PTPRM ZNF677 SUMO2 NDUFAF7 SULT1B1 CASP8 COX5B MKNK1 SMAD2 SAMD4B NSRP1 YES1 LRPAP1 ZNF70 PCSK7 ZFPM2 ZNF669 OTUD4 TRMT10B NMNAT1 FBXL18 CTSS WBP2 KLHL8 SCYL3 MCM3AP SMCR8 MYO5A APOBEC3D SLC26A2 RRP1 RNF7 KDELC2 HERC4 ZNF483 SOX11 EMC10 ACSL6 GCLC GSK3B NCOR1 CCL5 ACOT8 NDUFA10 APP ZNF772 LRTOMT OGFOD1 ZNF264 MTAP MAP3K2 ZNF570 ZBTB37 LIG1 ATP6V1A PDE12 TP53 C2ORF49 TONSL CALCRL GPAT4 MAPKAPK5 APOL1 SRCIN1 MNT TNK2 TENM2 TTC3 ZNF583 HBE1 DCUN1D2 HP ACOX1 PRKAR2A ADAR ZNF766 CYB561A3 LGMN GRK5 MMS22L OAS3 GMEB1 SON CX3CL1 RAB29 MSH6 PKNOX1 POU5F1 SMARCC1 FOXD2 AS3MT HIF1AN ITGA3 PGF C9ORF84 PCGF5 CXCR4 SUMF2 FCF1 CYP51A1 KIAA1191 C9ORF78 DGKZ FITM2 C5AR2 MAPK12 GNPTG ZNF800 ZC3H14 MLXIPL TSEN2 PYGB VNN2 P2RX4 MED22 AKT2 NUBPL UEVLD GALNT15 NLRP3 MXD1 RFC2 ZNF8 SHPK XIAP CCDC36 CWC25 SLC33A1 CYB5R4 SPTAN1 EXOSC6	0.05714	0.2107	1.12
GO:0007265	Ras protein signal transduction	biological_process	14/398	477/22360	CCDC125 RAB29 USP28 F2R RAB3B FGD5 TP53 FARP1 RAB22A SCAI MAPKAPK5 ARHGEF39 ITGA3 RAB40B	0.0576	0.2104	1.65
GO:0030334	regulation of cell migration	biological_process	26/398	1009/22360	SRCIN1 GAS6 WNT4 EMC10 F2R TIE1 P2RX4 AKT2 CX3CL1 ARHGEF39 SPN PTPRM CCL5 SRGAP2B LGMN TBCCD1 C5AR2 PIK3R3 CCR6 APP SCAI BCAS3 ITGA3 PGF ANO6 MYO1C	0.05806	0.2119	1.45
GO:0042552	myelination	biological_process	5/398	126/22360	ASPA AKT2 CXCR4 RARG MYO5A	0.0583	0.2127	2.23
GO:1901184	regulation of ERBB signaling pathway	biological_process	4/398	93/22360	APP LGMN EPN1 RNF115	0.05841	0.2129	2.42
GO:0030510	regulation of BMP signaling pathway	biological_process	4/398	93/22360	ITGA3 XIAP SOX11 SMAD2	0.05841	0.2129	2.42
GO:0030165	PDZ domain binding	molecular_function	4/398	93/22360	ATP2B1 SSTR2 PLEKHA2 ACOX1	0.05841	0.2129	2.42
GO:0050918	positive chemotaxis	biological_process	4/398	93/22360	CX3CL1 PGF CCL5 CCR4	0.05841	0.2129	2.42
GO:0043524	negative regulation of neuron apoptotic process	biological_process	6/398	161/22360	LGMN GABRB3 F2R NMNAT1 GCLC CX3CL1	0.05842	0.2124	2.09
GO:0051186	cofactor metabolic process	biological_process	14/398	478/22360	PDP2 GPAT4 HP DUOX1 IBA57 ACSL6 VNN2 HBE1 GCLC QPRT CRYZL1 ACOT8 COQ6	0.05849	0.2125	1.65

					NMNAT1			
GO:0045893	positive regulation of transcription, DNA-templated	biological_process	39/398	1622/22360	TP53 PKNOX1 IKZF3 POU5F1 NLRP3 WBP2 MED17 BCAS3 BRDT RARG SMARCC1 FOXD2 SBNO2 ZFPM2 WNT4 MLXIPL PSMD9 GMEB1 NPAT C1ORF61 YES1 MAP3K2 CX3CL1 TCF4 TAF8 SUMO2 RPRD1B MLX SMAD2 CAMK1 APP ZNF345 F2R PCGF5 CRX SOX11 KMT2D ARNT2 CHEK2	0.06	0.2178	1.35
GO:1901981	phosphatidylinositol phosphate binding	molecular_function	6/398	162/22360	GSDMB SNX27 PLEKHB2 PLEKHA2 SNX21 GSDMA	0.06006	0.2179	2.08
GO:0001653	peptide receptor activity	molecular_function	6/398	162/22360	CCR6 SSTR2 CXCR4 CALCRL CCR4 F2R	0.06006	0.2179	2.08
GO:0045778	positive regulation of ossification	biological_process	4/398	94/22360	ZHX3 WNT4 SOX11 ANO6	0.06066	0.2194	2.39
GO:0014015	positive regulation of gliogenesis	biological_process	4/398	94/22360	APP P2RX4 CXCR4 ASPA	0.06066	0.2194	2.39
GO:0051453	regulation of intracellular pH	biological_process	4/398	94/22360	SLC9A4 VPS33A SLC9A7 ATP6V1A	0.06066	0.2194	2.39
GO:0008366	axon ensheathment	biological_process	5/398	128/22360	AKT2 ASPA MYO5A CXCR4 RARG	0.06211	0.2239	2.19
GO:0005543	phospholipid binding	molecular_function	13/398	440/22360	SNX29 PXDC1 GSDMA TRIM72 GRK5 SNX21 ESYT3 PLEKHA2 PLEKHB2 GSDMB SNX27 SYT12 GAS6	0.06233	0.2245	1.66
GO:0042327	positive regulation of phosphorylation	biological_process	29/398	1157/22360	NMNAT1 TP53 PIK3R3 SYAP1 PGF MAPKAPK5 YES1 ADCY2 MLXIPL EIF2AK2 AKT2 CX3CL1 LTBR MAP3K2 CCL5 DGKZ PRKAR2A APP C5AR2 CAMK1 MAP3K9 GAS6 SRCIN1 CXCR4 F2R EMC10 TNK2 CHEK2 TAOK1	0.06362	0.2212	1.41
GO:0017137	Rab GTPase binding	molecular_function	7/398	201/22360	SMCR8 VPS52 TBCK RABGAP1 MYO5A DENND2A RAB29	0.06376	0.2215	1.96
GO:0009058	biosynthetic process	biological_process	143/398	6876/22360	MGLL IRAK4 PCGF5 GAS6 CYP51A1 PLPP2 MTFMT FITM2 COQ6 DGKZ DDX54 ZKSCAN1 ZNF800 MLXIPL NPAT GK5 GALNT15 SRSF10 TCF4 P2RX4 EIF2AK2 MED22 ASPA AKT2 ZNF8 RIPPLY3 NLRP3 OR7D2 MXD1 RFC2 ZDHHC15 BCAS3 SLC33A1 XIAP ZNF814 CRX TENM2 F2R MNT ZNF583 ZNF766 ZHX3 MLX A4GALT MMS22L CAMK1 FTO KDSR WNT4 GMEB1 PDE4C ZNF80 PLEKHA2 PIGL ZMYM5 TAF8 CX3CL1 PKNOX1 MSH6 IKZF3 POU5F1 HIF1AN ITGA3 GALNT16 RARG FOXD2 SMARCC1 BRDT ZNF286B B3GALT1 QPRT TRIM25 KDELC2 ZNF483 FDPS SOX11 GSK3B NCOR1 ACSL6 GCLC CCL5 NAGK ACOT8 RPRD1B RCAN1 ZNF441 ZNF772 PDP2 CIART APP PSMD9 TMTC1 SBNO2 OGFOD1 C1ORF61 ZNF7 MAP3K2 ZNF570 PGM2L1 ZNF264 MTAP TP53 LIG1 ZBTB37 GPAT4 MED17 DRG1 MAPKAPK5 SCAI PIK3R3 TONSL ZNF345 ZNF573 MDM4 ZNF789 SPN N4BP2L2 CHEK2 KMT2D ZNF430 ARNT2 SUMO2 ZNF677 NOA1 IBA57 COX5B DCTD SMAD2 MKNK1 DUOX1 SAMD4B ZFPM2 INTS6 YES1 ZNF445 ADCY2 ZNF70 ZNF274 ZNF669 NMNAT1 WBP2 MYO5A SLC26A2	0.06387	0.2217	1.17
GO:0043087	regulation of GTPase activity	biological_process	15/398	527/22360	TNK2 F2R TBCK WNT4 FGD5 CCDC125 CCL5 RGS17 ELMOD1 GSK3B BCAS3 CX3CL1 RABGAP1 RGS12 AKT2	0.06419	0.2227	1.6
GO:0016757	transferase activity, transferring glycosyl groups	molecular_function	10/398	319/22360	KDELC2 PYGB TMTC1 QPRT B3GALT1 MTAP A4GALT GALNT15 GALNT16 GSK3B	0.06514	0.2258	1.76
GO:0042803	protein homodimerization activity	molecular_function	24/398	930/22360	SLC9A7 ACSL6 TCF4 CHEK2 P2RX4 QPRT MLXIPL MGLL TENM2 MAP3K9 HIF1AN GNPTG MLX PGF SLC39A13 ANO6 SYAP1 SMAD2 ADD2 CD3G CCL5 ZHX3 IKZF3 MSH6	0.06603	0.2266	1.45
GO:0031123	RNA 3'-end processing	biological_process	6/398	166/22360	INTS6 ZC3H14 APP RPRD1B EXOSC6 PNPT1	0.06692	0.2295	2.03
GO:0008757	S-adenosylmethionine-dependent methyltransferase activity	molecular_function	6/398	166/22360	AS3MT KMT2D METTL21A LRTOMT NDUFAF7 TRMT10B	0.06692	0.2295	2.03

GO:0071702	organic substance transport	biological_process	69/398	3110/22360	SNX21 DENND2A C5AR2 CORO7 CCL5 ARFRP1 ACOT8 SNX27 CYP51A1 GSK3B NCOR1 SEC62 GAS6 DYNC2LI1 ZDHHC15 CALCRL BCAS3 MYO1C SLC33A1 CYB5R4 APOL1 SLC35E2B NLRP3 SYT12 ATP6V1A TP53 P2RX4 AKT2 STYX VPS52 RAB40B UEVLD VPS28 AP4M1 SLC25A26 PSMD9 XKR4 SMAD2 RABGAP1 SYNRG CAMK1 STX16 ANO6 COG1 VPS33A TBCK RAB3B ABCC3 RFTN1 ACOX1 SRCIN1 TRAF3IP1 F2R MYO5A SLC26A2 AP4E1 RAB29 CD3G MCM3AP BTN3A2 ESYT3 RAB22A CX3CL1 KRT18 PNPT1 YES1 SLC37A2 RILPL1 BBS1	0.06727	0.2304	1.25
GO:0044774	mitotic DNA integrity checkpoint	biological_process	5/398	131/22360	CHEK2 TP53 TAOK1 MDM4 MSH6	0.06811	0.2332	2.14
GO:0034641	cellular nitrogen compound metabolic process	biological_process	155/398	7520/22360	MLX MMS22L CAMK1 FTO DDX51 ZNF766 ZHX3 ADAR SNORA13 DIS3L ZNF583 CRX TENM2 F2R MNT HIF1AN ITGA3 RARG SMARCC1 FOXD2 BRDT ZNF286B MSH6 PKNOX1 IKZF3 POU5F1 ZMYM5 PNPT1 TAF8 CX3CL1 WNT4 GMEB1 ZNF80 PDE4C SON OAS3 DDX54 ZKSCAN1 ZNF800 RPL7L1 MTFMT C9ORF78 IRAK4 PCGF5 FCF1 USP28 GAS6 C9ORF84 CWC25 BCAS3 SLC33A1 EXOSC6 XIAP ZNF814 CCDC36 ZNF8 RIPPLY3 NLRP3 OR7D2 MXD1 RFC2 SRSF10 TCF4 VNN2 P2RX4 EIF2AK2 MED22 HNRNPUL1 AKT2 TSEN2 MLXIPL NPAT ZC3H14 NSRP1 IBA57 COX5B SMAD2 DCTD MKNK1 SAMD4B ZNF677 SUMO2 SULT1B1 NOA1 ATXN3 ZNF789 TAOK1 N4BP2L2 CHEK2 ZNF430 KMT2D ARNT2 ZNF345 ZNF573 MDM4 CCR6 APOBEC3D SLC26A2 RRP1 NMNAT1 WBP2 ZNF274 TRMT10B OTUD4 ZNF669 ZFPM2 INTS6 ISG20L2 YES1 ZNF445 PCSK7 ADCY2 ZNF70 RCAN1 ZNF441 ZNF772 PDP2 CIART APP CCL5 NAGK ACOT8 RPRD1B NCOR1 ACSL6 GCLC B3GALT1 QPRT TRIM25 ZNF483 SOX11 GPAT4 MED17 DRG1 MAPKAPK5 SCAI C2ORF49 TONSL TP53 LIG1 ZBTB37 PDE12 EYA3 MAP3K2 ZNF570 PGM2L1 ZNF264 MTAP SLFN13 PSMD9 TMTC1 SBNO2 OGFOD1 C1ORF61 ZNF7	0.0688	0.2354	1.16
GO:0004721	phosphoprotein phosphatase activity	molecular_function	10/398	322/22360	PPP1R37 PLPP2 PPM1K PTPRM PDP2 GSK3B RCAN1 EYA3 STYX EIF2AK2	0.06884	0.2353	1.74
GO:0016070	RNA metabolic process	biological_process	118/398	5605/22360	BRDT ZNF286B RARG SMARCC1 FOXD2 HIF1AN ITGA3 PKNOX1 IKZF3 POU5F1 CX3CL1 TAF8 PNPT1 ZMYM5 OAS3 ZNF80 SON WNT4 GMEB1 CAMK1 FTO MLX SNORA13 DIS3L ADAR ZHX3 ZNF766 DDX51 ZNF583 MNT F2R TENM2 CRX XIAP ZNF814 SLC33A1 EXOSC6 CWC25 BCAS3 MXD1 NLRP3 OR7D2 ZNF8 RIPPLY3 HNRNPUL1 MED22 TCF4 EIF2AK2 SRSF10 ZC3H14 TSEN2 MLXIPL NPAT ZKSCAN1 ZNF800 DDX54 MTFMT RPL7L1 C9ORF78 GAS6 FCF1 IRAK4 PCGF5 RRP1 APOBEC3D WBP2 ZNF669 TRMT10B ZNF274 ZNF70 INTS6 YES1 ISG20L2 ZNF445 ZFPM2 SMAD2 SAMD4B NSRP1 ZNF677 SUMO2 ZNF430 KMT2D ARNT2 CHEK2 ZNF789 N4BP2L2 MDM4 ZNF345 ZNF573 TONSL SCAI C2ORF49 MED17 DRG1 PDE12 ZBTB37 TP53 ZNF264 SLFN13 ZNF570 MAP3K2 C1ORF61 ZNF7 SBNO2 PSMD9 TMTC1 APP CIART ZNF441 RCAN1 ZNF772 RPRD1B CCL5 GCLC NCOR1 ZNF483 SOX11 TRIM25	0.06893	0.2355	1.18
GO:0016773	phosphotransferase activity, alcohol group as acceptor	molecular_function	35/398	1449/22360	DGKZ PRKAR2A CCL5 ADAR TBCK GRK5 APP MKNK1 MAPK12 CAMK1 MAP3K9 SRCIN1 GAS6 CXCR4 IRAK4 TNK2 F2R CHEK2 GSK3B TAOK1 SMCR8 SHPK SYAP1 PIK3R3 AAK1 MAPKAPK5 YES1 GK5 ADCY2 TIE1 PGM2L1 EIF2AK2 AKT2 MAP3K2 PEAK1	0.06907	0.2355	1.36
GO:0030163	protein catabolic process	biological_process	26/398	1027/22360	UBE3C CYP51A1 STYX GCLC CHEK2 VPS28 ATXN3 GSK3B MDM4 RNF7 FBXL4 USP28 TTC3 USP44 UBXN2A PSMD9 TRIM25 TRIM72 SMARCC1 KLHL8 FBXL18 CTSS RNF115 CASP8 LGMN SUMO2	0.06944	0.2366	1.42
GO:0090305	nucleic acid phosphodiester bond	biological_process	11/398	364/22360	RFC2 PDE12 OAS3 DIS3L NMNAT1 ISG20L2 TSEN2 SLFN13 CCDC36 EXOSC6 PNPT1	0.07018	0.23	1.7

	hydrolysis							
GO:0034333	adherens junction assembly	biological_process	4/398	98/22360	BCAS3 WHAMM WNT4 PEAK1	0.0702	0.23	2.29
GO:0090559	regulation of membrane permeability	biological_process	4/398	98/22360	TP53 GSK3B CASP8 SLC35F6	0.0702	0.23	2.29
GO:0016491	oxidoreductase activity	molecular_function	24/398	936/22360	CYP4V2 FTO DUOX1 STEAP2 NDUFA10 COX5B CYB5R4 NXN PDP2 HIF1AN COQ6 PPM1K CRYZL1 CYB561A3 CYP51A1 HBE1 UEVLD ACOX1 KIAA1191 HP CNR2 OGFOD1 H6PD KDSR	0.07023	0.2298	1.44
GO:0002224	toll-like receptor signaling pathway	biological_process	6/398	168/22360	IRAK4 LGMN RFTN1 CASP8 CTSS OTUD4	0.07054	0.2306	2.01
GO:0016072	rRNA metabolic process	biological_process	8/398	244/22360	RPL7L1 DDX51 FCF1 EXOSC6 ISG20L2 RRP1 SLFN13 DIS3L	0.07099	0.232	1.84
GO:0006639	acylglycerol metabolic process	biological_process	5/398	133/22360	GPAT4 MGLL DGKZ FITM2 GK5	0.07232	0.2357	2.11
GO:0016358	dendrite development	biological_process	8/398	245/22360	SRCIN1 APP SEZ6 TMEM106B CAMK1 FARP1 GSK3B KLHL1	0.0725	0.2361	1.83
GO:0006470	protein dephosphorylation	biological_process	11/398	366/22360	EIF2AK2 STYX RCAN1 GSK3B EYA3 PDP2 PTPRM PPM1K RPRD1B PLPP2 PPP1R37	0.0726	0.2363	1.69
GO:0032838	plasma membrane bounded cell projection cytoplasm	cellular_component	7/398	207/22360	BBS1 DYNC2LI1 TRAF3IP1 DNAL1 ZC3H14 TTLL3 PRKAR2A	0.07343	0.2389	1.9
GO:0031267	small GTPase binding	molecular_function	14/398	494/22360	TBCK FGD5 SMCR8 SRGAP2B RAB29 RILPL1 VPS52 ARHGEF39 MYO1C WHAMM DENND2A MYO5A RABGAP1 FARP1	0.07399	0.2405	1.59
GO:0006638	neutral lipid metabolic process	biological_process	5/398	134/22360	GPAT4 MGLL DGKZ FITM2 GK5	0.07448	0.2412	2.1
GO:0019222	regulation of metabolic process	biological_process	160/398	7804/22360	NCOR1 GSK3B GCLC TRIM25 EMC10 FDPS SOX11 ZNF483 ZNF772 RCAN1 ZNF441 MAP3K9 PDP2 CIART NXN APP CCL5 RPRD1B ACOT8 EYA3 MAP3K2 VPS28 ZNF570 STYX ZNF264 FAM161A PSMD9 OGFOD1 SBNO2 ZNF7 C1ORF61 MAPKAPK5 MED17 MYO1C SCAI PIK3R3 SYAP1 TONSL TP53 ZBTB37 PDE12 ATP6V1A N4BP2L2 TAOK1 ZNF789 ATXN3 SPN CHEK2 KMT2D ZNF430 ARNT2 USP44 ZNF573 ZNF345 MDM4 CNR2 NSRP1 SAMD4B SMAD2 MKNK1 SUMO2 ZNF677 CASP8 NOA1 LTBR ZNF274 OTUD4 ZNF669 ZFPM2 ZNF445 LRPAP1 YES1 ADCY2 ZNF70 SMCR8 PPP1R37 WBP2 NMNAT1 C9ORF78 CYP51A1 CXCR4 IRAK4 PCGF5 CNKSR3 GAS6 DDX54 ITIH5 ZKSCAN1 ZNF800 C5AR2 MAPK12 FITM2 DGKZ SRSF10 P2RX4 EIF2AK2 TCF4 AKT2 MED22 NPAT MLXIPL UBXN2A MIR654 ZC3H14 BCAS3 EXOSC6 SLC33A1 ZNF814 XIAP ZNF8 RIPPLY3 PPM1K NLRP3 OR7D2 RFC2 MXD1 HP DCUN1D2 ACOX1 ZNF583 FARP1 CRX TENM2 F2R TNK2 TRAF3IP1 SRCIN1 MNT MLX PAPLN CAMK1 FTO LGMN ZNF766 ZHX3 ADAR PRKAR2A ZMYM5 PNPT1 TAF8 TRABD2B CX3CL1 GMEB1 WNT4 ZNF80 SON OAS3 PGF ITGA3 HIF1AN RARG FOXD2 SMARCC1 BRDT ZNF286B RAB29 POU5F1 PKNOX1 MSH6 IKZF3	0.0745	0.2411	1.15
GO:0022890	inorganic cation transmembrane transporter activity	molecular_function	22/398	851/22360	SLC9A7 KCNN3 ATP2B1 P2RX4 STAC2 CNKSR3 GAS6 ANO6 SLC39A13 SLC33A1 CACNB2 TTYH1 SLC30A6 MYO5A COX5B APP ABCC9 KCNJ15 SLC12A2 ATP6V1A CTSS SLC9A4	0.07515	0.2431	1.45
GO:0030641	regulation of cellular pH	biological_process	4/398	100/22360	ATP6V1A SLC9A4 SLC9A7 VPS33A	0.0753	0.2434	2.25
GO:1990830	cellular response to leukemia inhibitory factor	biological_process	4/398	100/22360	SBNO2 UBXN2A RARG TRIM25	0.0753	0.2434	2.25
GO:0043547	positive regulation of GTPase activity	biological_process	13/398	453/22360	TBCK WNT4 F2R CCL5 CCDC125 BCAS3 GSK3B ELMOD1 RGS17 AKT2 RGS12 CX3CL1 RABGAP1	0.076	0.2449	1.61

GO:0043085	positive regulation of catalytic activity	biological_process	37/398	1559/22360	DGKZ PRKAR2A CCL5 CASP8 LGMN TBCK TOR1AIP2 RABGAP1 MAPK12 CAMK1 APP PDP2 MAP3K9 CCDC125 GAS6 SRCIN1 F2R CXCR4 RGS12 GSK3B DCUN1D2 TAOK1 MSH6 RFC2 NLRP3 PPM1K SYAP1 MAPKAPK5 BCAS3 ADCY2 WNT4 CX3CL1 AKT2 EIF2AK2 RGS17 MAP3K2 ELMOD1	0.07736	0.2491	1.33
GO:0030117	membrane coat	cellular_component	4/398	101/22360	AP4E1 AP4M1 SYNRG VPS33A	0.07793	0.2443	2.22
GO:0016301	kinase activity	molecular_function	37/398	1560/22360	GK5 ADCY2 YES1 TIE1 AKT2 PGM2L1 EIF2AK2 PEAK1 MAP3K2 SHPK SMCR8 SCYL3 SYAP1 PIK3R3 MAPKAPK5 AAK1 GAS6 SRCIN1 TNK2 F2R IRAK4 CXCR4 CHEK2 GSK3B TAOK1 PRKAR2A DGKZ ADAR CCL5 NAGK TBCK MAPK12 MKNK1 CAMK1 APP GRK5 MAP3K9	0.07795	0.2442	1.33
GO:0015630	microtubule cytoskeleton	cellular_component	31/398	1274/22360	MYO5A BCAS3 SPTAN1 WHAMM FHDC1 KRT18 EYA3 ODF2L YES1 TTI2 RILPL1 FAM161A BBS1 TSEN2 APP RABGAP1 DNAL1 PRKAR2A RPRD1B DIS3L TBCK TBCCD1 TTLL3 CEP112 NCOR1 TTLL7 TAOK1 GSK3B TRAF3IP1 DYNC2LI1 PCGF5	0.07811	0.2445	1.37
GO:0030426	growth cone	cellular_component	6/398	172/22360	SYAP1 MYO5A APP PCDH9 TENM2 ITGA3	0.07815	0.2445	1.96
GO:0008194	UDP-glycosyltransferase activity	molecular_function	6/398	172/22360	A4GALT KDELC2 B3GALT1 GSK3B GALNT15 GALNT16	0.07815	0.2445	1.96
GO:0120025	plasma membrane bounded cell projection	cellular_component	51/398	2243/22360	ZC3H14 BAALC FGD5 FAM161A FHDC1 AKT2 ATP2B1 P2RX4 ATP6V1A SYAP1 MYO1C DYNC2LI1 RGS12 TTLL3 GSK3B DGKZ DNAL1 APP PCDH9 RILPL1 ADCY2 PDE4C BBS1 CX3CL1 KCNN3 RAB22A RSPH3 RGS17 ODF2L CC2D2A GABRB3 KLHL1 SCYL3 SLC26A2 SYP MYO5A CCR6 AAK1 ITGA3 CNR2 SRCIN1 TRAF3IP1 TENM2 SEZ6 FARP1 PTPRM SPN TTLL7 PRKAR2A CASP8 TTYH1	0.07858	0.2456	1.28
GO:0007160	cell-matrix adhesion	biological_process	8/398	249/22360	WHAMM CX3CL1 WNT4 GSK3B ITGA3 BCAS3 PEAK1 PLEKHA2	0.07877	0.2448	1.81
GO:0090150	establishment of protein localization to membrane	biological_process	11/398	371/22360	CASP8 BBS1 TP53 ARFRP1 RILPL1 MYO1C SEC62 ZDHHC15 KRT18 RFTN1 AKT2	0.07889	0.2451	1.67
GO:0098662	inorganic cation transmembrane transport	biological_process	23/398	902/22360	CACNB2 SLC33A1 ANO6 SLC39A13 STEAP2 COX5B MYO5A SLC30A6 TTYH1 SLC9A4 KCNJ15 SLC12A2 ATP6V1A ABCC9 SLC9A7 ATP2B1 P2RX4 KCNN3 CX3CL1 STAC2 F2R GAS6 CNKSR3	0.07931	0.2462	1.43
GO:0045944	positive regulation of transcription by RNA polymerase II	biological_process	31/398	1276/22360	KMT2D ARNT2 ZNF345 CRX PCGF5 SOX11 MLX SMAD2 CAMK1 APP SUMO2 RPRD1B CX3CL1 TCF4 SBNO2 ZFPM2 MLXIPL GMEB1 C1ORF61 YES1 MED17 BCAS3 RARG FOXD2 SMARCC1 TP53 PKNOX1 IKZF3 POU5F1 NLRP3 WBP2	0.07944	0.2465	1.36
GO:0071773	cellular response to BMP stimulus	biological_process	6/398	173/22360	SOX11 SMAD2 XIAP SLC33A1 ITGA3 ZNF8	0.08014	0.2485	1.95
GO:0010634	positive regulation of epithelial cell migration	biological_process	6/398	173/22360	LGMN BCAS3 ITGA3 EMC10 P2RX4 CCR6	0.08014	0.2485	1.95
GO:0150034	distal axon	cellular_component	9/398	290/22360	AAK1 ITGA3 PCDH9 SYP SYAP1 MYO5A P2RX4 APP TENM2	0.08047	0.2492	1.74
GO:0003779	actin binding	molecular_function	13/398	457/22360	BCL7B CORO7 KLHL1 MYO1C SPTAN1 CACNB2 TMOD3 MYO5A WHAMM FHDC1 PSTPIP2 ADD2 CXCR4	0.08061	0.2495	1.6
GO:0032092	positive regulation of protein binding	biological_process	4/398	102/22360	APP SYAP1 GSK3B ADD2	0.08062	0.2494	2.2
GO:0009968	negative regulation of signal transduction	biological_process	34/398	1421/22360	CX3CL1 TRABD2B OTUD4 EYA3 CARD19 RGS17 LRPAP1 WNT4 PSMD9 SCAI TRIM72 HIF1AN ITGA3 MAPKAPK5 NLRP3 GCLC RGS12 GSK3B NCOR1 RABGAP1 GAS6 TRAF3IP1 CNKSR3 CCDC125 MNT EPN1 SLC35F6 SMAD2 RCAN1 NXN CCL5 LGMN CASP8 RNF115	0.08092	0.2502	1.34
GO:0070851	growth factor receptor binding	molecular_function	5/398	137/22360	YES1 APP IRAK4 PGF TNK2	0.08121	0.2509	2.05

GO:1901565	organonitrogen compound catabolic process	biological_process	34/398	1423/22360	SUMO2 LGMN CASP8 RNF115 RNF7 MDM4 TRIM25 QPRT USP44 USP28 TTC3 GCLC CHEK2 CYP51A1 GSK3B ATXN3 FBXL18 PPM1K CTSS GNS KLHL8 APOBEC3D SMARCC1 TRIM72 LRTOMT PSMD9 UBXN2A FBXL4 PDE4C ASPA STYX MTAP UBE3C VPS28	0.08221	0.2535	1.34
GO:0031401	positive regulation of protein modification process	biological_process	32/398	1329/22360	MAP3K9 C5AR2 APP CAMK1 PRKAR2A CCL5 DCUN1D2 TAOK1 CHEK2 CXCR4 TNK2 EMC10 F2R SRCIN1 GAS6 PGF MAPKAPK5 SYAP1 XIAP PIK3R3 TP53 NMNAT1 WBP2 MAP3K2 LTBR EIF2AK2 TRABD2B CX3CL1 AKT2 FAM161A YES1 ADCY2	0.08317	0.2564	1.35
GO:0010243	response to organonitrogen compound	biological_process	26/398	1046/22360	ADCY2 CNR2 ZNF106 TRIM25 AKT2 P2RX4 GCLC RGS17 PNPT1 ATXN3 GSK3B RABGAP1 GABRB3 ATP6V1A NLRP3 PRKAR2A LGMN TP53 DUOX1 TRIM72 SMARCC1 SYAP1 APP PIK3R3 MYO5A MYO1C	0.08321	0.2563	1.4
GO:0010522	regulation of calcium ion transport into cytosol	biological_process	4/398	103/22360	P2RX4 MYO5A CX3CL1 F2R	0.08336	0.255	2.18
GO:0032414	positive regulation of ion transmembrane transporter activity	biological_process	4/398	103/22360	CTSS CNKSR3 CACNB2 STAC2	0.08336	0.255	2.18
GO:0019395	fatty acid oxidation	biological_process	4/398	103/22360	CYP4V2 ACOT8 AKT2 ACOX1	0.08336	0.255	2.18
GO:0006641	triglyceride metabolic process	biological_process	4/398	103/22360	FITM2 GK5 GPAT4 MGLL	0.08336	0.255	2.18
GO:0007044	cell-substrate junction assembly	biological_process	4/398	103/22360	PEAK1 WNT4 BCAS3 WHAMM	0.08336	0.255	2.18
GO:0061097	regulation of protein tyrosine kinase activity	biological_process	4/398	103/22360	APP CCL5 SRCIN1 GAS6	0.08336	0.255	2.18
GO:0051707	response to other organism	biological_process	27/398	1093/22360	TRAF3IP1 CNR2 OAS3 CXCR4 TRIM25 SBNO2 F2R C19ORF66 EIF2AK2 IFNAR1 HNRNPUL1 CX3CL1 SLFN13 SPN HP NLRP3 ADAR CCL5 RAB29 IKZF3 PDE12 ABCC9 CCR4 CASP8 SHPK APOBEC3D MR1	0.08337	0.2541	1.39
GO:0000187	activation of MAPK activity	biological_process	7/398	213/22360	F2R MAPKAPK5 TAOK1 MAP3K9 MAP3K2 CXCR4 EIF2AK2	0.08404	0.256	1.85
GO:1903508	positive regulation of nucleic acid-templated transcription	biological_process	40/398	1716/22360	MAP3K2 TAF8 TCF4 CX3CL1 PSMD9 MLXIPL WNT4 NPAT GMEB1 SBNO2 ZFPM2 YES1 C1ORF61 BCAS3 MED17 SMARCC1 FOXD2 RARG BRDT TP53 NLRP3 WBP2 IKZF3 PKNOX1 POU5F1 CHEK2 ARNT2 KMT2D PCGF5 CRX ZNF345 F2R MNT SOX11 MLX APP SMAD2 CAMK1 SUMO2 RPRD1B	0.08462	0.2576	1.31
GO:0043207	response to external biotic stimulus	biological_process	27/398	1095/22360	SBNO2 F2R CXCR4 TRIM25 OAS3 CNR2 TRAF3IP1 SPN HP CX3CL1 HNRNPUL1 SLFN13 IFNAR1 EIF2AK2 C19ORF66 SHPK CASP8 CCR4 IKZF3 ABCC9 PDE12 NLRP3 CCL5 ADAR RAB29 MR1 APOBEC3D	0.0849	0.2583	1.39
GO:1902680	positive regulation of RNA biosynthetic process	biological_process	40/398	1717/22360	ZFPM2 SBNO2 GMEB1 NPAT WNT4 PSMD9 MLXIPL C1ORF61 YES1 MAP3K2 CX3CL1 TCF4 TAF8 TP53 POU5F1 PKNOX1 IKZF3 WBP2 NLRP3 MED17 BCAS3 BRDT RARG FOXD2 SMARCC1 F2R ZNF345 CRX PCGF5 MNT SOX11 KMT2D ARNT2 CHEK2 SUMO2 RPRD1B MLX CAMK1 SMAD2 APP	0.08522	0.2591	1.31
GO:0071407	cellular response to organic cyclic compound	biological_process	16/398	592/22360	ADCY2 NCOR1 GABRB3 KMT2D P2RX4 CHEK2 ATP2B1 GCLC CASP8 PDE12 WBP2 CCL5 DDX54 SSTR2 RARG APP	0.08529	0.2592	1.52
GO:0008637	apoptotic mitochondrial changes	biological_process	5/398	139/22360	GCLC TP53 GSK3B CASP8 SLC35F6	0.08591	0.2551	2.02
GO:0050801	ion homeostasis	biological_process	22/398	864/22360	CX3CL1 P2RX4 ATP2B1 GCLC SLC9A7 F2R CXCR4 MLXIPL XIAP STEAP2 APP CCR6 C5AR2 MYO5A CACNB2 SLC39A13 SLC12A2 ATP6V1A SLC9A4 CCL5 VPS33A CCR4	0.08604	0.2553	1.43
GO:0090575	RNA polymerase II transcription factor complex	cellular_component	6/398	176/22360	SMAD2 TCF4 RARG TAF8 MED17 TP53	0.08629	0.2559	1.92
GO:0061025	membrane fusion	biological_process	6/398	176/22360	AKT2 GAS6 UBXN2A TIE1 STX16 CXCR4	0.08629	0.2559	1.92

GO:0098656	anion transmembrane transport	biological_process	12/398	419/22360	SLC33A1 APOL1 ANO6 GABRB3 SLC26A2 AKT2 TTYH1 ABCC3 CLCC1 LRRC8B SLC12A2 SLC37A2	0.08647	0.2561	1.61
GO:0005929	cilium	cellular_component	17/398	638/22360	MYO5A CCR6 APP DNAL1 PRKAR2A FHDC1 RSPH3 TTLL3 ODF2L TTLL7 CC2D2A DYNC2LI1 RILPL1 TRAF3IP1 PDE4C FAM161A BBS1	0.08689	0.2572	1.5
GO:0031396	regulation of protein ubiquitination	biological_process	7/398	215/22360	XIAP GCLC NXN USP44 VPS28 UBXN2A DCUN1D2	0.08779	0.2598	1.83
GO:0031333	negative regulation of protein complex assembly	biological_process	5/398	140/22360	SPTAN1 TMOD3 ADD2 GSK3B TRAF3IP1	0.08832	0.2597	2.01
GO:0008509	anion transmembrane transporter activity	molecular_function	11/398	378/22360	ANO6 APOL1 SLC33A1 GABRB3 TTYH1 ABCC3 SLC26A2 CLCC1 LRRC8B SLC37A2 SLC12A2	0.08832	0.2597	1.63
GO:0000077	DNA damage checkpoint	biological_process	6/398	177/22360	TAOK1 TP53 USP28 CHEK2 MDM4 MSH6	0.0884	0.2596	1.9
GO:0000139	Golgi membrane	cellular_component	23/398	913/22360	COG1 NLRP3 ARFRP1 APP STEAP2 A4GALT SLC30A6 CORO7 GNPTG ZDHHC15 SLC39A13 GALNT16 STX16 AP4E1 SLC33A1 AP4M1 PCSK7 B3GALT1 WHAMM SLC9A7 VPS52 RAB40B GALNT15	0.08855	0.2599	1.42
GO:0033157	regulation of intracellular protein transport	biological_process	8/398	255/22360	MYO1C TP53 BCAS3 GSK3B CAMK1 AKT2 RAB29 GAS6	0.08884	0.2606	1.76
GO:0005884	actin filament	cellular_component	4/398	105/22360	YES1 MYO5A MYO1C PSTPIP2	0.08902	0.261	2.14
GO:0032006	regulation of TOR signaling	biological_process	4/398	105/22360	GAS6 TBCK SMCR8 MAPKAPK5	0.08902	0.261	2.14
GO:0034440	lipid oxidation	biological_process	4/398	105/22360	ACOT8 CYP4V2 AKT2 ACOX1	0.08902	0.261	2.14
GO:0031300	intrinsic component of organelle membrane	cellular_component	14/398	508/22360	PCSK7 SLC37A2 EMC10 FITM2 ESYT3 RAB3B TRABD2B P2RX4 A4GALT SYP STEAP2 SEC62 SLC39A13 STX16	0.08982	0.2622	1.55
GO:0046983	protein dimerization activity	molecular_function	34/398	1437/22360	SLC39A13 HIF1AN PGF ITGA3 SYAP1 TP53 MSH6 PKNOX1 IKZF3 MXD1 CD3G TCF4 TAF8 P2RX4 MLXIPL ADCY2 RILPL1 ANO6 GNPTG MAP3K9 MLX SMAD2 ADD2 ZHX3 CCL5 SLC9A7 ARNT2 ACSL6 GCLC CHEK2 TENM2 MGLL QPRT MNT	0.09172	0.2676	1.33
GO:0016788	hydrolase activity, acting on ester bonds	molecular_function	28/398	1151/22360	GSK3B PTPRM FARP1 MGLL H6PD RCAN1 PDP2 PLPP2 CCL5 PRKAR2A ACOT8 NT5DC3 DIS3L EYA3 PNPT1 EIF2AK2 SLFN13 STYX ASPA TSEN2 PDE4C ISG20L2 OAS3 EXOSC6 PPP1R37 PPM1K GNS PDE12	0.09185	0.2678	1.37
GO:0032091	negative regulation of protein binding	biological_process	4/398	106/22360	STYX CAMK1 LRPAP1 GSK3B	0.09193	0.2679	2.12
GO:0048024	regulation of mRNA splicing, via spliceosome	biological_process	4/398	106/22360	SRSF10 NSRP1 SON C9ORF78	0.09193	0.2679	2.12
GO:0008104	protein localization	biological_process	67/398	3069/22360	PSMD9 AP4M1 UEVLD VPS28 VPS52 RAB40B STYX AKT2 TP53 ATP6V1A NLRP3 MYO1C CACNB2 CYB5R4 ZDHHC15 CALCRL BCAS3 TONSL DYNC2LI1 GAS6 SEC62 GSK3B CYP51A1 CEP112 SNX27 PIGR ACOT8 ARFRP1 CCL5 CORO7 SNX21 C5AR2 DENND2A STAC2 BBS1 RILPL1 CC2D2A KRT18 RAB22A CX3CL1 TAF8 BTN3A2 MCM3AP WBP2 CD3G RAB29 AP4E1 ITGA3 AAK1 MYO5A F2R SRCIN1 TRAF3IP1 ACOX1 ATXN3 RFTN1 RAB3B CASP8 TBCK TOR1AIP2 VPS33A COG1 STX16 SMAD2 RABGAP1 CAMK1 SYNRG	0.09232	0.2687	1.23
GO:0004518	nuclease activity	molecular_function	8/398	257/22360	ISG20L2 OAS3 DIS3L SLFN13 PDE12 TSEN2 EXOSC6 PNPT1	0.09238	0.2687	1.75
GO:0015291	secondary active transmembrane transporter activity	molecular_function	8/398	257/22360	SLC37A2 SLC26A2 SLC12A2 SLC35E1 SLC9A4 SLC33A1 SLC35E2B SLC9A7	0.09238	0.2687	1.75
GO:0050792	regulation of viral process	biological_process	8/398	257/22360	TRIM25 EIF2AK2 C19ORF66 APOBEC3D ADAR CCL5 PDE12 OAS3	0.09238	0.2687	1.75

GO:0042995	cell projection	cellular_component	52/398	2319/22360	DNAL1 DGKZ COQ6 APP PCDH9 DYNC2LI1 RGS12 TTLL3 GSK3B ATP6V1A SYAP1 MYO1C ZC3H14 BAALC FGD5 FAM161A AKT2 FHDC1 ATP2B1 P2RX4 PRKAR2A CASP8 TTYH1 CNR2 TRAF3IP1 SRCIN1 TENM2 FARP1 SEZ6 PTPRM TTLL7 SPN SCYL3 SLC26A2 MYO5A CCR6 SYP AAK1 ITGA3 RILPL1 ADCY2 PDE4C BBS1 CX3CL1 KCNN3 RAB22A RSPH3 ODF2L RGS17 GABRB3 KLHL1 CC2D2A	0.09252	0.2687	1.26
GO:0001934	positive regulation of protein phosphorylation	biological_process	27/398	1105/22360	TP53 CCL5 PRKAR2A NMNAT1 PGF MAP3K9 MAPKAPK5 C5AR2 PIK3R3 APP SYAP1 CAMK1 CXCR4 EMC10 F2R TNK2 SRCIN1 YES1 GAS6 ADCY2 TAOK1 LTBR MAP3K2 EIF2AK2 CHEK2 AKT2 CX3CL1	0.09283	0.269	1.37
GO:0016604	nuclear body	cellular_component	23/398	918/22360	SPN SRSF10 CHEK2 TRIM25 NPAT TENM2 ZNF106 USP28 SON FBXL4 ZC3H14 CWC25 CIART NSRP1 DRG1 MYO1C GRK5 FTO TONSL SUMO2 TP53 DGKZ NMNAT1	0.093	0.2693	1.41
GO:0035326	enhancer binding	molecular_function	5/398	142/22360	SOX11 SMAD2 SMARCC1 CIART TP53	0.09327	0.2692	1.98
GO:0032434	regulation of proteasomal ubiquitin-dependent protein catabolic process	biological_process	5/398	142/22360	STYX GCLC SUMO2 GSK3B SMARCC1	0.09327	0.2692	1.98
GO:0044798	nuclear transcription factor complex	cellular_component	7/398	218/22360	MED17 TP53 SMAD2 SOX11 TAF8 RARG TCF4	0.09362	0.2699	1.8
GO:0018958	phenol-containing compound metabolic process	biological_process	4/398	107/22360	DUOX1 SULT1B1 MYO5A LRTOMT	0.09489	0.267	2.1
GO:0006885	regulation of pH	biological_process	4/398	107/22360	ATP6V1A SLC9A7 VPS33A SLC9A4	0.09489	0.267	2.1
GO:0050710	negative regulation of cytokine secretion	biological_process	4/398	107/22360	C5AR2 GAS6 NLRP3 CX3CL1	0.09489	0.267	2.1
GO:0030672	synaptic vesicle membrane	cellular_component	4/398	107/22360	SYT12 STX16 RAB3B SYP	0.09489	0.267	2.1
GO:0032651	regulation of interleukin-1 beta production	biological_process	4/398	107/22360	NLRP3 APP CX3CL1 F2R	0.09489	0.267	2.1
GO:0034645	cellular macromolecule biosynthetic process	biological_process	113/398	5429/22360	MSH6 PKNOX1 IKZF3 POU5F1 RARG SMARCC1 FOXD2 BRDT ZNF286B HIF1AN ITGA3 GALNT16 PIGL WNT4 GMEB1 ZNF80 TAF8 CX3CL1 ZMYM5 ZHX3 ZNF766 A4GALT MMS22L CAMK1 FTO MLX MNT CRX TENM2 F2R ZNF583 NLRP3 OR7D2 MXD1 RFC2 ZNF8 RIPPLY3 XIAP ZNF814 ZDHHC15 BCAS3 SLC33A1 MLXIPL NPAT TCF4 EIF2AK2 MED22 AKT2 SRSF10 GALNT15 MTFMT ZKSCAN1 ZNF800 GAS6 IRAK4 PCGF5 WBP2 INTS6 YES1 ZNF445 ZNF70 ZFPM2 ZNF669 ZNF274 NOA1 ZNF677 SUMO2 SMAD2 MKNK1 SAMD4B MDM4 ZNF345 ZNF573 CHEK2 ZNF430 KMT2D ARNT2 ZNF789 N4BP2L2 ZBTB37 LIG1 TP53 SCAI TONSL MED17 DRG1 MAPKAPK5 C1ORF61 ZNF7 PSMD9 TMTC1 SBNO2 OGFOD1 PGM2L1 ZNF264 MAP3K2 ZNF570 CCL5 ACOT8 RPRD1B APP RCAN1 ZNF441 ZNF772 CIART KDELC2 ZNF483 SOX11 B3GALT1 TRIM25 GCLC GSK3B NCOR1	0.09517	0.2671	1.17
GO:0001701	in utero embryonic development	biological_process	11/398	383/22360	TANC2 POU5F1 EPN1 RPL7L1 CASP8 ZFPM2 TIE1 TAF8 SMAD2 ARNT2 SLC35E2B	0.09552	0.2679	1.61
GO:0010033	response to organic substance	biological_process	79/398	3684/22360	LGMN CASP8 PRKAR2A ADAR STEAP2 CSF2RA SMAD2 DUOX1 F2R CNR2 ATXN3 HP RFTN1 CHEK2 HBE1 ARNT2 KMT2D CTSS WBP2 ITGA3 PGF SYP SMARCC1 RARG MYO5A CCR6 WNT4 YES1 OAS3 ADCY2 GABRB3 KRT18 LTBR RGS17 PNPT1 C19ORF66 IFNAR1 CX3CL1 OTUD4 CCR4 CCL5 SLC12A2 DDX54 APP MAPK12 SSTR2 IRAK4 CXCR4 TRIM25 GAS6 SOX11 GSK3B NCOR1 RABGAP1 GCLC TP53 ZNF8 SHPK NLRP3 ATP6V1A PDE12 CALCRL SLC33A1 MYO1C SYAP1 XIAP PIK3R3 TRIM72 MLXIPL PSMD9 SBNO2 TIE1 ZNF106 UBXN2A EIF2AK2 ATP2B1 P2RX4 AKT2 MTAP	0.09564	0.2681	1.2
GO:0007498	mesoderm development	biological_process	5/398	143/22360	SMAD2 IKZF3 ZFPM2 TIE1 ITGA3	0.09581	0.2684	1.96

GO:1903531	negative regulation of secretion by cell	biological_process	8/398	259/22360	PSMD9 F2R C5AR2 GAS6 NLRP3 SRCIN1 CYP51A1 CX3CL1	0.09601	0.2688	1.74
GO:1901698	response to nitrogen compound	biological_process	28/398	1158/22360	ADCY2 ZNF106 AKT2 P2RX4 PNPT1 RGS17 GABRB3 PDE12 ATP6V1A NLRP3 TP53 TRIM72 MYO5A PIK3R3 SYAP1 SMARCC1 MYO1C CNR2 TRIM25 GCLC RFTN1 RABGAP1 GSK3B ATXN3 PRKAR2A LGMN DUOX1 APP	0.09755	0.2727	1.36
GO:0005216	ion channel activity	molecular_function	17/398	648/22360	GABRB3 KCNN3 P2RX4 STAC2 CLCC1 LRRC8B GAS6 ANO6 GJC1 APOL1 CACNB2 TTYH1 MYO5A APP ABCC9 KCNJ15 CTSS	0.09776	0.2731	1.47
GO:0046928	regulation of neurotransmitter secretion	biological_process	4/398	108/22360	SYP GSK3B SYT12 CACNB2	0.09792	0.2734	2.08
GO:0046822	regulation of nucleocytoplasmic transport	biological_process	4/398	108/22360	TP53 GAS6 GSK3B CAMK1	0.09792	0.2734	2.08
GO:0060079	excitatory postsynaptic potential	biological_process	4/398	108/22360	GSK3B P2RX4 APP SEZ6	0.09792	0.2734	2.08
GO:0015980	energy derivation by oxidation of organic compounds	biological_process	9/398	301/22360	NOA1 CS PYGB AKT2 COX5B NDUFA10 PGM2L1 PNPT1 GSK3B	0.09822	0.2727	1.68
GO:0051270	regulation of cellular component movement	biological_process	28/398	1159/22360	CX3CL1 AKT2 P2RX4 ARHGEF39 TIE1 WNT4 PIK3R3 CCR6 SCAI MYO1C ITGA3 PGF BCAS3 SRGAP2B PTPRM SPN CCDC125 GAS6 SRCIN1 F2R EMC10 DUOX1 APP C5AR2 ANO6 CCL5 TBCCD1 LGMN	0.09838	0.273	1.36
GO:0019955	cytokine binding	molecular_function	5/398	144/22360	CCR6 IFNAR1 CSF2RA CCR4 CXCR4	0.09839	0.2728	1.95
GO:0015631	tubulin binding	molecular_function	10/398	343/22360	FAM161A DNAL1 TRAF3IP1 DRG1 BCAS3 TAOK1 TTLL7 RABGAP1 FHDC1 WHAMM	0.0989	0.2741	1.64
GO:0016874	ligase activity	molecular_function	6/398	182/22360	GCLC LIG1 TTLL3 ACSL6 TTLL7 TRIM25	0.09948	0.2756	1.85
GO:0051402	neuron apoptotic process	biological_process	8/398	261/22360	GABRB3 LGMN F2R GCLC APP NMNAT1 AKT2 CX3CL1	0.09973	0.2761	1.72
GO:0045177	apical part of cell	cellular_component	12/398	429/22360	CNKSR3 SLC9A4 DYNC2LI1 SLC12A2 ATP6V1A LGMN APP ATP2B1 MARVELD2 C5AR2 DUOX1 SLC26A2	0.1002	0.2773	1.57
GO:0043170	macromolecule metabolic process	biological_process	221/398	11118/22360	MLX PAPLN A4GALT CAMK1 FTO CSF2RA TBCK DDX51 RAB3B ZHX3 SNORA13 DIS3L CRX F2R TRAF3IP1 GALNT16 RARG BRDT ZNF286B IKZF3 ZMYM5 DDI2 PNPT1 DPP9 TAF8 TRABD2B WNT4 ZNF80 PIGL DDX54 ITIH5 ZKSCAN1 RPL7L1 PLPP2 MTFMT TTLL3 IRAK4 USP28 CNKSR3 GAS6 DESI1 BCAS3 ZDHHC15 ZNF814 TRIM72 RIPPLY3 PPM1K OR7D2 RAB40B SRSF10 EIF2AK2 TCF4 HNRNPUL1 NPAT UBXN2A TIE1 FBXL4 MIR654 NOA1 TTLL7 N4BP2L2 ZNF789 SPN ZNF430 ARNT2 USP44 ZNF573 ZNF345 MDM4 AAK1 CCR6 PPP1R37 GNS LTBR ZNF274 ZNF445 INTS6 ISG20L2 ADCY2 RCAN1 ZNF441 MAP3K9 CIART PDP2 NXN RNF115 RPRD1B TRIM25 B3GALT1 MYO1C MED17 DRG1 PIK3R3 SCAI SYAP1 METTL21A EYA3 VPS28 PEAK1 PGM2L1 STYX SLFN13 UBE3C FAM161A TMTC1 PSMD9 SBNO2 ZNF7 C1ORF61 GRK5 MMS22L LGMN ZNF766 ADAR PRKAR2A HP DCUN1D2 ZNF583 TENM2 TTC3 TNK2 SRCIN1 MNT ITGA3 PGF HIF1AN FOXD2 SMARCC1 RAB29 POU5F1 PKNOX1 MSH6 CX3CL1 GMEB1 SON OAS3 ZNF800 C5AR2 MAPK12 C9ORF78 CYP51A1 CXCR4 PCGF5 FCF1 SUMF2 C9ORF84 CWC25 EXOSC6 SPTAN1 SLC33A1 XIAP CCDC36 ZNF8 NLRP3 RFC2 MXD1 GALNT15 UEVLD AKT2 MED22 TSEN2 MLXIPL PYGB ZC3H14 NSRP1 SAMD4B SMAD2 MKNK1 SUMO2 ZNF677 CASP8 NDUFAF7 TAOK1 ATXN3 PTPRM CHEK2 KMT2D APOBEC3D RRP1 MCM3AP SCYL3 SMCR8 CTSS WBP2 FBXL18 NMNAT1 KLHL8 TRMT10B ZNF669 OTUD4 ZFPM2 LRPAP1 YES1 PCSK7 ZNF70 ZNF772 APP CCL5 ACOT8 NCOR1 GSK3B GCLC EMC10 KDELC2 RNF7 SOX11 ZNF483 HERC4 CALCRL APOL1 MAPKAPK5 C2ORF49 TONSL TP53 ZBTB37 LIG1 PDE12 MAP3K2 ZNF570 ZNF264 OGFOD1	0.1006	0.2778	1.12

GO:0019233	sensory perception of pain	biological_process	4/398	109/22360	MGLL P2RX4 CNR2 F2R	0.101	0.2787	2.06
GO:0016651	oxidoreductase activity, acting on NAD(P)H	molecular_function	5/398	145/22360	DUOX1 CYB5R4 NXN CRYZL1 NDUFA10	0.101	0.2787	1.94
GO:0005667	transcription factor complex	cellular_component	11/398	387/22360	TAF8 TCF4 RARG ARNT2 SMAD2 EYA3 MED17 SOX11 PKNOX1 TP53 MLXIPL	0.1016	0.2801	1.6
GO:0006811	ion transport	biological_process	42/398	1841/22360	SLC37A2 SLC25A26 XKR4 STAC2 ATP2B1 P2RX4 CX3CL1 KCNN3 AKT2 GABRB3 CTSS ATP6V1A SYT12 ABCC9 MYO5A SLC26A2 CALCRL SLC33A1 CYB5R4 CACNB2 SLC39A13 APOL1 GAS6 CNKSR3 LRRC8B CLCC1 F2R ABCC3 SLC9A7 NCOR1 SLC9A4 CCL5 KCNJ15 SLC12A2 RAB3B STEAP2 COX5B APP TTYH1 SLC30A6 GJC1 ANO6	0.1021	0.2813	1.28
GO:0010498	proteasomal protein catabolic process	biological_process	14/398	518/22360	ATXN3 GSK3B SMARCC1 GCLC TRIM72 STYX PSMD9 TRIM25 SUMO2 FBXL4 UBXN2A USP44 RNF7 FBXL18	0.1025	0.2823	1.52
GO:0032872	regulation of stress-activated MAPK cascade	biological_process	8/398	263/22360	APP EIF2AK2 MAP3K2 MAP3K9 NCOR1 LTBR TAOK1 EMC10	0.1036	0.2795	1.71
GO:0038127	ERBB signaling pathway	biological_process	5/398	146/22360	EPN1 LGMN PIGR RNF115 APP	0.1037	0.2796	1.92
GO:0051261	protein depolymerization	biological_process	4/398	110/22360	TMOD3 SPTAN1 TAOK1 ADD2	0.1041	0.2805	2.04
GO:0006665	sphingolipid metabolic process	biological_process	6/398	184/22360	A4GALT ESYT3 SUMF2 KDSR PLPP2 B3GALT1	0.1041	0.2805	1.83
GO:0007266	Rho protein signal transduction	biological_process	7/398	224/22360	ITGA3 ARHGEF39 FGD5 F2R SCAI FARP1 CCDC125	0.106	0.2851	1.76
GO:0055037	recycling endosome	cellular_component	7/398	224/22360	SLC9A7 PLEKHB2 VPS52 RAB29 MYO5A C1ORF210 APP	0.106	0.2851	1.76
GO:0008154	actin polymerization or depolymerization	biological_process	7/398	224/22360	MYO1C SPTAN1 TMOD3 ADD2 CORO7 PSTPIP2 WHAMM	0.106	0.2851	1.76
GO:0007568	aging	biological_process	11/398	390/22360	APP ATP2B1 GCLC CHEK2 CX3CL1 MAPKAPK5 PNPT1 MSH6 SLC12A2 MNT TP53	0.1063	0.2854	1.58
GO:0014013	regulation of gliogenesis	biological_process	5/398	147/22360	CXCR4 APP P2RX4 ASPA SOX11	0.1064	0.2856	1.91
GO:0007416	synapse assembly	biological_process	6/398	185/22360	GABRB3 ADD2 FARP1 PCDHB9 APP RAB29	0.1065	0.2857	1.82
GO:0017016	Ras GTPase binding	molecular_function	13/398	477/22360	MYO1C ARHGEF39 VPS52 FARP1 RABGAP1 WHAMM MYO5A DENND2A SMCR8 FGD5 TBCK RAB29 SRGAP2B	0.1066	0.2858	1.53
GO:1902936	phosphatidylinositol bisphosphate binding	molecular_function	4/398	111/22360	PLEKHA2 GSDMA GSDMB SNX21	0.1073	0.2875	2.02
GO:1901888	regulation of cell junction assembly	biological_process	4/398	111/22360	MYO1C PEAK1 WNT4 BCAS3	0.1073	0.2875	2.02
GO:0032411	positive regulation of transporter activity	biological_process	4/398	111/22360	CNKSR3 CTSS STAC2 CACNB2	0.1073	0.2875	2.02
GO:0070302	regulation of stress-activated protein kinase signaling cascade	biological_process	8/398	265/22360	APP EIF2AK2 EMC10 NCOR1 LTBR TAOK1 MAP3K9 MAP3K2	0.1075	0.2876	1.7
GO:0009117	nucleotide metabolic process	biological_process	15/398	567/22360	ACOT8 NMNAT1 SULT1B1 DCTD SLC26A2 COX5B PDP2 GPAT4 ADCY2 PDE4C QPRT MLXIPL PGM2L1 ACSL6 NCOR1	0.1085	0.2897	1.49
GO:0009165	nucleotide biosynthetic process	biological_process	9/398	307/22360	PDP2 GPAT4 SLC26A2 DCTD ACSL6 COX5B QPRT ADCY2 NMNAT1	0.1089	0.2899	1.65
GO:0006886	intracellular protein transport	biological_process	30/398	1267/22360	ARFRP1 ACOT8 SNX27 TBCK RAB3B RABGAP1 CAMK1 SYNRG STX16 GAS6 TRAF3IP1 DYNC2LI1 GSK3B RFTN1 SEC62 ACOX1 RAB29 MCM3AP TP53 ZDHHC15 BCAS3 AP4E1 MYO1C RILPL1 AP4M1 RAB22A AKT2 STYX RAB40B VPS28	0.1091	0.2902	1.33
GO:0032270	positive regulation of cellular protein metabolic process	biological_process	40/398	1753/22360	CASP8 LGMN SUMO2 PRKAR2A CCL5 MAP3K9 MAPK12 CAMK1 APP C5AR2 TNK2 EMC10 F2R CXCR4 GAS6 SRCIN1 GSK3B ATXN3 DCUN1D2 TAOK1 CHEK2 GCLC TP53 NLRP3 NMNAT1 WBP2 MAPKAPK5 PGF XIAP SYAP1 PIK3R3 FAM161A ADCY2 YES1 MAP3K2 LTBR CX3CL1 TRABD2B AKT2 EIF2AK2	0.1093	0.2906	1.28

GO:0010557	positive regulation of macromolecule biosynthetic process	biological_process	45/398	2000/22360	MAP3K2 TCF4 TAF8 AKT2 CX3CL1 GMEB1 NPAT WNT4 PSMD9 MLXIPL ZFPM2 SBNO2 C1ORF61 YES1 BCAS3 MAPKAPK5 MED17 RARG SMARCC1 FOXD2 BRDT TP53 WBP2 NLRP3 POU5F1 RFC2 PKNOX1 IKZF3 SPN CHEK2 KMT2D ARNT2 PCGF5 CRX F2R ZNF345 MNT SOX11 MLX APP CAMK1 SMAD2 SUMO2 CCL5 RPRD1B	0.1097	0.2915	1.26
GO:0003924	GTPase activity	molecular_function	21/398	843/22360	RAB3B TBCK ARFRP1 RAB29 CCL5 DRG1 BCAS3 RABGAP1 TNK2 F2R WNT4 FGD5 CCDC125 RGS17 ELMOD1 RAB40B GSK3B CX3CL1 RGS12 RAB22A AKT2	0.1108	0.2943	1.4
GO:0042180	cellular ketone metabolic process	biological_process	8/398	267/22360	PDP2 PSMD9 MLXIPL CRYZL1 KDSR WNT4 AKT2 COQ6	0.1115	0.296	1.68
GO:0043484	regulation of RNA splicing	biological_process	5/398	149/22360	SON C9ORF78 SRSF10 NSRP1 BRDT	0.1119	0.2969	1.89
GO:0033135	regulation of peptidyl-serine phosphorylation	biological_process	5/398	149/22360	GAS6 CNKSR3 APP CAMK1 MLXIPL	0.1119	0.2969	1.89
GO:0045121	membrane raft	cellular_component	10/398	351/22360	ADD2 F2R BAALC CASP8 PRKAR2A ADCY2 RFTN1 MYO1C APP ATP2B1	0.1124	0.2921	1.6
GO:0005976	polysaccharide metabolic process	biological_process	4/398	113/22360	PGM2L1 GSK3B AKT2 PYGB	0.1139	0.2959	1.99
GO:0098857	membrane microdomain	cellular_component	10/398	352/22360	ADCY2 PRKAR2A F2R CASP8 BAALC ADD2 ATP2B1 APP MYO1C RFTN1	0.1141	0.2962	1.6
GO:0020037	heme binding	molecular_function	5/398	150/22360	CYP51A1 DUOX1 CYP4V2 HBE1 CYB5R4	0.1148	0.2968	1.87
GO:0006790	sulfur compound metabolic process	biological_process	11/398	396/22360	SULT1B1 GNS ACOT8 GPAT4 PDP2 GCLC IBA57 ACSL6 MTAP NUBPL SLC26A2	0.1161	0.3001	1.56
GO:0006753	nucleoside phosphate metabolic process	biological_process	15/398	573/22360	ADCY2 PDE4C QPRT MLXIPL PGM2L1 ACSL6 NCOR1 ACOT8 NMNAT1 SULT1B1 DCTD SLC26A2 COX5B PDP2 GPAT4	0.1166	0.3008	1.47
GO:0043393	regulation of protein binding	biological_process	7/398	229/22360	APP SYAP1 LRPAP1 STYX CAMK1 GSK3B ADD2	0.1171	0.3019	1.72
GO:0019208	phosphatase regulator activity	molecular_function	4/398	114/22360	RCAN1 EIF2AK2 PPP1R37 STYX	0.1172	0.302	1.97
GO:0009259	ribonucleotide metabolic process	biological_process	12/398	441/22360	NCOR1 GPAT4 PDP2 PGM2L1 ACSL6 COX5B SLC26A2 MLXIPL PDE4C SULT1B1 ACOT8 ADCY2	0.1185	0.3052	1.53
GO:0051247	positive regulation of protein metabolic process	biological_process	42/398	1865/22360	CHEK2 GCLC ATXN3 GSK3B SPN DCUN1D2 TAOK1 GAS6 SRCIN1 TNK2 F2R EMC10 CXCR4 MAPK12 CAMK1 APP C5AR2 MAP3K9 PRKAR2A CCL5 CASP8 LGMN SUMO2 TRABD2B CX3CL1 AKT2 EIF2AK2 VPS28 MAP3K2 LTBR ADCY2 YES1 FAM161A XIAP SYAP1 PIK3R3 MAPKAPK5 PGF NLRP3 NMNAT1 WBP2 TP53	0.1193	0.3071	1.27
GO:0014070	response to organic cyclic compound	biological_process	24/398	992/22360	PNPT1 RFTN1 GABRB3 NCOR1 MTAP ARNT2 KMT2D P2RX4 ATP2B1 GCLC HBE1 CHEK2 TRIM25 ADCY2 DDX54 DUOX1 SSTR2 SMAD2 APP RARG CASP8 PDE12 CCL5 WBP2	0.1196	0.3077	1.36
GO:0050684	regulation of mRNA processing	biological_process	5/398	152/22360	ZC3H14 SON C9ORF78 SRSF10 NSRP1	0.1205	0.3085	1.85
GO:0050773	regulation of dendrite development	biological_process	5/398	152/22360	SRCIN1 SEZ6 TMEM106B CAMK1 GSK3B	0.1205	0.3085	1.85
GO:0004843	thiol-dependent ubiquitin-specific protease activity	molecular_function	4/398	115/22360	USP44 USP28 OTUD4 ATXN3	0.1206	0.3085	1.95
GO:0031124	mRNA 3'-end processing	biological_process	4/398	115/22360	PNPT1 RPRD1B APP ZC3H14	0.1206	0.3085	1.95
GO:1901214	regulation of neuron death	biological_process	11/398	399/22360	CASP8 F2R LGMN RILPL1 CCL5 RAB29 NMNAT1 GABRB3 GSK3B CX3CL1 GCLC	0.1212	0.3097	1.55
GO:0051403	stress-activated MAPK cascade	biological_process	9/398	314/22360	EMC10 IRAK4 APP EIF2AK2 LTBR NCOR1 TAOK1 MAP3K2 MAP3K9	0.1223	0.3072	1.61

GO:0035556	intracellular signal transduction	biological_process	67/398	3127/22360	RFC2 NLRP3 TP53 PIK3R3 SCAI XIAP SYAP1 MAPKAPK5 SPTAN1 CALCRL FGD5 MLXIPL PSMD9 STYX AKT2 P2RX4 EIF2AK2 ARHGEF39 MAP3K2 RAB40B CARD19 CCL5 DGKZ CCR4 MAPK12 APP C5AR2 RCAN1 MAP3K9 SOX11 CCDC125 CNKSR3 GAS6 EMC10 USP28 TRIM25 IRAK4 CXCR4 NCOR1 GSK3B MSH6 RAB29 NMNAT1 SMCR8 CCR6 MYO5A ITGA3 ADCY2 STAC2 RAB22A CX3CL1 LTBR PRKAR2A CASP8 RAB3B TBCK SMAD2 GPR155 CSF2RA MKNK1 MDM4 F2R TNK2 FARP1 SEZ6 CHEK2 TAOK1	0.1234	0.3098	1.2
GO:0005088	Ras guanyl-nucleotide exchange factor activity	molecular_function	5/398	153/22360	DENND2A FARP1 ARHGEF39 FGD5 SMCR8	0.1235	0.3099	1.84
GO:0016209	antioxidant activity	molecular_function	4/398	116/22360	DUOX1 NXN HBE1 HP	0.1241	0.3112	1.94
GO:0050848	regulation of calcium-mediated signaling	biological_process	4/398	116/22360	P2RX4 MYO5A GSK3B RCAN1	0.1241	0.3112	1.94
GO:0048259	regulation of receptor-mediated endocytosis	biological_process	4/398	116/22360	LRPAP1 APP AAK1 TNK2	0.1241	0.3112	1.94
GO:0051092	positive regulation of NF-kappaB transcription factor activity	biological_process	6/398	192/22360	CX3CL1 EIF2AK2 APP NLRP3 TRIM25 IRAK4	0.1242	0.311	1.76
GO:0007093	mitotic cell cycle checkpoint	biological_process	6/398	193/22360	MDM4 MSH6 CHEK2 USP44 TAOK1 TP53	0.1268	0.3163	1.75
GO:0006163	purine nucleotide metabolic process	biological_process	12/398	446/22360	COX5B PGM2L1 ACSL6 SLC26A2 NCOR1 GPAT4 PDP2 ACOT8 ADCY2 MLXIPL PDE4C SULT1B1	0.1268	0.3163	1.51
GO:0030148	sphingolipid biosynthetic process	biological_process	4/398	117/22360	PLPP2 B3GALT1 A4GALT KDSR	0.1276	0.318	1.92
GO:0001824	blastocyst development	biological_process	4/398	117/22360	SLC35E2B POU5F1 TAF8 RPL7L1	0.1276	0.318	1.92
GO:0006468	protein phosphorylation	biological_process	48/398	2175/22360	EIF2AK2 CX3CL1 AKT2 STYX MAP3K2 LTBR PEAK1 YES1 ADCY2 PSMD9 MLXIPL TIE1 SYAP1 PIK3R3 PGF AAK1 SPTAN1 MAPKAPK5 NMNAT1 SCYL3 TP53 SMCR8 CHEK2 GSK3B NCOR1 TAOK1 GAS6 SRCIN1 TRAF3IP1 CNKSR3 CXCR4 IRAK4 TNK2 F2R EMC10 APP GRK5 C5AR2 SMAD2 CSF2RA MKNK1 MAPK12 CAMK1 MAP3K9 PRKAR2A CCL5 ADAR TBCK	0.128	0.3187	1.24
GO:0032147	activation of protein kinase activity	biological_process	11/398	403/22360	MAPKAPK5 MAP3K2 MAP3K9 TAOK1 EIF2AK2 F2R CXCR4 ADCY2 GAS6 PRKAR2A CCL5	0.1283	0.3193	1.53
GO:0098655	cation transmembrane transport	biological_process	25/398	1048/22360	ATP6V1A SLC12A2 KCNJ15 ABCC9 SLC9A4 CTSS TTYH1 SLC30A6 STEAP2 MYO5A APP COX5B SLC33A1 CACNB2 SLC39A13 ANO6 GAS6 CNKSR3 STAC2 F2R CX3CL1 KCNN3 P2RX4 ATP2B1 SLC9A7	0.1287	0.3201	1.34
GO:0006643	membrane lipid metabolic process	biological_process	7/398	234/22360	A4GALT PIGL KDSR PLPP2 B3GALT1 ESYT3 SUMF2	0.1289	0.3205	1.68
GO:0098794	postsynapse	cellular_component	17/398	673/22360	BAALC TENM2 F2R YES1 SRCIN1 GSK3B GABRB3 P2RX4 SEZ6 FARP1 ADD2 DGKZ TANC2 SYAP1 APP MYO5A CAMK1	0.1291	0.3204	1.42
GO:0035148	tube formation	biological_process	5/398	155/22360	RARG SOX11 WNT4 CC2D2A BCAS3	0.1296	0.3215	1.81
GO:0016310	phosphorylation	biological_process	57/398	2631/22360	GAS6 CNKSR3 IRAK4 CXCR4 EMC10 GSK3B NCOR1 DGKZ NAGK CCL5 NDUFA10 C5AR2 APP MAPK12 MAP3K9 GNPTG GK5 MLXIPL PSMD9 TIE1 PGM2L1 EIF2AK2 AKT2 STYX MAP3K2 PEAK1 PDE12 TP53 SHPK SYAP1 PIK3R3 SPTAN1 MAPKAPK5 SRCIN1 TRAF3IP1 TNK2 F2R CHEK2 TAOK1 PRKAR2A ADAR TBCK GRK5 COX5B MKNK1 CSF2RA SMAD2 CAMK1 YES1 ADCY2 CX3CL1 LTBR NMNAT1 SCYL3 SMCR8 AAK1 PGF	0.1305	0.3235	1.22
GO:0071887	leukocyte apoptotic process	biological_process	4/398	118/22360	CCL5 TP53 CD3G GAS6	0.1312	0.3193	1.9

GO:0008324	cation transmembrane transporter activity	molecular_function	22/398	908/22360	SLC39A13 ANO6 SLC33A1 CACNB2 TTYH1 SLC30A6 MYO5A COX5B APP ABCC9 KCNJ15 ATP6V1A SLC12A2 CTSS SLC9A4 SLC9A7 KCNN3 ATP2B1 P2RX4 STAC2 CNKSR3 GAS6	0.1314	0.3196	1.36
GO:0061024	membrane organization	biological_process	22/398	908/22360	STX16 ANO6 AAK1 TRIM72 A4GALT SYP CASP8 TP53 CD3G GSK3B RFTN1 AKT2 WHAMM SLC35F6 XKR4 TMEM170A TIE1 UBXN2A EPN1 CXCR4 RILPL1 GAS6	0.1314	0.3196	1.36
GO:0019693	ribose phosphate metabolic process	biological_process	12/398	449/22360	MLXIPL SULT1B1 PDE4C ADCY2 ACOT8 GPAT4 NCOR1 PDP2 ACSL6 PGM2L1 COX5B SLC26A2	0.132	0.3188	1.5
GO:1903169	regulation of calcium ion transmembrane transport	biological_process	5/398	156/22360	CX3CL1 MYO5A STAC2 CACNB2 F2R	0.1326	0.3201	1.8
GO:0019787	ubiquitin-like protein transferase activity	molecular_function	13/398	494/22360	UBE3C TRIM72 XIAP DCUN1D2 HERC4 RNF7 FBXL18 FBXL4 USP44 TTC3 RNF115 SUMO2 TRIM25	0.1329	0.3207	1.48
GO:0048037	cofactor binding	molecular_function	14/398	539/22360	COQ6 PYGB OGFOD1 H6PD CRYZL1 CYP4V2 NUBPL CYP51A1 DUOX1 HBE1 GCLC CYB5R4 ACOX1 HIF1AN	0.133	0.3208	1.46
GO:0005623	cell	cellular_component	372/398	19337/22360	PSTPIP2 STYX UBE3C EYA3 C1ORF61 BAALC SBNO2 TMTC1 SCAI SYAP1 MED17 CACNB2 SEC62 ABCC10 QPRT B3GALT1 DENND2A CIART PDP2 CORO7 ZNF441 RCAN1 ARFRP1 CCR4 PLEKHB2 WHAMM ZNF274 LTBR SLC37A2 ZNF445 CCR6 AAK1 BTN3A2 GSDMA ARNT2 TMEM187 RFTN1 ZNF789 MDM4 USP44 ZNF573 CS ZNF345 DUOX1 DCTD TBCCD1 ASPA ATP2B1 TCF4 GK5 FBXL4 NPAT ABCB5 TRIM72 ZNF814 BCAS3 ZDHHC15 OR7D2 RIPPLY3 TTLL3 CNKSR3 GAS6 DESI1 USP28 H6PD COQ6 MTFMT TRABD2B IFNAR1 DPP9 RGS17 ZMYM5 C1ORF174 STAC2 PLEKHA2 WNT4 IKZF3 CD3G FARP1 C1ORF210 CRX CSF2RA A4GALT ANO6 GJC1 MLX SNORA13 DIS3L ZHX3 VPS33A RAB3B DDX51 MTAP ZNF264 MAP3K2 PCDHB9 TTI2 NUDT22 OGFOD1 TONSL TMOD3 MAPKAPK5 CALCRL ABCC9 LIG1 TP53 RGS12 NCOR1 RABGAP1 GSK3B SOX11 ZNF483 HERC4 RNF7 ZNF772 CCL5 PIGR ZNF669 OTUD4 RAB22A SLC35E1 PCSK7 YES1 ZFPM2 RRP1 SLC26A2 MYO5A APOBEC3D NMNAT1 SMCR8 PTPRM ATXN3 SLC35F6 SAMD4B SMAD2 LETMD1 COX5B NSRP1 COG1 CASP8 NDUFAF7 DHX30 TOR1AIP2 ZNF677 AKT2 MED22 GALNT15 ZC3H14 PYGB TSEN2 SLC25A26 CCDC36 XIAP EXOSC6 CYB5R4 CWC25 NLRP3 SHPK CEP112 KIAA1191 C9ORF84 SUMF2 CLCC1 CXCR4 C5AR2 DGKZ FITM2 KLHL1 RILPL1 SON GMEB1 AS3MT SPC25 FOXD2 SMARCC1 AP4E1 ITGA3 POU5F1 PKNOX1 MSH6 COL8A1 RAB29 ABCC3 ZNF583 ACOX1 HP DCUN1D2 SRCIN1 TENM2 TTC3 LRRC8B ADAR PRKAR2A CYB561A3 ZNF766 SLFN13 PGM2L1 VPS28 PEAK1 ZNF7 XKR4 FGD5 FAM161A PSMD9 CYP4V2 PIK3R3 SLC35E2B MYO1C DRG1 METTL21A ERMAP FDPS DYNC2LI1 CCDC125 TRIM25 NXN RPRD1B NAGK DNAL1 RNF115 KCNN3 ADCY2 INTS6 ISG20L2 GNS TANC2 ZNF430 SEZ6 TTLL7 N4BP2L2 SPN CNR2 HMCN2 IBA57 STEAP2 PRR11 KCNJ15 NOA1 CRYZL1 HNRNPUL1 EIF2AK2 SRSF10 VPS52 ELMOD1 RAB40B CARD19 UBXN2A TIE1 ZNF106 CNBD2 ICOSLG PPM1K SLC25A44 R3HCC1 TMEM170A IRAK4 MGLL SPATS2 ZKSCAN1 PCDH9 DDX54 SLC9A4 RPL7L1 PLPP2 SNX27 C19ORF66 RSPH3 TAF8 PNPT1 KRT18 CC2D2A DDI2 PIGL PDE4C ZNF80 KDSR GSDMB BRDT ZNF286B MARVELD2 RARG GALNT16 SRGAP2B ESYT3 TRAF3IP1 F2R CAMK1 SLC30A6 TMEM106B TTYH1 FTO STX16 RRP12 TBCK TMEM266 ZNF570 AP4M1 LRTOMT C2ORF49 APOL1 GPAT4 PDE12 VSIG1 ATP6V1A ZBTB37 GCLC ACSL6 KDELC2 EMC10 APP NDUFA10 BTNL9 ACOT8 ODF2L TRMT10B ZNF70 LRPAP1 BBS1 KLHL8 CTSS WBP2 FBXL18 COX6B2 ICA1L MCM3AP SCYL3 KMT2D CHEK2 TAOK1 MKNK1 SULT1B1 SUMO2 ADD2 NUBPL FHDC1 P2RX4 VNN2 ARHGEF39 MLXIPL	0.1336	0.3213	1.08

					SPTAN1 SLC33A1 RFC2 SYT12 MXD1 ZNF8 CYP51A1 C9ORF78 SLC9A7 FCF1 EPN1 PCGF5 MAPK12 SSTR2 SNX21 ZNF800 GNPTG SLC12A2 CX3CL1 GABRB3 OAS3 SYP SLC39A13 HIF1AN HBE1 MNT TNK2 NAP1L6 SYNRG MR1 GRK5 MMS22L LGMN			
GO:0017158	regulation of calcium ion-dependent exocytosis	biological_process	4/398	119/22360	GSK3B SYP SYT12 CACNB2	0.1349	0.3243	1.89
GO:2001237	negative regulation of extrinsic apoptotic signaling pathway	biological_process	4/398	119/22360	CX3CL1 CASP8 EYA3 GCLC	0.1349	0.3243	1.89
GO:0098693	regulation of synaptic vesicle cycle	biological_process	4/398	119/22360	CACNB2 RAB3B GSK3B SYP	0.1349	0.3243	1.89
GO:0060078	regulation of postsynaptic membrane potential	biological_process	5/398	157/22360	SEZ6 P2RX4 APP GABRB3 GSK3B	0.1358	0.326	1.79
GO:0009056	catabolic process	biological_process	61/398	2843/22360	RNF115 NAGK ACOT8 MGLL QPRT TRIM25 USP28 RNF7 GSK3B NCOR1 GCLC CYP51A1 TP53 PPM1K ATP6V1A PDE12 EXOSC6 TRIM72 MLXIPL PSMD9 FBXL4 PYGB UBXN2A LRTOMT ZC3H14 GK5 VPS28 PGM2L1 UBE3C MTAP ASPA STYX AKT2 SLFN13 LGMN SUMO2 SULT1B1 CASP8 VPS33A DIS3L DUOX1 SAMD4B FTO USP44 TTC3 MDM4 ATXN3 HP ACOX1 CHEK2 HBE1 SMCR8 NMNAT1 CTSS FBXL18 KLHL8 GNS SMARCC1 APOBEC3D PDE4C PNPT1	0.1368	0.3283	1.21
GO:0055082	cellular chemical homeostasis	biological_process	21/398	866/22360	FITM2 CCR4 ATP6V1A CCL5 VPS33A SLC9A4 SLC39A13 CACNB2 MYO5A C5AR2 CCR6 APP F2R MLXIPL CXCR4 GAS6 SLC9A7 CX3CL1 GCLC ATP2B1 P2RX4	0.1378	0.329	1.36
GO:0002221	pattern recognition receptor signaling pathway	biological_process	7/398	238/22360	RFTN1 IRAK4 LGMN CASP8 CTSS XIAP OTUD4	0.1389	0.3312	1.65
GO:0016579	protein deubiquitination	biological_process	9/398	322/22360	SMAD2 OTUD4 ATXN3 MDM4 NLRP3 USP28 USP44 PSMD9 TP53	0.1389	0.3312	1.57
GO:0006289	nucleotide-excision repair	biological_process	4/398	121/22360	RFC2 ATXN3 LIG1 TP53	0.1423	0.3343	1.86
GO:0006637	acyl-CoA metabolic process	biological_process	4/398	121/22360	ACOT8 PDP2 ACSL6 GPAT4	0.1423	0.3343	1.86
GO:0051341	regulation of oxidoreductase activity	biological_process	4/398	121/22360	CNR2 PDP2 PPM1K HP	0.1423	0.3343	1.86
GO:0042116	macrophage activation	biological_process	4/398	121/22360	APP SHPK CX3CL1 SBNO2	0.1423	0.3343	1.86
GO:0019216	regulation of lipid metabolic process	biological_process	11/398	411/22360	NCOR1 ACOX1 PDP2 PIK3R3 CYP51A1 AKT2 WNT4 MLXIPL FITM2 DGKZ FDPS	0.1432	0.3354	1.5
GO:0043123	positive regulation of I-kappaB kinase/NF-kappaB signaling	biological_process	6/398	199/22360	CX3CL1 TRIM25 LTBR IRAK4 F2R CASP8	0.1435	0.3359	1.69
GO:0007254	JNK cascade	biological_process	7/398	240/22360	MAP3K9 MAP3K2 IRAK4 TAOK1 LTBR NCOR1 APP	0.144	0.3356	1.64
GO:0042623	ATPase activity, coupled	molecular_function	12/398	456/22360	DDX54 MYO1C ATP2B1 ABCB5 ABCC3 DHX30 RFC2 DYNC2LI1 ABCC9 ABCC10 ATP6V1A MSH6	0.1445	0.3366	1.48
GO:0046873	metal ion transmembrane transporter activity	molecular_function	16/398	638/22360	MYO5A TTYH1 SLC30A6 CACNB2 SLC39A13 SLC9A4 SLC12A2 KCNJ15 ABCC9 P2RX4 ATP2B1 KCNN3 SLC9A7 GAS6 CNKSR3 STAC2	0.1452	0.3381	1.41
GO:0015078	proton transmembrane transporter activity	molecular_function	5/398	160/22360	COX5B SLC9A4 ATP6V1A SLC9A7 SLC33A1	0.1454	0.3384	1.76
GO:0008277	regulation of G protein-coupled receptor signaling pathway	biological_process	5/398	160/22360	MGLL RGS12 SYP CCL5 GRK5	0.1454	0.3384	1.76
GO:0008630	intrinsic apoptotic signaling pathway in response to DNA	biological_process	4/398	122/22360	CHEK2 TP53 MSH6 USP28	0.1461	0.3397	1.84

	damage							
GO:0042594	response to starvation	biological_process	6/398	200/22360	GAS6 EIF2AK2 SSTR2 WNT4 BCAS3 TP53	0.1464	0.3403	1.69
GO:0023057	negative regulation of signaling	biological_process	35/398	1551/22360	LGMN RNF115 CASP8 CCL5 RCAN1 NXN SMAD2 EPN1 SLC35F6 TRAF3IP1 CNKSR3 GAS6 MNT CCDC125 CNR2 RABGAP1 NCOR1 GSK3B GCLC RGS12 NLRP3 ITGA3 HIF1AN MAPKAPK5 SCAI TRIM72 WNT4 PSMD9 LRPAP1 CARD19 EYA3 RGS17 OTUD4 TRABD2B CX3CL1	0.1465	0.3403	1.27
GO:0016323	basolateral plasma membrane	cellular_component	7/398	241/22360	SLC9A4 ATP2B1 C5AR2 MARVELD2 VSIG1 ITGA3 MYO1C	0.1467	0.3407	1.63
GO:0060271	cilium assembly	biological_process	11/398	413/22360	BBS1 FAM161A RILPL1 DYNC2LI1 TRAF3IP1 DNAL1 ODF2L CC2D2A FHDC1 RABGAP1 TTLL3	0.1471	0.3414	1.5
GO:0006611	protein export from nucleus	biological_process	6/398	201/22360	MCM3AP GSK3B TP53 CAMK1 STYX GAS6	0.1493	0.3459	1.68
GO:0046907	intracellular transport	biological_process	44/398	2002/22360	RAB3B TBCK SNX27 ACOT8 ARFRP1 VPS33A COG1 STX16 CORO7 RABGAP1 CAMK1 SYNRG APP COX5B DENND2A DYNC2LI1 GAS6 TRAF3IP1 ACOX1 SEC62 GSK3B RFTN1 MCM3AP TP53 SYT12 RAB29 SPTAN1 CACNB2 MYO1C AP4E1 ZDHHC15 BCAS3 SYP MYO5A RILPL1 AP4M1 SRSF10 VPS28 VPS52 RAB40B RAB22A STYX AKT2 WHAMM	0.1498	0.3469	1.23
GO:0007173	epidermal growth factor receptor signaling pathway	biological_process	4/398	123/22360	EPN1 APP PIGR RNF115	0.15	0.3461	1.83
GO:0016477	cell migration	biological_process	37/398	1654/22360	CCR4 TBCCD1 LGMN ADD2 CCL5 DGKZ SLC12A2 ANO6 C5AR2 APP CXCR4 IRAK4 F2R EMC10 SRCIN1 GAS6 CNR2 SPN PTPRM SCYL3 SRGAP2B BCAS3 PGF ITGA3 MYO1C SCAI CCR6 PIK3R3 WNT4 TIE1 YES1 ARHGEF39 PEAK1 P2RX4 AKT2 PSTPIP2 CX3CL1	0.1505	0.3471	1.26
GO:0043488	regulation of mRNA stability	biological_process	6/398	202/22360	EXOSC6 PSMD9 SAMD4B PDE12 FTO ZC3H14	0.1523	0.3451	1.67
GO:0005788	endoplasmic reticulum lumen	cellular_component	9/398	328/22360	APOL1 APP H6PD WNT4 SUMF2 LRPAP1 KDELC2 GAS6 COL8A1	0.1523	0.3451	1.54
GO:0031328	positive regulation of cellular biosynthetic process	biological_process	46/398	2106/22360	WNT4 PSMD9 MLXIPL GMEB1 NPAT SBNO2 ZFPM2 C1ORF61 YES1 MAP3K2 TCF4 TAF8 P2RX4 CX3CL1 AKT2 TP53 NLRP3 WBP2 PKNOX1 IKZF3 POU5F1 RFC2 BCAS3 MED17 MAPKAPK5 RARG FOXD2 SMARCC1 BRDT CRX PCGF5 ZNF345 F2R MNT SOX11 SPN CHEK2 KMT2D ARNT2 SUMO2 CCL5 RPRD1B MLX APP SMAD2 CAMK1	0.153	0.3464	1.23
GO:0048661	positive regulation of smooth muscle cell proliferation	biological_process	4/398	124/22360	CCL5 CALCRL IRAK4 CX3CL1	0.1539	0.3483	1.81
GO:0101005	ubiquitinyl hydrolase activity	molecular_function	4/398	124/22360	ATXN3 OTUD4 USP44 USP28	0.1539	0.3483	1.81
GO:0036459	thiol-dependent ubiquitinyl hydrolase activity	molecular_function	4/398	124/22360	USP28 USP44 OTUD4 ATXN3	0.1539	0.3483	1.81
GO:0019899	enzyme binding	molecular_function	52/398	2410/22360	FGD5 ARHGEF39 VPS52 MAP3K2 HNRNPUL1 TP53 METTL21A RFC2 BCAS3 MAPKAPK5 MYO1C TRIM72 CXCR4 GAS6 NCOR1 GSK3B RPRD1B SLC12A2 APP DENND2A LRPAP1 YES1 ADCY2 RILPL1 LTBR WHAMM SMCR8 RAB29 SRGAP2B POU5F1 MSH6 ITGA3 MYO5A TNK2 SRCIN1 MDM4 N4BP2L2 ATXN3 DCUN1D2 CHEK2 FARP1 TOR1AIP2 SUMO2 TBCK ADD2 CASP8 NDUFAF7 PRKAR2A DIS3L GRK5 SMAD2 RABGAP1	0.1541	0.3483	1.21
GO:0005783	endoplasmic reticulum	cellular_component	47/398	2158/22360	GAS6 KDELC2 CLCC1 TMEM170A TENM2 SUMF2 EMC10 MGLL H6PD B3GALT1 LRRC8B SEZ6 CYP51A1 ACSL6 SEC62 ATXN3 FITM2 TOR1AIP2 MR1 DUOX1 TTYH1 APP GJC1 PIGL SLC37A2 LRTOMT LRPAP1 UBXN2A WNT4 KDSR FGD5 TMTC1 PNPT1 CARD19 NLRP3 COL8A1 ESYT3 TP53 CYP4V2 MYO5A SLC33A1 CYB5R4 APOL1 SLC39A13 ZDHHC15 CALCRL GPAT4	0.1544	0.3488	1.22
GO:0046330	positive regulation of JNK cascade	biological_process	5/398	163/22360	MAP3K2 MAP3K9 LTBR TAOK1 APP	0.1555	0.3511	1.72

GO:0051480	regulation of cytosolic calcium ion concentration	biological_process	10/398	374/22360	F2R CCR4 CXCR4 CX3CL1 P2RX4 ATP2B1 CCR6 C5AR2 MYO5A CACNB2	0.1577	0.3547	1.5
GO:1901800	positive regulation of proteasomal protein catabolic process	biological_process	4/398	125/22360	GSK3B ATXN3 SUMO2 GCLC	0.1579	0.355	1.8
GO:0016079	synaptic vesicle exocytosis	biological_process	4/398	125/22360	SYT12 CACNB2 GSK3B SYP	0.1579	0.355	1.8
GO:0098869	cellular oxidant detoxification	biological_process	4/398	125/22360	DUOX1 NXN HBE1 HP	0.1579	0.355	1.8
GO:0047485	protein N-terminus binding	molecular_function	4/398	125/22360	ACOX1 SMARCC1 TP53 NPAT	0.1579	0.355	1.8
GO:0030027	lamellipodium	cellular_component	6/398	204/22360	SRCIN1 DGKZ APP SCYL3 FGD5 PTPRM	0.1583	0.3553	1.65
GO:0009108	coenzyme biosynthetic process	biological_process	5/398	164/22360	COQ6 NMNAT1 ACSL6 QPRT PDP2	0.1589	0.3565	1.71
GO:0051048	negative regulation of secretion	biological_process	8/398	288/22360	F2R PSMD9 CYP51A1 CX3CL1 C5AR2 SRCIN1 NLRP3 GAS6	0.1594	0.3575	1.56
GO:0048878	chemical homeostasis	biological_process	29/398	1269/22360	GAS6 CXCR4 F2R GCLC SLC9A7 RABGAP1 ACOX1 SLC9A4 PRKAR2A VPS33A CCL5 SLC12A2 CCR4 FITM2 STEAP2 C5AR2 APP ADCY2 MLXIPL ATP2B1 P2RX4 CX3CL1 ATP6V1A XIAP MYO5A CCR6 CYB5R4 CACNB2 SLC39A13	0.1598	0.3582	1.28
GO:0007264	small GTPase mediated signal transduction	biological_process	15/398	601/22360	ARHGEF39 RAB40B FARP1 RAB22A USP28 F2R TNK2 FGD5 CCDC125 MAPKAPK5 ITGA3 SCAI RAB3B TP53 RAB29	0.16	0.3585	1.4
GO:0006897	endocytosis	biological_process	15/398	601/22360	LRPAP1 TNK2 XKR4 EPN1 RAB22A VPS28 HP SYNRG STEAP2 SYP APP ANO6 APOL1 CALCRL AAK1	0.16	0.3585	1.4
GO:0016197	endosomal transport	biological_process	7/398	246/22360	STX16 VPS28 SNX27 VPS52 ARFRP1 RAB29 DENND2A	0.1602	0.3586	1.6
GO:0071805	potassium ion transmembrane transport	biological_process	7/398	246/22360	SLC9A4 ABCC9 SLC12A2 KCNN3 KCNJ15 SLC9A7 ANO6	0.1602	0.3586	1.6
GO:0003676	nucleic acid binding	molecular_function	97/398	4738/22360	ZBTB37 LIG1 TP53 ZNF7 ZNF264 SLFN13 ZNF570 APP RCAN1 ZNF441 ZNF772 CIART ZNF483 FDPS SOX11 TRIM25 NCOR1 WBP2 MCM3AP MYO5A APOBEC3D RRP1 PRR3 ISG20L2 ZNF445 ZNF70 ZFPM2 ZNF669 OTUD4 TRMT10B ZNF274 NOA1 DHX30 SUMO2 ZNF677 IBA57 SMAD2 SAMD4B NSRP1 CS ZNF345 ZNF573 KMT2D ZNF430 ARNT2 ZNF789 RFTN1 NLRP3 MXD1 RFC2 ZNF8 ZNF814 EXOSC6 ZC3H14 TSEN2 MLXIPL MIR654 ZNF106 TCF4 EIF2AK2 HNRNPUL1 SRSF10 RPL7L1 DDX54 ZKSCAN1 ZNF800 SPATS2 R3HCC1 C9ORF84 PCGF5 FCF1 PKNOX1 MSH6 IKZF3 ZNF839 POU5F1 RARG FOXD2 SMARCC1 ZNF286B OAS3 GMEB1 ZNF80 SON C19ORF66 KRT18 PNPT1 ADAR ZHX3 RRP12 DIS3L ZNF766 DDX51 MLX MNT CRX ZNF583	0.161	0.3601	1.15
GO:0016779	nucleotidyltransferase activity	molecular_function	6/398	205/22360	TP53 MAPKAPK5 PNPT1 NMNAT1 RFC2 OAS3	0.1614	0.3573	1.64
GO:0061136	regulation of proteasomal protein catabolic process	biological_process	6/398	205/22360	ATXN3 GSK3B SUMO2 STYX SMARCC1 GCLC	0.1614	0.3573	1.64
GO:0071674	mononuclear cell migration	biological_process	4/398	126/22360	ANO6 CX3CL1 CCL5 LGMN	0.1619	0.3578	1.78
GO:0004672	protein kinase activity	molecular_function	30/398	1321/22360	SMCR8 SYAP1 MAPKAPK5 AAK1 ADCY2 YES1 TIE1 AKT2 EIF2AK2 PEAK1 MAP3K2 ADAR CCL5 PRKAR2A TBCK CAMK1 MAPK12 MKNK1 GRK5 APP MAP3K9 SRCIN1 GAS6 F2R TNK2 IRAK4 CXCR4 CHEK2 TAOK1 GSK3B	0.1633	0.3601	1.28
GO:0055065	metal ion homeostasis	biological_process	17/398	697/22360	XIAP STEAP2 APP CCR6 C5AR2 MYO5A CACNB2 SLC39A13 CCL5 SLC12A2 ATP6V1A CCR4 P2RX4 ATP2B1 CX3CL1 CXCR4 F2R	0.165	0.3637	1.37

GO:0010628	positive regulation of gene expression	biological_process	46/398	2120/22360	ZNF345 F2R CRX PCGF5 SOX11 GAS6 GSK3B NCOR1 KMT2D ARNT2 CHEK2 SUMO2 RPRD1B CCL5 MLX SMAD2 CAMK1 APP SBNO2 ZFPM2 WNT4 PSMD9 MLXIPL GMEB1 NPAT C1ORF61 YES1 MAP3K2 CX3CL1 TCF4 TAF8 TP53 PKNOX1 IKZF3 POU5F1 NLRP3 WBP2 MED17 MYO1C ITGA3 BCAS3 BRDT RARG FOXD2 SMARCC1 PIK3R3	0.1653	0.3642	1.22
GO:0043197	dendritic spine	cellular_component	5/398	166/22360	TENM2 P2RX4 APP FARP1 SEZ6	0.1659	0.3654	1.69
GO:0032675	regulation of interleukin-6 production	biological_process	5/398	166/22360	F2R APP C5AR2 GAS6 CX3CL1	0.1659	0.3654	1.69
GO:0043009	chordate embryonic development	biological_process	16/398	651/22360	CC2D2A ARNT2 TAF8 ZFPM2 TIE1 EPN1 SOX11 TRAF3IP1 SLC35E2B SMAD2 RARG CASP8 RPL7L1 RIPPLY3 TANC2 POU5F1	0.1662	0.3657	1.38
GO:0007204	positive regulation of cytosolic calcium ion concentration	biological_process	9/398	334/22360	CACNB2 P2RX4 CCR6 MYO5A C5AR2 CX3CL1 CXCR4 CCR4 F2R	0.1664	0.366	1.51
GO:0030003	cellular cation homeostasis	biological_process	17/398	699/22360	CXCR4 F2R SLC9A7 P2RX4 ATP2B1 CX3CL1 CCR4 VPS33A CCL5 SLC9A4 ATP6V1A SLC39A13 CACNB2 APP CCR6 MYO5A C5AR2	0.1683	0.3699	1.37
GO:0004842	ubiquitin-protein transferase activity	molecular_function	12/398	469/22360	XIAP UBE3C TRIM72 DCUN1D2 RNF7 FBXL18 HERC4 TRIM25 FBXL4 USP44 TTC3 RNF115	0.1697	0.3722	1.44
GO:0009897	external side of plasma membrane	cellular_component	11/398	424/22360	CCR6 MR1 CSF2RA ITGA3 BTNL9 ICOSLG CD3G CCR4 CXCR4 ERMAP BTN3A2	0.1698	0.3722	1.46
GO:0001158	enhancer sequence-specific DNA binding	molecular_function	4/398	128/22360	SMARCC1 TP53 SOX11 CIART	0.1701	0.3727	1.76
GO:0046328	regulation of JNK cascade	biological_process	6/398	208/22360	APP LTBR NCOR1 TAOK1 MAP3K2 MAP3K9	0.1708	0.3741	1.62
GO:0016705	oxidoreductase activity, acting on paired donors, with incorporation or reduction of molecular oxygen	molecular_function	7/398	250/22360	CYP4V2 CNR2 CYP51A1 FTO COQ6 OGFOD1 HIF1AN	0.1715	0.3703	1.57
GO:0097191	extrinsic apoptotic signaling pathway	biological_process	7/398	250/22360	GCLC CX3CL1 EYA3 GSK3B KRT18 LTBR CASP8	0.1715	0.3703	1.57
GO:0005815	microtubule organizing center	cellular_component	19/398	796/22360	PCGF5 BBS1 TSEN2 FAM161A RILPL1 TTI2 DYNC2LI1 YES1 TRAF3IP1 ODF2L EYA3 GSK3B KRT18 CEP112 TBCCD1 DIS3L RPRD1B PRKAR2A RABGAP1	0.1724	0.3718	1.34
GO:1904951	positive regulation of establishment of protein localization	biological_process	13/398	516/22360	RAB29 NLRP3 GAS6 TP53 PSMD9 CASP8 F2R CAMK1 AKT2 BCAS3 GSK3B VPS28 MYO1C	0.1727	0.3723	1.42
GO:0045202	synapse	cellular_component	29/398	1280/22360	SRCIN1 F2R TENM2 RGS12 SEZ6 FARP1 CEP112 GSK3B DGKZ RAB3B ADD2 CAMK1 TTYH1 APP STX16 YES1 BAALC P2RX4 ATP2B1 RGS17 GABRB3 SYT12 TANC2 SYP SYAP1 MYO5A CACNB2 ITGA3 AAK1	0.1729	0.3725	1.27
GO:0009451	RNA modification	biological_process	5/398	168/22360	MTFMT TRMT10B FTO APOBEC3D ADAR	0.173	0.3726	1.67
GO:0044309	neuron spine	cellular_component	5/398	168/22360	APP P2RX4 FARP1 SEZ6 TENM2	0.173	0.3726	1.67
GO:0007569	cell aging	biological_process	5/398	168/22360	MNT MAPKAPK5 PNPT1 CHEK2 TP53	0.173	0.3726	1.67
GO:0034097	response to cytokine	biological_process	32/398	1428/22360	SHPK TP53 PDE12 MYO1C CCR6 RARG UBXN2A SBNO2 PSMD9 OAS3 PNPT1 LTBR KRT18 OTUD4 MTAP CX3CL1 EIF2AK2 IFNAR1 C19ORF66 CASP8 CCR4 SLC12A2 CCL5 ADAR DUOX1 CSF2RA TRIM25 CXCR4 IRAK4 GAS6 GSK3B GCLC	0.1731	0.3723	1.26
GO:1902903	regulation of supramolecular fiber organization	biological_process	10/398	381/22360	TAOK1 TMOD3 DRG1 MYO1C SPTAN1 WHAMM APP CX3CL1 ADD2 WNT4	0.1735	0.3731	1.47

GO:0050790	regulation of catalytic activity	biological_process	55/398	2586/22360	XIAP SYAP1 PIK3R3 BCAS3 MAPKAPK5 NLRP3 METTL21A PPM1K RFC2 TP53 EIF2AK2 AKT2 STYX ELMOD1 MAP3K2 FGD5 APP MAPK12 RCAN1 ITIH5 MAP3K9 PDP2 DGKZ CCL5 RGS12 GSK3B GAS6 CCDC125 CXCR4 MSH6 PPP1R37 SMCR8 CX3CL1 RGS17 ADCY2 OAS3 WNT4 RABGAP1 CAMK1 PAPLN PRKAR2A ADAR TBCK LGMN TOR1AIP2 CASP8 FARP1 DCUN1D2 HP TAOK1 SRCIN1 CNR2 TNK2 USP44 F2R	0.1736	0.3731	1.19
GO:0010595	positive regulation of endothelial cell migration	biological_process	4/398	129/22360	EMC10 LGMN P2RX4 BCAS3	0.1743	0.3745	1.74
GO:0019722	calcium-mediated signaling	biological_process	7/398	251/22360	MYO5A CCR6 P2RX4 CCR4 RCAN1 GSK3B CXCR4	0.1744	0.3745	1.57
GO:2001234	negative regulation of apoptotic signaling pathway	biological_process	7/398	251/22360	CX3CL1 MNT GCLC SLC35F6 CASP8 WNT4 EYA3	0.1744	0.3745	1.57
GO:0050852	T cell receptor signaling pathway	biological_process	8/398	294/22360	BTN3A2 ICOSLG BTNL9 PSMD9 ERMAP RFTN1 RAB29 CD3G	0.1751	0.3757	1.53
GO:0043410	positive regulation of MAPK cascade	biological_process	15/398	610/22360	APP C5AR2 MAP3K9 MAPKAPK5 CCL5 NMNAT1 EIF2AK2 CX3CL1 LTBR TAOK1 MAP3K2 GAS6 CXCR4 EMC10 F2R	0.1759	0.3773	1.38
GO:0070646	protein modification by small protein removal	biological_process	9/398	338/22360	ATXN3 OTUD4 SMAD2 TP53 PSMD9 USP28 USP44 NLRP3 MDM4	0.1762	0.377	1.5
GO:0006935	chemotaxis	biological_process	17/398	704/22360	ANO6 SPTAN1 PGF C5AR2 APP CCR6 CCR4 LGMN SLC12A2 CCL5 PTPRM SPN CX3CL1 P2RX4 CXCR4 CNR2 GAS6	0.1766	0.3777	1.36
GO:0050731	positive regulation of peptidyl-tyrosine phosphorylation	biological_process	6/398	210/22360	CCL5 SRCIN1 YES1 GAS6 TP53 TNK2	0.1772	0.3783	1.61
GO:0043122	regulation of I-kappaB kinase/NF-kappaB signaling	biological_process	7/398	252/22360	CX3CL1 TRIM25 LTBR CARD19 IRAK4 F2R CASP8	0.1773	0.3784	1.56
GO:0021782	glial cell development	biological_process	4/398	130/22360	ASPA SOX11 AKT2 APP	0.1785	0.3808	1.73
GO:0030833	regulation of actin filament polymerization	biological_process	5/398	170/22360	ADD2 MYO1C SPTAN1 TMOD3 WHAMM	0.1804	0.3847	1.65
GO:0005506	iron ion binding	molecular_function	5/398	170/22360	OGFOD1 HIF1AN FTO CYP51A1 CYP4V2	0.1804	0.3847	1.65
GO:0030029	actin filament-based process	biological_process	19/398	801/22360	FGD5 WNT4 PSTPIP2 FHDC1 CX3CL1 WHAMM KLHL1 TAOK1 ATXN3 TBCK ADD2 MYO5A TMOD3 GJC1 CACNB2 SPTAN1 MYO1C CORO7 BCAS3	0.1804	0.3847	1.33
GO:0044782	cilium organization	biological_process	11/398	429/22360	DNAL1 TRAF3IP1 DYNC2LI1 RILPL1 FAM161A BBS1 TTLL3 RABGAP1 FHDC1 CC2D2A ODF2L	0.1808	0.3851	1.44
GO:1990266	neutrophil migration	biological_process	4/398	131/22360	IRAK4 C5AR2 CCL5 CX3CL1	0.1828	0.3839	1.72
GO:0006304	DNA modification	biological_process	4/398	131/22360	APOBEC3D FTO EXOSC6 OTUD4	0.1828	0.3839	1.72
GO:0031301	integral component of organelle membrane	cellular_component	12/398	476/22360	SEC62 SLC39A13 STX16 P2RX4 A4GALT SYP STEAP2 TRABD2B EMC10 FITM2 PCSK7 SLC37A2	0.1844	0.387	1.42
GO:0015267	channel activity	molecular_function	17/398	710/22360	P2RX4 KCNN3 GABRB3 GAS6 LRRC8B STAC2 CLCC1 MYO5A APP TTYH1 GJC1 ANO6 APOL1 CACNB2 CTSS ABCC9 KCNJ15	0.187	0.3923	1.35
GO:0043487	regulation of RNA stability	biological_process	6/398	213/22360	EXOSC6 PSMD9 FTO SAMD4B PDE12 ZC3H14	0.1871	0.3923	1.58
GO:0051054	positive regulation of DNA metabolic process	biological_process	6/398	213/22360	OTUD4 RFC2 MSH6 MAPKAPK5 EXOSC6 EYA3	0.1871	0.3923	1.58
GO:0042176	regulation of protein catabolic process	biological_process	11/398	432/22360	STYX CYP51A1 SMARCC1 GCLC CHEK2 VPS28 GSK3B ATXN3 MDM4 UBXN2A SUMO2	0.1876	0.3924	1.43
GO:0060548	negative regulation of cell death	biological_process	27/398	1194/22360	TP53 LGMN CASP8 CCL5 NMNAT1 PDE12 GRK5 XIAP WNT4 ZFPM2 F2R SON SLC35F6 LRPAP1 GAS6 MNT SOX11 MDM4 KRT18 GABRB3 GSK3B EYA3 GCLC EIF2AK2 AKT2 CX3CL1 ARNT2	0.1885	0.3942	1.27

GO:0051222	positive regulation of protein transport	biological_process	12/398	478/22360	PSMD9 TP53 F2R GAS6 NLRP3 RAB29 GSK3B BCAS3 MYO1C VPS28 AKT2 CAMK1	0.1887	0.3944	1.41
GO:0006873	cellular ion homeostasis	biological_process	17/398	711/22360	F2R CXCR4 CX3CL1 ATP2B1 P2RX4 SLC9A7 ATP6V1A VPS33A CCL5 SLC9A4 CCR4 C5AR2 MYO5A CCR6 APP SLC39A13 CACNB2	0.1887	0.3944	1.34
GO:0022803	passive transmembrane transporter activity	molecular_function	17/398	712/22360	CTSS ABCC9 KCNJ15 MYO5A APP TTYH1 ANO6 GJC1 APOL1 CACNB2 GAS6 LRRC8B STAC2 CLCC1 P2RX4 KCNN3 GABRB3	0.1905	0.3969	1.34
GO:0001838	embryonic epithelial tube formation	biological_process	4/398	133/22360	WNT4 CC2D2A RARG SOX11	0.1916	0.399	1.69
GO:2001236	regulation of extrinsic apoptotic signaling pathway	biological_process	5/398	173/22360	CX3CL1 GCLC CASP8 EYA3 LTBR	0.1916	0.399	1.62
GO:0030258	lipid modification	biological_process	8/398	300/22360	PLPP2 B3GALT1 DGKZ ACOT8 ACOX1 PIK3R3 CYP4V2 AKT2	0.1916	0.399	1.5
GO:0031098	stress-activated protein kinase signaling cascade	biological_process	9/398	344/22360	EMC10 IRAK4 APP EIF2AK2 LTBR NCOR1 TAOK1 MAP3K9 MAP3K2	0.1916	0.399	1.47
GO:0043025	neuronal cell body	cellular_component	13/398	526/22360	SRCIN1 CNR2 CCR4 SYAP1 MYO5A P2RX4 APP ATP2B1 SEZ6 CX3CL1 KCNN3 KLHL1 TTLL7	0.1931	0.3966	1.39
GO:0051168	nuclear export	biological_process	6/398	215/22360	TP53 GSK3B MCM3AP GAS6 STYX CAMK1	0.1939	0.398	1.57
GO:0060491	regulation of cell projection assembly	biological_process	6/398	215/22360	RABGAP1 DYNC2LI1 P2RX4 TENM2 ODF2L BCAS3	0.1939	0.398	1.57
GO:0051345	positive regulation of hydrolase activity	biological_process	20/398	858/22360	WNT4 F2R CCDC125 GSK3B ELMOD1 RGS17 RGS12 CX3CL1 AKT2 LGMN TBCK TOR1AIP2 CASP8 NLRP3 CCL5 MSH6 BCAS3 APP MAPK12 RABGAP1	0.1952	0.4004	1.31
GO:0032635	interleukin-6 production	biological_process	5/398	174/22360	F2R GAS6 C5AR2 APP CX3CL1	0.1955	0.4008	1.61
GO:0019783	ubiquitin-like protein-specific protease activity	molecular_function	4/398	134/22360	ATXN3 USP44 USP28 OTUD4	0.196	0.4017	1.68
GO:0051246	regulation of protein metabolic process	biological_process	66/398	3179/22360	MAP3K2 VPS28 EIF2AK2 AKT2 STYX FAM161A MLXIPL UBXN2A OGFOD1 MAPKAPK5 PIK3R3 XIAP SYAP1 TP53 NLRP3 NCOR1 GSK3B GCLC CYP51A1 CXCR4 EMC10 CNKSR3 GAS6 RCAN1 ITIH5 MAP3K9 NXN APP C5AR2 MAPK12 CCL5 ACOT8 LTBR ZNF274 OTUD4 TRABD2B CX3CL1 YES1 ADCY2 PGF SMARCC1 SMCR8 PPP1R37 WBP2 NMNAT1 TAOK1 ATXN3 SPN DCUN1D2 CHEK2 F2R USP44 TNK2 TRAF3IP1 SRCIN1 MDM4 PAPLN SAMD4B CAMK1 FTO MKNK1 SUMO2 LGMN CASP8 ADAR PRKAR2A	0.197	0.4031	1.17
GO:0031324	negative regulation of cellular metabolic process	biological_process	60/398	2871/22360	ACOT8 NXN CIART ITIH5 DDX54 APP SOX11 GAS6 CNKSR3 GSK3B NCOR1 GCLC ZNF8 TP53 RIPPLY3 MXD1 PDE12 TONSL XIAP SCAI SBNO2 MLXIPL NPAT ZC3H14 VPS28 SRSF10 STYX EIF2AK2 PRKAR2A ADAR ZHX3 PAPLN MLX SMAD2 SAMD4B FTO ZNF345 TENM2 USP44 MDM4 MNT SRCIN1 TRAF3IP1 N4BP2L2 HP KMT2D FARP1 CHEK2 PPP1R37 SMCR8 MSH6 POU5F1 NMNAT1 HIF1AN RARG SMARCC1 ZFPM2 WNT4 LRPAP1 ZNF274	0.1985	0.4059	1.17
GO:0016887	ATPase activity	molecular_function	15/398	622/22360	ABCB5 DDX54 MYO1C METTL21A ABCC9 RFC2 ATP6V1A MSH6 TOR1AIP2 DHX30 ATP2B1 ABCC3 DYNC2LI1 ABCC10 C9ORF84	0.1987	0.4061	1.35
GO:0051100	negative regulation of binding	biological_process	5/398	175/22360	GSK3B LRPAP1 CAMK1 STYX SOX11	0.1994	0.4074	1.61
GO:0002757	immune response-activating signal transduction	biological_process	17/398	717/22360	MYO1C ICOSLG BTNL9 XIAP C5AR2 ERMAP LGMN BTN3A2 CASP8 CD3G RAB29 CTSS RFTN1 OTUD4 PSMD9 IRAK4 YES1	0.1995	0.4074	1.33

GO:0032268	regulation of cellular protein metabolic process	biological_process	62/398	2976/22360	NLRP3 TP53 SYAP1 XIAP PIK3R3 MAPKAPK5 OGFOD1 UBXN2A MLXIPL FAM161A STYX AKT2 EIF2AK2 VPS28 MAP3K2 CCL5 MAPK12 C5AR2 APP NXN MAP3K9 ITIH5 RCAN1 GAS6 CNKSR3 EMC10 CXCR4 GCLC GSK3B NCOR1 NMNAT1 WBP2 PPP1R37 SMCR8 SMARCC1 PGF ADCY2 YES1 CX3CL1 TRABD2B OTUD4 ZNF274 LTBR PRKAR2A ADAR CASP8 LGMN SUMO2 MKNK1 FTO CAMK1 SAMD4B PAPLN SRCIN1 TRAF3IP1 TNK2 F2R USP44 CHEK2 DCUN1D2 ATXN3 TAOK1	0.2001	0.4085	1.17
GO:0019637	organophosphate metabolic process	biological_process	25/398	1105/22360	FDPS QPRT H6PD ACSL6 NCOR1 ACOT8 DGKZ FITM2 SULT1B1 PLPP2 DCTD COX5B PDP2 PIGL GK5 ADCY2 PDE4C PLEKHA2 MLXIPL PGM2L1 NMNAT1 SHPK SLC26A2 PIK3R3 GPAT4	0.2014	0.411	1.27
GO:0006874	cellular calcium ion homeostasis	biological_process	12/398	484/22360	CCL5 CXCR4 CCR4 F2R ATP2B1 CCR6 P2RX4 APP MYO5A C5AR2 CX3CL1 CACNB2	0.2021	0.4123	1.39
GO:0009792	embryo development ending in birth or egg hatching	biological_process	16/398	671/22360	SOX11 TRAF3IP1 TIE1 ZFPM2 EPN1 ARNT2 TAF8 CC2D2A POU5F1 TANC2 CASP8 RIPPLY3 RPL7L1 SMAD2 RARG SLC35E2B	0.2022	0.4123	1.34
GO:0006820	anion transport	biological_process	16/398	671/22360	SLC12A2 SLC33A1 CYB5R4 APOL1 ANO6 SLC26A2 TTYH1 LRRC8B CLCC1 XKR4 SLC37A2 NCOR1 GABRB3 P2RX4 AKT2 ABCC3	0.2022	0.4123	1.34
GO:0044267	cellular protein metabolic process	biological_process	115/398	5753/22360	RNF115 CCL5 RPRD1B MAP3K9 RCAN1 PDP2 NXN APP TRIM25 B3GALT1 EMC10 RNF7 KDELC2 HERC4 NCOR1 GSK3B GCLC TP53 METTL21A MAPKAPK5 APOL1 DRG1 PIK3R3 SYAP1 FAM161A TMTC1 PSMD9 OGFOD1 EYA3 MAP3K2 VPS28 PEAK1 STYX UBE3C SUMO2 CASP8 NDUFAF7 NOA1 SAMD4B MKNK1 SMAD2 USP44 MDM4 TAOK1 TTLL7 ATXN3 PTPRM CHEK2 KMT2D SCYL3 SMCR8 PPP1R37 FBXL18 WBP2 CTSS NMNAT1 KLHL8 AAK1 YES1 ADCY2 LTBR ZNF274 OTUD4 PLPP2 MTFMT ITIH5 C5AR2 MAPK12 PCGF5 CXCR4 IRAK4 USP28 SUMF2 CNKSR3 GAS6 TTLL3 PPM1K NLRP3 ZDHHC15 SPTAN1 XIAP TRIM72 MLXIPL TIE1 UBXN2A FBXL4 RAB40B GALNT15 UEVLD EIF2AK2 AKT2 TBCK LGMN RAB3B ADAR PRKAR2A PAPLN GRK5 A4GALT FTO CAMK1 CSF2RA TTC3 F2R TNK2 TRAF3IP1 SRCIN1 DCUN1D2 PGF HIF1AN GALNT16 SMARCC1 PIGL TRABD2B CX3CL1	0.2036	0.4147	1.12
GO:0006493	protein O-linked glycosylation	biological_process	4/398	136/22360	TMTC1 KDELC2 GALNT15 GALNT16	0.2051	0.4114	1.65
GO:0033674	positive regulation of kinase activity	biological_process	15/398	626/22360	SYAP1 CAMK1 MAP3K9 MAPKAPK5 CCL5 PRKAR2A DGKZ EIF2AK2 TAOK1 MAP3K2 SRCIN1 GAS6 ADCY2 CXCR4 F2R	0.2066	0.4142	1.35
GO:0016125	sterol metabolic process	biological_process	5/398	177/22360	APOL1 CYP4V2 FDPS CYP51A1 APP	0.2073	0.4155	1.59
GO:2000058	regulation of ubiquitin-dependent protein catabolic process	biological_process	5/398	177/22360	SUMO2 GSK3B GCLC SMARCC1 STYX	0.2073	0.4155	1.59
GO:0070588	calcium ion transmembrane transport	biological_process	9/398	350/22360	STAC2 F2R GAS6 CACNB2 ANO6 CX3CL1 MYO5A ATP2B1 P2RX4	0.2078	0.4162	1.44
GO:0046578	regulation of Ras protein signal transduction	biological_process	7/398	262/22360	ITGA3 ARHGEF39 FGD5 F2R SCAI FARP1 CCDC125	0.2082	0.4168	1.5
GO:0016791	phosphatase activity	molecular_function	12/398	487/22360	PTPRM PDP2 RCAN1 GSK3B EYA3 FARP1 STYX EIF2AK2 PPP1R37 PLPP2 NT5DC3 PPM1K	0.209	0.4179	1.38
GO:0001508	action potential	biological_process	4/398	137/22360	CACNB2 GJC1 P2RX4 CNR2	0.2097	0.4192	1.64
GO:1903507	negative regulation of nucleic acid-templated transcription	biological_process	30/398	1358/22360	SMAD2 DDX54 MLX CIART ZHX3 GCLC KMT2D NCOR1 N4BP2L2 GAS6 MNT SOX11 MDM4 TENM2 ZNF345 SCAI RARG TONSL HIF1AN POU5F1 MXD1 ZNF8 TP53 RIPPLY3 ZNF274 NPAT WNT4 MLXIPL ZFPM2 SBNO2	0.2097	0.4192	1.24

GO:0061630	ubiquitin protein ligase activity	molecular_function	7/398	263/22360	RNF7 XIAP FBXL18 TRIM72 TRIM25 USP44 RNF115	0.2115	0.4219	1.5
GO:1902679	negative regulation of RNA biosynthetic process	biological_process	30/398	1360/22360	N4BP2L2 NCOR1 KMT2D GCLC ZNF345 TENM2 MDM4 MNT SOX11 GAS6 CIART MLX DDX54 SMAD2 ZHX3 ZNF274 SBNO2 ZFPM2 MLXIPL WNT4 NPAT HIF1AN TONSL RARG SCAI RIPPLY3 TP53 ZNF8 MXD1 POU5F1	0.2124	0.4236	1.24
GO:0070838	divalent metal ion transport	biological_process	13/398	536/22360	GAS6 CCL5 STAC2 F2R ATP2B1 P2RX4 MYO5A CX3CL1 SLC30A6 CALCRL CACNB2 SLC39A13 ANO6	0.2148	0.4282	1.36
GO:0048870	cell motility	biological_process	39/398	1814/22360	PTPRM SPN EMC10 F2R CXCR4 IRAK4 CCDC125 CNR2 GAS6 SRCIN1 ANO6 DUOX1 APP C5AR2 CCR4 TBCCD1 ADD2 LGMN SLC12A2 CCL5 DGKZ PEAK1 ARHGEF39 AKT2 PSTPIP2 CX3CL1 P2RX4 TIE1 WNT4 YES1 MYO1C PGF ITGA3 BCAS3 SCAI CCR6 PIK3R3 SCYL3 SRGAP2B	0.2149	0.4282	1.21
GO:0006310	DNA recombination	biological_process	8/398	308/22360	TONSL CCDC36 MMS22L CCR6 EXOSC6 C9ORF84 MSH6 LIG1	0.215	0.4283	1.46
GO:0045860	positive regulation of protein kinase activity	biological_process	14/398	583/22360	PRKAR2A CCL5 MAPKAPK5 MAP3K9 CAMK1 SYAP1 F2R CXCR4 ADCY2 SRCIN1 GAS6 MAP3K2 TAOK1 EIF2AK2	0.2152	0.4233	1.35
GO:0050662	coenzyme binding	molecular_function	8/398	309/22360	COQ6 PYGB OGFOD1 CRYZL1 H6PD DUOX1 GCLC ACOX1	0.2181	0.4288	1.45
GO:0051258	protein polymerization	biological_process	8/398	309/22360	ADD2 WHAMM PSTPIP2 CORO7 TMOD3 MYO1C SPTAN1 DRG1	0.2181	0.4288	1.45
GO:0001704	formation of primary germ layer	biological_process	4/398	139/22360	COL8A1 ITGA3 SMAD2 POU5F1	0.219	0.429	1.62
GO:0034329	cell junction assembly	biological_process	7/398	266/22360	MARVELD2 WHAMM WNT4 BCAS3 PEAK1 MYO1C GJC1	0.2214	0.4328	1.48
GO:0032984	protein-containing complex disassembly	biological_process	9/398	355/22360	VPS33A SMCR8 OGFOD1 ADD2 SMARCC1 TMOD3 SPTAN1 TAOK1 GSK3B	0.2219	0.4336	1.42
GO:0006812	cation transport	biological_process	29/398	1317/22360	KCNN3 CX3CL1 ATP2B1 P2RX4 STAC2 SLC39A13 CACNB2 SLC33A1 CALCRL MYO5A ABCC9 SYT12 ATP6V1A CTSS SLC9A7 F2R CNKSR3 GAS6 ANO6 SLC30A6 TTYH1 COX5B APP STEAP2 RAB3B KCNJ15 SLC12A2 CCL5 SLC9A4	0.222	0.4337	1.24
GO:0030001	metal ion transport	biological_process	23/398	1021/22360	SLC9A4 CCL5 ATP6V1A SLC12A2 KCNJ15 ABCC9 CALCRL CACNB2 SLC39A13 ANO6 STEAP2 MYO5A TTYH1 SLC30A6 STAC2 F2R GAS6 CNKSR3 SLC9A7 P2RX4 ATP2B1 CX3CL1 KCNN3	0.2233	0.4361	1.27
GO:0043066	negative regulation of apoptotic process	biological_process	23/398	1021/22360	WNT4 SON SLC35F6 F2R GAS6 MDM4 MNT EYA3 GSK3B KRT18 GABRB3 EIF2AK2 GCLC ARNT2 CX3CL1 AKT2 LGMN TP53 CASP8 NMNAT1 CCL5 XIAP GRK5	0.2233	0.4361	1.27
GO:0010468	regulation of gene expression	biological_process	109/398	5466/22360	ZFPM2 ZNF70 YES1 ZNF445 ZNF274 ZNF669 SMCR8 WBP2 ZNF345 ZNF573 MDM4 ZNF789 N4BP2L2 ARNT2 ZNF430 KMT2D CHEK2 ZNF677 SUMO2 NSRP1 MKNK1 SMAD2 SAMD4B SBNO2 OGFOD1 PSMD9 C1ORF61 ZNF7 ZNF570 MAP3K2 ZNF264 TP53 PDE12 ZBTB37 MYO1C MED17 MAPKAPK5 TONSL PIK3R3 TRIM25 ZNF483 SOX11 GSK3B NCOR1 GCLC RPRD1B CCL5 CIART RCAN1 ZNF441 ZNF772 APP SON ZNF80 WNT4 GMEB1 PNPT1 ZMYM5 CX3CL1 TAF8 IKZF3 PKNOX1 POU5F1 HIF1AN ITGA3 ZNF286B BRDT SMARCC1 FOXD2 RARG TENM2 F2R CRX MNT ZNF583 ZNF766 LGMN ADAR ZHX3 MLX FTO CAMK1 MIR654 UBXN2A MLXIPL NPAT ZC3H14 SRSF10 MED22 AKT2 TCF4 EIF2AK2 RIPPLY3 ZNF8 MXD1 OR7D2 NLRP3 SLC33A1 EXOSC6 BCAS3 XIAP ZNF814 PCGF5 IRAK4 GAS6 C9ORF78 ZNF800 ZKSCAN1 MAPK12	0.2236	0.4363	1.12
GO:0072175	epithelial tube formation	biological_process	4/398	140/22360	WNT4 CC2D2A SOX11 RARG	0.2238	0.4365	1.61
GO:0072511	divalent inorganic cation transport	biological_process	13/398	540/22360	SLC30A6 CX3CL1 ATP2B1 P2RX4 MYO5A SLC39A13 ANO6 CACNB2 CALCRL CCL5 GAS6 F2R STAC2	0.2239	0.4366	1.35
GO:0010952	positive regulation of peptidase activity	biological_process	6/398	224/22360	NLRP3 APP MAPK12 LGMN CASP8 F2R	0.226	0.4393	1.5
GO:0030100	regulation of endocytosis	biological_process	6/398	224/22360	AAK1 VPS28 ANO6 TNK2 APP LRPAP1	0.226	0.4393	1.5

GO:0016311	dephosphorylation	biological_process	13/398	541/22360	PPM1K NT5DC3 RPRD1B PLPP2 PPP1R37 EIF2AK2 STYX FARP1 EYA3 RCAN1 GSK3B PTPRM PDP2	0.2263	0.4396	1.35
GO:0030518	intracellular steroid hormone receptor signaling pathway	biological_process	4/398	141/22360	WBP2 KMT2D DDX54 NCOR1	0.2286	0.4398	1.59
GO:0043666	regulation of phosphoprotein phosphatase activity	biological_process	4/398	141/22360	PPP1R37 EIF2AK2 RCAN1 GSK3B	0.2286	0.4398	1.59
GO:0071383	cellular response to steroid hormone stimulus	biological_process	7/398	269/22360	NCOR1 DDX54 KMT2D SSTR2 RARG WBP2 ATP2B1	0.2315	0.445	1.46
GO:2001257	regulation of cation channel activity	biological_process	5/398	183/22360	CACNB2 STAC2 APP CTSS MYO5A	0.2319	0.4451	1.53
GO:0001726	ruffle	cellular_component	5/398	183/22360	FGD5 MYO1C MYO5A RAB22A AKT2	0.2319	0.4451	1.53
GO:0043204	perikaryon	cellular_component	4/398	142/22360	CNR2 SYAP1 TTLL7 APP	0.2334	0.4463	1.58
GO:1902904	negative regulation of supramolecular fiber organization	biological_process	4/398	142/22360	SPTAN1 TMOD3 ADD2 TAOK1	0.2334	0.4463	1.58
GO:0008202	steroid metabolic process	biological_process	9/398	359/22360	APP CYP51A1 CYP4V2 APOL1 FDPS ACOT8 LGMN WNT4 SULT1B1	0.2335	0.4462	1.41
GO:0001932	regulation of protein phosphorylation	biological_process	34/398	1576/22360	TAOK1 NCOR1 CHEK2 CXCR4 TNK2 EMC10 F2R SRCIN1 GAS6 CNKSR3 TRAF3IP1 MAP3K9 C5AR2 APP CAMK1 PRKAR2A CCL5 ADAR MAP3K2 LTBR EIF2AK2 CX3CL1 STYX AKT2 MLXIPL YES1 ADCY2 PGF MAPKAPK5 SYAP1 PIK3R3 TP53 SMCR8 NMNAT1	0.2335	0.4462	1.21
GO:0018108	peptidyl-tyrosine phosphorylation	biological_process	10/398	405/22360	CCL5 GAS6 SRCIN1 YES1 TIE1 TNK2 TP53 APP EIF2AK2 PEAK1	0.2346	0.448	1.39
GO:0001012	RNA polymerase II regulatory region DNA binding	molecular_function	19/398	832/22360	TP53 WBP2 ZHX3 IKZF3 MXD1 MLX CIART RARG SMARCC1 SMAD2 GMEB1 CRX PCGF5 MLXIPL MNT SOX11 NCOR1 TCF4 ARNT2	0.2346	0.448	1.28
GO:0006813	potassium ion transport	biological_process	7/398	270/22360	SLC9A7 ANO6 KCNN3 SLC9A4 ABCC9 SLC12A2 KCNJ15	0.2349	0.4482	1.46
GO:0055074	calcium ion homeostasis	biological_process	12/398	498/22360	CCL5 F2R CCR4 CXCR4 CX3CL1 MYO5A C5AR2 CCR6 ATP2B1 APP P2RX4 CACNB2	0.2355	0.4492	1.35
GO:1902600	proton transmembrane transport	biological_process	5/398	184/22360	SLC33A1 SLC9A7 ATP6V1A SLC9A4 COX5B	0.2361	0.4502	1.53
GO:0032956	regulation of actin cytoskeleton organization	biological_process	9/398	360/22360	WNT4 ADD2 WHAMM CX3CL1 TAOK1 BCAS3 MYO1C SPTAN1 TMOD3	0.2364	0.4506	1.4
GO:0008168	methyltransferase activity	molecular_function	6/398	227/22360	KMT2D AS3MT METTL21A LRTOMT NDUFAF7 TRMT10B	0.2373	0.4521	1.48
GO:0060627	regulation of vesicle-mediated transport	biological_process	14/398	593/22360	SYT12 RAB29 RAB3B SYP APP CACNB2 ANO6 AAK1 LRPAP1 GAS6 TNK2 AKT2 VPS28 GSK3B	0.2373	0.4521	1.33
GO:0042578	phosphoric ester hydrolase activity	molecular_function	15/398	642/22360	STYX FARP1 EIF2AK2 PTPRM EYA3 GSK3B PDE4C PDP2 RCAN1 NT5DC3 CCL5 PPM1K PRKAR2A PPP1R37 PLPP2	0.2404	0.4525	1.31
GO:0019207	kinase regulator activity	molecular_function	6/398	228/22360	GAS6 PRKAR2A CCL5 PIK3R3 TAOK1 SMCR8	0.2412	0.4525	1.48
GO:1902905	positive regulation of supramolecular fiber organization	biological_process	6/398	228/22360	WNT4 DRG1 MYO1C WHAMM APP CX3CL1	0.2412	0.4525	1.48
GO:0033993	response to lipid	biological_process	23/398	1032/22360	YES1 CNR2 ADCY2 TRIM25 F2R TIE1 SBNO2 IFNAR1 ATP2B1 MTAP ARNT2 CX3CL1 KMT2D NCOR1 WBP2 CCL5 NLRP3 CASP8 SHPK RARG SSTR2 SMAD2 DDX54	0.2419	0.4535	1.25
GO:0005975	carbohydrate metabolic process	biological_process	15/398	643/22360	NAGK SHPK GNPTG GK5 PYGB CS MLXIPL H6PD B3GALT1 AKT2 GCLC PGM2L1 UEVLD NCOR1 GSK3B	0.2427	0.4549	1.31

GO:1903052	positive regulation of proteolysis involved in cellular protein catabolic process	biological_process	4/398	144/22360	GCLC SUMO2 ATXN3 GSK3B	0.2433	0.4558	1.56
GO:0005796	Golgi lumen	cellular_component	4/398	144/22360	APP LRPAP1 WNT4 GAS6	0.2433	0.4558	1.56
GO:0034340	response to type I interferon	biological_process	4/398	144/22360	ADAR C19ORF66 IFNAR1 OAS3	0.2433	0.4558	1.56
GO:0046683	response to organophosphorus	biological_process	4/398	144/22360	PNPT1 APP P2RX4 DUOX1	0.2433	0.4558	1.56
GO:0016192	vesicle-mediated transport	biological_process	47/398	2247/22360	VPS33A COG1 RAB3B STEAP2 SYNRG STX16 ANO6 SRCIN1 TNK2 HP CD3G RAB29 CTSS GNS SYP MYO5A AAK1 AP4E1 YES1 LRPAP1 BBS1 WHAMM RAB22A KRT18 CCL5 ARFRP1 SNX27 PIGR DENND2A APP CORO7 GAS6 EPN1 GSK3B SYT12 TRIM72 CALCRL MYO1C SPTAN1 CACNB2 APOL1 AP4M1 XKR4 PYGB AKT2 VPS52 VPS28	0.2441	0.4563	1.18
GO:0008361	regulation of cell size	biological_process	5/398	186/22360	SLC12A2 RARG ANO6 GSK3B LRRC8B	0.2447	0.4573	1.51
GO:0045862	positive regulation of proteolysis	biological_process	10/398	409/22360	GSK3B ATXN3 MAPK12 GCLC APP CASP8 F2R SUMO2 LGMN NLRP3	0.2458	0.4592	1.37
GO:0071345	cellular response to cytokine stimulus	biological_process	29/398	1334/22360	OAS3 PSMD9 UBXN2A SBNO2 IFNAR1 OTUD4 MTAP CX3CL1 LTBR KRT18 PNPT1 PDE12 TP53 SHPK CCR6 RARG MYO1C GAS6 TRIM25 CXCR4 IRAK4 GSK3B CCL5 ADAR SLC12A2 CCR4 CASP8 DUOX1 CSF2RA	0.2472	0.4616	1.22
GO:0003677	DNA binding	molecular_function	57/398	2767/22360	ZNF430 KMT2D ARNT2 ZNF583 ZNF789 MNT ZNF345 ZNF573 CRX SMAD2 MLX ADAR ZHX3 ZNF766 ZNF677 ZNF669 ZNF274 ZNF70 ZNF445 ZNF80 SON ZFPM2 GMEB1 ZNF286B RARG SMARCC1 FOXD2 MSH6 PKNOX1 IKZF3 POU5F1 WBP2 NCOR1 C9ORF84 ZNF483 SOX11 PCGF5 APP ZKSCAN1 ZNF800 CIART RCAN1 ZNF441 ZNF772 ZNF264 TCF4 ZNF570 ZNF7 MLXIPL ZNF814 MXD1 RFC2 NLRP3 ZBTB37 LIG1 TP53 ZNF8	0.248	0.463	1.16
GO:0045598	regulation of fat cell differentiation	biological_process	4/398	145/22360	TAF8 SYAP1 FTO ZFPM2	0.2483	0.4633	1.55
GO:0006631	fatty acid metabolic process	biological_process	10/398	410/22360	MLXIPL MGLL ACOT8 PDP2 ACOX1 GPAT4 AKT2 CYP4V2 MYO5A ACSL6	0.2487	0.4633	1.37
GO:0032680	regulation of tumor necrosis factor production	biological_process	5/398	187/22360	CX3CL1 GAS6 C5AR2 APP SPN	0.2491	0.4638	1.5
GO:0051020	GTPase binding	molecular_function	14/398	599/22360	RILPL1 FGD5 FARP1 WHAMM ARHGEF39 VPS52 RAB29 SRGAP2B SMCR8 TBCK RABGAP1 DENND2A MYO5A MYO1C	0.2513	0.4633	1.31
GO:0050730	regulation of peptidyl-tyrosine phosphorylation	biological_process	7/398	275/22360	GAS6 SRCIN1 YES1 APP CCL5 TNK2 TP53	0.2525	0.4653	1.43
GO:0006364	rRNA processing	biological_process	6/398	231/22360	DDX51 RPL7L1 FCF1 EXOSC6 ISG20L2 RRP1	0.2528	0.4657	1.46
GO:0016485	protein processing	biological_process	6/398	231/22360	CTSS XIAP PCSK7 DDI2 LGMN CASP8	0.2528	0.4657	1.46
GO:0046034	ATP metabolic process	biological_process	8/398	320/22360	MLXIPL NMNAT1 PDE12 ATP6V1A NCOR1 COX5B NDUFA10 PGM2L1	0.253	0.4657	1.4
GO:0042383	sarcolemma	cellular_component	4/398	146/22360	ABCC9 TRIM72 CACNB2 STAC2	0.2533	0.4661	1.54
GO:0008033	tRNA processing	biological_process	4/398	146/22360	TSEN2 MTFMT TRMT10B C2ORF49	0.2533	0.4661	1.54
GO:0032640	tumor necrosis factor production	biological_process	5/398	188/22360	CX3CL1 GAS6 C5AR2 APP SPN	0.2535	0.4661	1.49
GO:0098542	defense response to other organism	biological_process	14/398	600/22360	TRIM25 OAS3 TRAF3IP1 HP SPN SLFN13 C19ORF66 EIF2AK2 PDE12 ABCC9 ADAR NLRP3 MR1 APOBEC3D	0.2536	0.4662	1.31
GO:1901215	negative regulation of neuron death	biological_process	7/398	276/22360	LGMN GSK3B GABRB3 F2R NMNAT1 GCLC CX3CL1	0.2561	0.4706	1.42
GO:0043523	regulation of neuron apoptotic process	biological_process	6/398	232/22360	F2R LGMN GABRB3 CX3CL1 NMNAT1 GCLC	0.2568	0.4712	1.45

GO:0030705	cytoskeleton-dependent intracellular transport	biological_process	5/398	189/22360	DYNC2LI1 MYO5A APP TRAF3IP1 MYO1C	0.2579	0.4731	1.49
GO:0051174	regulation of phosphorus metabolic process	biological_process	41/398	1951/22360	MAP3K9 RCAN1 PDP2 C5AR2 APP CAMK1 DGKZ PRKAR2A ADAR CCL5 GSK3B TAOK1 NCOR1 CHEK2 FARP1 CXCR4 TNK2 F2R EMC10 SRCIN1 GAS6 CNKSR3 TRAF3IP1 PGF MAPKAPK5 SYAP1 PIK3R3 TP53 PPP1R37 SMCR8 NMNAT1 PDE12 MAP3K2 LTBR EIF2AK2 CX3CL1 AKT2 STYX MLXIPL YES1 ADCY2	0.2587	0.4744	1.18
GO:0015079	potassium ion transmembrane transporter activity	molecular_function	6/398	233/22360	SLC9A7 KCNN3 KCNJ15 SLC12A2 ABCC9 SLC9A4	0.2608	0.478	1.45
GO:0008064	regulation of actin polymerization or depolymerization	biological_process	5/398	190/22360	WHAMM ADD2 MYO1C SPTAN1 TMOD3	0.2624	0.4759	1.48
GO:0004386	helicase activity	molecular_function	5/398	190/22360	DDX54 TP53 DHX30 DDX51 MSH6	0.2624	0.4759	1.48
GO:0030036	actin cytoskeleton organization	biological_process	16/398	700/22360	FGD5 WNT4 KLHL1 TAOK1 ATXN3 PSTPIP2 FHDC1 CX3CL1 WHAMM ADD2 TBCK TMOD3 MYO1C SPTAN1 CORO7 BCAS3	0.2625	0.4758	1.28
GO:0051924	regulation of calcium ion transport	biological_process	7/398	278/22360	CX3CL1 MYO5A CCL5 P2RX4 STAC2 CACNB2 F2R	0.2634	0.4772	1.41
GO:0030048	actin filament-based movement	biological_process	4/398	148/22360	CACNB2 MYO1C GJC1 MYO5A	0.2635	0.4772	1.52
GO:0007006	mitochondrial membrane organization	biological_process	4/398	148/22360	GSK3B TP53 SLC35F6 CASP8	0.2635	0.4772	1.52
GO:0015085	calcium ion transmembrane transporter activity	molecular_function	6/398	234/22360	MYO5A ATP2B1 P2RX4 GAS6 STAC2 CACNB2	0.2648	0.4782	1.44
GO:0044297	cell body	cellular_component	14/398	605/22360	CCR4 CASP8 APP MYO5A SYAP1 SRCIN1 CNR2 KLHL1 TTLL7 P2RX4 ATP2B1 KCNN3 SEZ6 CX3CL1	0.2656	0.4795	1.3
GO:0002764	immune response-regulating signaling pathway	biological_process	18/398	799/22360	IRAK4 PSMD9 YES1 RFTN1 OTUD4 CASP8 BTN3A2 PIGR LGMN ERMAP CTSS CD3G RAB29 BTNL9 MYO1C ICOSLG C5AR2 XIAP	0.2661	0.4803	1.27
GO:0030832	regulation of actin filament length	biological_process	5/398	191/22360	ADD2 SPTAN1 MYO1C TMOD3 WHAMM	0.2669	0.4815	1.47
GO:0110053	regulation of actin filament organization	biological_process	7/398	279/22360	ADD2 WNT4 TMOD3 MYO1C SPTAN1 CX3CL1 WHAMM	0.2671	0.4817	1.41
GO:0006090	pyruvate metabolic process	biological_process	4/398	149/22360	NCOR1 MLXIPL PDP2 PGM2L1	0.2687	0.4839	1.51
GO:0051494	negative regulation of cytoskeleton organization	biological_process	4/398	149/22360	SPTAN1 TMOD3 ADD2 TAOK1	0.2687	0.4839	1.51
GO:0005777	peroxisome	cellular_component	4/398	149/22360	ACSL6 MYO5A ACOT8 ACOX1	0.2687	0.4839	1.51
GO:0061041	regulation of wound healing	biological_process	4/398	149/22360	DUOX1 WNT4 ANO6 F2R	0.2687	0.4839	1.51
GO:0045834	positive regulation of lipid metabolic process	biological_process	4/398	149/22360	DGKZ AKT2 MLXIPL WNT4	0.2687	0.4839	1.51
GO:1901991	negative regulation of mitotic cell cycle phase transition	biological_process	7/398	280/22360	USP44 TAOK1 TP53 PSMD9 MSH6 MDM4 CHEK2	0.2708	0.4869	1.4
GO:0022406	membrane docking	biological_process	5/398	192/22360	RAB3B STX16 ESYT3 CC2D2A VPS33A	0.2714	0.4878	1.46
GO:0042391	regulation of membrane potential	biological_process	11/398	465/22360	CNR2 AKT2 ABCB5 SEZ6 GCLC P2RX4 APP GJC1 CACNB2 GABRB3 GSK3B	0.2725	0.4886	1.33
GO:0048813	dendrite morphogenesis	biological_process	4/398	150/22360	GSK3B SRCIN1 TMEM106B FARP1	0.274	0.4869	1.5
GO:0043069	negative regulation of programmed cell death	biological_process	23/398	1050/22360	GSK3B EYA3 KRT18 GABRB3 GCLC EIF2AK2 ARNT2 CX3CL1 AKT2 WNT4 SON SLC35F6 F2R GAS6 MDM4 MNT XIAP GRK5 LGMN TP53 CASP8 NMNAT1 CCL5	0.2742	0.4862	1.23

GO:0043086	negative regulation of catalytic activity	biological_process	20/398	902/22360	APP XIAP BCAS3 ITIH5 PAPLN ADAR PRKAR2A CASP8 SMCR8 PPP1R37 TP53 FARP1 STYX HP GSK3B CNR2 GAS6 SRCIN1 USP44 TNK2	0.2759	0.4891	1.25
GO:0071902	positive regulation of protein serine/threonine kinase activity	biological_process	9/398	373/22360	CXCR4 F2R TAOK1 MAP3K2 MAP3K9 MAPKAPK5 EIF2AK2 SYAP1 CAMK1	0.2767	0.4903	1.36
GO:0006397	mRNA processing	biological_process	14/398	610/22360	PDE12 RPRD1B ADAR BRDT APP NSRP1 CWC25 ZC3H14 SON TSEN2 HNRNPUL1 C9ORF78 SRSF10 PNPT1	0.278	0.4925	1.29
GO:0042177	negative regulation of protein catabolic process	biological_process	4/398	151/22360	SMARCC1 MDM4 STYX CYP51A1	0.2792	0.4944	1.49
GO:0034332	adherens junction organization	biological_process	4/398	151/22360	WHAMM PEAK1 WNT4 BCAS3	0.2792	0.4944	1.49
GO:0006508	proteolysis	biological_process	41/398	1967/22360	UBXN2A FBXL4 PSMD9 PCSK7 VPS28 DDI2 STYX OTUD4 TRABD2B UBE3C DPP9 TP53 KLHL8 FBXL18 CTSS NLRP3 TRIM72 SMARCC1 XIAP USP28 TTC3 F2R USP44 TRIM25 MDM4 RNF7 DESI1 GAS6 HP ATXN3 GSK3B GCLC RNF115 CASP8 SUMO2 LGMN PAPLN ITIH5 MAPK12 SMAD2 APP	0.28	0.4955	1.17
GO:0017048	Rho GTPase binding	molecular_function	5/398	194/22360	ARHGEF39 FGD5 FARP1 WHAMM SRGAP2B	0.2806	0.4955	1.45
GO:0000790	nuclear chromatin	cellular_component	10/398	421/22360	TP53 WBP2 MSH6 TTI2 MXD1 NCOR1 RARG TCF4 SMARCC1 SMAD2	0.2813	0.4966	1.33
GO:0061337	cardiac conduction	biological_process	4/398	152/22360	ATP2B1 ABCC9 CACNB2 GJC1	0.2845	0.5021	1.48
GO:0044281	small molecule metabolic process	biological_process	44/398	2125/22360	ACOX1 NCOR1 GSK3B CYP51A1 GCLC ACSL6 H6PD QPRT MGLL FDPS PDP2 ITIH5 DCTD APP COX5B SULT1B1 CRYZL1 LGMN ACOT8 NAGK COQ6 UEVLD AKT2 ASPA MTAP VNN2 PGM2L1 PDE4C PSMD9 MLXIPL KDSR WNT4 ADCY2 GK5 APOL1 GPAT4 SLC26A2 CYP4V2 AS3MT APOBEC3D MYO5A GNS PPM1K NMNAT1	0.2846	0.5021	1.16
GO:0010565	regulation of cellular ketone metabolic process	biological_process	5/398	195/22360	WNT4 PSMD9 MLXIPL PDP2 AKT2	0.2853	0.4989	1.44
GO:0006479	protein methylation	biological_process	5/398	195/22360	NDUFAF7 RAB3B ZNF274 KMT2D METTL21A	0.2853	0.4989	1.44
GO:0000165	MAPK cascade	biological_process	23/398	1058/22360	C5AR2 APP CSF2RA MAPK12 MAP3K9 SPTAN1 MAPKAPK5 NMNAT1 CCL5 EIF2AK2 CX3CL1 STYX MAP3K2 TAOK1 LTBR NCOR1 GAS6 CNKSR3 PSMD9 CXCR4 IRAK4 EMC10 F2R	0.2894	0.5042	1.22
GO:0006875	cellular metal ion homeostasis	biological_process	14/398	615/22360	ATP6V1A CCL5 CCR4 C5AR2 MYO5A APP CCR6 CACNB2 SLC39A13 F2R CXCR4 CX3CL1 P2RX4 ATP2B1	0.2906	0.5061	1.28
GO:0043010	camera-type eye development	biological_process	8/398	331/22360	SOX11 PKNOX1 TRAF3IP1 COL8A1 PTPRM CC2D2A ATP2B1 RARG	0.2908	0.5063	1.36
GO:0097190	apoptotic signaling pathway	biological_process	15/398	664/22360	CHEK2 GCLC P2RX4 CX3CL1 LTBR KRT18 GSK3B EYA3 MNT WNT4 USP28 SLC35F6 MSH6 TP53 CASP8	0.2919	0.508	1.27
GO:0004857	enzyme inhibitor activity	molecular_function	10/398	425/22360	APP XIAP STYX PAPLN ITIH5 PRKAR2A GAS6 SMCR8 PPP1R37 TNK2	0.2937	0.5106	1.32
GO:0008021	synaptic vesicle	cellular_component	5/398	197/22360	SYT12 SYP APP RAB3B STX16	0.2947	0.5122	1.43
GO:0003774	motor activity	molecular_function	4/398	154/22360	MYO1C MYO5A DNAL1 DYNC2LI1	0.2953	0.5131	1.46
GO:0022607	cellular component assembly	biological_process	69/398	3441/22360	TP53 NLRP3 BCAS3 DRG1 SPTAN1 MYO1C TMOD3 TRIM72 PSMD9 FAM161A FGD5 UBXN2A OGFOD1 PCDHB9 PEAK1 SRSF10 VPS28 TCF4 EIF2AK2 P2RX4 FHDC1 NUBPL PSTPIP2 DNAL1 CCL5 CORO7 NDUFA10 APP QPRT TMEM170A GAS6 DYNC2LI1 GSK3B NCOR1 TTLL3 SMCR8 RAB29 CD3G MARVELD2 BBS1 WNT4 RILPL1 CC2D2A GABRB3 ODF2L PNPT1 WHAMM TRABD2B CX3CL1 ADD2 DHX30 NDUFAF7 CASP8 ADAR GJC1 ANO6 IBA57 RABGAP1 SMAD2 TTYH1 NAP1L6 TENM2 TRAF3IP1 MDM4 RFTN1 HBE1 CHEK2 KMT2D FARP1	0.2976	0.5117	1.13
GO:0019887	protein kinase regulator activity	molecular_function	5/398	198/22360	CCL5 GAS6 PRKAR2A TAOK1 SMCR8	0.2995	0.5148	1.42

GO:0043401	steroid hormone mediated signaling pathway	biological_process	5/398	198/22360	DDX54 NCOR1 KMT2D WBP2 RARG	0.2995	0.5148	1.42
GO:0050727	regulation of inflammatory response	biological_process	10/398	427/22360	NLRP3 CCL5 CNR2 MGLL SBNO2 SHPK XIAP APP CX3CL1 CALCRL	0.3	0.515	1.32
GO:0007166	cell surface receptor signaling pathway	biological_process	70/398	3496/22360	RARG SMARCC1 CCR6 HIF1AN ITGA3 PGF AAK1 CD3G RAB29 BTN3A2 IFNAR1 CX3CL1 TRABD2B OTUD4 CC2D2A LTBR GABRB3 KRT18 YES1 OAS3 WNT4 GRK5 SMAD2 CSF2RA DUOX1 ANO6 ADAR LGMN CASP8 SEZ6 RFTN1 TRAF3IP1 TNK2 F2R XIAP PIK3R3 TRIM72 CALCRL MYO1C ICOSLG SLC33A1 ATP6V1A ERMAP ZNF8 TP53 P2RX4 MTAP AKT2 EYA3 PSMD9 ZNF106 TIE1 APP C5AR2 NXN BTNL9 CCL5 CCR4 PIGR RNF115 GCLC GSK3B RABGAP1 BCL7B GAS6 SOX11 IRAK4 CXCR4 EPN1 TRIM25	0.3001	0.515	1.12
GO:0032502	developmental process	biological_process	136/398	7012/22360	EYA3 PEAK1 FGD5 PSMD9 SBNO2 XKR4 ZNF7 LRTOMT PCDHB9 GPAT4 CALCRL MAPKAPK5 SLC35E2B TMOD3 DRG1 C2ORF49 PIK3R3 SYAP1 TP53 LIG1 VSIG1 BCL7B RABGAP1 GSK3B GCLC ACSL6 EMC10 SOX11 DYNC2LI1 HERC4 RCAN1 NXN APP CCR4 PLEKHB2 ARFRP1 LTBR ZFPM2 YES1 MYO5A CCR6 ICA1L TANC2 N4BP2L2 TTLL7 SPN ATXN3 PTPRM CHEK2 SEZ6 ARNT2 ZNF430 KMT2D NSRP1 DUOX1 SMAD2 DHX30 TBCCD1 ADD2 CASP8 SULT1B1 VPS52 EIF2AK2 P2RX4 ATP2B1 TCF4 NUBPL AKT2 ASPA MLXIPL TIE1 BCAS3 ZDHHC15 EXOSC6 CYB5R4 SPTAN1 TRIM72 ABCB5 CCDC36 RIPPLY3 NLRP3 MXD1 EPN1 CXCR4 GAS6 SSTR2 MAPK12 RPL7L1 FITM2 SLC9A4 SLC12A2 ZMYM5 KRT18 GABRB3 KLHL1 CC2D2A PNPT1 TAF8 CX3CL1 WNT4 RILPL1 PGF ITGA3 HIF1AN SLC39A13 MARVELD2 SYP SMARCC1 FOXD2 RARG BRDT RAB29 CD3G COL8A1 SRGAP2B POU5F1 IKZF3 MSH6 PKNOX1 FARP1 CRX F2R TENM2 TNK2 TRAF3IP1 SRCIN1 MNT GJC1 ANO6 GRK5 FTO TMEM106B CAMK1 LGMN VPS33A ZHX3	0.3008	0.516	1.09
GO:0042325	regulation of phosphorylation	biological_process	36/398	1725/22360	CHEK2 NCOR1 TAOK1 GAS6 SRCIN1 TRAF3IP1 CNKSR3 TNK2 EMC10 F2R CXCR4 CAMK1 APP C5AR2 MAP3K9 PRKAR2A DGKZ CCL5 ADAR CX3CL1 STYX AKT2 EIF2AK2 MAP3K2 LTBR ADCY2 YES1 MLXIPL SYAP1 PIK3R3 MAPKAPK5 PGF PDE12 NMNAT1 SMCR8 TP53	0.3017	0.5174	1.17
GO:0004866	endopeptidase inhibitor activity	molecular_function	5/398	199/22360	PAPLN ITIH5 APP GAS6 XIAP	0.3042	0.5215	1.41
GO:0006119	oxidative phosphorylation	biological_process	4/398	156/22360	MLXIPL COX5B NDUFA10 PDE12	0.3062	0.5229	1.44
GO:0071322	cellular response to carbohydrate stimulus	biological_process	4/398	156/22360	GAS6 GCLC CALCRL MLXIPL	0.3062	0.5229	1.44
GO:0008203	cholesterol metabolic process	biological_process	4/398	156/22360	CYP51A1 FDPS APP APOL1	0.3062	0.5229	1.44
GO:0007169	transmembrane receptor protein tyrosine kinase signaling pathway	biological_process	17/398	768/22360	ZNF106 TIE1 EPN1 WNT4 YES1 RABGAP1 AKT2 RNF115 PIGR LGMN ATP6V1A MYO1C PGF TRIM72 PIK3R3 APP SMARCC1	0.3065	0.5229	1.24
GO:0070555	response to interleukin-1	biological_process	6/398	244/22360	IRAK4 PSMD9 OTUD4 CX3CL1 CCL5 GCLC	0.3066	0.5229	1.38
GO:0001654	eye development	biological_process	9/398	382/22360	CC2D2A PTPRM RARG ATP2B1 ABCB5 COL8A1 TRAF3IP1 PKNOX1 SOX11	0.3066	0.5229	1.32
GO:0006082	organic acid metabolic process	biological_process	27/398	1269/22360	SULT1B1 NAGK ACOT8 ITIH5 PDP2 MGLL QPRT NCOR1 ACOX1 ACSL6 GCLC NMNAT1 PPM1K GNS GPAT4 MYO5A AS3MT SLC26A2 CYP4V2 PSMD9 MLXIPL UEVLD PGM2L1 VNN2 MTAP ASPA AKT2	0.3073	0.5237	1.2
GO:0015629	actin cytoskeleton	cellular_component	12/398	525/22360	INTS6 YES1 SRCIN1 ADD2 DENND2A MYO5A RAB22A PSTPIP2 SPTAN1 MYO1C PEAK1 TMOD3	0.3081	0.5249	1.28
GO:0006164	purine nucleotide biosynthetic process	biological_process	5/398	200/22360	PDP2 ADCY2 SLC26A2 COX5B ACSL6	0.3091	0.5264	1.4

GO:1901796	regulation of signal transduction by p53 class mediator	biological_process	5/398	200/22360	CHEK2 MDM4 RFC2 TP53 MAPKAPK5	0.3091	0.5264	1.4
GO:0044262	cellular carbohydrate metabolic process	biological_process	7/398	290/22360	NAGK GK5 PYGB PGM2L1 AKT2 GSK3B GNPTG	0.3092	0.5263	1.36
GO:0007015	actin filament organization	biological_process	10/398	430/22360	CORO7 MYO1C SPTAN1 TMOD3 WHAMM FHDC1 CX3CL1 PSTPIP2 ADD2 WNT4	0.3095	0.523	1.31
GO:0008234	cysteine-type peptidase activity	molecular_function	11/398	478/22360	XIAP OTUD4 ATXN3 CTSS NLRP3 GAS6 LGMN USP28 F2R USP44 CASP8	0.3109	0.5252	1.29
GO:0042113	B cell activation	biological_process	6/398	245/22360	CCR6 ICOSLG EXOSC6 MSH6 IKZF3 CASP8	0.311	0.5252	1.38
GO:0023014	signal transduction by protein phosphorylation	biological_process	23/398	1069/22360	F2R EMC10 CXCR4 IRAK4 PSMD9 GAS6 CNKSR3 MAP3K2 LTBR NCOR1 TAOK1 CX3CL1 STYX EIF2AK2 NMNAT1 CCL5 SPTAN1 MAPKAPK5 MAP3K9 CSF2RA MAPK12 APP C5AR2	0.311	0.5252	1.21
GO:0051668	localization within membrane	biological_process	4/398	157/22360	RILPL1 CEP112 PIGR RFTN1	0.3118	0.5262	1.43
GO:0010212	response to ionizing radiation	biological_process	4/398	157/22360	CHEK2 USP28 TP53 EYA3	0.3118	0.5262	1.43
GO:0000228	nuclear chromosome	cellular_component	15/398	672/22360	TONSL SMAD2 CCDC36 MMS22L RARG SMARCC1 MSH6 MXD1 WBP2 TP53 RGS12 TCF4 NCOR1 TTI2 C9ORF84	0.312	0.5262	1.25
GO:0002520	immune system development	biological_process	24/398	1120/22360	SPN N4BP2L2 LTBR EIF2AK2 KMT2D PSMD9 WNT4 SBNO2 GAS6 EXOSC6 TMOD3 RARG APP CCR6 ADD2 CCR4 CASP8 NLRP3 CD3G VPS33A LIG1 IKZF3 PKNOX1 MSH6	0.3123	0.5265	1.2
GO:1902531	regulation of intracellular signal transduction	biological_process	42/398	2042/22360	NCOR1 TAOK1 GSK3B FARP1 SEZ6 CHEK2 EMC10 F2R TRIM25 IRAK4 CXCR4 SOX11 CCDC125 MDM4 CNKSR3 GAS6 RCAN1 MAP3K9 APP C5AR2 CASP8 TBCK CCL5 LTBR ARHGEF39 MAP3K2 CARD19 STYX CX3CL1 P2RX4 EIF2AK2 FGD5 MAPKAPK5 ITGA3 SCAI MYO5A XIAP SMCR8 TP53 RFC2 NLRP3 NMNAT1	0.3129	0.5272	1.16
GO:0007249	I-kappaB kinase/NF-kappaB signaling	biological_process	7/398	291/22360	F2R CASP8 IRAK4 TRIM25 CX3CL1 CARD19 LTBR	0.3132	0.5267	1.35
GO:0009890	negative regulation of biosynthetic process	biological_process	37/398	1784/22360	ZNF345 TENM2 GAS6 MDM4 MNT SOX11 GSK3B N4BP2L2 NCOR1 CHEK2 GCLC KMT2D ZHX3 ACOT8 MLX DDX54 CIART SMAD2 FTO SAMD4B MLXIPL WNT4 NPAT SBNO2 ZFPM2 ZNF274 EIF2AK2 RIPPLY3 ZNF8 TP53 MXD1 MSH6 POU5F1 HIF1AN RARG SCAI TONSL	0.3134	0.5268	1.17
GO:0099504	synaptic vesicle cycle	biological_process	5/398	201/22360	RAB3B CACNB2 GSK3B SYT12 SYP	0.3139	0.5275	1.4
GO:0046890	regulation of lipid biosynthetic process	biological_process	5/398	201/22360	FDPS CYP51A1 WNT4 MLXIPL FITM2	0.3139	0.5275	1.4
GO:0045444	fat cell differentiation	biological_process	6/398	246/22360	GRK5 TAF8 SYAP1 FTO AKT2 ZFPM2	0.3153	0.5295	1.37
GO:0032496	response to lipopolysaccharide	biological_process	9/398	385/22360	SBNO2 F2R SHPK CASP8 NLRP3 CCL5 CNR2 IFNAR1 CX3CL1	0.3169	0.532	1.31
GO:0050865	regulation of cell activation	biological_process	15/398	674/22360	GCLC CX3CL1 SPN GAS6 YES1 SOX11 CNR2 APP EXOSC6 ICOSLG CCL5 NLRP3 MSH6 IKZF3 SHPK	0.3171	0.5322	1.25
GO:0016331	morphogenesis of embryonic epithelium	biological_process	4/398	158/22360	WNT4 CC2D2A SOX11 RARG	0.3173	0.5323	1.42
GO:0048489	synaptic vesicle transport	biological_process	4/398	158/22360	SYP SYT12 GSK3B CACNB2	0.3173	0.5323	1.42
GO:0016052	carbohydrate catabolic process	biological_process	5/398	202/22360	MLXIPL NCOR1 PYGB PGM2L1 GK5	0.3188	0.5345	1.39
GO:0007623	circadian rhythm	biological_process	5/398	202/22360	TP53 NCOR1 CRX GSK3B CIART	0.3188	0.5345	1.39
GO:0007369	gastrulation	biological_process	5/398	202/22360	COL8A1 SMAD2 ARFRP1 POU5F1 ITGA3	0.3188	0.5345	1.39
GO:0010921	regulation of phosphatase activity	biological_process	5/398	202/22360	PPP1R37 GSK3B RCAN1 FARP1 EIF2AK2	0.3188	0.5345	1.39

GO:0016567	protein ubiquitination	biological_process	20/398	923/22360	DCUN1D2 RAB40B VPS28 GCLC UBE3C PCGF5 PSMD9 TRIM25 FBXL4 UBXN2A TTC3 USP44 RNF7 HERC4 NXN XIAP TRIM72 RNF115 FBXL18 KLHL8	0.3203	0.536	1.22
GO:0150063	visual system development	biological_process	9/398	386/22360	PKNOX1 SOX11 TRAF3IP1 COL8A1 PTPRM CC2D2A ABCB5 RARG ATP2B1	0.3204	0.536	1.31
GO:0014074	response to purine-containing compound	biological_process	4/398	159/22360	PNPT1 P2RX4 APP DUOX1	0.3229	0.5369	1.41
GO:0001764	neuron migration	biological_process	4/398	159/22360	ITGA3 CCR4 CX3CL1 GAS6	0.3229	0.5369	1.41
GO:0038093	Fc receptor signaling pathway	biological_process	5/398	203/22360	PSMD9 PIGR MYO1C YES1 CD3G	0.3237	0.5378	1.38
GO:0000977	RNA polymerase II regulatory region sequence-specific DNA binding	molecular_function	18/398	825/22360	MLXIPL CRX PCGF5 GMEB1 SOX11 MNT TCF4 ARNT2 TP53 WBP2 ZHX3 MXD1 IKZF3 MLX CIART SMARCC1 RARG SMAD2	0.3242	0.5385	1.23
GO:0006816	calcium ion transport	biological_process	11/398	483/22360	ATP2B1 P2RX4 MYO5A CX3CL1 CALCRL CACNB2 ANO6 GAS6 CCL5 STAC2 F2R	0.3263	0.5413	1.28
GO:0008289	lipid binding	molecular_function	17/398	777/22360	SYT12 ESYT3 SNX27 PLEKHB2 TRIM72 GRK5 SYP SNX21 APOL1 GAS6 PLEKHA2 GSDMB EPN1 GSDMA ACOX1 PXDC1 SNX29	0.3281	0.5441	1.23
GO:0007167	enzyme linked receptor protein signaling pathway	biological_process	24/398	1128/22360	APP SMAD2 LGMN PIGR RNF115 RABGAP1 EPN1 GAS6 SOX11 PGF ITGA3 SLC33A1 MYO1C SMARCC1 XIAP PIK3R3 TRIM72 ZNF8 ATP6V1A AKT2 WNT4 TIE1 ZNF106 YES1	0.3282	0.5441	1.2
GO:0031461	cullin-RING ubiquitin ligase complex	cellular_component	4/398	160/22360	FBXL4 RNF7 FBXL18 KLHL8	0.3286	0.5446	1.4
GO:0030136	clathrin-coated vesicle	cellular_component	6/398	249/22360	SYNRG VPS33A CD3G STEAP2 TNK2 AAK1	0.3286	0.5446	1.35
GO:0031669	cellular response to nutrient levels	biological_process	6/398	249/22360	TP53 WNT4 EIF2AK2 ATP2B1 P2RX4 GAS6	0.3286	0.5446	1.35
GO:0005262	calcium channel activity	molecular_function	5/398	204/22360	STAC2 CACNB2 GAS6 MYO5A P2RX4	0.3287	0.5442	1.38
GO:0043254	regulation of protein complex assembly	biological_process	11/398	484/22360	TRAF3IP1 TP53 ADD2 TRABD2B WHAMM EIF2AK2 TMOD3 MYO1C SPTAN1 DRG1 GSK3B	0.3295	0.5454	1.28
GO:0007268	chemical synaptic transmission	biological_process	17/398	778/22360	GSK3B ATXN3 GABRB3 P2RX4 CX3CL1 SEZ6 F2R CNR2 PCDHB9 CACNB2 GJC1 SYP MYO5A APP LGMN RAB3B SYT12	0.3306	0.5454	1.23
GO:0036477	somatodendritic compartment	cellular_component	19/398	879/22360	CCR4 MYO5A APP SYAP1 TENM2 ZC3H14 SRCIN1 ADCY2 CNR2 KLHL1 TTLL7 GSK3B P2RX4 ATP2B1 FARP1 SEZ6 CX3CL1 RGS12 KCNN3	0.3338	0.55	1.21
GO:0017156	calcium ion regulated exocytosis	biological_process	4/398	161/22360	CACNB2 GSK3B SYT12 SYP	0.3343	0.5467	1.4
GO:0050851	antigen receptor-mediated signaling pathway	biological_process	8/398	343/22360	BTNL9 ICOSLG RFTN1 BTN3A2 PSMD9 ERMAP CD3G RAB29	0.335	0.5476	1.31
GO:0046395	carboxylic acid catabolic process	biological_process	7/398	297/22360	AKT2 ASPA ACOX1 NAGK PPM1K ACOT8 QPRT	0.3375	0.5515	1.32
GO:0051495	positive regulation of cytoskeleton organization	biological_process	6/398	251/22360	WHAMM CX3CL1 WNT4 BCAS3 DRG1 MYO1C	0.3376	0.5515	1.34
GO:0030414	peptidase inhibitor activity	molecular_function	5/398	206/22360	PAPLN ITIH5 APP XIAP GAS6	0.3387	0.5526	1.36
GO:0070372	regulation of ERK1 and ERK2 cascade	biological_process	8/398	344/22360	CX3CL1 STYX APP C5AR2 F2R GAS6 CCL5 CNKSR3	0.3388	0.5526	1.31
GO:0001228	DNA-binding transcription activator activity, RNA polymerase II-specific	molecular_function	10/398	439/22360	TCF4 FOXD2 SMAD2 ARNT2 GMEB1 TP53 CRX SOX11 PKNOX1 IKZF3	0.3392	0.5531	1.28
GO:0009267	cellular response to starvation	biological_process	4/398	162/22360	WNT4 TP53 GAS6 EIF2AK2	0.34	0.5539	1.39
GO:0030425	dendrite	cellular_component	14/398	634/22360	RGS12 SEZ6 FARP1 P2RX4 GSK3B KLHL1 TTLL7 ADCY2 CNR2 SRCIN1 ZC3H14 TENM2	0.3415	0.5561	1.24

					SYAP1 APP			
GO:0048167	regulation of synaptic plasticity	biological_process	5/398	207/22360	SYT12 CX3CL1 APP SYP LGMN	0.3437	0.5596	1.36
GO:0050806	positive regulation of synaptic transmission	biological_process	5/398	207/22360	RAB3B CACNB2 LGMN SYT12 APP	0.3437	0.5596	1.36
GO:0071897	DNA biosynthetic process	biological_process	5/398	207/22360	LIG1 RFC2 TRIM25 TP53 MAPKAPK5	0.3437	0.5596	1.36
GO:0031410	cytoplasmic vesicle	cellular_component	54/398	2691/22360	APP SNX21 CORO7 PIGR SNX27 PLEKHB2 SLC9A7 GAS6 IRAK4 CXCR4 TRIM72 CALCRL MYO1C SPTAN1 SYT12 AKT2 VPS52 VPS28 GALNT15 AP4M1 FGD5 PYGB STEAP2 SYNRG TMEM106B ANO6 STX16 VPS33A CYB561A3 ADD2 LGMN RAB3B TMEM187 RFTN1 HP C1ORF210 F2R TNK2 MARVELD2 MYO5A SYP AAK1 AP4E1 RAB29 CD3G CTSS GNS ICA1L IFNAR1 WHAMM RAB22A GABRB3 LRPAP1 WNT4	0.3441	0.5597	1.13
GO:0043406	positive regulation of MAP kinase activity	biological_process	7/398	299/22360	MAPKAPK5 TAOK1 MAP3K9 MAP3K2 EIF2AK2 F2R CXCR4	0.3458	0.5623	1.32
GO:0051050	positive regulation of transport	biological_process	23/398	1086/22360	CTSS CCL5 RAB29 NLRP3 CASP8 RAB3B TP53 CAMK1 ANO6 MYO1C CACNB2 BCAS3 SOX11 CNKSR3 GAS6 F2R STAC2 PSMD9 AKT2 CX3CL1 P2RX4 VPS28 GSK3B	0.3461	0.5626	1.19
GO:0097447	dendritic tree	cellular_component	14/398	636/22360	TENM2 ADCY2 CNR2 ZC3H14 SRCIN1 KLHL1 TTLL7 GSK3B FARP1 RGS12 SEZ6 P2RX4 APP SYAP1	0.3471	0.5596	1.24
GO:0000976	transcription regulatory region sequence-specific DNA binding	molecular_function	19/398	885/22360	ARNT2 TCF4 GMEB1 MLXIPL CRX PCGF5 SOX11 MNT CIART MLX SMAD2 RARG SMARCC1 TP53 POU5F1 MXD1 IKZF3 WBP2 ZHX3	0.3479	0.5597	1.21
GO:0097708	intracellular vesicle	cellular_component	54/398	2694/22360	STEAP2 TMEM106B SYNRG ANO6 STX16 VPS33A CYB561A3 ADD2 LGMN RAB3B TMEM187 RFTN1 HP C1ORF210 F2R TNK2 MYO5A MARVELD2 SYP AAK1 AP4E1 CD3G RAB29 CTSS GNS ICA1L IFNAR1 WHAMM RAB22A GABRB3 LRPAP1 WNT4 APP SNX21 CORO7 PIGR SNX27 PLEKHB2 SLC9A7 GAS6 CXCR4 IRAK4 TRIM72 CALCRL SPTAN1 MYO1C SYT12 AKT2 VPS52 GALNT15 VPS28 AP4M1 FGD5 PYGB	0.3481	0.5598	1.13
GO:0045333	cellular respiration	biological_process	5/398	208/22360	NDUFA10 COX5B NOA1 CS PNPT1	0.3488	0.5608	1.35
GO:0010876	lipid localization	biological_process	10/398	442/22360	P2RX4 AKT2 ABCC3 FTO NCOR1 ANO6 APOL1 XKR4 ESYT3 FITM2	0.3494	0.5616	1.27
GO:0031902	late endosome membrane	cellular_component	4/398	164/22360	VPS28 CYB561A3 TMEM106B VPS33A	0.3516	0.5649	1.37
GO:0032259	methylation	biological_process	9/398	395/22360	MTAP AS3MT KMT2D ZNF274 TRMT10B LRTOMT METTL21A RAB3B NDUFAF7	0.3525	0.5662	1.28
GO:0051015	actin filament binding	molecular_function	5/398	209/22360	ADD2 CACNB2 MYO1C MYO5A PSTPIP2	0.3539	0.5681	1.34
GO:0007626	locomotory behavior	biological_process	5/398	209/22360	KLHL1 CIART MYO5A APP SEZ6	0.3539	0.5681	1.34
GO:0042393	histone binding	molecular_function	5/398	209/22360	BCAS3 KMT2D MSH6 TONSL BRDT	0.3539	0.5681	1.34
GO:0010632	regulation of epithelial cell migration	biological_process	7/398	301/22360	CCR6 P2RX4 PTPRM BCAS3 ITGA3 EMC10 LGMN	0.3542	0.5681	1.31
GO:0000122	negative regulation of transcription by RNA polymerase II	biological_process	19/398	888/22360	TP53 ZNF8 RIPPLY3 ZHX3 MXD1 POU5F1 HIF1AN MLX RARG SMAD2 MLXIPL ZNF345 ZFPM2 TENM2 MDM4 SOX11 MNT NCOR1 N4BP2L2	0.3551	0.5693	1.2
GO:1903364	positive regulation of cellular protein catabolic process	biological_process	4/398	165/22360	GCLC ATXN3 GSK3B SUMO2	0.3574	0.572	1.36
GO:2001056	positive regulation of cysteine-type endopeptidase activity	biological_process	4/398	165/22360	LGMN CASP8 F2R NLRP3	0.3574	0.572	1.36
GO:0071363	cellular response to growth factor stimulus	biological_process	16/398	739/22360	GCLC SOX11 YES1 GAS6 WNT4 SMAD2 APP SYAP1 XIAP SLC33A1 MYO1C PGF ITGA3 CCL5 ZNF8 LGMN	0.3579	0.5724	1.22

GO:0000978	RNA polymerase II proximal promoter sequence-specific DNA binding	molecular_function	12/398	542/22360	TP53 WBP2 MXD1 CIART SMARCC1 SMAD2 CRX MLXIPL GMEB1 SOX11 TCF4 ARNT2	0.3593	0.5725	1.24
GO:0040011	locomotion	biological_process	42/398	2073/22360	DGKZ CCL5 SLC12A2 ADD2 LGMN TBCCD1 CCR4 C5AR2 APP DUOX1 ANO6 SRCIN1 GAS6 CNR2 CCDC125 CXCR4 IRAK4 TRIM25 EMC10 F2R SPN PTPRM SRGAP2B SCYL3 MYO5A SCAI PIK3R3 CCR6 BCAS3 PGF ITGA3 SPTAN1 MYO1C YES1 WNT4 TIE1 P2RX4 CX3CL1 PSTPIP2 AKT2 ARHGEF39 PEAK1	0.3594	0.5725	1.14
GO:0070374	positive regulation of ERK1 and ERK2 cascade	biological_process	6/398	256/22360	APP CCL5 C5AR2 GAS6 CX3CL1 F2R	0.3605	0.5737	1.32
GO:0043408	regulation of MAPK cascade	biological_process	18/398	840/22360	STYX CX3CL1 EIF2AK2 TAOK1 NCOR1 LTBR MAP3K2 CNKSR3 GAS6 F2R EMC10 CXCR4 C5AR2 APP MAPKAPK5 MAP3K9 CCL5 NMNAT1	0.3605	0.5737	1.2
GO:0021915	neural tube development	biological_process	4/398	166/22360	CC2D2A RARG TRAF3IP1 SOX11	0.3633	0.5778	1.35
GO:0048880	sensory system development	biological_process	9/398	398/22360	PKNOX1 SOX11 COL8A1 TRAF3IP1 ABCB5 RARG ATP2B1 PTPRM CC2D2A	0.3635	0.578	1.27
GO:0043269	regulation of ion transport	biological_process	16/398	742/22360	CTSS CCL5 KCNJ15 SYT12 RAB3B MYO5A APP CACNB2 ANO6 CNKSR3 STAC2 F2R P2RX4 CX3CL1 AKT2 NCOR1	0.3659	0.5812	1.21
GO:0001775	cell activation	biological_process	34/398	1661/22360	P2RX4 CX3CL1 LTBR YES1 WNT4 SBNO2 PYGB CCR6 EXOSC6 ICOSLG SPTAN1 CTSS RAB29 CD3G NLRP3 GNS MSH6 PKNOX1 IKZF3 SHPK GCLC HP SPN GAS6 SOX11 CNR2 F2R APP ANO6 CCL5 DGKZ PIGR SNX27 CASP8	0.3664	0.5818	1.15
GO:0006281	DNA repair	biological_process	13/398	594/22360	MMS22L TONSL FTO LIG1 RFC2 MSH6 TP53 CHEK2 TAOK1 ATXN3 EYA3 TRIM25 USP28	0.3682	0.5838	1.23
GO:0060402	calcium ion transport into cytosol	biological_process	4/398	167/22360	MYO5A P2RX4 CX3CL1 F2R	0.3692	0.5852	1.35
GO:0046390	ribose phosphate biosynthetic process	biological_process	5/398	212/22360	PDP2 ADCY2 SLC26A2 COX5B ACSL6	0.3694	0.5854	1.33
GO:0019538	protein metabolic process	biological_process	123/398	6397/22360	MKNK1 SMAD2 SAMD4B SUMO2 NDUFAF7 CASP8 NOA1 SPN ATXN3 TAOK1 TTLL7 PTPRM CHEK2 KMT2D USP44 MDM4 AAK1 SCYL3 PPP1R37 SMCR8 NMNAT1 FBXL18 WBP2 CTSS KLHL8 LTBR ZNF274 OTUD4 YES1 ADCY2 PCSK7 MAP3K9 RCAN1 NXN PDP2 APP RNF115 CCL5 ACOT8 RPRD1B GSK3B NCOR1 GCLC B3GALT1 TRIM25 EMC10 RNF7 KDELC2 HERC4 DRG1 MAPKAPK5 APOL1 SYAP1 PIK3R3 TP53 METTL21A EYA3 MAP3K2 PEAK1 VPS28 UBE3C STYX PSMD9 FAM161A TMTC1 OGFOD1 PAPLN A4GALT GRK5 CSF2RA FTO CAMK1 TBCK LGMN RAB3B PRKAR2A ADAR DCUN1D2 HP TNK2 TTC3 F2R SRCIN1 TRAF3IP1 HIF1AN PGF GALNT16 SMARCC1 DDI2 DPP9 TRABD2B CX3CL1 PIGL ITIH5 C5AR2 MAPK12 PLPP2 MTFMT TTLL3 CYP51A1 PCGF5 CXCR4 IRAK4 USP28 SUMF2 DESI1 GAS6 CNKSR3 ZDHHC15 SPTAN1 XIAP TRIM72 NLRP3 PPM1K RAB40B UEVLD GALNT15 EIF2AK2 AKT2 MLXIPL FBXL4 TIE1 UBXN2A	0.3698	0.5858	1.08
GO:0043565	sequence-specific DNA binding	molecular_function	25/398	1200/22360	ZHX3 SMAD2 CIART MLX MNT SOX11 ZNF345 PCGF5 CRX ARNT2 NCOR1 POU5F1 IKZF3 MXD1 PKNOX1 WBP2 NLRP3 TP53 SMARCC1 FOXD2 RARG GMEB1 MLXIPL TCF4 ZNF274	0.3718	0.5866	1.17
GO:0060341	regulation of cellular localization	biological_process	20/398	946/22360	GSK3B VPS28 P2RX4 AKT2 CX3CL1 F2R STAC2 GAS6 BCAS3 ITGA3 CACNB2 MYO1C MYO5A SYP CAMK1 TP53 CASP8 RAB3B RAB29 SYT12	0.3731	0.5885	1.19
GO:0045786	negative regulation of cell cycle	biological_process	16/398	745/22360	MDM4 SOX11 GAS6 USP44 USP28 MLXIPL PSMD9 AKT2 CHEK2 WHAMM PNPT1 TAOK1 MSH6 TP53 MAPK12 PRR11	0.374	0.5883	1.21
GO:0060541	respiratory system development	biological_process	5/398	213/22360	SOX11 SMAD2 RARG ZFPM2 ITGA3	0.3747	0.5892	1.32

GO:0034765	regulation of ion transmembrane transport	biological_process	11/398	498/22360	STAC2 F2R CNKSR3 CTSS KCNJ15 CACNB2 ANO6 MYO5A APP CX3CL1 AKT2	0.3749	0.5894	1.24
GO:0048592	eye morphogenesis	biological_process	4/398	168/22360	SOX11 COL8A1 RARG PTPRM	0.3751	0.5895	1.34
GO:0031214	biomineral tissue development	biological_process	4/398	168/22360	ANO6 SBNO2 WNT4 GAS6	0.3751	0.5895	1.34
GO:0044839	cell cycle G2/M phase transition	biological_process	7/398	306/22360	FBXL18 MSH6 TP53 PSMD9 CHEK2 APP TAOK1	0.3756	0.5899	1.29
GO:0070201	regulation of establishment of protein localization	biological_process	18/398	846/22360	PSMD9 F2R GAS6 SRCIN1 GSK3B VPS28 CYP51A1 AKT2 CX3CL1 TP53 CASP8 CCL5 RAB29 NLRP3 BCAS3 MYO1C C5AR2 CAMK1	0.3756	0.5899	1.2
GO:0008610	lipid biosynthetic process	biological_process	17/398	796/22360	A4GALT MYO5A PIK3R3 GPAT4 ACOT8 DGKZ FITM2 PLPP2 CYP51A1 PIGL FDPS PLEKHA2 MLXIPL B3GALT1 WNT4 KDSR MGLL	0.3763	0.5907	1.2
GO:0001655	urogenital system development	biological_process	8/398	354/22360	SOX11 TRAF3IP1 WNT4 SMAD2 RARG SMARCC1 ITGA3 PGF	0.3781	0.5933	1.27
GO:0005759	mitochondrial matrix	cellular_component	11/398	499/22360	TP53 DHX30 NDUFAF7 CS PPM1K PDE12 ATXN3 PDP2 NDUFA10 IBA57 NUBPL	0.3783	0.5935	1.24
GO:0009755	hormone-mediated signaling pathway	biological_process	6/398	260/22360	DDX54 NCOR1 RARG WBP2 KMT2D SSTR2	0.3793	0.5948	1.3
GO:0070382	exocytic vesicle	cellular_component	5/398	214/22360	APP SYP SYT12 RAB3B STX16	0.3799	0.5956	1.31
GO:0015718	monocarboxylic acid transport	biological_process	4/398	169/22360	NCOR1 P2RX4 AKT2 ABCC3	0.3811	0.5969	1.33
GO:0051223	regulation of protein transport	biological_process	17/398	798/22360	GSK3B VPS28 CX3CL1 AKT2 CYP51A1 PSMD9 F2R SRCIN1 GAS6 BCAS3 MYO1C C5AR2 CAMK1 TP53 NLRP3 RAB29 CCL5	0.3816	0.5975	1.2
GO:0030217	T cell differentiation	biological_process	6/398	261/22360	PKNOX1 CD3G CCR6 NLRP3 SPN WNT4	0.3841	0.6004	1.29
GO:0015931	nucleobase-containing compound transport	biological_process	6/398	261/22360	MCM3AP RFTN1 MYO1C SLC33A1 PNPT1 SLC35E2B	0.3841	0.6004	1.29
GO:0002237	response to molecule of bacterial origin	biological_process	9/398	404/22360	CX3CL1 IFNAR1 SBNO2 CASP8 SHPK F2R CNR2 NLRP3 CCL5	0.386	0.5999	1.25
GO:0051649	establishment of localization in cell	biological_process	48/398	2405/22360	SEC62 GSK3B DYNC2LI1 GAS6 APP DENND2A CORO7 ACOT8 ARFRP1 SNX27 STYX AKT2 P2RX4 VPS28 SRSF10 VPS52 RAB40B AP4M1 MYO1C SPTAN1 CACNB2 BCAS3 ZDHHC15 SYT12 TP53 ACOX1 RFTN1 TRAF3IP1 F2R SYNRG CAMK1 RABGAP1 COX5B STX16 COG1 VPS33A CASP8 RAB3B TBCK RAB22A CX3CL1 WHAMM RILPL1 MYO5A SYP AP4E1 RAB29 MCM3AP	0.3861	0.5999	1.12
GO:0051017	actin filament bundle assembly	biological_process	4/398	170/22360	CX3CL1 FHDC1 WNT4 ADD2	0.3871	0.6012	1.32

GO:0005515	protein binding	molecular_function	248/398	13214/22360	NLRP3 RNF166 RFC2 SYT12 MXD1 SPTAN1 XIAP CCDC36 TSEN2 MLXIPL PYGB ZC3H14 ARHGEF39 P2RX4 AKT2 FHDC1 MED22 DGKZ SLC12A2 ANKRD45 GNPTG C5AR2 SSTR2 MAPK12 CXCR4 PCGF5 EPN1 SLC9A7 KIAA1191 C9ORF78 COL8A1 RAB29 POU5F1 PKNOX1 MSH6 PGF ITGA3 HIF1AN SLC39A13 AP4E1 SPC25 SYP SMARCC1 SON RILPL1 OAS3 KLHL1 GABRB3 CX3CL1 LGMN CYB561A3 ADAR PRKAR2A MMS22L GRK5 SYNRG MR1 LRRC8B TTC3 TENM2 TNK2 SRCIN1 MNT HP DCUN1D2 ACOX1 HBE1 TP53 ABCC9 CALCRL APOL1 TMOD3 MAPKAPK5 TONSL NUDT22 AP4M1 MAP3K2 MTAP CCL5 ACOT8 ZNF772 BTNL9 APP RNF7 NCOR1 BCL7B GSK3B GCLC ACSL6 COX6B2 ICA1L SCYL3 SMCR8 WBP2 CTSS NMNAT1 PRR3 MYO5A RRP1 BBS1 ZFPM2 LRPAP1 YES1 OTUD4 RAB22A TOR1AIP2 DHX30 SUMO2 ADD2 CASP8 SULT1B1 NDUFAF7 COG1 NSRP1 COX5B SMAD2 MKNK1 LETMD1 SLC35F6 TAOK1 ATXN3 PTPRM CHEK2 KMT2D PPM1K BCAS3 ICOSLG TRIM72 NPAT UBXN2A TIE1 ZNF106 VPS52 CARD19 SRSF10 ATP2B1 EIF2AK2 TCF4 ASPA HNRNPUL1 PLPP2 SNX27 COQ6 DDX54 ZKSCAN1 MGLL IRAK4 USP28 TMEM170A CNKSR3 DESI1 GAS6 KIAA1958 ESYT3 CD3G SRGAP2B IKZF3 MARVELD2 RARG BRDT WNT4 PDE4C STAC2 PLEKHA2 C1ORF174 KRT18 ZMYM5 DDI2 PNPT1 RGS17 C19ORF66 IFNAR1 DPP9 RSPH3 TAF8 TRABD2B TBCK RAB3B ZHX3 VPS33A DIS3L MLX ANO6 STX16 TMEM106B CAMK1 TTYH1 CSF2RA RABGAP1 CRX F2R TRAF3IP1 FARP1 ERMAP METTL21A MED17 CACNB2 MYO1C DRG1 SCAI PIK3R3 SYAP1 FAM161A FGD5 PSMD9 EYA3 VPS28 PEAK1 PSTPIP2 STYX UBE3C CCR4 PLEKHB2 RNF115 NAGK DNAL1 RPRD1B ARFRP1 CORO7 RCAN1 MAP3K9 CIART DENND2A TRIM25 QPRT DYNC2LI1 BTN3A2 PPP1R37 GNS AAK1 CCR6 INTS6 ISG20L2 ADCY2 LTBR ZNF274 WHAMM KCNN3 NOA1 KCNJ15 DUOX1 DCTD USP44 MDM4 TTLL7 N4BP2L2 SPN ARNT2	0.3891	0.6038	1.05
GO:0051098	regulation of binding	biological_process	9/398	405/22360	SOX11 LRPAP1 ADD2 STYX CAMK1 SMAD2 APP SYAP1 GSK3B	0.3898	0.6047	1.25
GO:0032386	regulation of intracellular transport	biological_process	10/398	454/22360	TP53 RAB29 GAS6 CACNB2 MYO1C BCAS3 GSK3B AKT2 CAMK1 SYP	0.3915	0.6057	1.24
GO:0019752	carboxylic acid metabolic process	biological_process	24/398	1158/22360	GCLC ACSL6 ACOX1 NCOR1 QPRT MGLL PDP2 ITIH5 ACOT8 NAGK ASPA AKT2 MTAP VNN2 PGM2L1 UEVLD MLXIPL PSMD9 CYP4V2 AS3MT MYO5A GPAT4 PPM1K NMNAT1	0.3918	0.606	1.16
GO:0045216	cell-cell junction organization	biological_process	4/398	171/22360	MYO1C GJC1 F2R MARVELD2	0.3932	0.608	1.31
GO:0032412	regulation of ion transmembrane transporter activity	biological_process	6/398	263/22360	CTSS APP MYO5A CNKSR3 CACNB2 STAC2	0.3937	0.6086	1.28
GO:0016324	apical plasma membrane	cellular_component	8/398	358/22360	ATP6V1A SLC12A2 SLC9A4 CNKSR3 SLC26A2 DUOX1 ATP2B1 MARVELD2	0.3943	0.6093	1.26
GO:0005856	cytoskeleton	cellular_component	47/398	2358/22360	YES1 INTS6 RILPL1 BBS1 RSPH3 WHAMM RAB22A CC2D2A KRT18 KLHL1 ODF2L RAB29 MYO5A SRCIN1 TRAF3IP1 FARP1 TAOK1 TTLL7 PRKAR2A DIS3L ADD2 TBCCD1 TBCK CASP8 RABGAP1 TTI2 TSEN2 FGD5 FAM161A FHDC1 PSTPIP2 EYA3 PEAK1 BCAS3 MYO1C SPTAN1 TMOD3 DYNC2LI1 PCGF5 TTLL3 CEP112 GSK3B NCOR1 DNAL1 RPRD1B DENND2A APP	0.3947	0.6097	1.12

GO:0010467	gene expression	biological_process	132/398	6913/22360	RRP1 WBP2 CTSS SMCR8 MCM3AP ZNF669 ZNF274 TRMT10B ZNF70 PCSK7 ISG20L2 YES1 INTS6 ZNF445 ZFPM2 MKNK1 SMAD2 SAMD4B NSRP1 NOA1 CASP8 SUMO2 ZNF677 ARNT2 KMT2D ZNF430 CHEK2 ZNF789 N4BP2L2 MDM4 ZNF345 ZNF573 TONSL C2ORF49 SCAI PIK3R3 DRG1 MED17 MYO1C MAPKAPK5 PDE12 ZBTB37 TP53 ZNF264 ZNF570 MAP3K2 C1ORF61 ZNF7 SBNO2 OGFOD1 PSMD9 TMTC1 APP CIART RCAN1 ZNF441 ZNF772 RPRD1B CCL5 GCLC GSK3B NCOR1 ZNF483 SOX11 TRIM25 ZNF286B BRDT FOXD2 SMARCC1 RARG HIF1AN ITGA3 IKZF3 PKNOX1 POU5F1 CX3CL1 TAF8 PNPT1 DDI2 ZMYM5 SON ZNF80 WNT4 GMEB1 FTO CAMK1 MLX SNORA13 ZHX3 ADAR DDX51 LGMN ZNF766 ZNF583 MNT TENM2 F2R CRX XIAP ZNF814 SLC33A1 EXOSC6 BCAS3 CWC25 MXD1 OR7D2 NLRP3 RIPPLY3 ZNF8 MED22 HNRNPUL1 AKT2 TCF4 EIF2AK2 SRSF10 ZC3H14 MIR654 UBXN2A MLXIPL TSEN2 NPAT MAPK12 ZNF800 ZKSCAN1 DDX54 MTFMT RPL7L1 C9ORF78 GAS6 FCF1 PCGF5 IRAK4	0.3967	0.6119	1.07
GO:0032970	regulation of actin filament-based process	biological_process	9/398	407/22360	ADD2 WNT4 WHAMM CX3CL1 TAOK1 BCAS3 MYO1C SPTAN1 TMOD3	0.3974	0.6106	1.24
GO:0006399	tRNA metabolic process	biological_process	5/398	218/22360	TRMT10B MTFMT TSEN2 SLFN13 C2ORF49	0.4012	0.615	1.29
GO:0007163	establishment or maintenance of cell polarity	biological_process	5/398	218/22360	CD3G GSK3B SNX27 SPN BCAS3	0.4012	0.615	1.29
GO:0032446	protein modification by small protein conjugation	biological_process	21/398	1009/22360	TRIM72 XIAP NXN KLHL8 FBXL18 RNF115 SUMO2 UBE3C GCLC VPS28 DCUN1D2 RAB40B HERC4 RNF7 UBXN2A TTC3 USP44 FBXL4 TRIM25 PCGF5 PSMD9	0.4023	0.6154	1.17
GO:0070925	organelle assembly	biological_process	20/398	958/22360	RILPL1 DYNC2LI1 TRAF3IP1 UBXN2A OGFOD1 BBS1 FAM161A FHDC1 TTLL3 CHEK2 ODF2L VPS28 CC2D2A NCOR1 DNAL1 SMCR8 DHX30 RABGAP1 DRG1 TMOD3	0.4024	0.6154	1.17
GO:0006814	sodium ion transport	biological_process	6/398	265/22360	P2RX4 ANO6 SLC9A7 SLC12A2 CNKSR3 SLC9A4	0.4033	0.6166	1.27
GO:0006259	DNA metabolic process	biological_process	21/398	1010/22360	C9ORF84 TRIM25 USP28 CHEK2 OTUD4 EYA3 ATXN3 TAOK1 NMNAT1 LIG1 MSH6 RFC2 TP53 APOBEC3D MMS22L CCR6 CCDC36 FTO TONSL EXOSC6 MAPKAPK5	0.4047	0.6185	1.17
GO:0007033	vacuole organization	biological_process	4/398	173/22360	UBXN2A SMCR8 TMEM106B VPS33A	0.4054	0.6194	1.3
GO:0042254	ribosome biogenesis	biological_process	7/398	313/22360	ISG20L2 FCF1 DHX30 RPL7L1 DDX51 RRP1 EXOSC6	0.4064	0.6208	1.26
GO:0008654	phospholipid biosynthetic process	biological_process	7/398	313/22360	DGKZ FDPS PIGL FITM2 PLEKHA2 PIK3R3 GPAT4	0.4064	0.6208	1.26
GO:0035725	sodium ion transmembrane transport	biological_process	5/398	219/22360	SLC9A7 ANO6 SLC9A4 CNKSR3 SLC12A2	0.4066	0.6207	1.28
GO:0002684	positive regulation of immune system process	biological_process	29/398	1424/22360	PSMD9 YES1 P2RX4 OTUD4 ERMAP BTN3A2 CD3G RAB29 CTSS NLRP3 MSH6 PGF EXOSC6 ICOSLG MYO1C CCR6 XIAP IRAK4 GAS6 RFTN1 N4BP2L2 SPN LGMN CASP8 CCL5 ANO6 BTNL9 C5AR2 APP	0.4088	0.6239	1.14
GO:0050769	positive regulation of neurogenesis	biological_process	11/398	508/22360	TMEM106B CAMK1 ASPA CX3CL1 APP P2RX4 TCF4 ITGA3 SOX11 CXCR4 CRX	0.4091	0.6234	1.22
GO:0070371	ERK1 and ERK2 cascade	biological_process	8/398	362/22360	C5AR2 APP STYX CX3CL1 F2R CNKSR3 CCL5 GAS6	0.4109	0.6209	1.24
GO:0045321	leukocyte activation	biological_process	30/398	1477/22360	CTSS RAB29 CD3G NLRP3 GNS MSH6 PKNOX1 IKZF3 SHPK CCR6 EXOSC6 SPTAN1 ICOSLG YES1 WNT4 PYGB SBNO2 CX3CL1 LTBR CCL5 PIGR SNX27 CASP8 APP ANO6 GAS6 SOX11 CNR2 HP SPN	0.4109	0.6209	1.14
GO:0006937	regulation of muscle contraction	biological_process	4/398	174/22360	SSTR2 P2RX4 F2R CALCRL	0.4115	0.6214	1.29
GO:0035304	regulation of protein dephosphorylation	biological_process	4/398	174/22360	EIF2AK2 RCAN1 GSK3B PPP1R37	0.4115	0.6214	1.29

GO:0061572	actin filament bundle organization	biological_process	4/398	174/22360	ADD2 WNT4 CX3CL1 FHDC1	0.4115	0.6214	1.29
GO:0050954	sensory perception of mechanical stimulus	biological_process	4/398	174/22360	LRTOMT MARVELD2 P2RX4 GABRB3	0.4115	0.6214	1.29
GO:0071347	cellular response to interleukin-1	biological_process	5/398	220/22360	CCL5 OTUD4 CX3CL1 PSMD9 IRAK4	0.4121	0.6216	1.28
GO:0033209	tumor necrosis factor-mediated signaling pathway	biological_process	5/398	220/22360	PSMD9 LTBR KRT18 CASP8 GAS6	0.4121	0.6216	1.28
GO:2000116	regulation of cysteine-type endopeptidase activity	biological_process	6/398	267/22360	NLRP3 XIAP GAS6 LGMN CASP8 F2R	0.4131	0.6227	1.26
GO:0046649	lymphocyte activation	biological_process	17/398	811/22360	WNT4 SOX11 GAS6 YES1 SPN CASP8 SNX27 PKNOX1 MSH6 IKZF3 CCL5 CD3G RAB29 NLRP3 EXOSC6 ICOSLG CCR6	0.4168	0.6281	1.18
GO:0000987	proximal promoter sequence-specific DNA binding	molecular_function	12/398	560/22360	TCF4 ARNT2 SOX11 GMEB1 CRX MLXIPL SMARCC1 SMAD2 CIART WBP2 MXD1 TP53	0.4175	0.629	1.2
GO:0007269	neurotransmitter secretion	biological_process	4/398	175/22360	CACNB2 GSK3B SYT12 SYP	0.4177	0.6291	1.28
GO:0002429	immune response-activating cell surface receptor signaling pathway	biological_process	11/398	511/22360	C5AR2 MYO1C ICOSLG BTNL9 RFTN1 YES1 RAB29 CD3G BTN3A2 PSMD9 ERMAP	0.4196	0.6305	1.21
GO:0051248	negative regulation of protein metabolic process	biological_process	27/398	1326/22360	ACOT8 PRKAR2A ADAR NXN ITIH5 PAPLN SAMD4B FTO APP USP44 MDM4 GAS6 SRCIN1 TRAF3IP1 CNKSR3 GSK3B NCOR1 CYP51A1 GCLC PPP1R37 SMCR8 XIAP SMARCC1 MLXIPL VPS28 STYX EIF2AK2	0.4207	0.632	1.14
GO:0006473	protein acetylation	biological_process	5/398	222/22360	CAMK1 WBP2 TAOK1 FAM161A GSK3B	0.423	0.6319	1.27
GO:0042060	wound healing	biological_process	13/398	612/22360	HBE1 P2RX4 CX3CL1 WNT4 ZFPM2 F2R GAS6 ANO6 DUOX1 TRIM72 SMAD2 PRKAR2A DGKZ	0.4244	0.6327	1.19
GO:0030522	intracellular receptor signaling pathway	biological_process	7/398	317/22360	CASP8 WBP2 DDX54 NCOR1 XIAP RARG KMT2D	0.4245	0.6327	1.24
GO:0034613	cellular protein localization	biological_process	41/398	2061/22360	ARFRP1 ACOT8 CASP8 RAB3B PIGR TOR1AIP2 SNX27 TBCK CAMK1 SYNRG RABGAP1 STX16 DYNC2LI1 TRAF3IP1 GAS6 SEC62 ACOX1 RFTN1 GSK3B RAB29 TP53 MCM3AP MYO5A AP4E1 MYO1C CACNB2 BCAS3 ITGA3 ZDHHC15 AP4M1 RILPL1 STAC2 BBS1 AKT2 STYX RAB22A TAF8 VPS28 KRT18 RAB40B CC2D2A	0.426	0.6348	1.12
GO:0007596	blood coagulation	biological_process	8/398	366/22360	ANO6 P2RX4 HBE1 F2R ZFPM2 PRKAR2A DGKZ GAS6	0.4277	0.6356	1.23
GO:0010631	epithelial cell migration	biological_process	8/398	366/22360	LGMN EMC10 BCAS3 ITGA3 PTPRM PIK3R3 CCR6 P2RX4	0.4277	0.6356	1.23
GO:0022898	regulation of transmembrane transporter activity	biological_process	6/398	270/22360	CACNB2 MYO5A APP STAC2 CNKSR3 CTSS	0.4279	0.6356	1.25
GO:0030073	insulin secretion	biological_process	5/398	223/22360	SMAD2 MYO5A CCL5 CYB5R4 PSMD9	0.4285	0.6363	1.26
GO:0051648	vesicle localization	biological_process	7/398	318/22360	VPS33A SYT12 GSK3B CACNB2 MYO1C SYP MYO5A	0.429	0.6368	1.24
GO:0043005	neuron projection	cellular_component	28/398	1383/22360	KCNN3 CX3CL1 P2RX4 ATP2B1 RGS17 KLHL1 GABRB3 ADCY2 ZC3H14 BAALC FAM161A MYO5A SYP SYAP1 MYO1C ITGA3 AAK1 FARP1 RGS12 SEZ6 TTLL7 GSK3B CNR2 SRCIN1 TENM2 APP PCDH9 CASP8	0.4316	0.6402	1.14
GO:0009306	protein secretion	biological_process	14/398	665/22360	CX3CL1 CYP51A1 SRCIN1 GAS6 F2R PSMD9 SMAD2 MYO5A C5AR2 CYB5R4 NLRP3 CCL5 RAB3B BTN3A2	0.4333	0.6425	1.18
GO:0009410	response to xenobiotic stimulus	biological_process	7/398	319/22360	CNR2 SULT1B1 GCLC CHEK2 AS3MT ARNT2 RGS17	0.4336	0.6428	1.23
GO:0005635	nuclear envelope	cellular_component	11/398	515/22360	RRP12 TMEM170A TOR1AIP2 MCM3AP P2RX4 APP SCAI GRK5 MYO1C MLX RAB40B	0.4337	0.6427	1.2

GO:0006936	muscle contraction	biological_process	8/398	368/22360	F2R TRIM72 SSTR2 P2RX4 GJC1 TMOD3 CACNB2 CALCRL	0.4362	0.6431	1.22
GO:0002253	activation of immune response	biological_process	17/398	818/22360	BTNL9 ICOSLG MYO1C C5AR2 XIAP LGMN ERMAP CASP8 BTN3A2 CTSS RAB29 CD3G RFTN1 OTUD4 IRAK4 PSMD9 YES1	0.4363	0.6431	1.17
GO:0000226	microtubule cytoskeleton organization	biological_process	13/398	616/22360	SPC25 DRG1 BCAS3 DNAL1 CHEK2 TTLL3 NCOR1 TAOK1 ATXN3 GSK3B CC2D2A TRAF3IP1 SON	0.4374	0.6445	1.19
GO:0048514	blood vessel morphogenesis	biological_process	15/398	717/22360	COL8A1 PKNOX1 PIK3R3 PGF BCAS3 HIF1AN CALCRL GJC1 WNT4 EPN1 ZFPM2 TIE1 EMC10 CX3CL1 PTPRM	0.4379	0.6451	1.18
GO:0090132	epithelium migration	biological_process	8/398	369/22360	LGMN EMC10 P2RX4 PIK3R3 CCR6 ITGA3 BCAS3 PTPRM	0.4405	0.6485	1.22
GO:0070848	response to growth factor	biological_process	16/398	769/22360	WNT4 SOX11 YES1 GAS6 GCLC LGMN ZNF8 CCL5 SLC33A1 MYO1C ITGA3 PGF SMAD2 SYAP1 XIAP APP	0.4417	0.6501	1.17
GO:1903827	regulation of cellular protein localization	biological_process	12/398	568/22360	CASP8 TP53 RAB29 MYO1C ITGA3 BCAS3 CAMK1 STAC2 GAS6 VPS28 GSK3B AKT2	0.4446	0.6542	1.19
GO:0099572	postsynaptic specialization	cellular_component	8/398	370/22360	ADD2 BAALC SRCIN1 DGKZ YES1 TANC2 P2RX4 CAMK1	0.4448	0.6543	1.21
GO:0015276	ligand-gated ion channel activity	molecular_function	5/398	226/22360	MYO5A APP P2RX4 KCNJ15 GABRB3	0.4451	0.6545	1.24
GO:0044212	transcription regulatory region DNA binding	molecular_function	21/398	1027/22360	NCOR1 KMT2D ARNT2 TCF4 GMEB1 MLXIPL PCGF5 CRX SOX11 MNT CIART MLX SMAD2 RARG SMARCC1 TP53 POU5F1 MXD1 IKZF3 ZHX3 WBP2	0.4469	0.6566	1.15
GO:0051493	regulation of cytoskeleton organization	biological_process	12/398	569/22360	WHAMM CX3CL1 TAOK1 GSK3B TRAF3IP1 WNT4 BCAS3 TMOD3 SPTAN1 MYO1C DRG1 ADD2	0.4481	0.6582	1.18
GO:0050728	negative regulation of inflammatory response	biological_process	4/398	180/22360	CX3CL1 CNR2 NLRP3 CALCRL	0.449	0.6569	1.25
GO:1903034	regulation of response to wounding	biological_process	4/398	180/22360	ANO6 F2R WNT4 DUOX1	0.449	0.6569	1.25
GO:1903305	regulation of regulated secretory pathway	biological_process	4/398	180/22360	GSK3B CACNB2 SYP SYT12	0.449	0.6569	1.25
GO:0000287	magnesium ion binding	molecular_function	5/398	227/22360	ADCY2 MAPK12 MSH6 GCLC IRAK4	0.4507	0.6588	1.24
GO:0051346	negative regulation of hydrolase activity	biological_process	11/398	520/22360	FARP1 XIAP APP ITIH5 GSK3B PAPLN BCAS3 GAS6 PPP1R37 TNK2 TP53	0.4517	0.6601	1.19
GO:0005261	cation channel activity	molecular_function	11/398	520/22360	STAC2 GAS6 CTSS KCNJ15 ABCC9 CACNB2 ANO6 MYO5A APP P2RX4 KCNN3	0.4517	0.6601	1.19
GO:0050817	coagulation	biological_process	8/398	372/22360	HBE1 P2RX4 ANO6 PRKAR2A DGKZ GAS6 F2R ZFPM2	0.4534	0.6622	1.21
GO:0007599	hemostasis	biological_process	8/398	372/22360	P2RX4 HBE1 ANO6 GAS6 DGKZ PRKAR2A ZFPM2 F2R	0.4534	0.6622	1.21
GO:0006888	endoplasmic reticulum to Golgi vesicle-mediated transport	biological_process	5/398	228/22360	SPTAN1 COG1 WHAMM RAB29 GAS6	0.4563	0.6655	1.23
GO:0043177	organic acid binding	molecular_function	5/398	228/22360	HIF1AN OGFOD1 ACOX1 GCLC HBE1	0.4563	0.6655	1.23

GO:0050794	regulation of cellular process	biological_process	229/398	12274/22360	SNX27 DDX54 ITIH5 ZKSCAN1 CNKSR3 GAS6 MGLL IRAK4 USP28 OR7D2 RIPPLY3 ZNF814 TRIM72 BCAS3 ICOSLG NPAT TIE1 ZNF106 UBXN2A EIF2AK2 TCF4 ASPA CARD19 RAB40B SRSF10 ZHX3 TBCK RAB3B FTO TMEM106B CAMK1 CSF2RA RABGAP1 GPR155 MLX PAPLN ANO6 TRAF3IP1 CRX F2R FARP1 CD3G SRGAP2B IKZF3 RARG ZNF286B BRDT WNT4 STAC2 ZNF80 PDE4C IFNAR1 TAF8 TRABD2B ZMYM5 KRT18 CC2D2A PNPT1 RGS17 RPRD1B ARFRP1 CCR4 PLEKHB2 RNF115 MAP3K9 ZNF441 RCAN1 PDP2 CIART NXN CCDC125 DYNC2LI1 TRIM25 ERMAP SCAI PIK3R3 SYAP1 DRG1 CACNB2 MED17 MYO1C ZNF7 C1ORF61 FAM161A FGD5 PSMD9 SBNO2 STYX EYA3 VPS28 PEAK1 NOA1 TBCCD1 DUOX1 PRR11 CNR2 MDM4 ZNF573 USP44 ZNF345 SEZ6 ARNT2 ZNF430 RFTN1 N4BP2L2 SPN ZNF789 PPP1R37 BTN3A2 CCR6 AAK1 ZNF445 ADCY2 WHAMM LTBR ZNF274 DGKZ SLC12A2 FITM2 C5AR2 SSTR2 MAPK12 ZNF800 PCGF5 EPN1 CXCR4 CYP51A1 C9ORF78 NLRP3 RFC2 MXD1 SYT12 ZNF8 SHPK XIAP CCDC36 EXOSC6 SLC33A1 SPTAN1 ZC3H14 MLXIPL P2RX4 AKT2 MED22 ARHGEF39 ADAR PRKAR2A ZNF766 LGMN GRK5 SRCIN1 MNT TENM2 TNK2 ZNF583 HP DCUN1D2 RAB29 POU5F1 MSH6 PKNOX1 SMARCC1 SYP FOXD2 PGF ITGA3 HIF1AN OAS3 RILPL1 GMEB1 SON CX3CL1 GABRB3 CCL5 ACOT8 PIGR APP ZNF772 BTNL9 SOX11 ZNF483 EMC10 GCLC RGS12 BCL7B NCOR1 GSK3B ZBTB37 PDE12 ATP6V1A TP53 TONSL CALCRL MAPKAPK5 TMOD3 OGFOD1 MTAP ZNF264 MAP3K2 ZNF570 ZNF677 SUMO2 ADD2 CASP8 SAMD4B MKNK1 SMAD2 NSRP1 SLC35F6 CHEK2 KMT2D TAOK1 ATXN3 PTPRM FBXL18 WBP2 CTSS NMNAT1 SMCR8 APOBEC3D MYO5A YES1 LRPAP1 ZNF70 ZFPM2 OTUD4 ZNF669 RAB22A ODF2L	0.4571	0.6663	1.05
GO:0030030	cell projection organization	biological_process	33/398	1657/22360	RILPL1 BBS1 FAM161A FGD5 P2RX4 WHAMM CX3CL1 FHDC1 CC2D2A KLHL1 ODF2L RAB29 BCAS3 ITGA3 SPTAN1 SRCIN1 TRAF3IP1 DYNC2LI1 CXCR4 TENM2 TTLL3 SEZ6 FARP1 GSK3B PTPRM DNAL1 LGMN APP RABGAP1 TTYH1 TMEM106B CAMK1 ANO6	0.4576	0.6669	1.12
GO:0016607	nuclear speck	cellular_component	10/398	472/22360	SON FBXL4 ZNF106 DGKZ ZC3H14 CWC25 NSRP1 SRSF10 GRK5 FTO	0.4586	0.6681	1.19
GO:0006396	RNA processing	biological_process	30/398	1501/22360	PNPT1 SRSF10 TRMT10B HNRNPUL1 TMTC1 TSEN2 SON ZC3H14 INTS6 ISG20L2 CWC25 EXOSC6 C2ORF49 BRDT RRP1 TP53 PDE12 NCOR1 C9ORF78 FCF1 DDX54 NSRP1 APP SMAD2 RPL7L1 DDX51 MTFMT ADAR RPRD1B SNORA13	0.4605	0.6707	1.12
GO:0043235	receptor complex	cellular_component	9/398	423/22360	TIE1 PIGR NT5DC3 CD3G CALCRL GABRB3 ITGA3 CSF2RA APP	0.4609	0.6705	1.2
GO:0042063	gliogenesis	biological_process	7/398	325/22360	P2RX4 APP AKT2 ASPA CX3CL1 SOX11 CXCR4	0.4614	0.6688	1.21
GO:0030162	regulation of proteolysis	biological_process	17/398	827/22360	GCLC OTUD4 STYX GSK3B ATXN3 GAS6 F2R XIAP SMARCC1 APP MAPK12 ITIH5 PAPLN NLRP3 LGMN SUMO2 CASP8	0.4619	0.6693	1.15
GO:0022843	voltage-gated cation channel activity	molecular_function	5/398	229/22360	KCNJ15 APP GAS6 STAC2 CACNB2	0.462	0.6693	1.23
GO:0010941	regulation of cell death	biological_process	39/398	1976/22360	CD3G RAB29 NMNAT1 NLRP3 PDE12 IKZF3 TP53 RARG XIAP LRPAP1 RILPL1 WNT4 ZFPM2 SON EIF2AK2 AKT2 CX3CL1 KRT18 GABRB3 LTBR EYA3 NOA1 CCL5 LGMN CASP8 GRK5 MAP3K9 ANO6 GAS6 MNT SOX11 MDM4 F2R SLC35F6 CHEK2 GCLC ARNT2 HP GSK3B	0.4627	0.6701	1.11
GO:1903362	regulation of cellular protein catabolic process	biological_process	6/398	277/22360	STYX GCLC SMARCC1 SUMO2 GSK3B ATXN3	0.4633	0.6708	1.22
GO:0032787	monocarboxylic acid metabolic process	biological_process	14/398	675/22360	ACOX1 NCOR1 AKT2 PGM2L1 VNN2 ACSL6 MGLL MLXIPL PDP2 GPAT4 AS3MT CYP4V2 MYO5A ACOT8	0.465	0.6723	1.17
GO:0010001	glial cell differentiation	biological_process	5/398	230/22360	CXCR4 APP AKT2 SOX11 ASPA	0.4676	0.6759	1.22
GO:0031256	leading edge membrane	cellular_component	4/398	183/22360	MYO1C FGD5 AKT2 ATP2B1	0.4681	0.6764	1.23

GO:0060401	cytosolic calcium ion transport	biological_process	4/398	183/22360	MYO5A P2RX4 CX3CL1 F2R	0.4681	0.6764	1.23
GO:1990837	sequence-specific double-stranded DNA binding	molecular_function	19/398	932/22360	TP53 MXD1 IKZF3 POU5F1 WBP2 ZHX3 CIART MLX SMAD2 RARG SMARCC1 MLXIPL PCGF5 CRX GMEB1 SOX11 MNT ARNT2 TCF4	0.4682	0.6762	1.15
GO:0030098	lymphocyte differentiation	biological_process	8/398	376/22360	SPN CCR6 WNT4 NLRP3 GAS6 CD3G PKNOX1 IKZF3	0.4708	0.6796	1.2
GO:0042592	homeostatic process	biological_process	41/398	2087/22360	SLC9A4 PRKAR2A CCL5 VPS33A SLC12A2 PIGR CCR4 FITM2 STEAP2 C5AR2 APP FTO NXN ANO6 GAS6 CXCR4 LRRC8B F2R GCLC SLC9A7 TAOK1 RABGAP1 ACOX1 ATP6V1A PKNOX1 VSIG1 RFC2 XIAP MYO5A CCR6 CYB5R4 CACNB2 MAPKAPK5 TMOD3 SLC39A13 ADCY2 MLXIPL BBS1 P2RX4 ATP2B1 CX3CL1	0.4721	0.6813	1.1
GO:0048469	cell maturation	biological_process	4/398	184/22360	GPAT4 APP CCR6 CX3CL1	0.4745	0.6809	1.22
GO:0002521	leukocyte differentiation	biological_process	12/398	579/22360	APP CCR6 LTBR SPN IKZF3 PKNOX1 CD3G NLRP3 GAS6 CASP8 SBNO2 WNT4	0.4831	0.6925	1.16
GO:0004197	cysteine-type endopeptidase activity	molecular_function	8/398	379/22360	CTSS NLRP3 GAS6 LGMN USP28 F2R CASP8 XIAP	0.4841	0.6934	1.19
GO:0019439	aromatic compound catabolic process	biological_process	14/398	681/22360	SLFN13 MTAP PNPT1 LRTOMT ZC3H14 PDE4C PSMD9 FTO SAMD4B APOBEC3D EXOSC6 DIS3L PDE12 NMNAT1	0.4845	0.6937	1.15
GO:0034702	ion channel complex	cellular_component	7/398	330/22360	TTYH1 GABRB3 ANO6 CACNB2 ABCC9 LRRC8B CLCC1	0.4851	0.6944	1.19
GO:0030324	lung development	biological_process	4/398	186/22360	ITGA3 ZFPM2 SMAD2 SOX11	0.4874	0.6948	1.21
GO:0019221	cytokine-mediated signaling pathway	biological_process	20/398	991/22360	OAS3 GAS6 TRIM25 IRAK4 CXCR4 PSMD9 OTUD4 MTAP CX3CL1 IFNAR1 LTBR KRT18 ADAR CCL5 CASP8 TP53 CCR4 DUOX1 CSF2RA CCR6	0.4881	0.6956	1.13
GO:0071396	cellular response to lipid	biological_process	14/398	683/22360	YES1 ADCY2 SBNO2 ATP2B1 CX3CL1 KMT2D NCOR1 CCL5 WBP2 NLRP3 SHPK RARG SSTR2 DDX54	0.491	0.6986	1.15
GO:2000146	negative regulation of cell motility	biological_process	8/398	381/22360	SRGAP2B CCDC125 WNT4 TIE1 SCAI C5AR2 CX3CL1 PTPRM	0.493	0.6999	1.18
GO:0031253	cell projection membrane	cellular_component	8/398	381/22360	ATP2B1 TTYH1 AKT2 SLC26A2 ITGA3 MYO1C FGD5 BBS1	0.493	0.6999	1.18
GO:0008284	positive regulation of cell population proliferation	biological_process	22/398	1098/22360	GAS6 SOX11 IRAK4 MLXIPL ZFPM2 F2R EMC10 SLC35F6 AKT2 KMT2D ARNT2 CX3CL1 N4BP2L2 CCL5 RPRD1B LGMN C5AR2 GRK5 RARG PGF CALCRL ICOSLG	0.4961	0.7039	1.13
GO:0043281	regulation of cysteine-type endopeptidase activity involved in apoptotic process	biological_process	5/398	235/22360	GAS6 XIAP NLRP3 CASP8 F2R	0.4962	0.7039	1.2
GO:0050804	modulation of chemical synaptic transmission	biological_process	10/398	482/22360	CNR2 SYT12 LGMN RAB3B F2R SYP APP CX3CL1 GSK3B CACNB2	0.4977	0.7058	1.17
GO:0002758	innate immune response-activating signal transduction	biological_process	8/398	383/22360	IRAK4 LGMN PSMD9 CASP8 CTSS RFTN1 XIAP OTUD4	0.5019	0.7079	1.17
GO:0034341	response to interferon-gamma	biological_process	7/398	334/22360	MYO1C TRIM25 PDE12 OAS3 CX3CL1 CCL5 C19ORF66	0.5043	0.7111	1.18
GO:0004497	monooxygenase activity	molecular_function	4/398	189/22360	COQ6 CYP51A1 CYP4V2 CNR2	0.5069	0.7143	1.19
GO:0007584	response to nutrient	biological_process	5/398	237/22360	P2RX4 ATP2B1 GCLC GAS6 TRIM25	0.5078	0.7154	1.19
GO:0010594	regulation of endothelial cell migration	biological_process	5/398	237/22360	P2RX4 BCAS3 LGMN EMC10 PTPRM	0.5078	0.7154	1.19
GO:0005085	guanyl-nucleotide exchange factor activity	molecular_function	5/398	237/22360	FGD5 ARHGEF39 SMCR8 DENND2A FARP1	0.5078	0.7154	1.19
GO:0006898	receptor-mediated endocytosis	biological_process	7/398	335/22360	APOL1 HP AAK1 CALCRL APP TNK2 LRPAP1	0.5092	0.7168	1.17
GO:0043542	endothelial cell migration	biological_process	6/398	286/22360	PTPRM BCAS3 P2RX4 PIK3R3 EMC10 LGMN	0.51	0.7177	1.18
GO:0030323	respiratory tube development	biological_process	4/398	190/22360	ZFPM2 ITGA3 SMAD2 SOX11	0.5135	0.7189	1.18

GO:0003723	RNA binding	molecular_function	41/398	2110/22360	FDPS TRIM25 FCF1 CS RFTN1 RRP12 NOA1 ADAR DIS3L RPL7L1 SUMO2 DHX30 DDX51 IBA57 SAMD4B SMAD2 DDX54 NSRP1 SPATS2 ZC3H14 ISG20L2 OAS3 ZNF106 MIR654 SON EIF2AK2 C19ORF66 SLFN13 OTUD4 HNRNPUL1 KRT18 TRMT10B PNPT1 SRSF10 POU5F1 TP53 APOBEC3D MYO5A RRP1 EXOSC6 PRR3	0.5148	0.7198	1.09
GO:0000086	G2/M transition of mitotic cell cycle	biological_process	6/398	287/22360	TAOK1 PSMD9 CHEK2 APP FBXL18 MSH6	0.5153	0.7203	1.17
GO:0006839	mitochondrial transport	biological_process	6/398	287/22360	TP53 CASP8 SLC35F6 GSK3B PNPT1 COX5B	0.5153	0.7203	1.17
GO:0031668	cellular response to extracellular stimulus	biological_process	6/398	287/22360	GAS6 TP53 WNT4 ATP2B1 P2RX4 EIF2AK2	0.5153	0.7203	1.17
GO:0045785	positive regulation of cell adhesion	biological_process	10/398	487/22360	CX3CL1 GSK3B ITGA3 ICOSLG NLRP3 YES1 CCL5 COL8A1 WNT4 PLEKHA2	0.5177	0.723	1.15
GO:0004674	protein serine/threonine kinase activity	molecular_function	19/398	950/22360	PRKAR2A AAK1 MAP3K9 MAPKAPK5 GRK5 SYAP1 CAMK1 MKNK1 MAPK12 CXCR4 IRAK4 F2R TNK2 TAOK1 MAP3K2 GSK3B EIF2AK2 CHEK2 AKT2	0.5185	0.724	1.12
GO:0009611	response to wounding	biological_process	15/398	743/22360	GAS6 ZFPM2 F2R WNT4 CX3CL1 P2RX4 HBE1 POU5F1 PRKAR2A DGKZ NMNAT1 DUOX1 TRIM72 SMAD2 ANO6	0.5194	0.725	1.13
GO:0017157	regulation of exocytosis	biological_process	5/398	239/22360	SYP SYT12 GSK3B CACNB2 RAB3B	0.5195	0.725	1.18
GO:0035821	modification of morphology or physiology of other organism	biological_process	4/398	191/22360	EIF2AK2 CCL5 CASP8 APOL1	0.5201	0.7239	1.18
GO:0009966	regulation of signal transduction	biological_process	71/398	3725/22360	TRIM25 EPN1 IRAK4 MGLL CXCR4 EMC10 CNKSR3 GAS6 SOX11 CCDC125 RABGAP1 NCOR1 GSK3B GCLC RGS12 RNF115 CCL5 MAP3K9 RCAN1 NXN C5AR2 APP FGD5 PSMD9 ARHGEF39 MAP3K2 CARD19 EYA3 EIF2AK2 P2RX4 STYX TP53 NLRP3 RFC2 MAPKAPK5 MYO1C SCAI XIAP TRIM72 F2R SLC35F6 TRAF3IP1 MNT MDM4 TAOK1 CHEK2 FARP1 SEZ6 KMT2D LGMN TBCK CASP8 GRK5 SMAD2 WNT4 LRPAP1 LTBR RGS17 OTUD4 CX3CL1 TRABD2B SMCR8 RAB29 WBP2 NMNAT1 AAK1 ITGA3 PGF HIF1AN MYO5A SYP	0.5202	0.7238	1.07
GO:0006260	DNA replication	biological_process	6/398	288/22360	LIG1 MSH6 RFC2 CHEK2 MMS22L TONSL	0.5206	0.7242	1.17
GO:0006954	inflammatory response	biological_process	17/398	847/22360	XIAP APP CCR6 C5AR2 CALCRL ANO6 NLRP3 CCL5 CCR4 SHPK CX3CL1 HP CNR2 CXCR4 MGLL SBNO2 F2R	0.5212	0.7248	1.13
GO:0051726	regulation of cell cycle	biological_process	27/398	1371/22360	PRR11 GRK5 APP MAPK12 LGMN RPRD1B TAOK1 CHEK2 USP44 USP28 GAS6 MDM4 SOX11 MNT DRG1 TMOD3 CCDC36 TP53 FBXL18 MSH6 PNPT1 WHAMM AKT2 MLXIPL PSMD9 WNT4 SON	0.5228	0.7266	1.11
GO:0043549	regulation of kinase activity	biological_process	19/398	952/22360	TAOK1 MAP3K2 EIF2AK2 F2R CXCR4 ADCY2 GAS6 SRCIN1 MAPKAPK5 MAP3K9 CAMK1 PIK3R3 APP SYAP1 SMCR8 ADAR CCL5 DGKZ PRKAR2A	0.5243	0.7285	1.12
GO:1904018	positive regulation of vasculature development	biological_process	5/398	240/22360	CX3CL1 EMC10 TIE1 HIF1AN PGF	0.5253	0.7297	1.17
GO:0019932	second-messenger-mediated signaling	biological_process	10/398	489/22360	ADCY2 PRKAR2A CXCR4 CCR4 MYO5A P2RX4 CCR6 CALCRL GSK3B RCAN1	0.5257	0.7301	1.15
GO:2001235	positive regulation of apoptotic signaling pathway	biological_process	4/398	192/22360	CASP8 GSK3B LTBR TP53	0.5267	0.7289	1.17
GO:0030097	hemopoiesis	biological_process	20/398	1006/22360	RARG CCR6 APP TMOD3 IKZF3 PKNOX1 NLRP3 CD3G VPS33A CASP8 ADD2 KMT2D EIF2AK2 SPN N4BP2L2 LTBR GAS6 SBNO2 PSMD9 WNT4	0.5294	0.7317	1.12
GO:0001894	tissue homeostasis	biological_process	5/398	241/22360	P2RX4 VSIG1 PIGR BBS1 F2R	0.5312	0.734	1.17

GO:0097153	cysteine-type endopeptidase activity involved in apoptotic process	molecular_function	5/398	241/22360	F2R CASP8 XIAP GAS6 NLRP3	0.5312	0.734	1.17
GO:0006909	phagocytosis	biological_process	6/398	290/22360	ANO6 MYO1C XKR4 CD3G YES1 GAS6	0.5312	0.734	1.16
GO:0050663	cytokine secretion	biological_process	6/398	290/22360	CX3CL1 NLRP3 GAS6 C5AR2 BTN3A2 F2R	0.5312	0.734	1.16
GO:0017144	drug metabolic process	biological_process	13/398	644/22360	LRTOMT QPRT WNT4 MTAP HBE1 P2RX4 ACOX1 HP ACOT8 NAGK SULT1B1 DUOX1 COX5B	0.5327	0.7353	1.13
GO:0031965	nuclear membrane	cellular_component	7/398	340/22360	MLX GRK5 SCAI P2RX4 MCM3AP TOR1AIP2 RRP12	0.5336	0.7363	1.16
GO:0002696	positive regulation of leukocyte activation	biological_process	8/398	390/22360	APP ICOSLG EXOSC6 MSH6 YES1 NLRP3 GAS6 CCL5	0.5337	0.7362	1.15
GO:0031334	positive regulation of protein complex assembly	biological_process	6/398	291/22360	TP53 GSK3B DRG1 MYO1C WHAMM TRABD2B	0.5366	0.74	1.16
GO:0051641	cellular localization	biological_process	60/398	3145/22360	VPS28 SRSF10 RAB40B VPS52 AKT2 STYX P2RX4 AP4M1 MYO1C CACNB2 SPTAN1 ZDHHC15 BCAS3 TP53 SYT12 SEC62 GSK3B CEP112 DYNC2LI1 GAS6 CORO7 DENND2A APP SNX27 PIGR ARFRP1 ACOT8 CC2D2A KRT18 CX3CL1 RAB22A TAF8 WHAMM STAC2 BBS1 RILPL1 AP4E1 ITGA3 SYP MYO5A ESYT3 MCM3AP RAB29 ACOX1 RFTN1 F2R TRAF3IP1 STX16 RABGAP1 SYNRG CAMK1 TMEM106B COX5B RAB3B CASP8 TBCCD1 TBCK TOR1AIP2 COG1 VPS33A	0.5411	0.7413	1.07
GO:0002768	immune response-regulating cell surface receptor signaling pathway	biological_process	12/398	595/22360	CD3G RAB29 YES1 PIGR PSMD9 ERMAP BTN3A2 C5AR2 RFTN1 BTNL9 ICOSLG MYO1C	0.5412	0.7412	1.13
GO:0043491	protein kinase B signaling	biological_process	6/398	292/22360	GAS6 CCL5 P2RX4 PIK3R3 CX3CL1 AKT2	0.5419	0.742	1.15
GO:0007165	signal transduction	biological_process	131/398	7008/22360	ANO6 GRK5 CAMK1 CSF2RA GPR155 TBCK LGMN RAB3B ADAR PRKAR2A FARP1 F2R TENM2 TNK2 TRAF3IP1 MNT PGF ITGA3 HIF1AN RARG SYP SMARCC1 CD3G RAB29 MSH6 KRT18 GABRB3 CC2D2A RGS17 IFNAR1 TRABD2B CX3CL1 WNT4 PDE4C STAC2 OAS3 DDX54 C5AR2 SSTR2 MAPK12 SNX27 DGKZ SLC12A2 CXCR4 MGLL IRAK4 EPN1 USP28 CNKSR3 GAS6 SPTAN1 ICOSLG SLC33A1 XIAP TRIM72 ZNF8 NLRP3 OR7D2 RFC2 ARHGEF39 CARD19 RAB40B P2RX4 EIF2AK2 AKT2 MLXIPL TIE1 ZNF106 DUOX1 SMAD2 MKNK1 CASP8 RFTN1 TAOK1 SPN PTPRM CHEK2 KMT2D SEZ6 SLC35F6 MDM4 CNR2 AAK1 CCR6 MYO5A SMCR8 BTN3A2 WBP2 CTSS NMNAT1 LTBR OTUD4 RAB22A LRPAP1 YES1 ADCY2 RCAN1 MAP3K9 BTNL9 NXN APP PIGR CCR4 RNF115 CCL5 ARFRP1 NCOR1 RABGAP1 BCL7B GSK3B GCLC RGS12 TRIM25 EMC10 CCDC125 SOX11 CALCRL MAPKAPK5 MYO1C SCAI PIK3R3 SYAP1 TP53 ERMAP ATP6V1A EYA3 MAP3K2 MTAP STYX FGD5 PSMD9	0.5431	0.7435	1.05
GO:0051099	positive regulation of binding	biological_process	4/398	195/22360	SYAP1 APP GSK3B ADD2	0.5465	0.7475	1.15
GO:0001959	regulation of cytokine-mediated signaling pathway	biological_process	4/398	195/22360	CASP8 GAS6 CCL5 OTUD4	0.5465	0.7475	1.15
GO:0006629	lipid metabolic process	biological_process	30/398	1540/22360	LGMN PLPP2 FITM2 SULT1B1 DGKZ ACOT8 PDP2 APP A4GALT MGLL B3GALT1 SUMF2 FDPS NCOR1 ACOX1 ACSL6 CYP51A1 ESYT3 GPAT4 APOL1 PIK3R3 MYO5A CYP4V2 KDSR WNT4 MLXIPL PLEKHA2 PIGL GK5 AKT2	0.5466	0.7473	1.09
GO:0008219	cell death	biological_process	49/398	2556/22360	MSH6 IKZF3 NMNAT1 RAB29 CD3G RARG RILPL1 LRPAP1 SON ZFPM2 WNT4 GSDMB CX3CL1 LTBR KRT18 GABRB3 NOA1 CASP8 LGMN GRK5 ANO6 MDM4 MNT SLC35F6 F2R ARNT2 GSDMA CHEK2 HP TAOK1 PDE12 NLRP3 TP53 XIAP XKR4 AKT2 P2RX4 EIF2AK2 EYA3 CCL5 APP MAP3K9 SOX11 GAS6 USP28 CXCR4 GCLC GSK3B BCL7B	0.5469	0.7475	1.08

GO:0004707	MAP kinase activity	molecular_function	8/398	393/22360	CXCR4 F2R MAP3K2 MAP3K9 TAOK1 MAPKAPK5 EIF2AK2 MAPK12	0.5475	0.7481	1.14
GO:0051051	negative regulation of transport	biological_process	12/398	597/22360	SRCIN1 LRPAP1 GAS6 PSMD9 F2R SLC35F6 CYP51A1 AKT2 CX3CL1 NLRP3 C5AR2 APP	0.5486	0.749	1.13
GO:0009617	response to bacterium	biological_process	15/398	752/22360	CCL5 RAB29 NLRP3 IKZF3 CCR4 CASP8 SHPK MR1 CNR2 F2R SBNO2 IFNAR1 CX3CL1 HP SPN	0.5488	0.7491	1.12
GO:0050866	negative regulation of cell activation	biological_process	5/398	244/22360	GCLC SOX11 CX3CL1 CNR2 SPN	0.5489	0.749	1.15
GO:0005244	voltage-gated ion channel activity	molecular_function	6/398	294/22360	KCNJ15 GAS6 STAC2 APP ANO6 CACNB2	0.5527	0.7536	1.15
GO:0071466	cellular response to xenobiotic stimulus	biological_process	4/398	196/22360	CHEK2 ARNT2 AS3MT SULT1B1	0.5532	0.7541	1.15
GO:0014069	postsynaptic density	cellular_component	7/398	344/22360	CAMK1 P2RX4 TANC2 SRCIN1 DGKZ BAALC ADD2	0.5534	0.7542	1.14
GO:0005539	glycosaminoglycan binding	molecular_function	5/398	245/22360	PGF GNS APP NLRP3 LRPAP1	0.5548	0.7559	1.15
GO:0043413	macromolecule glycosylation	biological_process	6/398	295/22360	GALNT16 GALNT15 A4GALT B3GALT1 TMTC1 KDELC2	0.5581	0.7594	1.14
GO:0006486	protein glycosylation	biological_process	6/398	295/22360	GALNT15 GALNT16 A4GALT TMTC1 B3GALT1 KDELC2	0.5581	0.7594	1.14
GO:0005874	microtubule	cellular_component	9/398	446/22360	BCAS3 TTLL7 FHDC1 TTLL3 MYO5A WHAMM FAM161A DYNC2LI1 DNAL1	0.5582	0.7591	1.13
GO:0002790	peptide secretion	biological_process	14/398	703/22360	CX3CL1 CYP51A1 PSMD9 F2R SRCIN1 GAS6 CYB5R4 MYO5A C5AR2 SMAD2 RAB3B BTN3A2 NLRP3 CCL5	0.5585	0.7593	1.12
GO:0007010	cytoskeleton organization	biological_process	28/398	1439/22360	TRAF3IP1 CHEK2 TTLL3 TAOK1 NCOR1 GSK3B ATXN3 DNAL1 ADD2 TBCK FITM2 CORO7 FGD5 WNT4 SON WHAMM PSTPIP2 CX3CL1 FHDC1 KRT18 KLHL1 CC2D2A SPC25 BCAS3 TMOD3 DRG1 MYO1C SPTAN1	0.5587	0.7594	1.09
GO:0048660	regulation of smooth muscle cell proliferation	biological_process	4/398	197/22360	CCL5 CX3CL1 CALCRL IRAK4	0.5599	0.7594	1.14
GO:0001227	DNA-binding transcription repressor activity, RNA polymerase II-specific	molecular_function	5/398	246/22360	MNT POU5F1 MXD1 MLX MLXIPL	0.5608	0.7604	1.14
GO:0035303	regulation of dephosphorylation	biological_process	5/398	246/22360	FARP1 EIF2AK2 PPP1R37 GSK3B RCAN1	0.5608	0.7604	1.14
GO:0045930	negative regulation of mitotic cell cycle	biological_process	8/398	396/22360	CHEK2 TAOK1 PNPT1 MDM4 MSH6 TP53 PSMD9 USP44	0.5614	0.7608	1.13
GO:1902533	positive regulation of intracellular signal transduction	biological_process	22/398	1123/22360	C5AR2 APP MAPKAPK5 MAP3K9 NMNAT1 CCL5 SMCR8 CASP8 TP53 CX3CL1 EIF2AK2 P2RX4 MAP3K2 TAOK1 LTBR SOX11 GAS6 EMC10 F2R IRAK4 CXCR4 TRIM25	0.5625	0.7621	1.1
GO:0016071	mRNA metabolic process	biological_process	20/398	1018/22360	EXOSC6 NSRP1 CWC25 BRDT SAMD4B FTO APP TP53 RPRD1B PDE12 POU5F1 ADAR C9ORF78 SRSF10 PNPT1 HNRNPUL1 SON TSEN2 PSMD9 ZC3H14	0.5634	0.7632	1.1
GO:0048659	smooth muscle cell proliferation	biological_process	4/398	199/22360	CX3CL1 CCL5 CALCRL IRAK4	0.5733	0.7751	1.13
GO:0032279	asymmetric synapse	cellular_component	7/398	348/22360	TANC2 DGKZ SRCIN1 BAALC ADD2 CAMK1 P2RX4	0.5734	0.7751	1.13
GO:0006869	lipid transport	biological_process	8/398	399/22360	XKR4 ESYT3 NCOR1 ANO6 APOL1 P2RX4 ABCC3 AKT2	0.5754	0.7776	1.13
GO:0042110	T cell activation	biological_process	11/398	553/22360	SPN ICOSLG CCR6 WNT4 CASP8 RAB29 CD3G CCL5 YES1 NLRP3 PKNOX1	0.5773	0.7787	1.12
GO:1902115	regulation of organelle assembly	biological_process	5/398	249/22360	RABGAP1 DYNC2LI1 DRG1 ODF2L SMCR8	0.5787	0.7804	1.13
GO:0040013	negative regulation of locomotion	biological_process	9/398	451/22360	CX3CL1 C5AR2 SCAI PTPRM CCDC125 SRGAP2B TIE1 WNT4 TRIM25	0.5801	0.7821	1.12
GO:0016050	vesicle organization	biological_process	7/398	350/22360	F2R STX16 VPS28 ZDHHC15 RAB22A AKT2 SYP	0.5834	0.7863	1.12
GO:0000209	protein polyubiquitination	biological_process	7/398	350/22360	RNF115 TTC3 FBXL4 PSMD9 FBXL18 UBE3C XIAP	0.5834	0.7863	1.12

GO:0051650	establishment of vesicle localization	biological_process	6/398	300/22360	MYO5A SYP GSK3B MYO1C CACNB2 SYT12	0.5853	0.7885	1.12
GO:0000151	ubiquitin ligase complex	cellular_component	6/398	300/22360	FBXL18 RNF7 KLHL8 PCGF5 FBXL4 DCUN1D2	0.5853	0.7885	1.12
GO:0009653	anatomical structure morphogenesis	biological_process	56/398	2958/22360	SMAD2 TMEM106B CAMK1 GJC1 VPS33A TBCCD1 FARP1 PTPRM SRCIN1 TRAF3IP1 CRX F2R RARG FOXD2 SMARCC1 MYO5A HIF1AN PGF ITGA3 COL8A1 PKNOX1 POU5F1 CX3CL1 CC2D2A KRT18 ZMYM5 PNPT1 RILPL1 WNT4 ZFPM2 APP ARFRP1 RPL7L1 FITM2 GSK3B SOX11 CXCR4 EPN1 EMC10 PIK3R3 C2ORF49 CALCRL BCAS3 SPTAN1 TMOD3 LIG1 VSIG1 NUBPL VPS52 EYA3 PEAK1 PSMD9 MLXIPL FGD5 SBNO2 TIE1	0.589	0.7918	1.06
GO:0043687	post-translational protein modification	biological_process	8/398	402/22360	FBXL18 RNF7 GAS6 PSMD9 SUMF2 FBXL4 APP APOL1	0.5895	0.7923	1.12
GO:0001822	kidney development	biological_process	6/398	301/22360	SMAD2 ITGA3 PGF SOX11 TRAF3IP1 WNT4	0.5907	0.7937	1.12
GO:1902749	regulation of cell cycle G2/M phase transition	biological_process	5/398	251/22360	TAOK1 TP53 PSMD9 MSH6 APP	0.5907	0.7937	1.12
GO:0009743	response to carbohydrate	biological_process	5/398	251/22360	MLXIPL CALCRL GAS6 GCLC SMAD2	0.5907	0.7937	1.12
GO:0032269	negative regulation of cellular protein metabolic process	biological_process	24/398	1240/22360	USP44 GAS6 SRCIN1 TRAF3IP1 CNKSR3 GSK3B NCOR1 GCLC PRKAR2A ADAR NXN ITIH5 PAPLN SAMD4B FTO APP MLXIPL VPS28 STYX EIF2AK2 PPP1R37 SMCR8 XIAP SMARCC1	0.5924	0.7949	1.09
GO:0045859	regulation of protein kinase activity	biological_process	17/398	870/22360	MAPKAPK5 MAP3K9 CAMK1 APP SYAP1 SMCR8 ADAR CCL5 PRKAR2A TAOK1 MAP3K2 EIF2AK2 F2R CXCR4 ADCY2 GAS6 SRCIN1	0.5926	0.795	1.1
GO:0010950	positive regulation of endopeptidase activity	biological_process	4/398	202/22360	LGMN CASP8 F2R NLRP3	0.5935	0.796	1.11
GO:0071248	cellular response to metal ion	biological_process	4/398	202/22360	APP P2RX4 SYT12 LGMN	0.5935	0.796	1.11
GO:0015672	monovalent inorganic cation transport	biological_process	12/398	609/22360	COX5B ANO6 SLC33A1 ABCC9 KCNJ15 ATP6V1A SLC12A2 SLC9A4 KCNN3 P2RX4 SLC9A7 CNKSR3	0.5939	0.7961	1.11
GO:0051604	protein maturation	biological_process	6/398	302/22360	CTSS PCSK7 LGMN CASP8 XIAP DDI2	0.5962	0.798	1.12
GO:0071222	cellular response to lipopolysaccharide	biological_process	5/398	252/22360	CCL5 NLRP3 CX3CL1 SHPK SBNO2	0.5968	0.7986	1.11
GO:0045732	positive regulation of protein catabolic process	biological_process	5/398	252/22360	GCLC VPS28 GSK3B ATXN3 SUMO2	0.5968	0.7986	1.11
GO:0044255	cellular lipid metabolic process	biological_process	23/398	1189/22360	FITM2 PLPP2 ACOT8 DGKZ PDP2 A4GALT SUMF2 MGLL B3GALT1 FDPS ACOX1 ACSL6 ESYT3 GPAT4 CYP4V2 PIK3R3 MYO5A PLEKHA2 KDSR MLXIPL PIGL GK5 AKT2	0.5981	0.7999	1.09
GO:0002218	activation of innate immune response	biological_process	8/398	404/22360	OTUD4 XIAP RFTN1 CTSS CASP8 IRAK4 LGMN PSMD9	0.5989	0.8005	1.11
GO:0071478	cellular response to radiation	biological_process	4/398	203/22360	CHEK2 AKT2 TP53 USP28	0.6003	0.8022	1.11
GO:0035770	ribonucleoprotein granule	cellular_component	5/398	253/22360	APOBEC3D SAMD4B DHX30 OGFOD1 ICOSLG	0.6028	0.8053	1.11

GO:0023052	signaling	biological_process	139/398	7503/22360	SLC33A1 CYB5R4 SPTAN1 ICOSLG TRIM72 XIAP ZNF8 RFC2 SYT12 OR7D2 NLRP3 ARHGEF39 RAB40B CARD19 AKT2 EIF2AK2 ATP2B1 P2RX4 TIE1 ZNF106 MLXIPL DDX54 MAPK12 SSTR2 C5AR2 SNX27 SLC12A2 DGKZ USP28 EPN1 IRAK4 CXCR4 MGLL CNKSR3 GAS6 ITGA3 PGF HIF1AN SYP SMARCC1 RARG MSH6 RAB29 CD3G RGS17 GABRB3 KRT18 CC2D2A CX3CL1 TRABD2B IFNAR1 STAC2 PDE4C WNT4 OAS3 GJC1 ANO6 CAMK1 GPR155 CSF2RA GRK5 RAB3B LGMN TBCK ADAR PRKAR2A FARP1 F2R TENM2 TNK2 MNT TRAF3IP1 MAPKAPK5 MYO1C CACNB2 CALCRL PIK3R3 SCAI SYAP1 TP53 ERMAP ABCC9 ATP6V1A MAP3K2 EYA3 STYX MTAP FGD5 PSMD9 PCDHB9 BTNL9 NXN MAP3K9 RCAN1 APP RNF115 CCR4 PIGR ARFRP1 CCL5 BCL7B NCOR1 RABGAP1 GSK3B RGS12 GCLC EMC10 TRIM25 SOX11 CCDC125 AAK1 MYO5A CCR6 SMCR8 BTN3A2 CTSS WBP2 NMNAT1 LTBR OTUD4 RAB22A ADCY2 YES1 LRPAP1 DUOX1 MKNK1 SMAD2 CASP8 PTPRM TAOK1 RFTN1 SPN ATXN3 SEZ6 KMT2D CHEK2 SLC35F6 CNR2 MDM4	0.6033	0.8058	1.04
GO:1902495	transmembrane transporter complex	cellular_component	7/398	354/22360	ABCC9 LRRC8B CLCC1 TTYH1 GABRB3 CACNB2 ANO6	0.6036	0.806	1.11
GO:0033043	regulation of organelle organization	biological_process	27/398	1405/22360	USP44 SLC35F6 TRAF3IP1 DYNC2LI1 TAOK1 GSK3B ADD2 CASP8 CAMK1 RABGAP1 WNT4 ZNF274 ODF2L WHAMM AKT2 CX3CL1 TP53 SMCR8 WBP2 BCAS3 TMOD3 MAPKAPK5 DRG1 SPTAN1 MYO1C MYO5A CCDC36	0.6058	0.8081	1.08
GO:1905114	cell surface receptor signaling pathway involved in cell-cell signaling	biological_process	14/398	717/22360	XIAP RARG GRK5 APP NXN ITGA3 TRABD2B SEZ6 P2RX4 GSK3B GABRB3 BCL7B PSMD9 WNT4	0.6074	0.8094	1.1
GO:0016032	viral process	biological_process	19/398	982/22360	CASP8 TP53 ACOT8 MSH6 PDE12 RAB29 ADAR CCL5 APOBEC3D CXCR4 TRIM25 OAS3 FDPS GAS6 VPS28 KRT18 LTBR EIF2AK2 C19ORF66	0.6127	0.8162	1.09
GO:0016055	Wnt signaling pathway	biological_process	11/398	562/22360	APP GRK5 XIAP RARG NXN ITGA3 WNT4 PSMD9 TRABD2B BCL7B GSK3B	0.6132	0.8167	1.1
GO:0070507	regulation of microtubule cytoskeleton organization	biological_process	4/398	205/22360	DRG1 GSK3B TAOK1 TRAF3IP1	0.6138	0.8173	1.1
GO:0007565	female pregnancy	biological_process	4/398	205/22360	PGF ITGA3 EPN1 WNT4	0.6138	0.8173	1.1
GO:0045892	negative regulation of transcription, DNA-templated	biological_process	25/398	1302/22360	NCOR1 N4BP2L2 KMT2D GCLC ZNF345 TENM2 MDM4 SOX11 MNT GAS6 CIART MLX SMAD2 ZHX3 SBNO2 ZFPM2 WNT4 MLXIPL HIF1AN RARG TP53 ZNF8 RIPPLY3 MXD1 POU5F1	0.6155	0.8185	1.08
GO:0120035	regulation of plasma membrane bounded cell projection organization	biological_process	14/398	720/22360	BCAS3 ITGA3 RABGAP1 TMEM106B CAMK1 RAB29 ODF2L GSK3B SEZ6 CX3CL1 P2RX4 TENM2 DYNC2LI1 SRCIN1	0.618	0.8216	1.09
GO:0035239	tube morphogenesis	biological_process	19/398	984/22360	COL8A1 PKNOX1 PIK3R3 RARG SMAD2 BCAS3 PGF CALCRL HIF1AN GJC1 SOX11 EPN1 WNT4 EMC10 TIE1 ZFPM2 CX3CL1 CC2D2A PTPRM	0.6187	0.8223	1.08
GO:0006575	cellular modified amino acid metabolic process	biological_process	4/398	206/22360	DUOX1 VNN2 GCLC SULT1B1	0.6206	0.8246	1.09
GO:0010821	regulation of mitochondrion organization	biological_process	4/398	206/22360	SLC35F6 CASP8 GSK3B TP53	0.6206	0.8246	1.09
GO:0050679	positive regulation of epithelial cell proliferation	biological_process	4/398	206/22360	SOX11 C5AR2 EMC10 PGF	0.6206	0.8246	1.09
GO:0005764	lysosome	cellular_component	16/398	826/22360	CALCRL MYO5A TMEM106B PIGR LGMN CYB561A3 CTSS VPS33A GNS ATP6V1A P2RX4 IFNAR1 CXCR4 SLC35F6 PYGB ABCC10	0.6207	0.8241	1.09
GO:0198738	cell-cell signaling by wnt	biological_process	11/398	564/22360	GRK5 APP XIAP RARG ITGA3 NXN PSMD9 WNT4 TRABD2B BCL7B GSK3B	0.6213	0.8247	1.1
GO:0008015	blood circulation	biological_process	11/398	564/22360	ABCC9 RCAN1 CACNB2 GJC1 F2R YES1 GAS6 PTPRM P2RX4 GCLC ATP2B1	0.6213	0.8247	1.1

GO:0070085	glycosylation	biological_process	6/398	307/22360	GALNT16 GALNT15 A4GALT B3GALT1 TMTC1 KDELC2	0.6238	0.8274	1.1
GO:0003690	double-stranded DNA binding	molecular_function	20/398	1039/22360	TP53 WBP2 ZHX3 MSH6 MXD1 IKZF3 POU5F1 MLX CIART RARG SMARCC1 SMAD2 MLXIPL PCGF5 CRX GMEB1 SOX11 MNT TCF4 ARNT2	0.6245	0.8279	1.08
GO:0099568	cytoplasmic region	cellular_component	10/398	513/22360	SPTAN1 TTLL3 AKT2 BBS1 HMCN2 PRKAR2A ZC3H14 DNAL1 TRAF3IP1 DYNC2LI1	0.6254	0.8289	1.1
GO:0015077	monovalent inorganic cation transmembrane transporter activity	molecular_function	10/398	513/22360	SLC9A4 CNKSR3 KCNJ15 SLC12A2 ATP6V1A ABCC9 SLC9A7 SLC33A1 COX5B KCNN3	0.6254	0.8289	1.1
GO:0006887	exocytosis	biological_process	20/398	1040/22360	HP GSK3B SRCIN1 GAS6 PYGB MYO5A APP SYP STEAP2 TRIM72 ANO6 SPTAN1 CACNB2 CTSS CCL5 VPS33A GNS SYT12 PIGR RAB3B	0.6275	0.8313	1.08
GO:0005773	vacuole	cellular_component	18/398	934/22360	IFNAR1 P2RX4 ABCC3 ABCC10 CXCR4 PYGB SLC35F6 MYO5A TMEM106B CALCRL RAB29 VPS33A CTSS ATP6V1A GNS CYB561A3 LGMN PIGR	0.628	0.8317	1.08
GO:0071346	cellular response to interferon-gamma	biological_process	6/398	308/22360	CX3CL1 OAS3 PDE12 CCL5 MYO1C TRIM25	0.6293	0.8332	1.09
GO:0072331	signal transduction by p53 class mediator	biological_process	6/398	308/22360	TP53 USP28 MDM4 RFC2 MAPKAPK5 CHEK2	0.6293	0.8332	1.09
GO:0015711	organic anion transport	biological_process	10/398	514/22360	NCOR1 CYB5R4 SLC33A1 ANO6 P2RX4 SLC26A2 AKT2 ABCC3 XKR4 SLC37A2	0.6297	0.8333	1.09
GO:0051249	regulation of lymphocyte activation	biological_process	10/398	515/22360	SPN EXOSC6 ICOSLG CCL5 YES1 GAS6 NLRP3 SOX11 IKZF3 MSH6	0.634	0.8375	1.09
GO:0098793	presynapse	cellular_component	10/398	515/22360	RAB3B SYT12 AAK1 CACNB2 STX16 APP P2RX4 ATP2B1 SYP SYAP1	0.634	0.8375	1.09
GO:0007219	Notch signaling pathway	biological_process	4/398	208/22360	EPN1 HIF1AN AAK1 APP	0.6343	0.8375	1.08
GO:0000785	chromatin	cellular_component	12/398	620/22360	MXD1 MSH6 WBP2 SMCR8 TP53 SMAD2 SMARCC1 RARG BCAS3 TTI2 TCF4 NCOR1	0.6362	0.8398	1.09
GO:0050867	positive regulation of cell activation	biological_process	8/398	412/22360	EXOSC6 ICOSLG APP MSH6 CCL5 YES1 NLRP3 GAS6	0.637	0.8406	1.09
GO:0008047	enzyme activator activity	molecular_function	11/398	568/22360	RGS17 ELMOD1 TAOK1 RGS12 GAS6 BCAS3 RABGAP1 APP TBCK TOR1AIP2 CCL5	0.6375	0.8411	1.09
GO:0071356	cellular response to tumor necrosis factor	biological_process	7/398	361/22360	PSMD9 CASP8 CCL5 GAS6 LTBR KRT18 CX3CL1	0.6394	0.8431	1.09
GO:0002460	adaptive immune response based on somatic recombination of immune receptors built from immunoglobulin superfamily domains	biological_process	7/398	361/22360	MSH6 NLRP3 BTN3A2 CCR6 EXOSC6 RFTN1 SPN	0.6394	0.8431	1.09
GO:0023051	regulation of signaling	biological_process	77/398	4134/22360	LGMN TBCK CASP8 RAB3B GRK5 SMAD2 TRAF3IP1 MNT CNR2 MDM4 F2R SLC35F6 CHEK2 FARP1 SEZ6 KMT2D TAOK1 RAB29 WBP2 NMNAT1 SMCR8 MYO5A SYP AAK1 ITGA3 PGF HIF1AN LRPAP1 WNT4 OTUD4 CX3CL1 TRABD2B LTBR RGS17 CCL5 RNF115 C5AR2 APP MAP3K9 RCAN1 NXN CNKSR3 GAS6 SOX11 CCDC125 TRIM25 EPN1 IRAK4 MGLL CXCR4 EMC10 GCLC RGS12 RABGAP1 NCOR1 GSK3B NLRP3 RFC2 ABCC9 SYT12 TP53 SCAI XIAP TRIM72 MAPKAPK5 MYO1C CACNB2 FGD5 PSMD9 EIF2AK2 ATP2B1 P2RX4 STYX ARHGEF39 MAP3K2 EYA3 CARD19	0.641	0.8448	1.05
GO:0045766	positive regulation of angiogenesis	biological_process	4/398	209/22360	TIE1 EMC10 PGF CX3CL1	0.6411	0.8447	1.08

GO:0006996	organelle organization	biological_process	78/398	4189/22360	ZDHHC15 BCAS3 MYO1C SPTAN1 DRG1 MAPKAPK5 TMOD3 CCDC36 TP53 ATP6V1A RFC2 EYA3 VPS28 FHDC1 PSTPIP2 AKT2 NUBPL FGD5 FAM161A UBXN2A OGFOD1 CORO7 NDUFA10 FITM2 DNAL1 ACOT8 GSK3B NCOR1 TTLL3 GCLC PCGF5 TMEM170A C9ORF84 DYNC2LI1 SPC25 SYP SMARCC1 MYO5A BRDT SMCR8 WBP2 RAB29 MSH6 CC2D2A KLHL1 KRT18 PNPT1 ODF2L ZNF274 WHAMM RAB22A CX3CL1 WNT4 BBS1 SON RILPL1 STX16 RABGAP1 CAMK1 TMEM106B ADD2 TBCK DHX30 TOR1AIP2 NDUFAF7 CASP8 VPS33A COG1 ATXN3 TAOK1 ACOX1 CHEK2 KMT2D NAP1L6 SLC35F6 F2R USP44 TRAF3IP1	0.6415	0.845	1.05
GO:0048568	embryonic organ development	biological_process	9/398	465/22360	TRAF3IP1 SOX11 EPN1 ZFPM2 CASP8 RARG SMAD2 CC2D2A VPS52	0.6427	0.8464	1.09
GO:0071219	cellular response to molecule of bacterial origin	biological_process	5/398	260/22360	SHPK SBNO2 CCL5 NLRP3 CX3CL1	0.6454	0.8489	1.08
GO:0008017	microtubule binding	molecular_function	5/398	260/22360	DRG1 FAM161A FHDC1 TRAF3IP1 WHAMM	0.6454	0.8489	1.08
GO:0071453	cellular response to oxygen levels	biological_process	5/398	260/22360	HIF1AN PSMD9 TP53 ATP6V1A MDM4	0.6454	0.8489	1.08
GO:0046474	glycerophospholipid biosynthetic process	biological_process	5/398	260/22360	PIGL DGKZ PIK3R3 PLEKHA2 GPAT4	0.6454	0.8489	1.08
GO:0072593	reactive oxygen species metabolic process	biological_process	6/398	311/22360	TP53 CYB5R4 HP DUOX1 P2RX4 HBE1	0.646	0.8488	1.08
GO:0052547	regulation of peptidase activity	biological_process	10/398	519/22360	MAPK12 XIAP APP ITIH5 PAPLN GAS6 NLRP3 CASP8 F2R LGMN	0.6511	0.8544	1.08
GO:0097060	synaptic membrane	cellular_component	9/398	467/22360	SYAP1 SYP ATP2B1 GABRB3 ITGA3 SRCIN1 TENM2 F2R BAALC	0.6517	0.855	1.08
GO:0008283	cell population proliferation	biological_process	44/398	2342/22360	RARG PGF COL8A1 IKZF3 TAF8 CX3CL1 WNT4 ZFPM2 GRK5 FTO SMAD2 TBCK LGMN ARNT2 KMT2D N4BP2L2 SPN PTPRM MNT MDM4 F2R TNK2 SLC35F6 XIAP CALCRL ICOSLG MXD1 RIPPLY3 TP53 EIF2AK2 AKT2 MLXIPL OGFOD1 C5AR2 APP SSTR2 CCL5 RPRD1B ACSL6 GAS6 SOX11 IRAK4 USP28 EMC10	0.6523	0.8556	1.06
GO:0031344	regulation of cell projection organization	biological_process	14/398	730/22360	RAB29 BCAS3 ITGA3 TMEM106B CAMK1 RABGAP1 TENM2 DYNC2LI1 SRCIN1 ODF2L GSK3B SEZ6 CX3CL1 P2RX4	0.6539	0.8561	1.08
GO:0008016	regulation of heart contraction	biological_process	5/398	262/22360	CACNB2 GJC1 ABCC9 ATP2B1 P2RX4	0.6576	0.8608	1.07
GO:0030336	negative regulation of cell migration	biological_process	7/398	365/22360	SRGAP2B WNT4 TIE1 SCAI C5AR2 CX3CL1 PTPRM	0.66	0.8637	1.08
GO:0009612	response to mechanical stimulus	biological_process	4/398	212/22360	GCLC MAP3K2 LTBR CASP8	0.6616	0.8656	1.06
GO:0042593	glucose homeostasis	biological_process	5/398	263/22360	GAS6 GCLC CYB5R4 MLXIPL RABGAP1	0.6637	0.8679	1.07
GO:0071560	cellular response to transforming growth factor beta stimulus	biological_process	5/398	263/22360	YES1 SMAD2 SOX11 WNT4 ITGA3	0.6637	0.8679	1.07
GO:0048871	multicellular organismal homeostasis	biological_process	10/398	522/22360	FTO P2RX4 ADCY2 VSIG1 PRKAR2A F2R MLXIPL BBS1 CXCR4 PIGR	0.664	0.8678	1.08
GO:0070661	leukocyte proliferation	biological_process	7/398	366/22360	WNT4 CCL5 IKZF3 SOX11 SPN ICOSLG CX3CL1	0.6652	0.8692	1.07
GO:0048638	regulation of developmental growth	biological_process	7/398	366/22360	LGMN ZFPM2 GPAT4 RABGAP1 GSK3B APP FTO	0.6652	0.8692	1.07
GO:0008236	serine-type peptidase activity	molecular_function	6/398	315/22360	PCSK7 HP PAPLN ITIH5 DPP9 APP	0.6683	0.8728	1.07
GO:0050658	RNA transport	biological_process	4/398	213/22360	PNPT1 MYO1C RFTN1 MCM3AP	0.6685	0.8728	1.06
GO:0015081	sodium ion transmembrane	molecular_function	4/398	213/22360	SLC12A2 CNKSR3 SLC9A4 SLC9A7	0.6685	0.8728	1.06

	transporter activity							
GO:0042127	regulation of cell population proliferation	biological_process	36/398	1916/22360	KMT2D ARNT2 SPN N4BP2L2 PTPRM GAS6 MDM4 SOX11 MNT IRAK4 SLC35F6 TNK2 EMC10 F2R APP C5AR2 GRK5 SMAD2 SSTR2 FTO CCL5 RPRD1B LGMN EIF2AK2 CX3CL1 AKT2 MLXIPL ZFPM2 XIAP RARG CALCRL PGF ICOSLG IKZF3 TP53 RIPPLY3	0.6687	0.8726	1.06
GO:0033500	carbohydrate homeostasis	biological_process	5/398	264/22360	GCLC GAS6 CYB5R4 RABGAP1 MLXIPL	0.6699	0.8735	1.06
GO:0050707	regulation of cytokine secretion	biological_process	5/398	264/22360	CX3CL1 GAS6 NLRP3 C5AR2 F2R	0.6699	0.8735	1.06
GO:0051271	negative regulation of cellular component movement	biological_process	8/398	419/22360	CX3CL1 C5AR2 SCAI PTPRM CCDC125 SRGAP2B TIE1 WNT4	0.6707	0.8741	1.07
GO:0072001	renal system development	biological_process	6/398	316/22360	WNT4 SOX11 TRAF3IP1 PGF ITGA3 SMAD2	0.674	0.8782	1.07
GO:0099003	vesicle-mediated transport in synapse	biological_process	4/398	214/22360	SYT12 SYP CACNB2 GSK3B	0.6754	0.8798	1.05
GO:0070820	tertiary granule	cellular_component	4/398	214/22360	CTSS HP ANO6 SPTAN1	0.6754	0.8798	1.05
GO:0051056	regulation of small GTPase mediated signal transduction	biological_process	7/398	368/22360	ARHGEF39 ITGA3 FARP1 SCAI F2R FGD5 CCDC125	0.6755	0.8795	1.07
GO:0007281	germ cell development	biological_process	5/398	265/22360	CCR6 CCDC36 KMT2D WNT4 ICA1L	0.676	0.8799	1.06
GO:0031667	response to nutrient levels	biological_process	10/398	525/22360	GAS6 WNT4 TP53 TRIM25 SSTR2 GCLC ATP2B1 P2RX4 EIF2AK2 BCAS3	0.6769	0.8809	1.07
GO:0009986	cell surface	cellular_component	19/398	1003/22360	F2R CXCR4 WNT4 LRPAP1 SPN CX3CL1 BTN3A2 CCR4 ERMAP CD3G ANO6 BTNL9 ICOSLG ITGA3 DUOX1 CSF2RA MR1 APP CCR6	0.6771	0.8809	1.06
GO:0006401	RNA catabolic process	biological_process	9/398	473/22360	PDE12 DIS3L ZC3H14 PSMD9 SAMD4B SLFN13 FTO PNPT1 EXOSC6	0.679	0.8827	1.07
GO:0010948	negative regulation of cell cycle process	biological_process	8/398	421/22360	TAOK1 CHEK2 USP44 TP53 PSMD9 MLXIPL MSH6 MDM4	0.6804	0.8843	1.07
GO:0090068	positive regulation of cell cycle process	biological_process	6/398	318/22360	APP CHEK2 TMOD3 MDM4 WNT4 TP53	0.6852	0.8903	1.06
GO:0050778	positive regulation of immune response	biological_process	21/398	1113/22360	C5AR2 XIAP EXOSC6 BTNL9 ICOSLG MYO1C MSH6 CD3G RAB29 CCL5 CTSS NLRP3 CASP8 BTN3A2 ERMAP LGMN OTUD4 RFTN1 YES1 PSMD9 IRAK4	0.6855	0.8905	1.06
GO:1902532	negative regulation of intracellular signal transduction	biological_process	11/398	580/22360	ITGA3 RCAN1 MAPKAPK5 SCAI CASP8 NLRP3 NCOR1 GSK3B CARD19 CNKSR3 CCDC125	0.6867	0.8918	1.07
GO:0031406	carboxylic acid binding	molecular_function	4/398	216/22360	OGFOD1 ACOX1 HIF1AN GCLC	0.6891	0.8947	1.04
GO:0017171	serine hydrolase activity	molecular_function	6/398	319/22360	PCSK7 ITIH5 PAPLN HP APP DPP9	0.6908	0.8967	1.06
GO:0006915	apoptotic process	biological_process	41/398	2198/22360	GAS6 MNT MDM4 CXCR4 F2R USP28 SLC35F6 CHEK2 GCLC GSDMA ARNT2 BCL7B TAOK1 GSK3B CCL5 NOA1 LGMN CASP8 APP GRK5 MAP3K9 ANO6 WNT4 XKR4 SON P2RX4 EIF2AK2 AKT2 CX3CL1 LTBR GABRB3 KRT18 EYA3 CD3G NLRP3 NMNAT1 MSH6 IKZF3 TP53 XIAP RARG	0.6913	0.8971	1.05
GO:0005102	signaling receptor binding	molecular_function	36/398	1927/22360	BTNL9 DDX54 SMAD2 APP CASP8 ACOT8 CCL5 ACOX1 NCOR1 ARNT2 TNK2 F2R CRX IRAK4 GAS6 MYO1C MED17 ICOSLG HIF1AN AAK1 BCAS3 ITGA3 PGF RARG BTN3A2 ERMAP TP53 CD3G WBP2 CX3CL1 P2RX4 UBXN2A BBS1 WNT4 YES1 LRPAP1	0.6933	0.8995	1.05
GO:0033002	muscle cell proliferation	biological_process	5/398	268/22360	CCL5 CX3CL1 CALCRL IRAK4 ZFPM2	0.6945	0.9008	1.05
GO:0016051	carbohydrate biosynthetic process	biological_process	4/398	217/22360	PGM2L1 AKT2 B3GALT1 GSK3B	0.696	0.9026	1.04
GO:0006367	transcription initiation from RNA polymerase II promoter	biological_process	4/398	217/22360	RARG TCF4 TP53 MED17	0.696	0.9026	1.04
GO:0043112	receptor metabolic process	biological_process	4/398	217/22360	LGMN CALCRL LRPAP1 RAB29	0.696	0.9026	1.04

GO:0030278	regulation of ossification	biological_process	4/398	217/22360	WNT4 ANO6 ZHX3 SOX11	0.696	0.9026	1.04
GO:0007154	cell communication	biological_process	138/398	7533/22360	USP28 EPN1 IRAK4 MGLL CXCR4 CNKSR3 GAS6 SNX27 SLC12A2 DGKZ DDX54 MAPK12 SSTR2 C5AR2 TIE1 ZNF106 MLXIPL ARHGEF39 RAB40B CARD19 AKT2 EIF2AK2 ATP2B1 P2RX4 ZNF8 RFC2 SYT12 OR7D2 NLRP3 SLC33A1 CYB5R4 ICOSLG SPTAN1 TRIM72 XIAP F2R TENM2 TNK2 MNT TRAF3IP1 FARP1 RAB3B LGMN TBCK ADAR PRKAR2A GJC1 ANO6 CAMK1 GPR155 CSF2RA GRK5 STAC2 PDE4C WNT4 OAS3 RGS17 GABRB3 KRT18 CC2D2A CX3CL1 TRABD2B IFNAR1 MSH6 RAB29 CD3G ITGA3 PGF HIF1AN SYP SMARCC1 RARG EMC10 TRIM25 SOX11 CCDC125 BCL7B RABGAP1 NCOR1 GSK3B RGS12 GCLC RNF115 CCR4 PIGR ARFRP1 CCL5 BTNL9 NXN MAP3K9 RCAN1 APP FGD5 PSMD9 PCDHB9 MAP3K2 EYA3 STYX MTAP TP53 ERMAP ATP6V1A MAPKAPK5 MYO1C CACNB2 CALCRL PIK3R3 SCAI SYAP1 SLC35F6 CNR2 MDM4 PTPRM TAOK1 RFTN1 SPN ATXN3 SEZ6 KMT2D CHEK2 CASP8 DUOX1 MKNK1 SMAD2 ADCY2 YES1 LRPAP1 LTBR OTUD4 RAB22A SMCR8 BTN3A2 CTSS WBP2 NMNAT1 AAK1 MYO5A CCR6	0.6972	0.9032	1.03
GO:0009987	cellular process	biological_process	337/398	18568/22360	ZNF789 RFTN1 ARNT2 GSDMA ZNF345 CS ZNF573 USP44 MDM4 DCTD DUOX1 TBCCD1 ZNF274 LTBR WHAMM ZNF445 AAK1 CCR6 BTN3A2 PPP1R37 B3GALT1 QPRT PDP2 CIART RCAN1 ZNF441 CORO7 PLEKHB2 CCR4 ARFRP1 EYA3 UBE3C STYX PSTPIP2 SBNO2 TMTC1 C1ORF61 CACNB2 MED17 SYAP1 SCAI FARP1 CRX GJC1 ANO6 MLX CSF2RA A4GALT RAB3B DDX51 SNORA13 DIS3L VPS33A ZHX3 RGS17 ZMYM5 TRABD2B IFNAR1 PLEKHA2 STAC2 WNT4 IKZF3 CD3G TTLL3 USP28 H6PD GAS6 CNKSR3 MTFMT NT5DC3 COQ6 ASPA TCF4 ATP2B1 FBXL4 MIR654 NPAT GK5 ZDHHC15 BCAS3 TRIM72 ABCB5 ZNF814 RIPPLY3 OR7D2 PTPRM ATXN3 SLC35F6 NSRP1 SMAD2 SAMD4B COX5B NDUFAF7 CASP8 TOR1AIP2 DHX30 ZNF677 COG1 RAB22A OTUD4 ZNF669 ZFPM2 PCSK7 YES1 SLC26A2 RRP1 APOBEC3D MYO5A SMCR8 NMNAT1 GSK3B RABGAP1 NCOR1 RGS12 ZNF483 HERC4 SOX11 RNF7 ZNF772 PIGR CCL5 MAP3K2 ZNF264 MTAP OGFOD1 PCDHB9 MAPKAPK5 TMOD3 CALCRL TONSL TP53 LIG1 ACOX1 DCUN1D2 HP ZNF583 TTC3 TENM2 LRRC8B SRCIN1 ZNF766 PRKAR2A ADAR KLHL1 SON GMEB1 RILPL1 ITGA3 AS3MT SPC25 SMARCC1 FOXD2 MSH6 PKNOX1 POU5F1 COL8A1 RAB29 SUMF2 CXCR4 C9ORF84 C5AR2 FITM2 DGKZ UEVLD GALNT15 MED22 AKT2 PYGB TSEN2 ZC3H14 CYB5R4 EXOSC6 CWC25 CCDC36 XIAP SHPK NLRP3 SPN TTLL7 N4BP2L2 ZNF430 SEZ6 CNR2 PRR11 IBA57 STEAP2 CRYZL1 NOA1 ADCY2 INTS6 ISG20L2 GNS TRIM25 DYNC2LI1 FDPS CCDC125 NXN MAP3K9 RNF115 RPRD1B DNAL1 NAGK PEAK1 VPS28 SLFN13 PGM2L1 XKR4 PSMD9 FAM161A FGD5 ZNF7 MYO1C DRG1 CYP4V2 PIK3R3 ERMAP METTL21A F2R TRAF3IP1 STX16 PAPLN GPR155 TMEM106B CAMK1 FTO TTYH1 TBCK PNPT1 CC2D2A KRT18 TAF8 ZNF80 PDE4C KDSR GSDMB PIGL GALNT16 BRDT ZNF286B RARG MARVELD2 ESYT3 SRGAP2B TMEM170A MGLL IRAK4 ZKSCAN1 ITIH5 DDX54 SNX27 PLPP2 RPL7L1 SLC9A4 SRSF10 VPS52 CARD19 RAB40B HNRNPUL1 EIF2AK2 UBXN2A ZNF106 TIE1 ICOSLG PPM1K TAOK1 KMT2D CHEK2 MKNK1 SULT1B1 ADD2 SUMO2 TRMT10B ODF2L BBS1 ZNF70 LRPAP1 SCYL3 ICA1L KLHL8 WBP2 CTSS FBXL18 BCL7B ACSL6 GCLC EMC10 KDELC2 BTNL9 NDUFA10 APP ACOT8 ZNF570 LRTOMT APOL1 GPAT4 C2ORF49 ATP6V1A PDE12 VSIG1 ZBTB37 HBE1 TNK2 NAP1L6 MNT MMS22L GRK5 LGMN GABRB3 CX3CL1 OAS3 SLC39A13 HIF1AN PGF SYP C9ORF78 SLC9A7 CYP51A1 FCF1 PCGF5 EPN1 ZNF800 GNPTG SSTR2 MAPK12 SLC12A2 ARHGEF39 FHDC1 NUBPL VNN2 P2RX4 MLXIPL	0.6976	0.9035	1.02

					SPTAN1 SLC33A1 ZNF8 SYT12 MXD1 RFC2			
GO:0030234	enzyme regulator activity	molecular_function	22/398	1171/22360	TOR1AIP2 TBCK CCL5 PRKAR2A PAPLN ITIH5 RCAN1 RABGAP1 APP TNK2 GAS6 TAOK1 RGS12 SMCR8 PPP1R37 BCAS3 PIK3R3 XIAP RGS17 ELMOD1 STYX EIF2AK2	0.6978	0.9035	1.06
GO:0072358	cardiovascular system development	biological_process	16/398	849/22360	COL8A1 PKNOX1 PIK3R3 HIF1AN CALCRL PGF BCAS3 NXN GJC1 WNT4 EPN1 ZFPM2 TIE1 EMC10 CX3CL1 PTPRM	0.6982	0.9038	1.06
GO:0009749	response to glucose	biological_process	4/398	218/22360	GCLC GAS6 SMAD2 MLXIPL	0.7029	0.9097	1.03
GO:0007423	sensory organ development	biological_process	11/398	584/22360	SOX11 TRAF3IP1 LRTOMT PTPRM GABRB3 CC2D2A ATP2B1 PKNOX1 COL8A1 ABCB5 RARG	0.7032	0.9098	1.06
GO:0071559	response to transforming growth factor beta	biological_process	5/398	270/22360	ITGA3 SMAD2 WNT4 YES1 SOX11	0.7068	0.9143	1.04
GO:0030424	axon	cellular_component	12/398	639/22360	GSK3B P2RX4 TENM2 ZC3H14 SRCIN1 PCDH9 ITGA3 AAK1 APP MYO5A SYP SYAP1	0.7109	0.9194	1.06
GO:0030099	myeloid cell differentiation	biological_process	9/398	480/22360	VPS33A PKNOX1 SBNO2 CASP8 RARG APP KMT2D LTBR TMOD3	0.7111	0.9194	1.05
GO:0030072	peptide hormone secretion	biological_process	5/398	271/22360	CYB5R4 PSMD9 SMAD2 MYO5A CCL5	0.713	0.9216	1.04
GO:0006694	steroid biosynthetic process	biological_process	4/398	220/22360	WNT4 ACOT8 CYP51A1 FDPS	0.7167	0.9262	1.02
GO:0001568	blood vessel development	biological_process	15/398	801/22360	PKNOX1 COL8A1 GJC1 BCAS3 PGF CALCRL HIF1AN PIK3R3 EMC10 TIE1 ZFPM2 EPN1 WNT4 PTPRM CX3CL1	0.7175	0.927	1.05
GO:0032880	regulation of protein localization	biological_process	21/398	1124/22360	MYO1C ITGA3 BCAS3 AAK1 CAMK1 C5AR2 CASP8 TP53 NLRP3 CCL5 RAB29 VPS28 GSK3B CX3CL1 CYP51A1 AKT2 STAC2 F2R PSMD9 GAS6 SRCIN1	0.7182	0.9276	1.05
GO:0010638	positive regulation of organelle organization	biological_process	13/398	695/22360	WNT4 GSK3B AKT2 CX3CL1 WHAMM SMCR8 CASP8 TP53 WBP2 MAPKAPK5 DRG1 MYO1C BCAS3	0.7213	0.9314	1.05
GO:0098978	glutamatergic synapse	cellular_component	7/398	377/22360	TANC2 DGKZ YES1 GSK3B CAMK1 MYO5A ATP2B1	0.7224	0.9326	1.04
GO:0002695	negative regulation of leukocyte activation	biological_process	4/398	221/22360	SPN CX3CL1 CNR2 SOX11	0.7235	0.9338	1.02
GO:0007187	G protein-coupled receptor signaling pathway, coupled to cyclic nucleotide second messenger	biological_process	5/398	273/22360	CALCRL SSTR2 GRK5 CNR2 ADCY2	0.7253	0.9359	1.03
GO:0006006	glucose metabolic process	biological_process	4/398	222/22360	H6PD GSK3B PGM2L1 AKT2	0.7304	0.9422	1.01
GO:0051091	positive regulation of DNA-binding transcription factor activity	biological_process	6/398	327/22360	NLRP3 IRAK4 TRIM25 CX3CL1 EIF2AK2 APP	0.7359	0.9491	1.03
GO:0051321	meiotic cell cycle	biological_process	5/398	275/22360	BRDT CCDC36 C9ORF84 MSH6 WNT4	0.7377	0.9512	1.02
GO:0010720	positive regulation of cell development	biological_process	11/398	593/22360	APP CAMK1 TMEM106B ITGA3 TCF4 P2RX4 CX3CL1 ASPA SOX11 CRX CXCR4	0.7407	0.9548	1.04
GO:0030155	regulation of cell adhesion	biological_process	15/398	808/22360	CCL5 COL8A1 NLRP3 ITGA3 BCAS3 ICOSLG CXCR4 WNT4 PLEKHA2 YES1 ATXN3 GSK3B SPN PEAK1 CX3CL1	0.7424	0.9567	1.04
GO:0022613	ribonucleoprotein complex biogenesis	biological_process	10/398	541/22360	SRSF10 EXOSC6 RRP1 OGFOD1 FCF1 DDX51 DHX30 RPL7L1 ISG20L2 ADAR	0.7466	0.9619	1.04
GO:0043405	regulation of MAP kinase activity	biological_process	7/398	382/22360	EIF2AK2 MAPKAPK5 MAP3K9 MAP3K2 TAOK1 F2R CXCR4	0.7486	0.9642	1.03
GO:0031227	intrinsic component of endoplasmic reticulum membrane	cellular_component	5/398	277/22360	ESYT3 SEC62 FITM2 EMC10 SLC37A2	0.7501	0.9659	1.01

GO:0010951	negative regulation of endopeptidase activity	biological_process	5/398	277/22360	XIAP GAS6 APP ITIH5 PAPLN	0.7501	0.9659	1.01
GO:0035295	tube development	biological_process	22/398	1189/22360	SMAD2 GJC1 SOX11 TRAF3IP1 EMC10 EPN1 PTPRM PKNOX1 COL8A1 PIK3R3 RARG PGF ITGA3 BCAS3 HIF1AN CALCRL ZFPM2 TIE1 WNT4 CX3CL1 VPS52 CC2D2A	0.7505	0.966	1.04
GO:0051094	positive regulation of developmental process	biological_process	29/398	1570/22360	MSH6 NLRP3 SYAP1 EXOSC6 HIF1AN ITGA3 PGF TIE1 ZFPM2 WNT4 CX3CL1 ASPA TCF4 P2RX4 ZHX3 CASP8 MAPK12 SMAD2 CAMK1 TMEM106B APP ANO6 SOX11 GAS6 EMC10 CRX CXCR4 N4BP2L2 RABGAP1	0.7508	0.9661	1.04
GO:0034622	cellular protein-containing complex assembly	biological_process	24/398	1298/22360	SRSF10 TCF4 EIF2AK2 WHAMM NUBPL PSTPIP2 PSMD9 OGFOD1 DRG1 SPTAN1 MYO1C TMOD3 TP53 NLRP3 NAP1L6 TMEM170A CORO7 NDUFA10 SMAD2 ADD2 DHX30 NDUFAF7 DNAL1 ADAR	0.7515	0.9668	1.04
GO:0045666	positive regulation of neuron differentiation	biological_process	7/398	383/22360	CRX SOX11 ITGA3 TMEM106B CAMK1 CX3CL1 TCF4	0.7539	0.9696	1.03
GO:0042326	negative regulation of phosphorylation	biological_process	9/398	490/22360	NCOR1 MLXIPL SMCR8 SRCIN1 PRKAR2A ADAR TRAF3IP1 CNKSR3 PDE12	0.7573	0.9738	1.03
GO:0052548	regulation of endopeptidase activity	biological_process	9/398	490/22360	ITIH5 PAPLN APP XIAP LGMN CASP8 F2R NLRP3 GAS6	0.7573	0.9738	1.03
GO:0009101	glycoprotein biosynthetic process	biological_process	7/398	384/22360	GALNT15 GALNT16 A4GALT TMTC1 B3GALT1 KDELC2 ACOT8	0.7591	0.9756	1.02
GO:0030135	coated vesicle	cellular_component	7/398	385/22360	AAK1 SYNRG STEAP2 APP TNK2 CD3G VPS33A	0.7644	0.9821	1.02
GO:0045597	positive regulation of cell differentiation	biological_process	20/398	1086/22360	CRX WNT4 CXCR4 GAS6 SOX11 P2RX4 TCF4 ASPA CX3CL1 CASP8 ZHX3 NLRP3 ITGA3 HIF1AN APP SYAP1 CAMK1 TMEM106B MAPK12 SMAD2	0.7674	0.9858	1.03
GO:0050878	regulation of body fluid levels	biological_process	10/398	546/22360	ANO6 GPAT4 P2RX4 HBE1 ZFPM2 F2R ADCY2 GAS6 DGKZ PRKAR2A	0.7686	0.9871	1.03
GO:0009266	response to temperature stimulus	biological_process	5/398	280/22360	GCLC ATP2B1 GSK3B ATXN3 CASP8	0.7687	0.9869	1
GO:0045787	positive regulation of cell cycle	biological_process	8/398	440/22360	MDM4 TP53 MLXIPL WNT4 LGMN CHEK2 APP TMOD3	0.7733	0.9926	1.02
GO:0007049	cell cycle	biological_process	38/398	2072/22360	NPAT WNT4 PSMD9 MLXIPL SON KRT18 PNPT1 WHAMM AKT2 TP53 LIG1 FBXL18 RFC2 MSH6 TMOD3 DRG1 SPC25 BRDT CCDC36 USP28 USP44 GAS6 MNT SOX11 MDM4 C9ORF84 NCOR1 TAOK1 CHEK2 LGMN RPRD1B PRR11 APP GRK5 CAMK1 TTYH1 RABGAP1 MAPK12	0.7738	0.993	1.03
GO:0005126	cytokine receptor binding	molecular_function	6/398	334/22360	PGF SMAD2 CX3CL1 IRAK4 CASP8 CCL5	0.7756	0.9951	1.01
GO:0007017	microtubule-based process	biological_process	15/398	818/22360	TTLL3 CHEK2 ATXN3 GSK3B CC2D2A TAOK1 NCOR1 DYNC2LI1 TRAF3IP1 SON SPC25 APP DRG1 BCAS3 DNAL1	0.7783	0.9983	1.03
GO:0010038	response to metal ion	biological_process	7/398	388/22360	P2RX4 APP GCLC SLC39A13 SYT12 CASP8 LGMN	0.7801	1	1.01
GO:0005739	mitochondrion	cellular_component	32/398	1747/22360	COQ6 NOA1 DHX30 MTFMT NDUFAF7 CASP8 NDUFA10 IBA57 COX5B MAPK12 LETMD1 PDP2 CS SLC35F6 ACSL6 GSK3B ATXN3 SLC25A44 PPM1K LIG1 RAB29 MXD1 PDE12 POU5F1 TP53 COX6B2 GK5 SLC25A26 FBXL4 NUBPL CARD19 PNPT1	0.7833	1	1.03
GO:0034612	response to tumor necrosis factor	biological_process	7/398	389/22360	CASP8 PSMD9 GAS6 CCL5 LTBR KRT18 CX3CL1	0.7854	1	1.01
GO:0010008	endosome membrane	cellular_component	12/398	658/22360	IRAK4 CYB561A3 PLEKHB2 SNX27 VPS33A VPS28 VPS52 SLC9A7 RAB22A TMEM106B SNX21 STEAP2	0.7869	1	1.02
GO:0030111	regulation of Wnt signaling pathway	biological_process	7/398	390/22360	PSMD9 ITGA3 GSK3B NXN APP XIAP TRABD2B	0.7906	1	1.01

GO:0048856	anatomical structure development	biological_process	118/398	6506/22360	SRGAP2B RAB29 CD3G COL8A1 IKZF3 MSH6 PKNOX1 POU5F1 SMARCC1 FOXD2 RARG MARVELD2 HIF1AN PGF ITGA3 SLC39A13 RILPL1 WNT4 TAF8 CX3CL1 CC2D2A ZMYM5 KRT18 GABRB3 KLHL1 PNPT1 VPS33A FTO TMEM106B CAMK1 GJC1 ANO6 SRCIN1 TRAF3IP1 MNT CRX F2R TENM2 FARP1 NLRP3 MXD1 RIPPLY3 CCDC36 TRIM72 ABCB5 BCAS3 CYB5R4 SPTAN1 EXOSC6 MLXIPL TIE1 TCF4 EIF2AK2 P2RX4 ATP2B1 NUBPL AKT2 ASPA VPS52 SLC9A4 RPL7L1 FITM2 SSTR2 MAPK12 GAS6 EPN1 CXCR4 TANC2 ICA1L MYO5A CCR6 ZFPM2 LTBR TBCCD1 ADD2 DHX30 SULT1B1 CASP8 SMAD2 DUOX1 SEZ6 ARNT2 ZNF430 KMT2D SPN ATXN3 N4BP2L2 TTLL7 PTPRM LIG1 VSIG1 TP53 C2ORF49 PIK3R3 CALCRL GPAT4 DRG1 SLC35E2B TMOD3 LRTOMT ZNF7 PCDHB9 PSMD9 FGD5 SBNO2 XKR4 EYA3 PEAK1 ARFRP1 CCR4 APP RCAN1 NXN SOX11 DYNC2LI1 EMC10 ACSL6 GSK3B	0.7937	1	1.02
GO:0001505	regulation of neurotransmitter levels	biological_process	7/398	392/22360	P2RX4 SYP GSK3B CACNB2 LRTOMT SYT12 RAB3B	0.8012	1	1
GO:0030133	transport vesicle	cellular_component	9/398	500/22360	RAB3B SYT12 GALNT15 STX16 APP TMEM187 SYP STEAP2 SYNRG	0.8037	1	1.01
GO:0051128	regulation of cellular component organization	biological_process	49/398	2694/22360	SEZ6 TAOK1 SRCIN1 TRAF3IP1 TNK2 SLC35F6 TENM2 USP44 RABGAP1 TMEM106B CAMK1 ANO6 ADD2 TBCCD1 CASP8 WHAMM TRABD2B CX3CL1 ZMYM5 ZNF274 ODF2L LRPAP1 WNT4 MYO5A ITGA3 AAK1 NMNAT1 WBP2 RAB29 SMCR8 GSK3B DYNC2LI1 APP FITM2 P2RX4 EIF2AK2 AKT2 PEAK1 VPS28 FGD5 OGFOD1 CCDC36 BCAS3 SPTAN1 MYO1C DRG1 MAPKAPK5 TMOD3 TP53	0.8085	1	1.02
GO:0030054	cell junction	cellular_component	26/398	1429/22360	SYT12 SYAP1 SYP MARVELD2 TMOD3 ITGA3 YES1 BAALC P2RX4 PEAK1 RGS17 GABRB3 KRT18 PRKAR2A APP PCDH9 STX16 GJC1 SRCIN1 HMCN2 TNK2 TENM2 CXCR4 RGS12 FARP1 PTPRM	0.8119	1	1.02
GO:0005789	endoplasmic reticulum membrane	cellular_component	23/398	1265/22360	ESYT3 CYP4V2 CYB5R4 SLC33A1 GPAT4 PIGL SLC37A2 KDSR PNPT1 CARD19 FITM2 TOR1AIP2 MR1 TTYH1 GJC1 TMEM170A EMC10 MGLL LRRC8B CYP51A1 ACSL6 SEC62 ATXN3	0.8133	1	1.02
GO:0016020	membrane	cellular_component	194/398	10751/22360	SLC37A2 ADCY2 TMEM116 KCNN3 WHAMM LTBR BTN3A2 CCR6 AAK1 MS4A7 CNR2 HMCN2 GSDMA SEZ6 TMEM187 RFTN1 SPN KCNJ15 NOA1 DUOX1 STEAP2 PRR11 BAALC XKR4 FGD5 TMTC1 PSTPIP2 VPS28 ERMAP CYP4V2 PIK3R3 SCAI SYAP1 SLC35E2B DRG1 MYO1C CACNB2 MED17 C1ORF43 ABCC10 B3GALT1 SEC62 ARFRP1 CCR4 PLEKHB2 CORO7 PIGL PLEKHA2 STAC2 GSDMB WNT4 KDSR TRABD2B IFNAR1 PNPT1 RGS17 IKZF3 CD3G ESYT3 MARVELD2 RARG GALNT16 C1ORF210 F2R FARP1 DIS3L RRP12 VPS33A RAB3B DDX51 TTYH1 SLC30A6 TMEM106B GPR155 CSF2RA A4GALT GJC1 ANO6 STX16 MLX ZNF106 TIE1 EIF2AK2 ATP2B1 RAB40B CARD19 VPS52 OR7D2 ABCB5 TRIM72 ICOSLG ZDHHC15 CNKSR3 TMEM170A IRAK4 MGLL SLC25A44 TMEM233 SLC9A4 COQ6 SNX27 PLPP2 PCDH9 DDX54 SLC35E1 PCSK7 YES1 LRPAP1 BBS1 RAB22A COX6B2 MCM3AP SLC26A2 MYO5A SLC35F6 TMEM260 PTPRM ATXN3 COG1 CASP8 TOR1AIP2 ADD2 LETMD1 COX5B AP4M1 PCDHB9 LRTOMT TMEM266 TMEM105 VSIG1 ABCC9 ATP6V1A APOL1 GPAT4 CALCRL EMC10 RGS12 ACSL6 NCOR1 RABGAP1 GSK3B PIGR APP NDUFA10 BTNL9 OAS3 RILPL1 CX3CL1 GABRB3 RAB29 SYP SLC39A13 AP4E1 ITGA3 PGF SRCIN1 TENM2 TNK2 LRRC8B ABCC3 ACOX1 ADAR PRKAR2A CYB561A3 SYNRG MR1 GRK5 PYGB SLC25A26 AKT2 NUBPL P2RX4 VNN2 GALNT15 ARHGEF39 SYT12 NLRP3 SLC33A1 CYB5R4 SPTAN1 LRRC57 CLCC1 EPN1 CXCR4 CEP112 CYP51A1 SLC9A7 SLC12A2 DGKZ FITM2 SSTR2 C5AR2 SNX21 GNPTG	0.8141	1	1.01
GO:1901990	regulation of mitotic cell cycle	biological_process	9/398	503/22360	APP CHEK2 TMOD3 TAOK1 MSH6 MDM4 USP44 PSMD9 TP53	0.8176	1	1.01

	phase transition							
GO:0002682	regulation of immune system process	biological_process	39/398	2153/22360	YES1 PSMD9 P2RX4 EIF2AK2 CX3CL1 OTUD4 NLRP3 CTSS CD3G RAB29 MSH6 IKZF3 ERMAP BTN3A2 SHPK RARG XIAP CCR6 PGF ICOSLG MYO1C EXOSC6 GAS6 TRAF3IP1 CNR2 SOX11 IRAK4 KMT2D SPN RFTN1 N4BP2L2 CCL5 LGMN PIGR CASP8 APP C5AR2 BTNL9 ANO6	0.8319	1	1.02
GO:0008233	peptidase activity	molecular_function	21/398	1163/22360	PCSK7 DPP9 TRABD2B OTUD4 DDI2 NLRP3 CTSS XIAP GAS6 DESI1 F2R USP44 USP28 ATXN3 HP LGMN CASP8 APP MAPK12 ITIH5 PAPLN	0.8363	1	1.01
GO:0072359	circulatory system development	biological_process	22/398	1220/22360	TRAF3IP1 SOX11 EPN1 EMC10 PTPRM SMAD2 GJC1 NXN WNT4 ZFPM2 TIE1 CX3CL1 CC2D2A COL8A1 PKNOX1 RIPPLY3 PIK3R3 ITGA3 PGF BCAS3 HIF1AN CALCRL	0.8425	1	1.01
GO:0001944	vasculature development	biological_process	15/398	837/22360	GJC1 HIF1AN CALCRL PGF BCAS3 PIK3R3 PKNOX1 COL8A1 PTPRM CX3CL1 ZFPM2 EMC10 TIE1 WNT4 EPN1	0.8468	1	1.01
GO:0097435	supramolecular fiber organization	biological_process	13/398	728/22360	WNT4 WHAMM PSTPIP2 CX3CL1 FHDC1 TAOK1 ADD2 APP CORO7 TMOD3 DRG1 SPTAN1 MYO1C	0.8487	1	1
GO:0002376	immune system process	biological_process	69/398	3827/22360	OAS3 YES1 WNT4 CX3CL1 OTUD4 C19ORF66 IFNAR1 LTBR IKZF3 MSH6 PKNOX1 GNS RAB29 CD3G CTSS BTN3A2 MCM3AP RARG APOBEC3D CCR6 PGF ITGA3 CNR2 TRAF3IP1 KMT2D SPN HP RFTN1 N4BP2L2 VPS33A ADAR RAB3B CASP8 ADD2 LGMN MR1 ANO6 SBNO2 PYGB PSMD9 SLFN13 EIF2AK2 P2RX4 ABCC9 PDE12 NLRP3 LIG1 SHPK ERMAP XIAP SPTAN1 ICOSLG MYO1C TMOD3 EXOSC6 APOL1 SOX11 GAS6 CXCR4 IRAK4 TRIM25 SLC12A2 CCL5 SNX27 PIGR CCR4 C5AR2 APP BTNL9	0.8542	1	1.01
GO:0005694	chromosome	cellular_component	21/398	1169/22360	TTI2 TCF4 SMCR8 TP53 MSH6 MXD1 RFC2 WBP2 BCAS3 CCDC36 TONSL RARG SPC25 SMARCC1 PCGF5 C9ORF84 NCOR1 RGS12 CHEK2 SMAD2 MMS22L	0.8546	1	1.01
GO:0046903	secretion	biological_process	34/398	1890/22360	CYP51A1 ABCC3 HP GSK3B SOX11 GAS6 SRCIN1 F2R SMAD2 APP C5AR2 STEAP2 ANO6 VPS33A CCL5 SLC9A4 RAB3B PIGR CX3CL1 P2RX4 PYGB PSMD9 TRIM72 MYO5A SYP CACNB2 SPTAN1 CYB5R4 GPAT4 GNS SYT12 CTSS NLRP3 BTN3A2	0.863	1	1.01
GO:0042175	nuclear outer membrane-endoplasmic reticulum membrane network	cellular_component	23/398	1288/22360	LRRC8B MGLL EMC10 TMEM170A ACSL6 CYP51A1 ATXN3 SEC62 TOR1AIP2 FITM2 TTYH1 MR1 GJC1 SLC37A2 PIGL KDSR CARD19 PNPT1 ESYT3 CYP4V2 GPAT4 CYB5R4 SLC33A1	0.8805	1	1
GO:0065003	protein-containing complex assembly	biological_process	39/398	2175/22360	NDUFA10 SMAD2 CORO7 ANO6 ADAR CCL5 DNAL1 ADD2 DHX30 NDUFAF7 CASP8 HBE1 KMT2D GSK3B TRAF3IP1 MDM4 NAP1L6 QPRT TMEM170A TRIM72 MYO1C SPTAN1 DRG1 TMOD3 NLRP3 CD3G TP53 TCF4 P2RX4 WHAMM EIF2AK2 CX3CL1 TRABD2B PSTPIP2 NUBPL SRSF10 PNPT1 PSMD9 OGFOD1	0.8812	1	1.01
GO:0070062	extracellular exosome	cellular_component	43/398	2403/22360	APP GNPTG NAGK SLC12A2 PIGR GAS6 LRRC57 QPRT CXCR4 SYAP1 MYO1C ICOSLG SPTAN1 ATP6V1A ATP2B1 P2RX4 MTAP UEVLD VPS28 AP4M1 PYGB GPR155 ANO6 PRKAR2A LGMN RAB3B SPN N4BP2L2 TAOK1 RFTN1 HP SLC35F6 CS MYO5A SLC26A2 ITGA3 RAB29 GNS RAB22A KRT18 YES1 SLC37A2 WNT4	0.8939	1	1.01

GO:0071944	cell periphery	cellular_component	118/398	6603/22360	PCDH9 SSTR2 C5AR2 SNX27 PLPP2 SLC12A2 SLC9A4 DGKZ SLC9A7 CYP51A1 CEP112 CXCR4 MGLL IRAK4 EPN1 CNKSR3 SPTAN1 ICOSLG SLC33A1 BCAS3 TRIM72 ABCB5 SYT12 OR7D2 ARHGEF39 RAB40B NUBPL AKT2 P2RX4 ATP2B1 VNN2 TIE1 GJC1 ANO6 TTYH1 CSF2RA MR1 GRK5 RAB3B DIS3L PRKAR2A ACOX1 FARP1 ABCC3 TENM2 F2R TNK2 LRRC8B C1ORF210 SRCIN1 ITGA3 MARVELD2 SYP ESYT3 IKZF3 RAB29 CD3G RGS17 KRT18 GABRB3 TRABD2B CX3CL1 IFNAR1 PLEKHA2 STAC2 WNT4 GSDMB OAS3 RILPL1 BTNL9 APP PIGR CCR4 RABGAP1 GSK3B RGS12 ACSL6 ABCC10 CACNB2 MYO1C CALCRL SYAP1 ERMAP ABCC9 VSIG1 ATP6V1A VPS28 TMEM266 PSTPIP2 BAALC XKR4 FGD5 PCDHB9 LRTOMT DUOX1 STEAP2 CASP8 ADD2 KCNJ15 PTPRM RFTN1 ATXN3 SPN GSDMA SEZ6 HMCN2 CNR2 AAK1 SLC26A2 CCR6 BTN3A2 LTBR RAB22A KCNN3 BBS1 ADCY2 LRPAP1 YES1	0.9183	1	1

TABLE S4 KEGG enrichment analysis of the circRNA parental genes

Pathway ID	Description	GeneRatio	BgRatio	*P* value	Q value	Gene list	Enrich_factor
hsa03430	Mismatch repair	3/146	24/8877	0.001566	0.06421	LIG1 RFC2 MSH6	7.6
hsa00130	Ubiquinone and other terpenoid-quinone biosynthesis	1/146	11/8877	0.02957	0.1819	COQ6	5.53
hsa01524	Platinum drug resistance	6/146	76/8877	0.000732	0.04502	CASP8 XIAP TP53 AKT2 MSH6 PIK3R3	4.8
hsa04216	Ferroptosis	3/146	40/8877	0.009765	0.1264	TP53 ACSL6 GCLC	4.56
hsa00061	Fatty acid biosynthesis	1/146	14/8877	0.04634	0.228	ACSL6	4.34
hsa00601	Glycosphingolipid biosynthesis - lacto and neolacto series	2/146	29/8877	0.02622	0.1792	A4GALT B3GALT1	4.19
hsa04923	Regulation of lipolysis in adipocytes	4/146	58/8877	0.006449	0.1133	AKT2 PIK3R3 ADCY2 MGLL	4.19
hsa05210	Colorectal cancer	6/146	88/8877	0.001712	0.06016	TP53 AKT2 SMAD2 GSK3B MSH6 PIK3R3	4.15
hsa00603	Glycosphingolipid biosynthesis - globo and isoglobo series	1/146	15/8877	0.05262	0.2354	A4GALT	4.05
hsa05142	Chagas disease (American trypanosomiasis)	8/146	121/8877	0.0005556	0.04556	CCL5 CASP8 AKT2 MAPK12 SMAD2 PIK3R3 CD3G IRAK4	4.02
hsa05213	Endometrial cancer	4/146	61/8877	0.007962	0.1152	TP53 AKT2 GSK3B PIK3R3	3.99
hsa00760	Nicotinate and nicotinamide metabolism	2/146	31/8877	0.03125	0.183	QPRT NMNAT1	3.92
hsa05162	Measles	10/146	155/8877	0.0001918	0.04718	TP53 OAS3 AKT2 EIF2AK2 GSK3B ADAR PIK3R3 CD3G IRAK4 IFNAR1	3.92
hsa00512	Mucin type O-glycan biosynthesis	2/146	31/8877	0.03125	0.183	GALNT16 GALNT15	3.92
hsa02010	ABC transporters	4/146	63/8877	0.009101	0.1244	ABCC3 ABCB5 ABCC10 ABCC9	3.86
hsa04919	Thyroid hormone signaling pathway	8/146	131/8877	0.0009748	0.04796	TP53 AKT2 WNT4 MED17 GSK3B NCOR1 PIK3R3 RCAN1	3.71
hsa04215	Apoptosis - multiple species	2/146	34/8877	0.03974	0.208	CASP8 XIAP	3.58
hsa04722	Neurotrophin signaling pathway	7/146	124/8877	0.002864	0.07045	ZNF274 TP53 AKT2 MAPK12 GSK3B PIK3R3 IRAK4	3.43
hsa00561	Glycerolipid metabolism	4/146	71/8877	0.01482	0.1519	DGKZ PLPP2 GPAT4 MGLL	3.43
hsa00120	Primary bile acid biosynthesis	1/146	18/8877	0.07333	0.2653	ACOT8	3.38
hsa01210	2-Oxocarboxylic acid metabolism	1/146	18/8877	0.07333	0.2653	CS	3.38
hsa04620	Toll-like receptor signaling pathway	7/146	128/8877	0.003478	0.0713	CCL5 CASP8 AKT2 MAPK12 PIK3R3 IRAK4 IFNAR1	3.33
hsa03030	DNA replication	2/146	37/8877	0.04937	0.2292	LIG1 RFC2	3.29
hsa04115	p53 signaling pathway	4/146	74/8877	0.01749	0.1594	CASP8 TP53 CHEK2 MDM4	3.29
hsa04917	Prolactin signaling pathway	4/146	74/8877	0.01749	0.1594	AKT2 MAPK12 GSK3B PIK3R3	3.29
hsa04550	Signaling pathways regulating pluripotency of stem cells	8/146	152/8877	0.002701	0.07383	POU5F1 PCGF5 AKT2 WNT4 MAPK12 SMAD2 GSK3B PIK3R3	3.2
hsa00604	Glycosphingolipid biosynthesis - ganglio series	1/146	19/8877	0.08082	0.2651	SLC33A1	3.2
hsa00531	Glycosaminoglycan degradation	1/146	19/8877	0.08082	0.2651	GNS	3.2
hsa04971	Gastric acid secretion	4/146	77/8877	0.02048	0.1679	SLC9A4 KCNJ15 SSTR2 ADCY2	3.16
hsa05212	Pancreatic cancer	4/146	78/8877	0.02155	0.171	TP53 AKT2 SMAD2 PIK3R3	3.12
hsa05214	Glioma	4/146	79/8877	0.02266	0.1742	TP53 AKT2 PIK3R3 CAMK1	3.08
hsa04932	Non-alcoholic fatty liver disease (NAFLD)	9/146	178/8877	0.002244	0.069	COX6B2 NDUFA10 CASP8 COX5B AKT2 MLXIPL MLX GSK3B PIK3R3	3.07
hsa00770	Pantothenate and CoA biosynthesis	1/146	20/8877	0.08859	0.2724	VNN2	3.04
hsa00500	Starch and sucrose metabolism	2/146	40/8877	0.06013	0.2348	PYGB PGM2L1	3.04
hsa05222	Small cell lung cancer	5/146	101/8877	0.01562	0.1537	XIAP TP53 AKT2 ITGA3 PIK3R3	3.01
hsa04370	VEGF signaling pathway	3/146	61/8877	0.04009	0.2055	AKT2 MAPK12 PIK3R3	2.99
hsa05164	Influenza A	14/146	286/8877	0.0003514	0.04322	FDPS CCL5 OAS3 NLRP3 AKT2 MAPK12 EIF2AK2 HNRNPUL1 GSK3B TRIM25 ADAR	2.98
hsa04931	Insulin resistance	6/146	123/8877	0.01071	0.1255	PYGB AKT2 MLXIPL MLX GSK3B PIK3R3	2.97
hsa01521	EGFR tyrosine kinase inhibitor resistance	4/146	82/8877	0.0262	0.1841	GAS6 AKT2 GSK3B PIK3R3	2.97
hsa00100	Steroid biosynthesis	1/146	21/8877	0.09661	0.2612	CYP51A1	2.9
hsa05160	Hepatitis C	8/146	170/8877	0.005638	0.1067	CASP8 TP53 OAS3 AKT2 EIF2AK2 GSK3B PIK3R3 IFNAR1	2.86
hsa01522	Endocrine resistance	6/146	128/8877	0.01316	0.1472	TP53 AKT2 MAPK12 NCOR1 PIK3R3 ADCY2	2.85
hsa04668	TNF signaling pathway	6/146	130/8877	0.01425	0.1524	CCL5 CASP8 AKT2 MAPK12 CX3CL1 PIK3R3	2.81
hsa04062	Chemokine signaling pathway	10/146	218/8877	0.003058	0.06839	CCL5 GRK5 CCR4 CXCR4 AKT2 GSK3B CX3CL1 CCR6 PIK3R3 ADCY2	2.79
hsa04210	Apoptosis	7/146	153/8877	0.01001	0.1231	CASP8 XIAP TP53 SPTAN1 AKT2 PIK3R3 CTSS	2.78
hsa05217	Basal cell carcinoma	3/146	66/8877	0.05148	0.2345	TP53 WNT4 GSK3B	2.76
hsa00670	One carbon pool by folate	1/146	22/8877	0.1049	0.2716	MTFMT	2.76
hsa00071	Fatty acid degradation	2/146	44/8877	0.07624	0.2605	ACSL6 ACOX1	2.76
hsa00900	Terpenoid backbone biosynthesis	1/146	22/8877	0.1049	0.2716	FDPS	2.76
hsa05161	Hepatitis B	8/146	178/8877	0.007562	0.1163	CASP8 TP53 AKT2 MAPK12 SMAD2 PIK3R3 IRAK4 IFNAR1	2.73
hsa05230	Central carbon metabolism in cancer	3/146	69/8877	0.05915	0.2425	TP53 AKT2 PIK3R3	2.64
hsa04660	T cell receptor signaling pathway	5/146	115/8877	0.02796	0.1859	AKT2 MAPK12 GSK3B PIK3R3 CD3G	2.64
hsa03060	Protein export	1/146	23/8877	0.1134	0.2847	SEC62	2.64
hsa04625	C-type lectin receptor signaling pathway	5/146	116/8877	0.02904	0.1832	CASP8 NLRP3 AKT2 MAPK12 PIK3R3	2.62
hsa04142	Lysosome	6/146	139/8877	0.02	0.1697	GNPTG AP4E1 GNS AP4M1 CTSS LGMN	2.62
hsa00340	Histidine metabolism	1/146	24/8877	0.1222	0.3006	ASPA	2.53

hsa05211	Renal cell carcinoma	3/146	72/8877	0.06746	0.2477	AKT2 PIK3R3 ARNT2	2.53
hsa00270	Cysteine and methionine metabolism	2/146	48/8877	0.0943	0.2636	MTAP GCLC	2.53
hsa05167	Kaposi sarcoma-associated herpesvirus infection	10/146	241/8877	0.006465	0.106	CASP8 TP53 CCR4 AKT2 MAPK12 EIF2AK2 GSK3B PIK3R3 RCAN1 IFNAR1	2.52
hsa04973	Carbohydrate digestion and absorption	2/146	49/8877	0.09911	0.265	AKT2 PIK3R3	2.48
hsa05223	Non-small cell lung cancer	3/146	74/8877	0.07336	0.2578	TP53 AKT2 PIK3R3	2.46
hsa00520	Amino sugar and nucleotide sugar metabolism	2/146	50/8877	0.104	0.2722	CYB5R4 NAGK	2.43
hsa05032	Morphine addiction	4/146	101/8877	0.05742	0.2435	GRK5 GABRB3 PDE4C ADCY2	2.41
hsa05215	Prostate cancer	4/146	101/8877	0.05742	0.2435	TP53 AKT2 GSK3B PIK3R3	2.41
hsa05218	Melanoma	3/146	76/8877	0.07954	0.2644	TP53 AKT2 PIK3R3	2.4
hsa04910	Insulin signaling pathway	6/146	153/8877	0.03204	0.1791	PRKAR2A PYGB AKT2 MKNK1 GSK3B PIK3R3	2.38
hsa04066	HIF-1 signaling pathway	4/146	102/8877	0.05951	0.24	LTBR AKT2 MKNK1 PIK3R3	2.38
hsa05225	Hepatocellular carcinoma	7/146	179/8877	0.02399	0.1788	TP53 AKT2 WNT4 SMARCC1 SMAD2 GSK3B PIK3R3	2.38
hsa04914	Progesterone-mediated oocyte maturation	4/146	102/8877	0.05951	0.24	AKT2 MAPK12 PIK3R3 ADCY2	2.38
hsa04662	B cell receptor signaling pathway	3/146	78/8877	0.08601	0.2713	AKT2 GSK3B PIK3R3	2.34
hsa00563	Glycosylphosphatidylinositol (GPI)-anchor biosynthesis	1/146	26/8877	0.1403	0.3196	PIGL	2.34
hsa00592	alpha-Linolenic acid metabolism	1/146	26/8877	0.1403	0.3196	ACOX1	2.34
hsa05120	Epithelial cell signaling in Helicobacter pylori infection	3/146	78/8877	0.08601	0.2713	CCL5 ATP6V1A MAPK12	2.34
hsa04211	Longevity regulating pathway	4/146	105/8877	0.06607	0.2463	TP53 AKT2 PIK3R3 ADCY2	2.32
hsa04664	Fc epsilon RI signaling pathway	3/146	79/8877	0.08935	0.2714	AKT2 MAPK12 PIK3R3	2.31
hsa05220	Chronic myeloid leukemia	3/146	79/8877	0.08935	0.2714	TP53 AKT2 PIK3R3	2.31
hsa04622	RIG-I-like receptor signaling pathway	3/146	79/8877	0.08935	0.2714	CASP8 MAPK12 TRIM25	2.31
hsa04213	Longevity regulating pathway - multiple species	3/146	79/8877	0.08935	0.2714	AKT2 PIK3R3 ADCY2	2.31
hsa04072	Phospholipase D signaling pathway	6/146	159/8877	0.0385	0.2059	DGKZ PLPP2 AKT2 F2R PIK3R3 ADCY2	2.29
hsa04520	Adherens junction	3/146	80/8877	0.09276	0.2623	YES1 SMAD2 PTPRM	2.28
hsa05226	Gastric cancer	6/146	162/8877	0.04204	0.2111	TP53 AKT2 WNT4 SMAD2 GSK3B PIK3R3	2.25
hsa04611	Platelet activation	5/146	136/8877	0.05708	0.2463	AKT2 MAPK12 F2R PIK3R3 ADCY2	2.24
hsa05231	Choline metabolism in cancer	4/146	109/8877	0.07549	0.2616	DGKZ PLPP2 AKT2 PIK3R3	2.23
hsa04260	Cardiac muscle contraction	3/146	82/8877	0.0998	0.264	COX6B2 CACNB2 COX5B	2.22
hsa04978	Mineral absorption	2/146	55/8877	0.1304	0.3145	ATP2B1 STEAP2	2.21
hsa04024	cAMP signaling pathway	8/146	222/8877	0.02868	0.1857	ATP2B1 AKT2 F2R SSTR2 PDE4C PIK3R3 ADCY2 ACOX1	2.19
hsa04926	Relaxin signaling pathway	5/146	139/8877	0.06241	0.2362	AKT2 MAPK12 SMAD2 PIK3R3 ADCY2	2.19
hsa01040	Biosynthesis of unsaturated fatty acids	1/146	28/8877	0.1593	0.3212	ACOX1	2.17
hsa04621	NOD-like receptor signaling pathway	8/146	224/8877	0.03019	0.1811	CCL5 CASP8 XIAP OAS3 NLRP3 MAPK12 IRAK4 IFNAR1	2.17
hsa04714	Thermogenesis	9/146	253/8877	0.02421	0.1752	COX6B2 NDUFA10 COX5B MAPK12 NDUFAF7 SMARCC1 ACSL6 ADCY2 MGLL	2.16
hsa00600	Sphingolipid metabolism	2/146	57/8877	0.1417	0.314	KDSR PLPP2	2.13
hsa04933	AGE-RAGE signaling pathway in diabetic complications	4/146	115/8877	0.09108	0.2636	AKT2 MAPK12 SMAD2 PIK3R3	2.11
hsa03420	Nucleotide excision repair	2/146	58/8877	0.1475	0.3211	LIG1 RFC2	2.1
hsa04217	Necroptosis	6/146	176/8877	0.06158	0.2367	CASP8 XIAP PYGB NLRP3 EIF2AK2 IFNAR1	2.07
hsa04012	ErbB signaling pathway	3/146	88/8877	0.1226	0.2986	AKT2 GSK3B PIK3R3	2.07
hsa05110	Vibrio cholerae infection	2/146	59/8877	0.1535	0.32	ATP6V1A SLC12A2	2.06
hsa00630	Glyoxylate and dicarboxylate metabolism	1/146	30/8877	0.179	0.344	CS	2.03
hsa05418	Fluid shear stress and atherosclerosis	5/146	150/8877	0.08469	0.2706	TP53 AKT2 MAPK12 PIK3R3 SUMO2	2.03
hsa01212	Fatty acid metabolism	2/146	61/8877	0.1656	0.3312	ACSL6 ACOX1	1.99
hsa04261	Adrenergic signaling in cardiomyocytes	5/146	155/8877	0.09627	0.2631	ATP2B1 CACNB2 AKT2 MAPK12 ADCY2	1.96
hsa00030	Pentose phosphate pathway	1/146	31/8877	0.1891	0.3498	H6PD	1.96
hsa00020	Citrate cycle (TCA cycle)	1/146	31/8877	0.1891	0.3498	CS	1.96
hsa04146	Peroxisome	3/146	94/8877	0.1479	0.3192	ACOT8 ACSL6 ACOX1	1.94
hsa05163	Human cytomegalovirus infection	10/146	313/8877	0.03836	0.2097	CCL5 CASP8 TP53 CXCR4 AKT2 MAPK12 GSK3B CX3CL1 PIK3R3 ADCY2	1.94
hsa05014	Amyotrophic lateral sclerosis (ALS)	2/146	63/8877	0.1781	0.345	TP53 MAPK12	1.93
hsa05170	Human immunodeficiency virus 1 infection	9/146	283/8877	0.04747	0.229	CASP8 CXCR4 AKT2 MAPK12 RNF7 PIK3R3 CD3G IRAK4 APOBEC3D	1.93
hsa04666	Fc gamma R-mediated phagocytosis	3/146	95/8877	0.1524	0.3204	PLPP2 AKT2 PIK3R3	1.92
hsa04912	GnRH signaling pathway	3/146	96/8877	0.1569	0.3216	MAP3K2 MAPK12 ADCY2	1.9
hsa05133	Pertussis	3/146	96/8877	0.1569	0.3216	NLRP3 MAPK12 IRAK4	1.9
hsa04110	Cell cycle	4/146	129/8877	0.1343	0.3146	TP53 SMAD2 GSK3B CHEK2	1.89
hsa04064	NF-kappa B signaling pathway	4/146	129/8877	0.1343	0.3146	XIAP LTBR TRIM25 IRAK4	1.89
hsa04020	Calcium signaling pathway	6/146	194/8877	0.09449	0.2612	ATP2B1 CXCR4 F2R CAMK1 ADCY2 P2RX4	1.88
hsa05224	Breast cancer	5/146	163/8877	0.1167	0.29	TP53 AKT2 WNT4 GSK3B PIK3R3	1.87
hsa05200	Pathways in cancer	17/146	557/8877	0.01708	0.1616	CASP8 XIAP CSF2RA PGF TP53 CXCR4 AKT2 WNT4 ITGA3 SMAD2 F2R GSK3B MSH6	1.86
hsa04672	Intestinal immune network for IgA production	4/146	131/8877	0.1413	0.316	PIGR ICOSLG LTBR CXCR4	1.86
hsa03410	Base excision repair	1/146	33/8877	0.2097	0.3738	LIG1	1.84
hsa04071	Sphingolipid signaling pathway	4/146	133/8877	0.1484	0.3174	TP53 AKT2 MAPK12 PIK3R3	1.83

hsa04010	MAPK signaling pathway	10/146	332/8877	0.05518	0.2424	MAP3K2 CACNB2 PGF TP53 TAOK1 AKT2 MAPK12 MAPKAPK5 MKNK1 IRAK4	1.83
hsa05010	Alzheimer disease	6/146	199/8877	0.1053	0.267	COX6B2 NDUFA10 CASP8 COX5B APP GSK3B	1.83
hsa04723	Retrograde endocannabinoid signaling	5/146	168/8877	0.1307	0.3122	NDUFA10 MAPK12 GABRB3 ADCY2 MGLL	1.81
hsa05414	Dilated cardiomyopathy (DCM)	3/146	101/8877	0.1804	0.344	CACNB2 ITGA3 ADCY2	1.81
hsa04130	SNARE interactions in vesicular transport	1/146	34/8877	0.2203	0.3844	STX16	1.79
hsa04750	Inflammatory mediator regulation of TRP channels	3/146	102/8877	0.1853	0.348	MAPK12 PIK3R3 ADCY2	1.79
hsa03018	RNA degradation	3/146	102/8877	0.1853	0.348	PNPT1 EXOSC6 DIS3L	1.79
hsa05221	Acute myeloid leukemia	2/146	68/8877	0.2111	0.3709	AKT2 PIK3R3	1.79
hsa04970	Salivary secretion	3/146	103/8877	0.1903	0.3468	ATP2B1 SLC12A2 ADCY2	1.77
hsa04966	Collecting duct acid secretion	1/146	35/8877	0.231	0.3974	ATP6V1A	1.74
hsa04120	Ubiquitin mediated proteolysis	4/146	140/8877	0.1749	0.3442	UBE3C XIAP RNF7 HERC4	1.74
hsa04972	Pancreatic secretion	3/146	105/8877	0.2004	0.3625	ATP2B1 SLC12A2 ADCY2	1.74
hsa00250	Alanine, aspartate and glutamate metabolism	1/146	35/8877	0.231	0.3974	ASPA	1.74
hsa04068	FoxO signaling pathway	4/146	140/8877	0.1749	0.3442	AKT2 MAPK12 SMAD2 PIK3R3	1.74
hsa04150	mTOR signaling pathway	5/146	176/8877	0.155	0.3204	ATP6V1A AKT2 WNT4 GSK3B PIK3R3	1.73
hsa04015	Rap1 signaling pathway	6/146	211/8877	0.1342	0.3174	PGF AKT2 MAPK12 F2R PIK3R3 ADCY2	1.73
hsa05169	Epstein-Barr virus infection	10/146	352/8877	0.07794	0.2626	CASP8 TP53 OAS3 AKT2 MAPK12 EIF2AK2 PIK3R3 CD3G IRAK4 IFNAR1	1.73
hsa04916	Melanogenesis	3/146	106/8877	0.2056	0.3692	WNT4 GSK3B ADCY2	1.72
hsa04510	Focal adhesion	6/146	213/8877	0.1394	0.3205	XIAP PGF AKT2 ITGA3 GSK3B PIK3R3	1.71
hsa04728	Dopaminergic synapse	4/146	142/8877	0.1829	0.3461	AKT2 MAPK12 LRTOMT GSK3B	1.71
hsa05205	Proteoglycans in cancer	6/146	214/8877	0.1421	0.3121	TP53 AKT2 WNT4 MAPK12 SMAD2 PIK3R3	1.7
hsa04922	Glucagon signaling pathway	3/146	107/8877	0.2108	0.3731	PYGB AKT2 ADCY2	1.7
hsa05166	Human T-cell leukemia virus 1 infection	10/146	362/8877	0.09146	0.2616	FDPS XIAP TP53 LTBR AKT2 SMAD2 CHEK2 PIK3R3 ADCY2 CD3G	1.68
hsa05016	Huntington disease	6/146	217/8877	0.1502	0.3185	COX6B2 NDUFA10 CASP8 TP53 COX5B DNAL1	1.68
hsa04925	Aldosterone synthesis and secretion	3/146	109/8877	0.2214	0.3836	ATP2B1 CAMK1 ADCY2	1.67
hsa04623	Cytosolic DNA-sensing pathway	2/146	74/8877	0.2536	0.4187	CCL5 ADAR	1.64
hsa04960	Aldosterone-regulated sodium reabsorption	1/146	37/8877	0.2527	0.4229	PIK3R3	1.64
hsa05412	Arrhythmogenic right ventricular cardiomyopathy (ARVC)	2/146	74/8877	0.2536	0.4187	CACNB2 ITGA3	1.64
hsa04657	IL-17 signaling pathway	3/146	111/8877	0.2322	0.3939	CASP8 MAPK12 GSK3B	1.64
hsa00350	Tyrosine metabolism	1/146	38/8877	0.2638	0.4269	LRTOMT	1.6
hsa04976	Bile secretion	2/146	77/8877	0.2758	0.4377	ABCC3 ADCY2	1.58
hsa04670	Leukocyte transendothelial migration	3/146	117/8877	0.266	0.4277	CXCR4 MAPK12 PIK3R3	1.56
hsa00564	Glycerophospholipid metabolism	3/146	117/8877	0.266	0.4277	DGKZ PLPP2 GPAT4	1.56
hsa00190	Oxidative phosphorylation	4/146	158/8877	0.2533	0.421	COX6B2 NDUFA10 COX5B ATP6V1A	1.54
hsa04921	Oxytocin signaling pathway	4/146	159/8877	0.258	0.4203	CACNB2 CAMK1 ADCY2 RCAN1	1.53
hsa03320	PPAR signaling pathway	2/146	80/8877	0.2987	0.4593	ACSL6 ACOX1	1.52
hsa01523	Antifolate resistance	1/146	40/8877	0.2862	0.4513	ABCC3	1.52
hsa05020	Prion diseases	1/146	40/8877	0.2862	0.4513	CCL5	1.52
hsa04725	Cholinergic synapse	3/146	121/8877	0.2895	0.4507	AKT2 PIK3R3 ADCY2	1.51
hsa05165	Human papillomavirus infection	10/146	408/8877	0.1735	0.3442	CASP8 TP53 ATP6V1A AKT2 WNT4 ITGA3 EIF2AK2 GSK3B PIK3R3 IFNAR1	1.49
hsa04630	JAK-STAT signaling pathway	4/146	167/8877	0.2975	0.4603	CSF2RA AKT2 PIK3R3 IFNAR1	1.46
hsa01100	Metabolic pathways	33/146	1376/8877	0.04859	0.2299	FDPS SLC33A1 ASPA COX6B2 NDUFA10 A4GALT DGKZ PYGB CS KDSR PLPP2 GALNT16	1.46
hsa05033	Nicotine addiction	1/146	42/8877	0.309	0.4721	GABRB3	1.45
hsa04060	Cytokine-cytokine receptor interaction	8/146	337/8877	0.2331	0.3928	CCL5 CSF2RA CCR4 LTBR CXCR4 CX3CL1 CCR6 IFNAR1	1.44
hsa04915	Estrogen signaling pathway	4/146	172/8877	0.3233	0.4791	KRT18 AKT2 PIK3R3 ADCY2	1.41
hsa05219	Bladder cancer	1/146	43/8877	0.3205	0.4808	TP53	1.41
hsa05216	Thyroid cancer	1/146	43/8877	0.3205	0.4808	TP53	1.41
hsa04920	Adipocytokine signaling pathway	2/146	87/8877	0.3542	0.5156	AKT2 ACSL6	1.4
hsa05145	Toxoplasmosis	5/146	217/8877	0.3146	0.4777	CASP8 XIAP AKT2 MAPK12 IRAK4	1.4
hsa04934	Cushing syndrome	4/146	176/8877	0.3446	0.5046	WNT4 KMT2D GSK3B ADCY2	1.38
hsa05143	African trypanosomiasis	1/146	44/8877	0.3321	0.4892	APOL1	1.38
hsa05203	Viral carcinogenesis	6/146	265/8877	0.3156	0.4763	CASP8 TP53 CCR4 LTBR EIF2AK2 PIK3R3	1.38
hsa04911	Insulin secretion	2/146	91/8877	0.387	0.5503	KCNN3 ADCY2	1.34
hsa04962	Vasopressin-regulated water reabsorption	1/146	46/8877	0.3555	0.5144	DYNC2LI1	1.32
hsa04360	Axon guidance	4/146	186/8877	0.4	0.5591	CXCR4 WNT4 GSK3B PIK3R3	1.31
hsa04151	PI3K-Akt signaling pathway	8/146	375/8877	0.3584	0.5156	PGF TP53 AKT2 ITGA3 F2R GSK3B PIK3R3 IFNAR1	1.3
hsa05410	Hypertrophic cardiomyopathy (HCM)	2/146	94/8877	0.4122	0.5729	CACNB2 ITGA3	1.29
hsa04371	Apelin signaling pathway	3/146	141/8877	0.4177	0.5677	AKT2 SMAD2 ADCY2	1.29
hsa04380	Osteoclast differentiation	4/146	192/8877	0.4346	0.5874	AKT2 MAPK12 PIK3R3 IFNAR1	1.27
hsa04340	Hedgehog signaling pathway	1/146	48/8877	0.3791	0.5422	GSK3B	1.27
hsa04975	Fat digestion and absorption	1/146	49/8877	0.391	0.5528	PLPP2	1.24

hsa00565	Ether lipid metabolism	1/146	49/8877	0.391	0.5528	PLPP2	1.24
hsa04540	Gap junction	2/146	98/8877	0.4462	0.5998	MAP3K2 ADCY2	1.24
hsa04727	GABAergic synapse	2/146	100/8877	0.4634	0.6195	GABRB3 ADCY2	1.22
hsa04144	Endocytosis	6/146	305/8877	0.4952	0.6514	VPS28 GRK5 CXCR4 SMAD2 EPN1 RAB22A	1.2
hsa04961	Endocrine and other factor-regulated calcium reabsorption	1/146	51/8877	0.4149	0.5734	ATP2B1	1.19
hsa04913	Ovarian steroidogenesis	1/146	51/8877	0.4149	0.5734	ADCY2	1.19
hsa04979	Cholesterol metabolism	1/146	51/8877	0.4149	0.5734	LRPAP1	1.19
hsa04070	Phosphatidylinositol signaling system	2/146	105/8877	0.5069	0.6633	DGKZ PIK3R3	1.16
hsa04390	Hippo signaling pathway	3/146	160/8877	0.551	0.7097	WNT4 SMAD2 GSK3B	1.14
hsa05152	Tuberculosis	5/146	269/8877	0.5814	0.7411	CASP8 AKT2 MAPK12 IRAK4 CTSS	1.13
hsa05012	Parkinson disease	3/146	161/8877	0.5582	0.7152	COX6B2 NDUFA10 COX5B	1.13
hsa04218	Cellular senescence	6/146	332/8877	0.6301	0.7868	TP53 AKT2 MAPK12 SMAD2 CHEK2 PIK3R3	1.1
hsa04810	Regulation of actin cytoskeleton	4/146	221/8877	0.6116	0.7755	CXCR4 ITGA3 F2R PIK3R3	1.1
hsa04930	Type II diabetes mellitus	1/146	56/8877	0.4752	0.6319	PIK3R3	1.09
hsa00240	Pyrimidine metabolism	1/146	57/8877	0.4874	0.6446	DCTD	1.07
hsa04310	Wnt signaling pathway	3/146	172/8877	0.6383	0.793	TP53 WNT4 GSK3B	1.06
hsa05168	Herpes simplex infection	6/146	350/8877	0.7231	0.8894	CCL5 CASP8 TP53 OAS3 EIF2AK2 IFNAR1	1.04
hsa04022	cGMP-PKG signaling pathway	3/146	175/8877	0.6603	0.8163	ATP2B1 AKT2 ADCY2	1.04
hsa04928	Parathyroid hormone synthesis, secretion and action	2/146	117/8877	0.613	0.7733	PDE4C ADCY2	1.04
hsa03022	Basal transcription factors	1/146	59/8877	0.5116	0.6659	TAF8	1.03
hsa01200	Carbon metabolism	2/146	118/8877	0.6219	0.7805	CS H6PD	1.03
hsa00480	Glutathione metabolism	1/146	60/8877	0.5238	0.6782	GCLC	1.01
hsa04080	Neuroactive ligand-receptor interaction	6/146	362/8877	0.7856	0.9615	GABRB3 F2R SSTR2 CALCRL CNR2 P2RX4	1.01
hsa04640	Hematopoietic cell lineage	3/146	185/8877	1	1	CSF2RA ITGA3 CD3G	0.99
hsa00970	Aminoacyl-tRNA biosynthesis	1/146	62/8877	1	1	MTFMT	0.98
hsa05130	Pathogenic Escherichia coli infection	1/146	63/8877	1	1	KRT18	0.97
hsa04141	Protein processing in endoplasmic reticulum	3/146	190/8877	1	1	SEC62 ATXN3 EIF2AK2	0.96
hsa04659	Th17 cell differentiation	3/146	195/8877	1	1	MAPK12 SMAD2 CD3G	0.94
hsa04152	AMPK signaling pathway	2/146	129/8877	1	1	AKT2 PIK3R3	0.94
hsa05202	Transcriptional misregulation in cancer	3/146	195/8877	1	1	TP53 NCOR1 ARNT2	0.94
hsa04114	Oocyte meiosis	2/146	129/8877	1	1	MAPK12 ADCY2	0.94
hsa00310	Lysine degradation	1/146	65/8877	1	1	KMT2D	0.94
hsa05131	Shigellosis	1/146	66/8877	1	1	MAPK12	0.92
hsa04140	Autophagy - animal	2/146	133/8877	1	1	AKT2 PIK3R3	0.91
hsa04270	Vascular smooth muscle contraction	2/146	136/8877	1	1	CALCRL ADCY2	0.89
hsa04726	Serotonergic synapse	2/146	137/8877	1	1	GABRB3 APP	0.89
hsa00230	Purine metabolism	2/146	137/8877	1	1	PDE4C ADCY2	0.89
hsa04137	Mitophagy - animal	1/146	73/8877	1	1	TP53	0.83
hsa00140	Steroid hormone biosynthesis	1/146	74/8877	1	1	LRTOMT	0.82
hsa01230	Biosynthesis of amino acids	1/146	77/8877	1	1	CS	0.79
hsa05134	Legionellosis	1/146	77/8877	1	1	CASP8	0.79
hsa04927	Cortisol synthesis and secretion	1/146	77/8877	1	1	ADCY2	0.79
hsa05100	Bacterial invasion of epithelial cells	1/146	77/8877	1	1	PIK3R3	0.79
hsa04014	Ras signaling pathway	3/146	244/8877	1	1	PGF AKT2 PIK3R3	0.75
hsa05140	Leishmaniasis	2/146	165/8877	1	1	MAPK12 IRAK4	0.74
hsa04918	Thyroid hormone synthesis	1/146	82/8877	1	1	ADCY2	0.74
hsa04658	Th1 and Th2 cell differentiation	2/146	177/8877	1	1	MAPK12 CD3G	0.69
hsa04514	Cell adhesion molecules (CAMs)	3/146	269/8877	1	1	ICOSLG SPN PTPRM	0.68
hsa04721	Synaptic vesicle cycle	1/146	90/8877	1	1	ATP6V1A	0.68
hsa04530	Tight junction	2/146	181/8877	1	1	WHAMM MARVELD2	0.67
hsa04512	ECM-receptor interaction	1/146	98/8877	1	1	ITGA3	0.62
hsa04713	Circadian entrainment	1/146	100/8877	1	1	ADCY2	0.61
hsa04350	TGF-beta signaling pathway	1/146	99/8877	1	1	SMAD2	0.61
hsa05323	Rheumatoid arthritis	2/146	202/8877	1	1	CCL5 ATP6V1A	0.6
hsa04610	Complement and coagulation cascades	1/146	101/8877	1	1	F2R	0.6
hsa05132	Salmonella infection	1/146	103/8877	1	1	MAPK12	0.59
hsa05146	Amoebiasis	1/146	106/8877	1	1	PIK3R3	0.57
hsa05206	MicroRNAs in cancer	3/146	319/8877	1	1	ZFPM2 TP53 MDM4	0.57
hsa04724	Glutamatergic synapse	1/146	118/8877	1	1	ADCY2	0.52
hsa03008	Ribosome biogenesis in eukaryotes	1/146	118/8877	1	1	FCF1	0.52
hsa05321	Inflammatory bowel disease (IBD)	1/146	154/8877	1	1	SMAD2	0.39

hsa04145	Phagosome	2/146	315/8877	1	1	ATP6V1A CTSS	0.39
hsa03040	Spliceosome	1/146	176/8877	1	1	SRSF10	0.35
hsa05416	Viral myocarditis	1/146	177/8877	1	1	CASP8	0.34
hsa03013	RNA transport	1/146	187/8877	1	1	SUMO2	0.33
hsa04650	Natural killer cell mediated cytotoxicity	2/146	479/8877	1	1	PIK3R3 IFNAR1	0.25
hsa04612	Antigen processing and presentation	2/146	550/8877	1	1	CTSS LGMN	0.22
hsa04740	Olfactory transduction	1/146	586/8877	1	1	OR7D2	0.1
